# A Comparative Study of Gene-Expression Data of Basal Cell Carcinoma and Melanoma Reveals New Insights about the Two Cancers

**DOI:** 10.1371/journal.pone.0030750

**Published:** 2012-01-25

**Authors:** Kun Xu, Xizeng Mao, Minesh Mehta, Juan Cui, Chi Zhang, Ying Xu

**Affiliations:** 1 Department of Biochemistry and Molecular Biology and Institute of Bioinformatics, University of Georgia, Athens, Georgia, United States of America; 2 Department of Statistics, University of Georgia, Athens, Georgia, United States of America; 3 College of Medicine, American University of Antigua, New York, New York, United States of America; 4 College of Computer Science and Technology, Jilin University, Changchun, Jilin, China; Memorial Sloan Kettering Cancer Center, United States of America

## Abstract

A comparative analysis of genome-scale transcriptomic data of two types of skin cancers, melanoma and basal cell carcinoma in comparison with other cancer types, was conducted with the aim of identifying key regulatory factors that either cause or contribute to the aggressiveness of melanoma, while basal cell carcinoma generally remains a mild disease. Multiple cancer-related pathways such as cell proliferation, apoptosis, angiogenesis, cell invasion and metastasis, are considered, but our focus is on energy metabolism, cell invasion and metastasis pathways. Our findings include the following. (a) Both types of skin cancers use both glycolysis and increased oxidative phosphorylation (electron transfer chain) for their energy supply. (b) Advanced melanoma shows substantial up-regulation of key genes involved in fatty acid metabolism (β-oxidation) and oxidative phosphorylation, with aerobic metabolism being far more efficient than anaerobic glycolysis, providing a source of the energetics necessary to support the rapid growth of this cancer. (c) While advanced melanoma is similar to pancreatic cancer in terms of the activity level of genes involved in promoting cell invasion and metastasis, the main metastatic form of basal cell carcinoma is substantially reduced in this activity, partially explaining why this cancer type has been considered as far less aggressive. Our method of using comparative analyses of transcriptomic data of multiple cancer types focused on specific pathways provides a novel and highly effective approach to cancer studies in general.

## Introduction

The rapidly increasing pool [1], [2] of large-scale transcriptomic data for various cancer types has provided unprecedented opportunities for computational cancer biologists to study common characteristics across multiple cancer types as well as distinct properties of individual cancer types, which could provide novel insights about different cancer phenotypes at the molecular level. Here we present a comparative analysis of transcriptomic data collected on cancer and control tissue samples of two skin cancer types, melanoma and basal cell carcinoma, which have very distinct characteristics.

Skin cancer is one of the most common cancer types in the USA. Currently over 3.5 million cases of skin cancers are diagnosed and reported annually [3]. It has been estimated that three out of ten Caucasians will develop skin cancer during their lifetime [4]. The most common skin cancer is basal cell carcinoma (BCC), which develops in the basal cell layer of the skin, and primarily occurs in fair-skinned individuals. Sunlight is known to be a major factor for causing the disease. BCC is rarely deadly since it generally does not metastasize [5]. In contrast, melanoma is a rare type of skin cancer but is among the deadliest forms of cancers [6]. The tumor is derived from melanocytes, cells that produce the dark pigment. While melanoma is not limited to skin, it generally starts from the skin. A number of genes or their mutations have been found to be associated with the development of melanoma such as MC1R [7], CDK4 [8] and CDKN2A [9]. The early stage of the disease is referred to as the *radial growth phase* when the tumor grows mostly horizontally. The behavior of the tumor drastically changes as soon as it starts to grow vertically, i.e., entering the *vertical growth phase*. It generally starts invading neighboring tissues when its thickness goes beyond 1 mm [10].

While some information is known about the potential causes of the two skin cancer types, such as excessive exposure to sunlight and development of the basal-cell nevus syndrome being the main causes of basal cell carcinoma and a few rare mutations in the aforementioned genes being the main reason for the development of melanoma, a detailed understanding about why the two skin cancer types behave so differently remains to be very limited.

Through comparative analyses of genome-scale transcriptomic data on the two cancer types, we have gained a number of new insights which could shed new lights on our efforts to understand the detailed mechanisms of these two rather different skin cancer types. To put our analysis in a larger context, seven other cancer types have also been included, which range from relatively slow growing cancer to the fastest growing cancers, i.e, prostate, breast, kidney, colon, stomach, lung and pancreatic. By using transcriptomic data collected on cancer *versus* control tissues and comparing expression changes of the genes involved in different pathways associated with energy metabolism, we found that: (i) multiple genes involved in oxidative phosphorylation are up-regulated in both melanoma and BCC, which is unique to only skin cancers among the nine types of cancer we examined and is inconsistent with Warburg's thesis [11]; (ii) interestingly, the key enzyme in ATP generation in the oxidative phosphorylation pathway is up-regulated only in advanced melanoma but not in any form of BCC; (iii) the level and scale of up-regulated genes involved in cell invasion and metastasis in advanced melanoma are comparable to those of pancreatic cancer, while the corresponding values in BCC are essentially at the lower end among all the nine cancer types we examined. We believe that our comparative transcriptomic data analyses of multiple cancer types focused on specific cancer related pathways provide a novel and highly effective approach to cancer studies, which could lead to substantial new insights about cancer formation (when the relevant data are available) and progression.

## Results

Our analysis was done on two gene-expression datasets. One set consisted of 52 tissue samples for the study of melanoma. Of the 52 tissue samples, 18 were common nevi (moles) (CMN), 11 were dysplastic nevi (pre-cancerous) (DN), 8 in the radial growth phase (RGP) (early stage) and 15 in the vertical growth phase (VGP) (advanced stage) [12]. For this particular dataset, the common nevi tissues were used as the control set since the original study did not include normal skin tissues [12]. The second set consisted of 31 tissues for the study of BCC. Of the 31 tissue samples, 8 were in the superficial form (early stage), 7 in the morphea form (intermediate stage) and 8 in the nodular form (advanced stage), along with 8 normal skin epithelial tissues as the control [13].

### 1. Differentially expressed genes in the two skin cancer types

In this study, a gene is considered *differentially* expressed at a specific stage of a cancer if the distribution of its expression levels among cancer tissues at that stage is deemed to be statistically different from the distribution of its expression levels among the control tissues (see Material and Methods). For BCC, 158, 406 and 494 genes were found to be differentially expressed in the superficial, morphea and nodular forms, respectively, compared to the controls, which is consistent with our previous observation that the number of differentially expressed genes increases as a cancer advances [14], [15]. Using the same cutoff, 123, 326 and 1,647 genes were deemed to be differentially expressed in DN, RGP and VGP melanoma. In our previous study, we found that there is a strong correlation between the number of differentially expressed genes and the five-year survival rate associated with a particular cancer [14]. Thus, the high number of differentially expressed genes in VGP melanoma is consistent with the clinical statistics regarding the mortality rate of this cancer. There is a possibility that this number could be potentially under-estimated since the controls (moles) for the melanoma analysis are not normal skin tissues and moles are probably the first step moving towards melanoma. The detailed lists of differentially expressed genes are given in Table S1. Overall it was found that the numbers of differentially expressed genes in the two cancer types are comparable in their early stages; and a substantial rise in the number of differentially expressed genes in the advanced stage, VGP, of melanoma was observed.

A careful analysis of the pathways in the KEGG database that are enriched by the differentially expressed genes among the cancer tissues of BCC and melanoma was conducted with the DAVID program (see Material and Methods). For BCC, no pathways were found to be clearly enriched in the early stage, while 19 and 18 pathways were enriched in the intermediate and advanced stages, respectively. For melanoma, 1, 8 and 61 pathways were enriched in the DN, RGP and VGP forms, respectively. The names of these enriched pathways are given in Table S2.

It was noted that 11 enriched pathways are specific to VGP melanoma, the deadliest form among all the skin cancer types, including pathways associated with amino sugar and nucleotide sugar metabolism, linoleic acid metabolism and the citratric acid cycle (TCA cycle). In addition, some pathways are significantly enriched in only melanoma among the two skin cancer types considered, such as fatty acid metabolism, cell cycle, apoptosis and the ErbB signaling pathway. For BCC, it was noted that its advanced stage cancer has three pathways uniquely enriched among all the cancer types under consideration, including the spliceosome, GnRH signaling and long-term potentiation pathways.

We have also checked if some of the annotated proto-oncogene and tumor-suppressor genes (http://www.uniprot.org/keywords/) show differential expressions in BCC and melanoma. Overall it was found that 2, 5 and 4 oncogenes are over-expressed in early, intermediate and advanced stage of BCC samples, respectively, and 0, 1 and 0 tumor suppressor genes are respectively under-expressed in the three stages. Similarly, for melanoma, 9, 1 and 32 oncogenes are over-expressed in precancerous, early stage and advanced melanoma, respectively, and 0, 4 and 4 tumor suppressor genes are respectively under-expressed in the three stages.

Some of these up-regulated oncogenes have been reported as key regulatory genes for some of the skin cancer types. Among the up-regulated oncogenes in melanoma, ABL2, NRAS, PDGFC and FGF1 have been reported to be melanoma-associated oncogenes [16]. Thus, RAB6B, REL and WHSC1L1, as identified by our analysis, may represent additional oncogenes for melanoma. For BCC, HRAS, RRAS and RUNX1 have been reported as BCC-associated oncogenes [17]. Hence, ECT2, PLAG1, RAB6C and SSPN, identified by our analysis, may represent additional oncogenes for BCC. It is interesting that nine oncogenes exhibit up-regulation in the pre-cancer stage of melanoma, which may be the initial switch of the tumorgenesis leading to melanoma. Moreover, the large increase in the number of up-regulated oncogenes in VGP melanoma may suggest the aggressiveness of the cancer. A detailed list of all the identified oncogenes and tumor-suppressor genes is given in Figure S1.

### 2. Differentially expressed genes involved in energy metabolism

**Figure 1:**
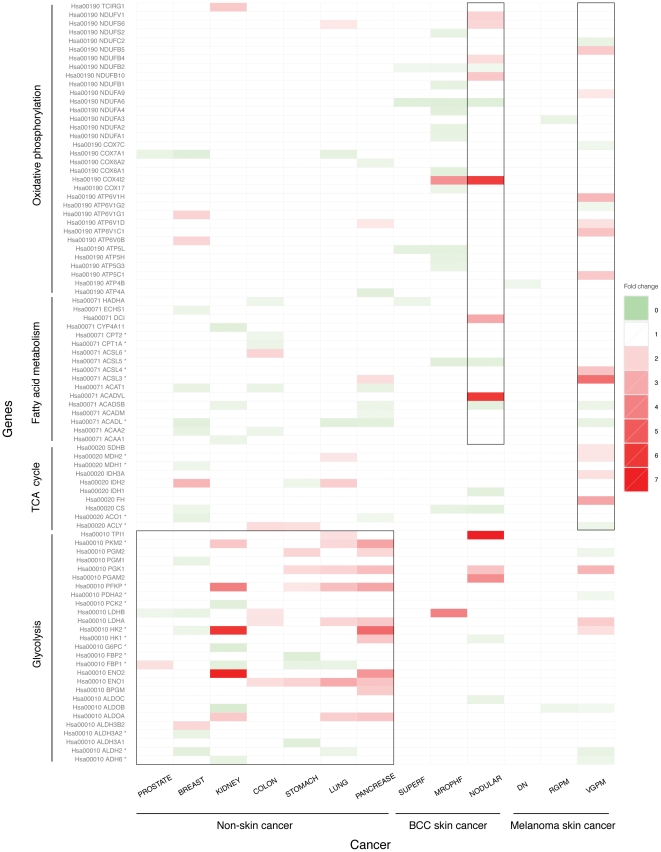
Expression level changes of genes involved in the four types of energy metabolism for two skin cancer types and seven non-skin cancer types. Each row represents expression changes of a gene across all the cancer types under study. Rate limiting enzymes in each pathway are indicated using *. Each column represents one cancer type. The fold change of gene expression is color-coded with red, white and green for up-, no and down-regulation.

We have examined expression fold-changes of genes involved in four energy metabolic pathways: glycolysis, fatty acid metabolism, the TCA cycle and oxidative phosphorylation (also called the *electron transfer chain*) in the two skin cancer types and compared them with the other seven non-skin cancer types. Figure 1 shows expression level changes of genes involved in the four energy pathways of the two skin cancer types and the other seven cancer types. By examining the figure, the following observations can be made.

1. Substantial increases in expression levels of multiple genes involved in glycolysis are observed in both the advanced forms of the two skin cancer types and five of the seven reference cancer types, namely kidney, colon, stomach, lung and pancreatic, consistent with Warburg's thesis [18].
2. Two enzyme-encoding genes involved in fatty acid metabolism show substantial up-regulation in both BCC and melanoma, in contrast to the seven non-skin cancer, indicating a unique way that skin cancer obtains energy not only from glucose metabolism like other cancers but also from fatty acid metabolism, which is a more efficient energy generation pathway.
3. Moderately increased expression levels of genes involved in the TCA cycle are also observed in advanced melanoma, along with breast, colon, stomach and lung cancers.
4. Most interesting is the finding that multiple genes involved in oxidative phosphorylation are up-regulated in the advanced form of both skin cancer types, which strongly suggests that both skin cancer types obtain much of their energy through oxidative phosphorylation, which produces an order of magnitude more ATPs than each of the other three energy pathways (per glucose). This is very surprising as this indicates the two skin cancer types, even in their advanced forms, are not under hypoxic condition, and hence do not show the Warburg effect.
5. ATP synthase is up-regulated only in advanced melanoma, in addition to the protein complex responsible for electron transfer but not in any form of BCC, indicating that ATP synthesis is faster in advanced melanoma than BCC.

**Figure 2:**
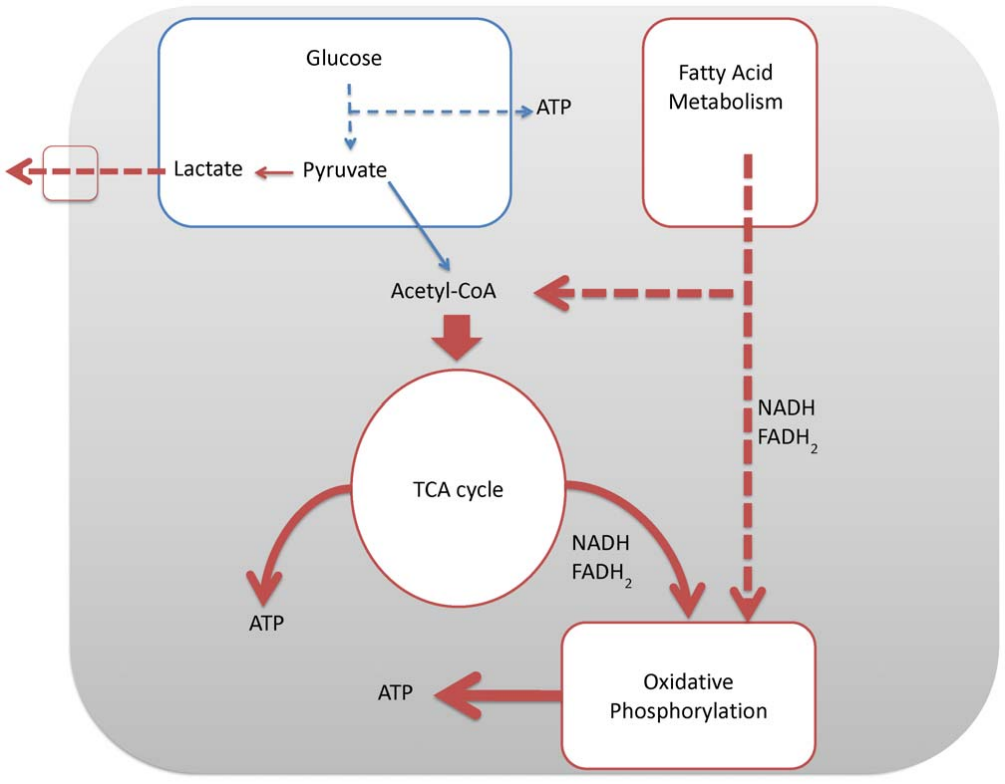
A model for energy metabolism for VGP melanoma.

Further analysis of the differentially expressed genes in advanced melanoma suggests that the increased levels of acetyl-CoA, NADH and FADH_2_ derived from fatty acid oxidation can enter mitochondria and undergo oxidative phosphorylation. This process would be highly advantageous for a tumor as β-oxidation of fatty acids yields a larger number of acetyl-CoA molecules compared to glycolysis, and thus it has a larger number of substrates for the TCA cycle and subsequent oxidative phosphorylation. In order to utilize the increased number of acetyl-CoAs, the cells may need to have an increased rate of oxidative phosphorylation. Acetyl-CoA is an allosteric inhibitor of the PDH enzymes. As we observed, PDHA2 is substantially down-regulated in VGP melanoma, which can be attributed to the increased level of acetyl-CoA. As the PDHA2 activity decreases, pyruvate will naturally be diverted to lactic acid formation, which is shown by the up-regulation of LDHA and lactate transporters (MCT proteins SLC16As 3&6). Figure 2 gives an energy model for advanced melanoma based on the results of our data analysis.

### 3. Differentially expressed genes involved in tumor invasion and metastasis

**Figure 3:**
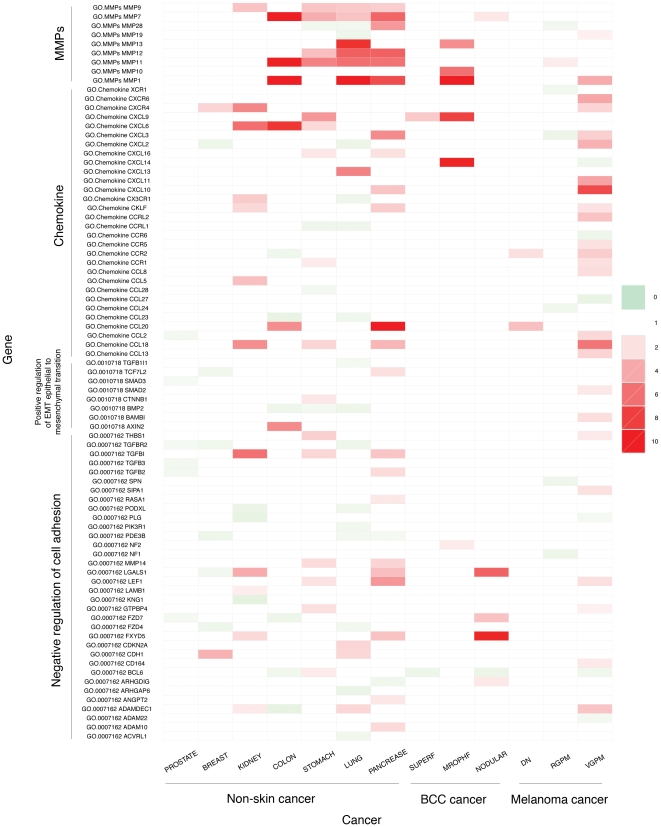
Expression changes of genes involved in the pro-metastasis of the two skin cancer types and other seven non-skin cancer types.

We have studied expression changes of genes involved in the metastatic process, with the goal of identifying possible reasons why the two skin cancer types have substantial differences in their ability to metastasize. For this, focus was placed on the pro-metastasis gene families, namely the positive regulation of the epithelial-mesenchymal transition (EMT), the negative regulation of cell adhesion, the chemokine and MMP families, both of which promote degradation of extracellular matrices. Figure 3 shows the observed expression changes of genes involved in these processes of the two skin cancer types, along with the other seven cancer types. We have made the following observations from the data in Figure 3.

**Figure 4:**
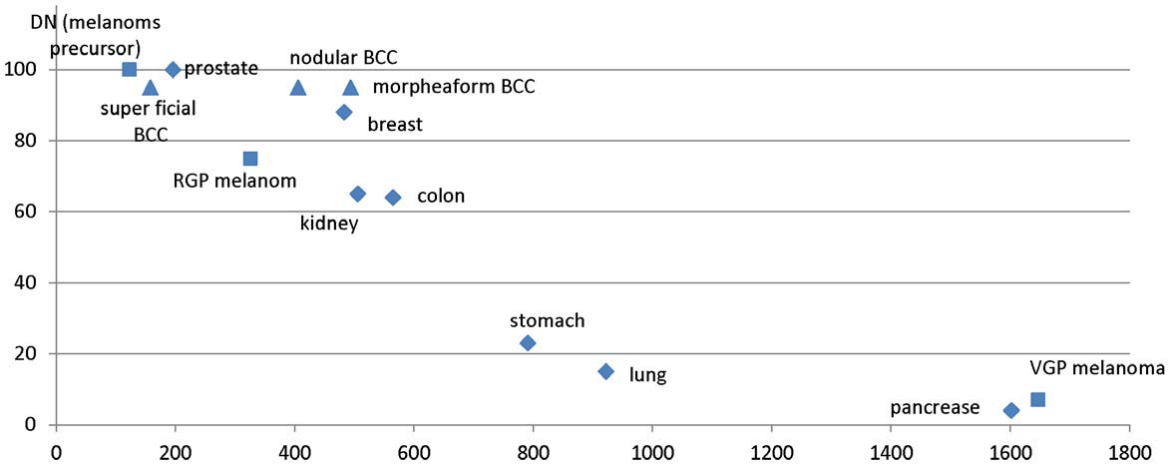
Correlation between 5-year survival rate and the number of differentially genes in each cancer type using the same statistical significance cutoff (see Material and Methods). The x-axis is the five-year survival rate ranging from 0 to 100% (data used from www.cancer.org), and the y-axis is the number of differentially expressed genes for each cancer type.

1. The VGP melanoma has the greatest number of up-regulated genes involved in the pro-metastasis gene family, having even more such genes than pancreatic cancer; While simply counting the number of up-regulated genes may be a rather crude way to assess the ability of a cancer to metastasize, Figure 4 shows that there is a strong (negative) correlation between this number and the five-year survival rate of a cancer.
2. VGP melanoma is the only skin cancer type with up-regulated genes involved in positive regulation of the epithelial-mesenchymal transition, which is considered to be the crucial developmental and regulatory program for cell invasion and metastasis [19], [20]; in addition, the significant up-regulation of genes involved in degrading the cell-cell adhesion molecules and the increased negative regulation of the cell adhesion, the matrix metalloproteinases and chemokines all suggest that VGP is highly metastatic [21].
3. The lymphatic spread represents a major way for metastasis. We noted that the number of differentially expressed genes in lymphocyte proliferation is much higher in VGP melanoma compared to all the other skin cancer subtypes under consideration (see Figure S2).
4. It has been reported that a chaotic circadian rhythm may lead to faster tumor growth [22]. From the above figure, we noted that key genes of the circadian rhythm pathway are differentially expressed in VGP melanoma but only to a very limited extent in other skin cancer subtypes under consideration. CRY2, the most important gene controlling cell circadian rhythm [23], shows down-regulation only in VGP melanoma among all the cancer types under consideration. Items (2)–(4) above suggest that VGP melanoma has the greatest activities for cell-invasion and metastasis.
5. In contrast, only a few genes of BCC are up-regulated in the aforementioned processes. Among the two different BCC subtypes, it is the morphea form not the nodular form, that has the most up-regulated genes, which is consistent with previous reports that this form represents the BCC form with the greatest number of metastasis cases [24]. We observed that in the morphea BCC, a number of genes encoding the collagens and proteins involved in anti-metastasis are highly up-regulated, suggesting that the cancer is under the control of the immune system in inhibiting its invasion and metastasis throughout its development.

Overall our analysis suggests that VGP melanoma has the highest potential and unique ability to metastasize among the cancer types studied herein, while the BCC's ability to metastasize is the weakest among the nine types of cancers under consideration.

### 4. Differentially expressed genes involved in other cancer-related processes

#### 4a. Differentially expressed genes involved in cell proliferation

Self-sufficiency in growth signals is a major acquired capability of any cancer. Our analysis of differentially expressed genes involved in positive regulation of cell proliferation indicates that VGP melanoma has substantially more genes up-regulated in this category than all the other skin cancer forms. Specifically, we noted that the number of such up-regulated genes is comparable to that of the pancreatic cancer with the detailed data given in Figure S3. Particularly worth noting is that VGP melanoma is the only cancer type among the nine cancer types showing substantial up-regulation of the genes of the Jak-STAT signaling pathway, which is a crucial pathway that promotes cell growth.

#### 4b. Differentially expressed genes involved in apoptosis

The morphea BCC, nodular BCC and VGP melanoma all have numerous numbers of differentially expressed genes involved in negative regulation of cell death as shown in Figure S4. From this figure we can see an increasing trend in the number of up-regulated genes as the cancer type becomes more aggressive. Specifically it was noted that this number for VGP is higher than all the other cancer types under consideration except for the pancreatic cancer.

#### 4c. Differentially expressed genes involved in angiogenesis

**Figure 5:**
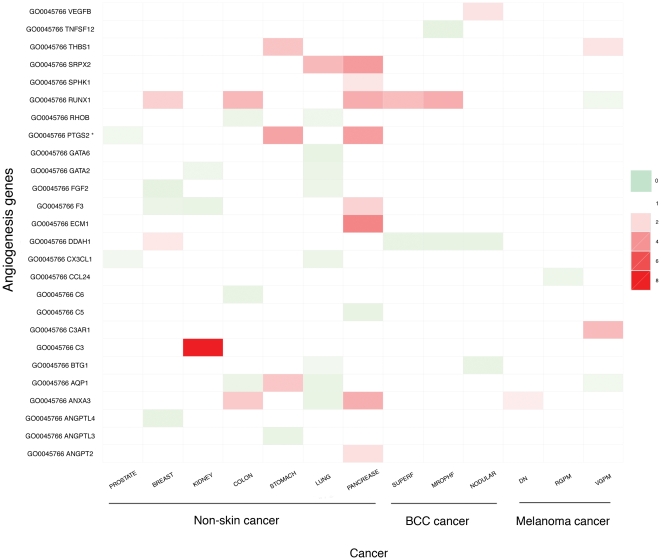
Expression changes of genes involved in the pro-angiogenesis of the two skin cancer types and other seven non-skin cancer types.

None of the skin cancer types show increased activities of angiogenesis, unlike the advanced form of the other cancers as shown in Figure 5. This observation is consistent with our earlier finding that skin cancers are generally not under hypoxic stress and hence probably have no pressure to activate the angiogenesis pathway.

### 5. Signature genes for the two skin cancer types

We have predicted signature genes and gene groups for melanoma and BCC, respectively, where a *signature* (or *marker*) gene or gene group refers to genes whose expression pattern is unique to a specific cancer type. We then tested the performance of the identified signature genes for the two cancer types, respectively, on two datasets, independent of the training datasets used, i.e., GSE3189 for melanoma and GSE12542 for BCC. For melanoma, the top five 1-gene signatures, OAS3 (82.9% on training and 71.4% on testing set), RALBP1 (82.9% on training and 71.4% on testing set), GLA (80.4% on training and 71.4% on testing set), LLGL1 (80.4% on training and 71.4% on testing set) and SERPINA6 (80.4% on training and 71.4% on testing set), all have better than 80% classification accuracy between melanoma cases and control cases in the test set. Among the top markers, OAS3 and SERPINA6 are predicted to be blood secretory by our prediction program [25], hence suggesting the potential feasibility in identifying diagnostic markers for melanoma through blood tests. Similarly, four 1-gene signatures with classification accuracy better that 71% are predicted to be urine excretory, CCL18 (73.2% on training and 71.4% on testing set), HEXB (73.2% on training and 71.4% on testing set), IFI30 (73.2% on training and 71.4% on testing set) and STC1 (73.2% on training and 71.4% on testing), by using our prediction program [26], suggesting the potential feasibility in identifying diagnostic markers for melanoma through urine tests. Among the top 2-gene signatures, three pairs reach classification accuracy better than 85% on both the training and the testing datasets. Three pairs, with training classification accuracies better than 90% and testing classification accuracy better than 70%, are predicted to be blood secretory and only 1 pair CTSK_RNASE6 (87.8% on training and 73.0% on testing sets), with training classification accuracy better than 87% and testing classification accuracy better than 70%, are predicted to be urine excretory.

For BCC, the top two 1-gene markers, CS (90.3% on training and 87% on testing set) and TACSTD1 (87.1% on training and 87% on testing dataset), both have at least 87% classification accuracy on both the training and testing datasets. Among the top markers, EGR1 (87.1% on training and 81.3% on testing set) is predicted to be blood secretory. Similarly, RAB3D (80.6% on training and 80% on testing dataset) is predicted to be urine excretory, with a classification accuracy better that 80% in both sets, providing potential diagnostic markers for melanoma through urine tests. The top two 2-gene signatures all have classification accuracies better than 90% on both the training and testing sets. Also, four pair signatures, with classification accuracy better than 80% on both the training and testing sets, are predicted to encode blood secretory proteins. The detailed list of all these marker genes is given in Table S3.

## Materials and Methods

### 1. Microarray gene expression data for nine cancer types

Microarray gene expression data for both skin cancer types were downloaded from the GEO database of NCBI [27]. The melanoma data is the dataset GSE12391 and the BCC data is the dataset GSE6520. The gene-expression data for the seven cancer types used in this study: breast, colon, kidney, lung, pancreatic, prostate and stomach, are also downloaded from the GEO database of NCBI. For each dataset used for each cancer type, we have made sure that the dataset was generated using the same platform by the same research group. For each of the classification problem solved in this study, we have used a training dataset and a separate testing set for each cancer type. The details of the data are given in Table S4.

Considering that different microarray datasets used in this study cover different gene sets, we have considered the genes that belong to all microarray datasets used in this study for all the comparative analyses throughout this paper, which consists of 4,401 genes. The detailed list of these genes is given in Table S5. When mapping genes across different datasets, we rely on the NCBI gene IDs of the genes, i.e., two genes in different datasets are considered as the same genes if their IDs are identical.

The cancer gene list is downloaded from the Cancer Gene Census website, which contains 457 confirmed cancer genes (http://www.sanger.ac.uk/genetics/CGP/Census/).

### 2. Identification of differentially expressed genes

For each dataset used in this study, we have used the normalized expression data from the original study. We fully understand that gene-expression data across different datasets may not necessarily directly comparable; so we have compared the fold-changes between the diseased and the control tissues for each cancer type with fold-changes of expression data of another cancer type. Figure S5 shows that the global fold-changes between two cancer types are generally comparable across the nine cancer types under consideration. It should be noted that when calculating the fold-change of an individual gene for a specific cancer type, we did not use the information of paired diseased-control tissues, instead we estimated the fold change based on the distributions of gene-expressions of the gene across all the cancer tissues versus control tissues, for each cancer type. We did so because some of the datasets have paired information wile other datasets do not.

For each dataset, the Mann-Whitney test was applied to identify genes that are differentially expressed in cancer *versus* control samples as follows: Given the null hypothesis 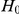 that a gene is not differentially expressed between the cancer *versus* the control groups, rejection of this hypothesis means that the gene is differentially expressed in cancer. We consider a gene as *up-regulated* if the statistical significance, *p*-value, is less than 0.01 and its fold-increase is at least 1.5. A *down-regulated* gene is defined similarly.

For the non-skin cancer data, we only consider those genes with consistent up/down-regulation in both the training and the testing data sets as differentially expressed genes.

### 3. Pathway enrichment analysis of differentially expressed genes

Functional analysis and pathway enrichment analysis were conducted using DAVID [28], where the pathway information is based on the annotation from KEGG (http://www.genome.ad.jp/kegg/). A *p*-value<0.05 was used as the threshold to determine if a pathway is enriched or not by the identified differentially expressed genes. Note that the observations made throughout this paper are generally stable with respect to the p-value cutoff. Also note all the pathway enrichment analysis is based on the 4,401 genes that are shared by all the datasets used in this study.

### 4. Prediction of signature genes

To derive signature genes or gene groups, we conducted an exhaustive search for all the k-gene (k = 1, 2) combinations among the differentially expressed genes, using a linear SVM-based classifier. We have used 5-fold cross validation to validate each identified signature. We refer the reader to [29] for the detailed procedure used.

## Discussion

Our gene-expression data analysis revealed that both skin cancer types utilize oxidative phosphorylation as the key energy metabolism in addition to glycolysis. All evidence revealed by our study strongly indicates that the two skin cancer types are not under hypoxic stress, hence explaining why the two cancer types do not show the Warburg effect. The high energy metabolism in melanoma, powered by the substantially more efficient energy pathway, the up-regulated oxidative phosphorylation compared to the alternatives, along with its high ability to metastasize, explained why the cancer is so aggressive. In contrast, BCC, while using energy from oxidative phosphorylation, seems to have an obvious block from the immune system, which appears to prevent cell invasion and metastasis, hence making the cancer one of the least deadly cancers.

The new insights derived in this study are global in nature and lack of detailed mechanism information due to the low-resolution nature of the non-paired datasets used in this study. We anticipate that higher-resolution insights could be derived using the same computational approach but on paired cancer-control datasets.

We believe that our study provides a new and highly effective way to gain new insights and understanding about certain unique characteristics of different cancers in their formation and progression through mining large-scale gene-expression data across multiple cancer types, but focused on key relevant cancer pathways.

## Supporting Information

Figure S1 Expression level changes of proto-oncogene and tumor-suppressor genes for two skin cancer types.
(PDF)

Figure S2 Expression level changes of genes involved in the positive regulation of lymphocyte proliferation for two skin cancer types and seven non-skin cancer types.
(PDF)

Figure S3 Expression level changes of genes involved in the positive regulation of cell proliferation for two skin cancer types and seven non-skin cancer types.
(PDF)

Figure S4 Expression level changes of genes involved in the negative regulation of cell death for two skin cancer types and seven non-skin cancer types.
(PDF)

Figure S5 Comparison of the gene expression fold change.
(PDF)

Table S1 Differentially expressed genes in skin cancer.
(PDF)

Table S2 The enriched pathways by all the cancer types in the study.
(PDF)

Table S3 The top gene signatures for the skin cancer.
(PDF)

Table S4 A summary of the cancer data used in our analysis.
(PDF)

Table S5 The common genes shared by the datasets used in this study.
(PDF)
