# Supplementary material for: A Comparative Study of Gene-Expression Data of Basal Cell Carcinoma and Melanoma Reveals New Insights about the Two Cancers
Source: PLoS One. 2012 Jan 25;7(1):e30750. doi: 10.1371/journal.pone.0030750 (PMC3266277; doi:10.1371/journal.pone.0030750)

Figure S1: Expression level changes of proto-oncogene and tumor-suppressor genes for two skin cancer types. We only consider the up-regulated proto-oncogene and down-regulated tumor-suppressor genes. Each row represents expression changes of a gene across all the cancer types under study. Each column represents one cancer type. The fold change of gene expression is color-coded with red, white and green for up-, no and down-regulation.

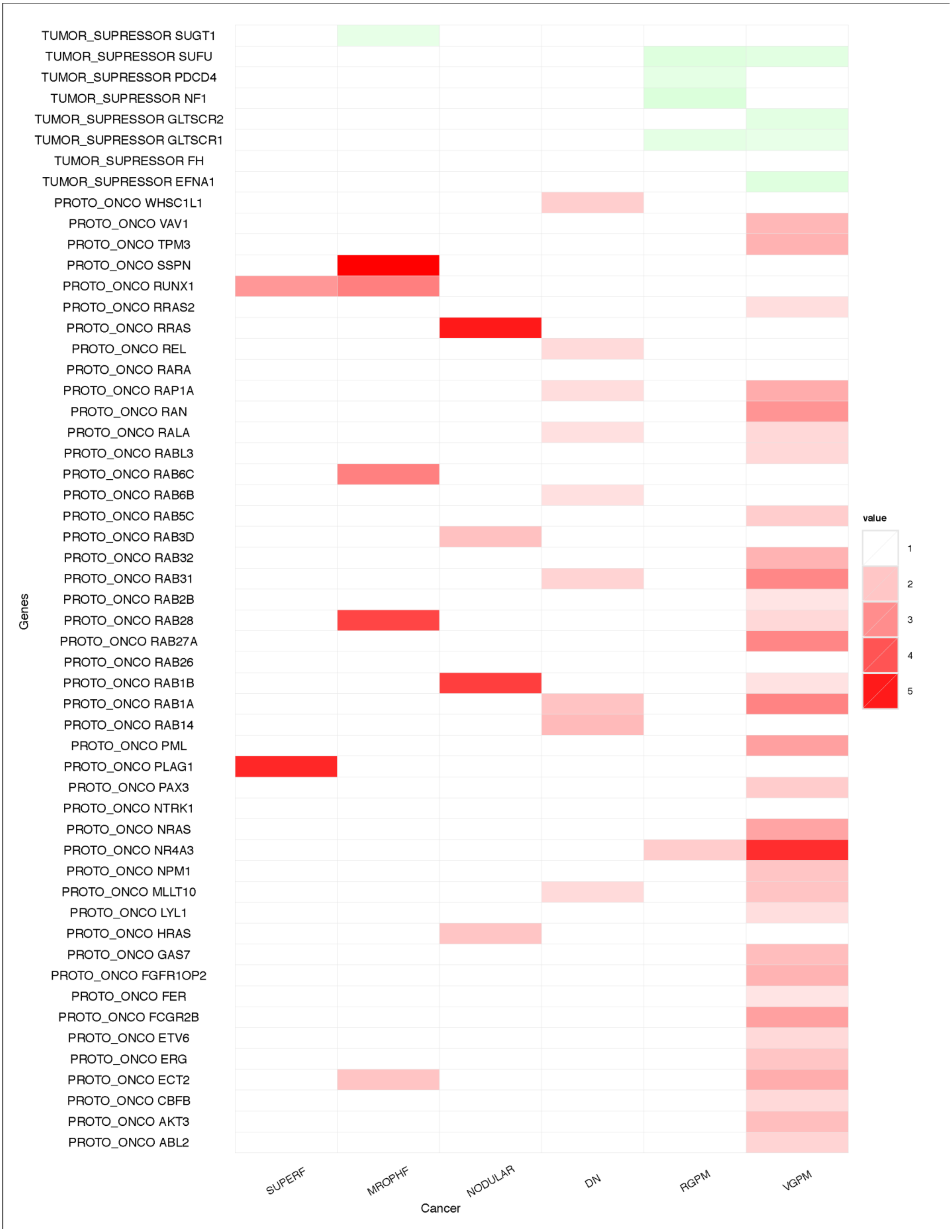

Supplement: Figure S1 — Expression level changes of proto-oncogene and tumor-suppressor genes for two skin cancer types. (PDF) [file pone.0030750.s001.pdf]
