# Supplementary material for: A Comparative Study of Gene-Expression Data of Basal Cell Carcinoma and Melanoma Reveals New Insights about the Two Cancers
Source: PLoS One. 2012 Jan 25;7(1):e30750. doi: 10.1371/journal.pone.0030750 (PMC3266277; doi:10.1371/journal.pone.0030750)
Supplement: Figure S5 — Comparison of the gene expression fold change. (PDF) [file pone.0030750.s005.pdf]

Figure S5: Comparison of the gene expression fold change (A) between training and testing datasets of lung cancer, and (B-I) between different types of cancer. The distributions of the fold-changes (FC) of individual genes across all genes between cancer and the corresponding control tissues for the seven types of cancers were checked and found to be similar. (J) Comparison of the gene expression fold changes between paired and unpaired breast cancer. (PC: Pearson Correlation, P-value <0.005))

A

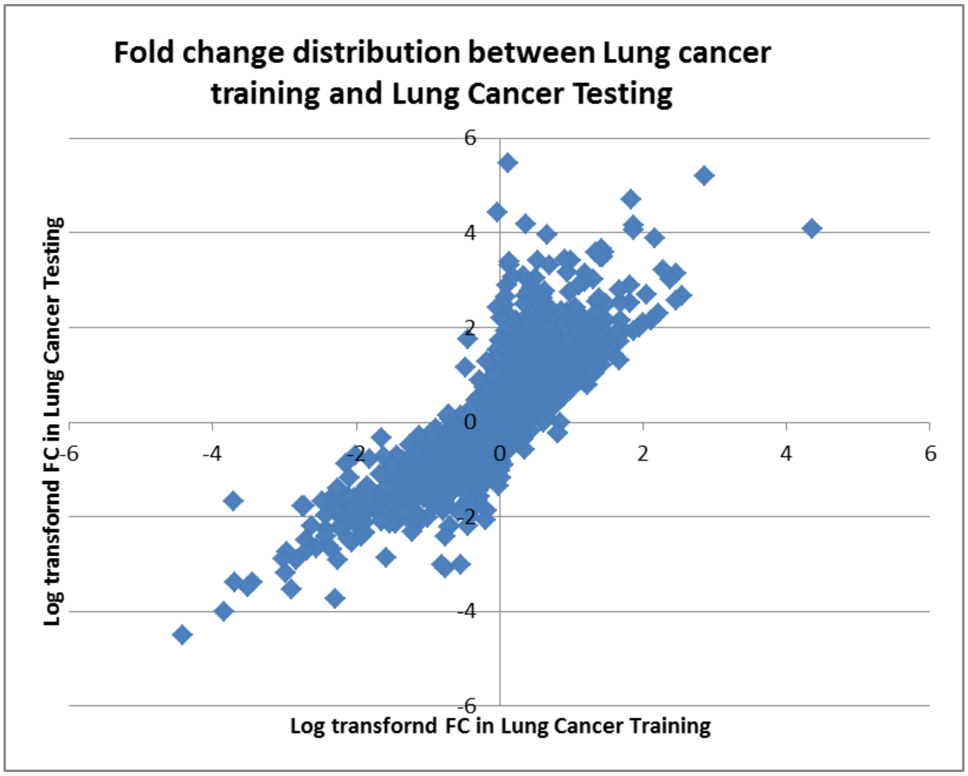

PC=0.8208

B

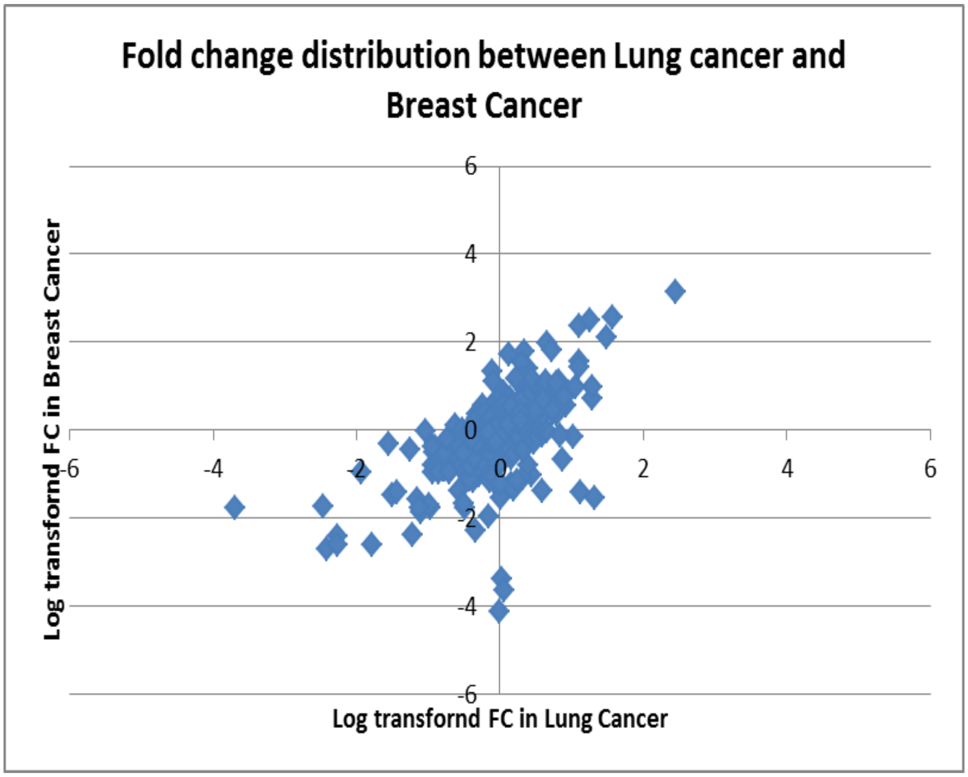

PC=0.6493

C

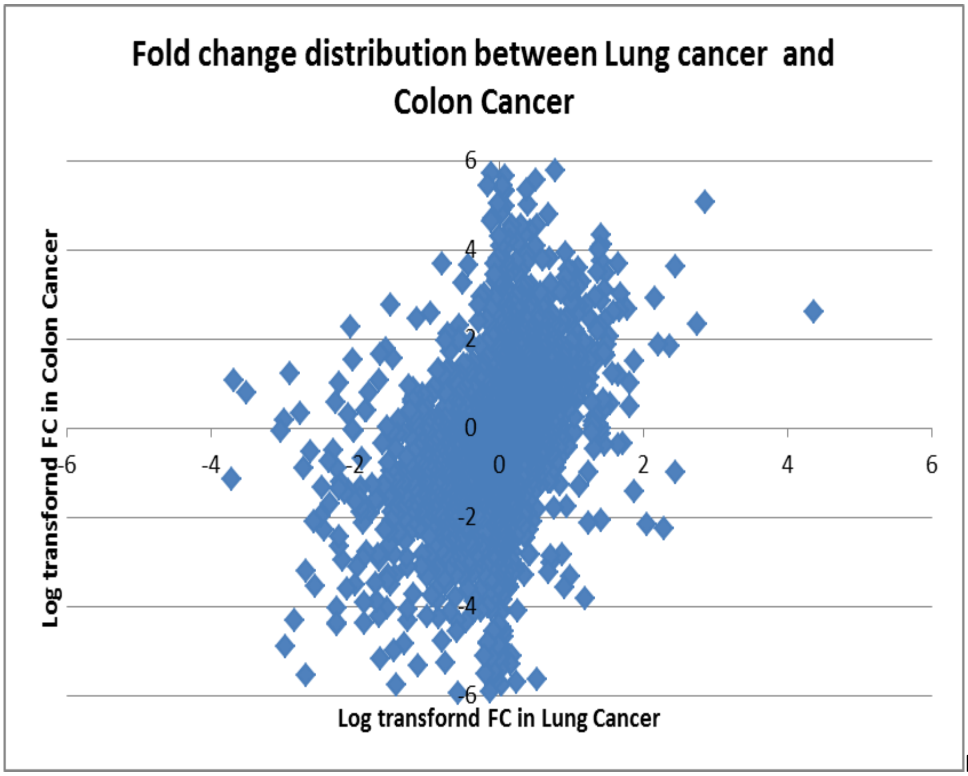

PC=0.4218

D

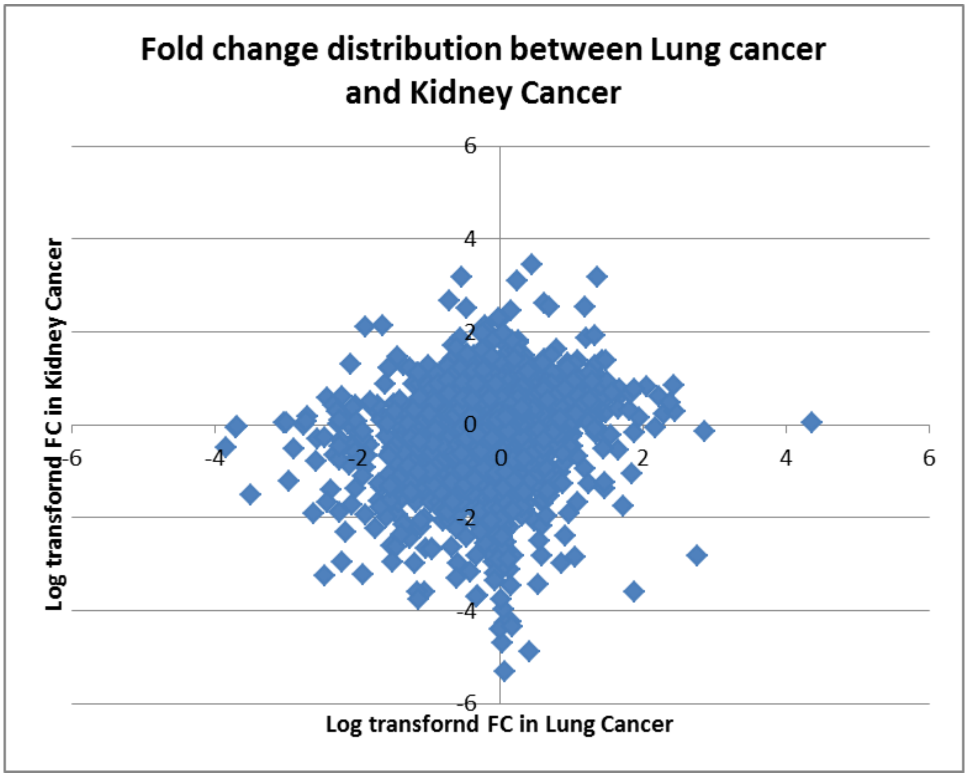

PC=0.2252

E

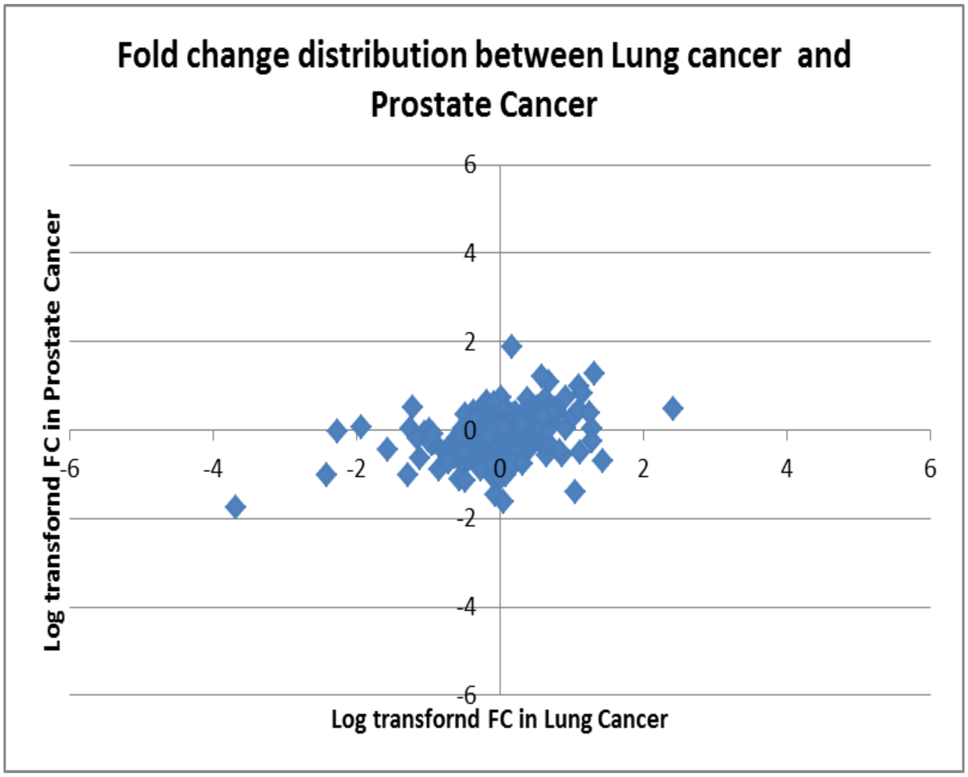

PC=0.3754

F

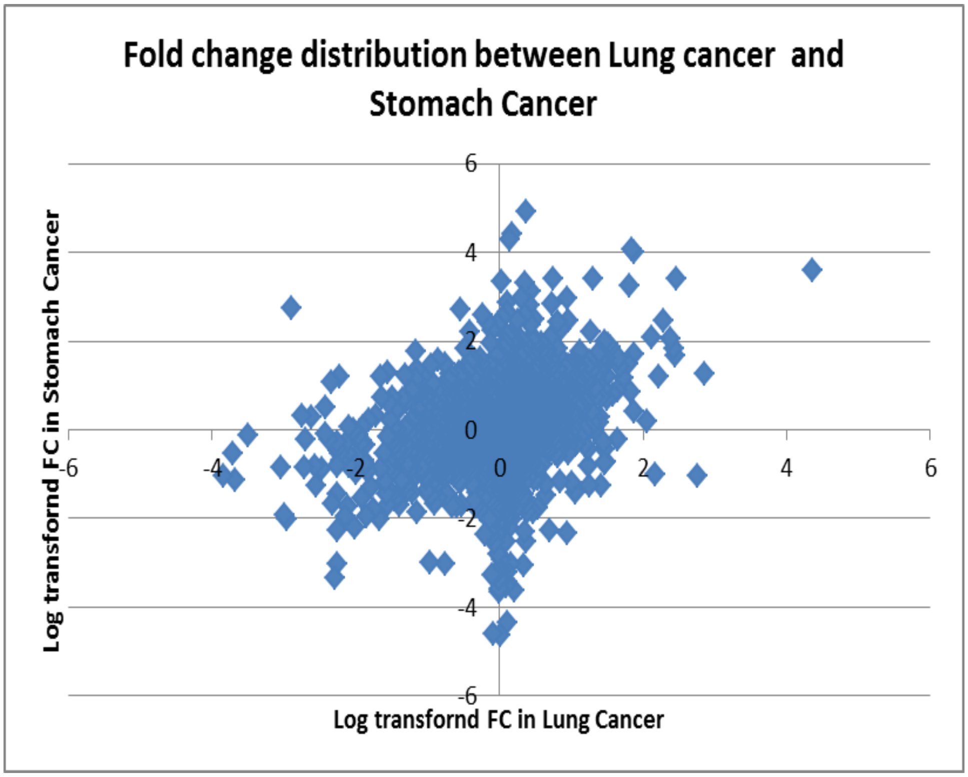

PC=0.3549

G

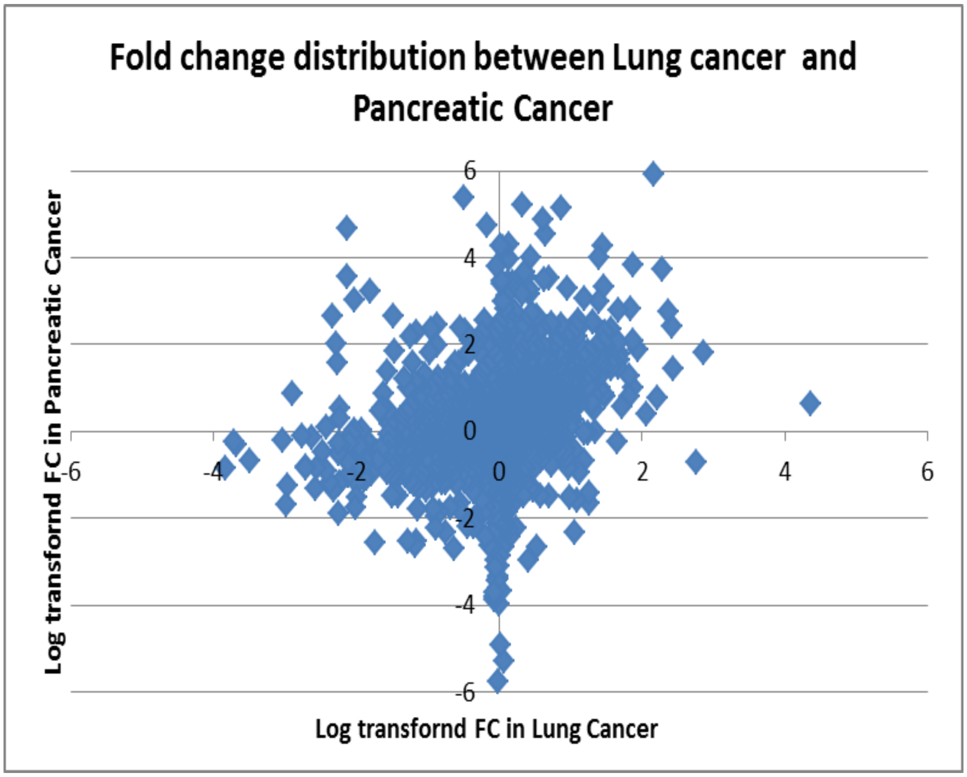

PC=0.3424

H

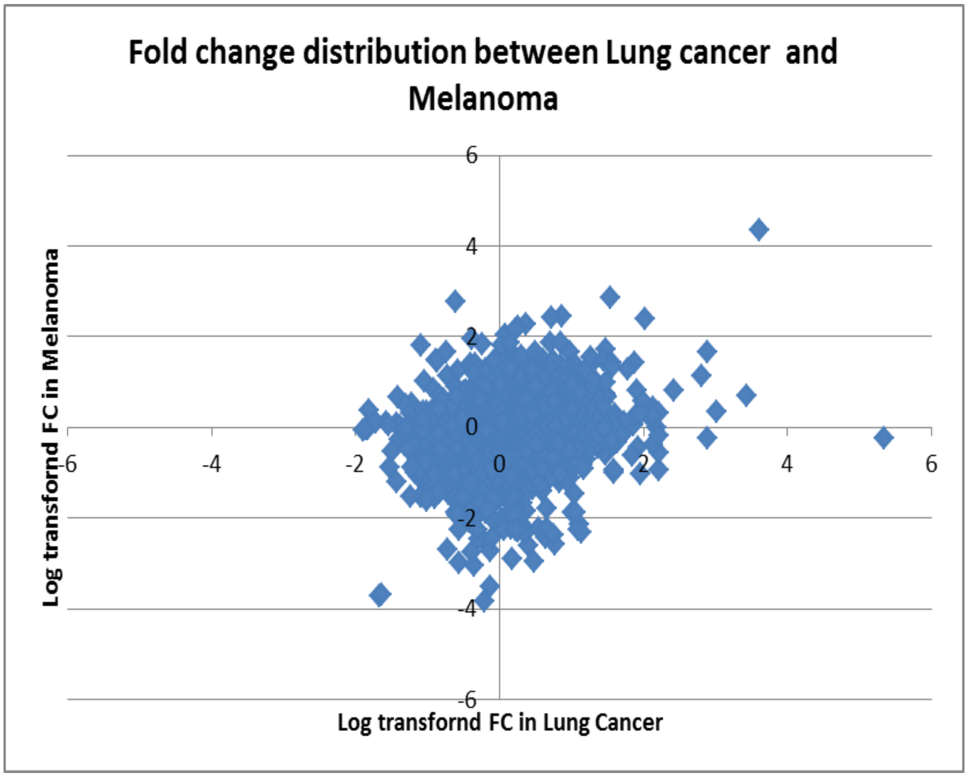

PC=0.2379

I

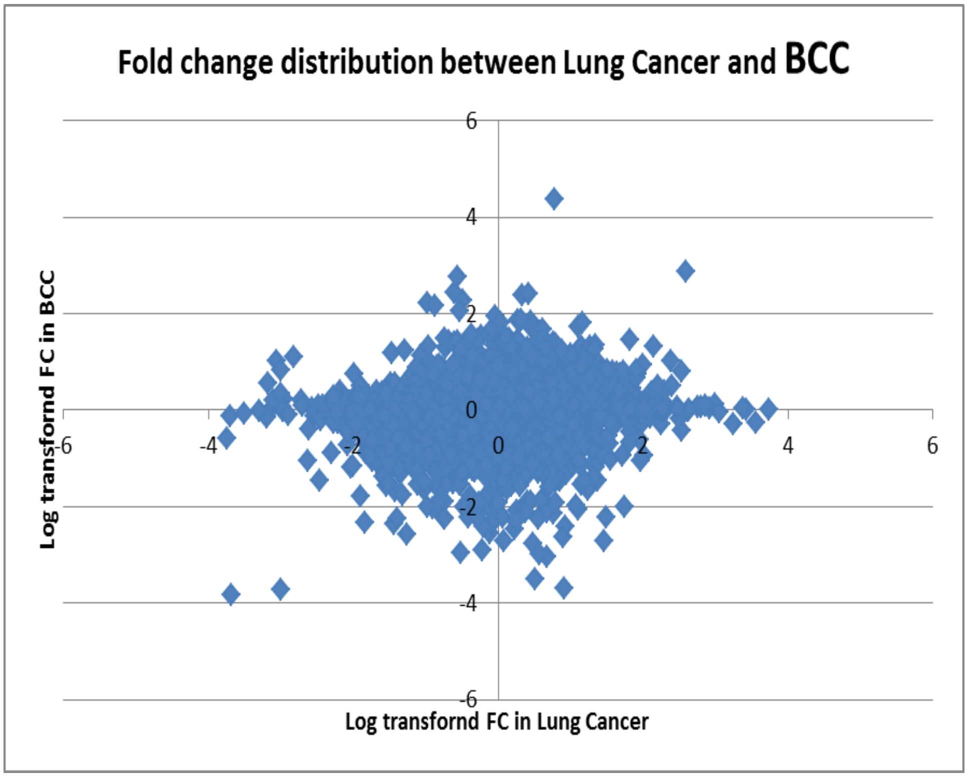

PC=0.1236

J

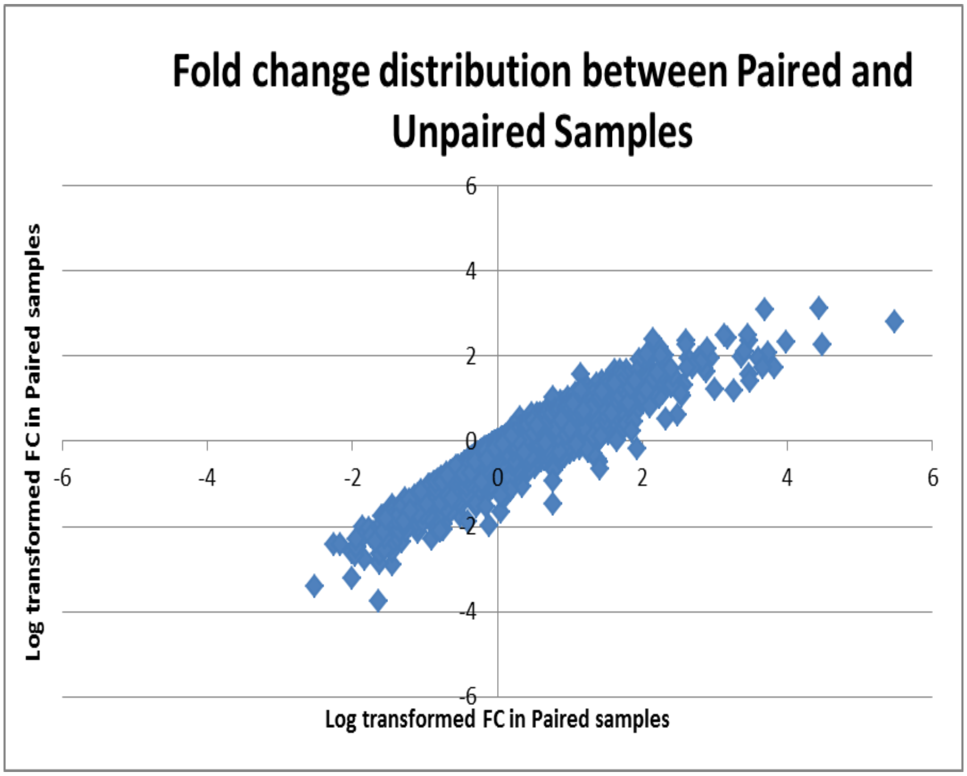

PC=0.8388
