# Supplementary material for: A Comparative Study of Gene-Expression Data of Basal Cell Carcinoma and Melanoma Reveals New Insights about the Two Cancers
Source: PLoS One. 2012 Jan 25;7(1):e30750. doi: 10.1371/journal.pone.0030750 (PMC3266277; doi:10.1371/journal.pone.0030750)
Supplement: Table S1 — Differentially expressed genes in skin cancer. (PDF) [file pone.0030750.s006.pdf]

Table S1. Differentially expressed genes in skin cancer. (Each cell in the represents the ratio of tumor mean /control mean in the corresponding cancer type and gene.)

	BCC			MELANOMA		
genes	SUPERF	MROPHF	NODULAR	DN	RGPM	VGPM
A4GNT	0.18	0.36				
AACS		0.23				
AASDHPPT	3.7					1.5
AATK		0.34				
ABCA7				1.69		
ABCB10						1.71
ABCC4						2.71
ABCC5	0.32		0.27			
ABCD3			0.36			
ABCG5						1.59
ABHD4					0.65	
ABHD6		0.14	0.12			0.55
ABI2						1.6
ABL2						1.74
ABLIM1						0.49
ABLIM2						0.58
ABTB1						1.57
ACACB					1.57	
ACAD9						0.48
ACADL						0.42
ACADSB			0.34			0.5
ACADVL			5.96			
ACBD6	0.6					
ACE						1.55
ACLY						0.59
ACOX2		0.22	0.22			
ACP2						1.91
ACP5						1.93
ACR					0.43	
ACSL3						4.46
ACSL4						2.41
ACSL5		0.23	0.29			
ACTG1						0.6
ACTR1A	2.84					
ACTR1B		0.33				
ACTR3						2.7

ACYP2						1.74
ADAM12		4.13				
ADAM22						0.6
ADAM28		2.58				1.75
ADAM30						0.51
ADAM33					0.49	
ADAM9		1.87				
ADAMDEC1						3
ADAMTS5			0.17			
ADAMTS6		1.64				1.62
ADCY4			3.93			
ADD1					0.66	
ADH6						0.58
ADIPOR1					0.51	
ADM2			4.77			
ADNP			4.03			
ADORA2A					0.64	
ADORA3						2.6
ADRB2						0.52
ADRB3					0.66	
ADSS						2.45
AEBP1			5.67			
AFG3L1		0.52				2.25
AGA						1.94
AGPS						3.69
AGTRL1						1.5
AHI1					0.52	1.72
AHSA1						2.06
AHSG			4.16			
AIM2						3.55
AK3L1						0.42
AK5						1.63
AKAP10						2.19
AKR1C1		0.32	0.27			
AKR1C4			0.29			
AKR1D1						0.31
AKR7A2		0.44				
AKT3						2.13
ALDH2						0.43
ALDOB					0.59	0.66
ALDOC			0.49			

ALG1						1.57
ALG3					0.64	
ALG6						2.09
ALOX12		0.18				
ALOX15B		0.09				
ALPPL2					0.45	
ALS2CR2						2.39
ALS2CR4						0.54
AMOT					0.46	
AMOTL1						1.95
AMOTL2	0.47	0.52	0.35			
ANAPC13				1.54		1.9
ANAPC2						2.02
ANG						0.54
ANK2					0.28	
ANK3			0.17			0.43
ANKMY1					1.53	1.73
ANKRD10						1.82
ANKRD12		2.41				0.53
ANKRD28						1.71
ANKRD5						1.63
ANKRD6						1.63
ANLN			3.93			
ANTXR1		5.34				
ANXA1		2.78				
ANXA3				1.52		
ANXA5						1.79
ANXA7			0.45			
ANXA9		0.33				
AP1B1						1.52
AP1S1		0.59				
AP2B1						1.88
AP3D1						1.71
AP3M2						1.54
APBA3						0.59
APC		2.13				
APC2						1.82
APCS						0.58
APEH			3.82			
APOC4			8.25			
APOD		0.14	0.11			

APOL2						1.61
APOL3			3.6			
APRIN						0.57
APXL					0.49	
AQP1						0.61
AQP2					0.6	
AQP5					0.45	0.53
AQP9			0.12			
AR						0.29
ARF1						0.59
ARHGAP18			0.32			
ARHGAP22						1.5
ARHGAP26		0.32				
ARHGAP5						1.66
ARHGAP8			11.05			
ARHGDIG			1.78			
ARHGEF15					0.45	0.63
ARHGEF4						0.51
ARHGEF5						0.48
ARHGEF9						1.65
ARID1A						0.66
ARIH2			1.97			
ARL1					0.6	2.64
ARL4A						0.52
ARL6						1.72
ARL6IP4			6.93			
ARMC2		2.34				
ARMC5			4.93			
ARMC6					0.41	
ARMCX3						3.14
ARPC2			4.6			
ARRDC2	3.6		4.86			
ARSB						1.91
ARTN						1.5
ARVCF						0.49
ASAH1						3.99
ASCC1						1.71
ASCL2			7.39			0.49
ASL						1.81
ASMTL						1.5
ASRGL1					1.74	2.18

ASS		0.63				
ASTN						0.64
ASTN2					0.66	
ATF5						1.8
ATM						2.47
ATOX1						1.99
ATP1A2			0.2			0.38
ATP1B3			0.39			0.6
ATP2A2	0.35	0.54				
ATP2B1	7.72		4.97			
ATP4B				0.59		
ATP5C1						2.26
ATP5G3		0.44				
ATP5H		0.39				
ATP5L	0.33	0.31				
ATP6AP2						2.01
ATP6V0E						2.17
ATP6V1C1						2.41
ATP6V1D						1.69
ATP6V1G2						0.65
ATP6V1H						2.68
ATP8A1						1.63
ATRX						1.93
AVPI1		0.38				
AVPR2						1.59
B3GNT1					0.5	
B4GALT3		0.45	0.42			
B4GALT5				1.85		
BAALC						2.62
BAG1		0.45				
BAMBI						2.11
BAP1		1.76	3.62			
BARX2		0.39	0.41			
BASP1		3.48			0.53	
BAT5						3.41
BBOX1						0.48
BCAN						1.64
BCAP31			8.36			
BCAT1						2.19
BCKDHA		0.3				
BCL11A						0.42

BCL2					0.56	
BCL2A1						4.53
BCL6	0.4		0.31			0.48
BCOR				2.02		
BCR			0.37			
BCS1L	0.5	0.55				
BDKRB1						0.45
BECN1				1.65		2.09
BET1						2.29
BET1L		0.52	0.49			
BEX1		3.16	4.63			0.61
BEX2						0.6
BEXL1						1.61
BFAR						1.95
BGLAP			5.49			
BHLHB3						2.86
BICD1						2.53
BICD2					0.44	
BIN2						2.48
BIRC2						2.76
BLMH						0.58
BLOC1S1			3.55			
BLVRB		0.48				
BMP1						1.5
BMP2K						2.13
BMP7		0.53				
BNC2	25.85	27.07	6.76			
BOLL						0.66
BRD1						0.55
BRD4			5.92			
BRD9						1.6
BRDT						0.64
BRIP1						2.14
BRMS1L						1.66
BRUNOL4						1.5
BTBD14B					0.54	1.5
BTC						0.37
BTG1			0.36			
BTG2					0.66	
BTG4				0.52		
BTN2A2						1.62

BTN3A1						1.73
BTN3A2						1.5
BTNL3		4.6				
BTRC		0.41				
BUB1B						1.55
BZRAP1						0.63
C10orf11						1.51
C10orf22		2.37				
C10orf27					0.5	
C10orf57						0.42
C10orf65						2.02
C11orf1					0.49	
C11orf11			0.55			
C11orf2			4.21			
C11orf9					0.39	0.48
C12orf5						2.89
C14orf10						1.87
C14orf109						1.83
C14orf112	0.57	0.52				
C14orf119						1.75
C14orf122			2.08			
C14orf124		0.39	0.38			
C14orf132					0.42	0.43
C14orf133					0.64	
C14orf138						1.82
C14orf142						2.85
C14orf149						1.62
C14orf151						1.73
C14orf155						0.39
C14orf156		0.31				
C14orf173			2.72			
C14orf21			2.77			
C14orf37						1.53
C15orf20						2.12
C15orf24						1.61
C16orf33			1.75			
C17orf37		0.59				
C18orf10					0.55	
C19orf26			4.41			
C19orf33			5.41			
C1orf19						2.18

C1orf25						1.7
C1orf33	0.41					
C1orf35		0.64				
C1orf38						2.66
C1QA						6.43
C1QTNF2						0.6
C1QTNF6		2.54				1.94
C2					0.24	
C20orf100					0.42	
C20orf103	3	22.83				
C20orf108						1.69
C20orf11						0.62
C20orf116		0.54	0.47			
C20orf24				1.51		1.72
C20orf30						2.41
C20orf4		0.56				
C20orf42						0.46
C20orf43			1.88			
C20orf6		4.3				
C20orf77					1.52	2.53
C21orf25			0.28			
C21orf45					0.63	
C21orf55						1.54
C21orf56			2.64			
C21orf63		0.35	0.42			
C21orf91						1.67
C2orf16					1.53	1.91
C2orf27						3.98
C2orf30						3.29
C2orf7		0.35	0.37			
C3AR1						2.88
C3orf1						1.63
C4orf15		2.82	3.05			
C4orf8		0.54				
C5orf13			3.04			
C5orf5						1.5
C6orf105			0.16			0.5
C6orf117						0.51
C6orf129						2.08
C6orf130					0.63	
C6orf149					0.47	

C6orf15		6.09				
C6orf203				1.96		2.13
C6orf208						1.5
C6orf27					0.38	
C6orf48		1.8				
C6orf51						1.82
C6orf62			4.44			2.01
C6orf85					0.3	
C7orf20		0.33	0.36			2.03
C7orf25						2.11
C7orf26						1.59
C7orf31			0.4			
C7orf36						1.84
C9orf125					0.55	0.52
C9orf156						1.62
C9orf25					0.48	
C9orf58		0.27	0.28			
C9orf84						0.53
C9orf88					0.44	
C9orf9					0.66	0.58
CA12						0.52
CA2				2.09		
CABLES2			2.64			
CACNA1A						1.5
CACNB3					0.38	
CACNB4			0.32			
CALCB	3.6					
CALD1		3.91	2.49			
CALU		3.33				
CAMK2G			1.83			0.61
CAMLG						0.64
CAMTA2			0.42			
CAPN10		0.59				
CAPN6					0.57	
CAPZA1						0.64
CARD12						1.8
CARD15			0.15			
CARD9						1.78
CASC3						0.53
CASP2				1.5		
CASQ2		0.28	0.26			

CATSPER1						2.82
CATSPER2					0.66	
CBFA2T2					0.58	
CBFB						1.67
CBLB						1.74
CBX3						2.56
CBX5						2.27
CCDC3						0.32
CCDC5						2.14
CCL13						2.48
CCL18						5.75
CCL2						2.34
CCL20				3.17		
CCL24					0.49	
CCL27						0.25
CCL8						2.14
CCNA2						2.03
CCND2	1.89					
CCNF	2.91					
CCNH						1.59
CCPG1						4.38
CCR1						2.07
CCR2				2.09		2.7
CCR5						2.02
CCR6						0.39
CCRL2						3.06
CCT4		2.03				
CCT6A						2.29
CD160		0.24				
CD164						1.71
CD1A		0.12	0.22			0.42
CD1C				1.88		
CD1D						1.84
CD1E				1.84		
CD207			0.15			
CD209						1.6
CD28						2.81
CD34						0.43
CD36						4.08
CD3E			3.95			
CD4					0.44	

CD47						2.83
CD48						3.09
CD53						5.6
CD58						3.27
CD59			0.47			
CD63						0.64
CD68	2.16	3.18	1.79			2.99
CD80						1.56
CD83						2.86
CD8A						2.44
CD9		0.24		1.72		
CD96						1.88
CDC16						2.45
CDC23						2.21
CDC25A					1.54	1.52
CDC2L5						1.54
CDC40				1.81		1.55
CDC42BPA						2.16
CDC42EP1					0.57	
CDC42EP4					0.48	
CDC42SE2						1.99
CDC45L			1.74			
CDC6						2.21
CDC7	2.18	3.43				
CDCA1		4.01				3.97
CDCA3						1.5
CDH22					0.4	0.46
CDIPT		0.29				
CDK2AP1						1.82
CDK5R2					0.35	
CDK5RAP1						1.6
CDK7						1.5
CDK9					1.5	
CDKN1A		0.29				
CDO1					0.42	
CDON					1.68	
CEACAM6					0.46	
CEBPB						0.59
CECR2		0.31				
CENTA1						1.52
CENTD2						1.55

CER1		0.32				
CFLAR						2.09
CGRRF1						2.13
CHCHD2						0.62
CHCHD3						2.58
CHD1L						2.21
CHD4			0.38			
CHD9		2.45			0.27	
CHEK1						2.03
CHGA	25.94	18.7	29.97			
CHRD	2.15					
CHRNA3			0.37			
CHRND						1.68
CHST11						4.26
CHST2	2.7	2.79				1.75
CHST6		0.34			1.58	3.32
CIAS1						1.94
CITED1						1.65
CITED2		0.47				
CKLF						1.99
CKS2						1.67
CLCN3						1.79
CLCN5						1.84
CLDN18		3.24				
CLDN5						0.54
CLDN7		2.27	3.38			
CLIC4	2.68	3.75	2.39			2.33
CLK3			5.49			
CLPTM1	0.53	0.41	0.39			
CLTCL1			0.23			
CLU			5.08			
CMAS						2.15
CNN3						3.64
CNOT3			4.62			
CNOT7				1.53		
CNP			3.06			
CNTFR						0.61
COBL		0.26	0.2			
COCH		0.1	0.08			
COG8						1.77
COL17A1						0.37

COL18A1		2.34				1.52
COL21A1						0.53
COL3A1		10.18				
COL4A1		7.77	4.2			1.8
COL4A5						1.5
COL4A6						0.5
COL5A2						2.59
COL5A3		2.33				
COL6A1		6.24				
COL6A3	4.97				0.57	
COL9A2			2.57			
COLEC12			0.3			
COMMD1			0.42			
COPA						1.56
COPS8						2.22
COQ3						1.97
COQ6						1.57
CORO2B					0.51	
CORT			6.09			
COTL1						2.33
COX17		0.55				
COX4I2		3.6	5.81			
COX6A1		0.29				
COX7C						0.64
CPA3			0.23			
CPA4					0.5	
CPAMD8		0.53				
CPB1			0.44			
CPD						1.63
CPEB3			0.27			0.39
CPLX1			3.28			
CPM			0.34			
CPN1						1.61
CPOX		0.49	0.37			
CPSF6						2.01
CPVL				1.55		3.74
CRAT		0.1	0.12			
CRBN						2.18
CREB1						3.51
CREBL2						1.57
CREM						2.43

CRIM1						0.47
CRY2						0.51
CRYAA			2.85			
CRYL1			4.98			
CS		0.4	0.33			
CSAD	0.43					
CSDA			3.85			
CSNK2A2		0.51				
CSPG2		4.35	14.33			
CSRP3		0.5				
CSTA						0.57
CSTB		0.37				1.96
CSTF2						1.61
CTAGE5			0.48			0.55
CTBP2	1.78					
CTDSPL					0.58	0.62
CTHRC1						7.12
CTNNBIP1						0.3
CTNNBL1						1.74
CTNND2		0.24				0.59
CTSB						3.4
CTSL						3.21
CTSW						1.93
CTTN		5.57	4.91			
CUEDC2	0.26	0.34				
CUL4B	2.28					2.68
CUTL1					0.51	
CXCL10						7.44
CXCL11						4.12
CXCL14		9.34				0.45
CXCL2						3.62
CXCL3					0.31	2.63
CXCL9	2.83	7.84				
CXCR4						2.46
CXCR6						4.06
CXorf12						0.57
CXorf9						2.37
CXXC5				0.66		0.62
CYBB						2.15
CYCS						2.73
CYP11A1					0.65	

CYP11B1					0.36	
CYP11B2						1.62
CYP20A1						2.31
CYP26B1			0.22			
CYP2E1						0.5
CYP2J2						0.62
CYP3A4						0.37
CYP3A5					0.58	0.33
CYP3A7						0.44
CYP4B1	0.21		0.14		0.48	0.23
CYP4F2		0.25	0.24			
CYP4F8						0.52
CYP51A1		0.43	0.34		0.54	
CYP7B1	3.75					
CYR61						2.01
DAB2						2.44
DACT1		7.04				
DAPK2		0.5				
DAPP1				1.5		
DBI						0.6
DCAMKL1		1.86				
DCBLD2						3
DCDC2						3.67
DCHS1	3.01					
DCI			3.03			
DCLRE1B						1.65
DCLRE1C						1.63
DCTN4						2.75
DCTN6						2.17
DCXR		0.56				
DDAH1	0.35	0.35	0.35			
DDEF1						2.74
DDIT4			4.03			
DDIT4L			0.15			
DDOST			2.63			
DDR1						0.36
DDT		0.25				
DDX17				1.72		
DDX18					0.6	
DDX24				1.98		
DDX50					0.55	0.63

DDX52				1.63		
DDX55						1.53
DDX56			5.45			
DEAF1						2.06
DEF6		6.03				
DEFA6						1.5
DEFB1	0.37					
DFFA						2.05
DFFB						1.68
DFNA5						5.94
DGCR2						0.42
DHCR24		0.31	0.28		0.49	0.47
DHCR7		0.35				
DHDH						1.66
DHPS		0.4				
DHRS7		5.31				2.09
DHRS8						3.38
DHX33				1.62		2.16
DHX57						1.62
DLG3						0.47
DLG5						0.44
DLG7						2.53
DLGAP1						3.12
DLGAP4			4.77		0.36	
DLX2						2
DLX3					0.49	
DMAP1			2.42			
DMD	4.4					0.48
DMTF1		2.23				
DNA2L						1.86
DNAJA2						2.15
DNAJA3			0.43			
DNAJA4		0.32				
DNAJB11						2.21
DNAJB4	3.04					
DNAJC10						1.69
DNAJC12						1.5
DNAJC3				1.97		1.7
DNAJC5B		1.65				
DNAJC8			3.02			
DNM1					0.66	

DNM1L						2.57
DNMT2						1.5
DNMT3B						0.62
DOCK11						1.85
DOCK5						1.5
DOCK8		2.34				
DOK3						1.5
DOK4						0.4
DONSON			0.45			2.27
DPEP2						2.05
DPF2			4.1		0.66	
DPP3		0.53				
DPYSL2	3.11					
DSC3						0.42
DSP						0.5
DTX2		0.47	0.48			
DUOX2						0.66
DUSP1		0.31				
DUSP10						2.26
DUSP11				1.69		
DUSP14	3.47					
DUSP2	0.31	0.18	0.3			
DUSP4					0.58	
DUSP9			1.93			
DVL1						1.5
DYRK3						2.02
DYSF			0.13			
E2F5	4.15					
EBI2						6.06
ECHDC1						2.66
ECT2		2.03				2.43
EDAR		9.08				
EDF1			1.85			
EEF1A1						0.59
EEF1E1						2.26
EEF2K						0.66
EFHC2		4.33				0.55
EFHD1					0.47	
EFNA1						0.44
EFNB3					0.56	0.64
EGFL7						1.85

EGLN1			3.67			
EGR1	0.15	0.13	0.17			
EHD3						1.93
EIF2S1						2.66
EIF2S2						1.78
EIF3S7						0.6
EIF4A1						2.53
EIF4B					0.55	
EIF4ENIF1						0.6
EIF4G1		1.52	8.56			
EIF5						1.79
EIF5A					1.6	
ELAC2			0.63			
ELF2	0.45					
ELF3						2.13
ELF5		0.22			0.54	
ELK1			0.4			
ELL2						0.57
ELL3						0.54
ELMOD1						0.37
ELOVL2	0.46					
ELOVL5				2.44		2.98
ELOVL6					0.36	
EMD			4.64			
EMILIN2						2.75
EMILIN3					0.52	
EML4			16.23			
EMP2		0.43	0.62			0.53
EMR2						1.6
EMR3						2.09
EMX2						0.4
ENAH		3.89				
ENDOGL1						2.27
ENPP1			0.28			
ENPP6	0.23					
ENTPD2					0.66	
ENTPD8					1.66	
EP300				1.8		
EPB41L1						0.61
EPB41L4B						0.39
EPHA5				2.33		

EPHX2						0.53
EPO					1.67	
ERBB2						0.65
ERCC2					0.46	0.48
ERF		4.25	10.05			
ERG						2.01
ERO1L		0.38				2.41
ERVWE1		6.82				
ESR1						0.66
ETFA				1.54		2.36
ETNK1						2.85
ETS2						0.42
ETV6						1.66
EVI2B	0.37					
EVPL					0.63	
EVX1					0.53	
EXO1						2.29
EXOSC1			0.57			
EXOSC7						0.63
EZH1			0.3			
F10			0.2			
F11R						0.62
F2						1.51
F2R		5.92				
F2RL1			0.34			
FA2H		0.07	0.17			
FAAH		0.44				
FABP4		0.08	0.05			
FABP5						3.63
FADS2		0.12	0.19			
FALZ					0.66	
FAM13C1						0.43
FAM20A		8.99	6.19			1.68
FAM29A	2.64			1.79		2.07
FAM32A	0.4					
FAM40A			0.37			
FAM49A						2.23
FANCC						0.54
FANCE						0.66
FAP						2.35
FARP1						1.65

FARSLA		0.55				
FBL		2.45	7.48	0.62		0.57
FBLN1				1.65		
FBXL16						0.35
FBXL19					0.4	
FBXO11						2.41
FBXO17					0.51	
FBXO32		4.67		1.74		2.42
FBXO36						2.02
FBXO7					0.39	
FBXO8			0.43			
FBXW10		0.18	0.18			
FBXW7						0.45
FBXW9		0.51				
FCER1G						2.07
FCER2						1.73
FCGBP						0.39
FCGR2A						4.17
FCGR2B			0.18			2.67
FCGR3A						4.86
FCN1						3.11
FDFT1			0.36			
FEM1B			0.35			
FEM1C						1.75
FEN1	2.21		5.2			1.78
FER						1.5
FER1L3						0.47
FEZ2		2.21				1.71
FGD1					0.58	
FGF1						2.77
FGF16		2.13	4.03			
FGF20			0.22			
FGF22		0.44				
FGF3						0.59
FGFR1			0.43			
FGFR1OP2						2.32
FGFR3						0.27
FGG			3.07			
FGL2			0.24			
FH						3.11
FHOD1						1.69

FIGN						1.84
FIGNL1						1.74
FKBP11			3			2.55
FKBP3						1.73
FKBP8			17.65			
FKBP9L						2.27
FLG		7.28				0.61
FLNA			8.28			
FLNC					1.53	
FLOT2					1.98	
FMNL1					2.28	2.31
FMO1						0.51
FMO3						1.5
FOSL1						1.81
FOXD2					0.45	
FOXK2	3.33					
FOXO1A						0.56
FOXQ1						0.25
FRAS1		3.5				
FRS3						1.5
FSTL5						3.23
FTH1					0.63	1.98
FUCA1						3.41
FUCA2						2.59
FUSIP1				1.71		2.34
FVT1			0.39			
FXYD5			9.13			
FYB						2.52
FZD2		2.93				
FZD5	7.03					
FZD7			3.1			
FZR1						1.89
GAB1						1.59
GABARAPL1		0.19				
GABPA						1.83
GABPB2	0.33		0.28			1.64
GABRA2					0.47	0.62
GABRE						0.36
GALK1						1.73
GALNTL1		0.19				
GALNTL5						0.66

GALR3			2.39			
GAP43						0.63
GAS2	8.71		9.36			
GAS6			32.02			
GAS7						2.16
GATA3						0.37
GATM					0.37	0.24
GBA			3.16			
GBA2			0.48			
GBF1			0.66			
GBP5						3.42
GCNT3		0.12				
GDA						2.05
GDF15						7.82
GDF5						1.84
GDPD2						0.27
GEM		7.32				2.57
GFPT1						2.01
GFPT2			0.39			
GFRA2					0.66	
GFRA3						0.6
GFRA4						1.5
GGH						2.99
GHITM			0.41			
GHR	0.25	0.14	0.2			
GHRH			4.4			
GJA1		2.07				
GJB4		0.18				
GJB6						2.9
GK						2.43
GLA						1.82
GLE1L	1.62		11.75			
GLRA3	0.51					
GLS						2.01
GLTSCR1					0.54	0.6
GLTSCR2						0.51
GLYAT					0.55	
GNA12			9.27			
GNA13	2.33	2.78				1.77
GNAI2	3.41	2.75	3.98			
GNB5						1.5

GNG13					0.48	
GNG7					0.46	
GNG8			14.5			
GNLY						3.39
GNPNAT1			0.47			
GNS	2.2					
GOLGA1						0.62
GOLGA2					0.62	
GOSR1						1.6
GOT2			0.57			
GP9						2.47
GPD1					0.66	
GPD1L					0.66	
GPM6A		0.24				
GPR143						2.3
GPR18						2.16
GPR19						1.89
GPR30						0.66
GPR34						2.74
GPR37					0.23	
GPR37L1		0.36				
GPR4						2.88
GPR61			0.6			
GPR65						3.06
GPR75						0.51
GPR84						1.8
GPRASP2					0.58	0.62
GPSM3			4.32			1.56
GRAP						1.54
GRB7		0.29				
GRID1						0.52
GRIK3						1.5
GRIN3B						1.67
GRINL1A					0.59	
GRK7						0.39
GRM1			40.94			
GSTM5			0.06		0.61	
GSTO1						2.44
GSTT2						0.32
GTF2A2						1.9
GTF2IRD1				0.64		0.48

GTF3C5		2.03	4.44			
GTPBP2			5.39			
GTPBP3					1.7	1.99
GTPBP4						1.5
GTPBP6						1.86
GULP1	0.33					
GYPC			0.29			
GZMA						2.1
GZMB						2.52
GZMK						3.18
H2AFZ			1.9			2.73
HABP4		0.42				1.6
HADH2		0.42	0.47			
HADHA	0.55					
HADHSC		0.38				
HAMP	0.21	0.33				
HAO2		0.04	0.08			
HARSL						1.63
HAS2						2.33
HAT1						2.84
HBQ1						0.6
HBXIP						2.02
HCN2					0.47	
HCN4					0.41	
HCRT					0.6	
HDAC5					0.51	0.59
HEBP2			0.39			
HECTD1		0.31				
HEPH			2.22			
HERC3						0.52
HES1					0.55	0.49
HES6						1.57
HESX1						1.76
HEXA						2.28
HEXB						2.5
HEY1				1.91		
HFE						1.8
HGD						0.58
HIBCH						2.33
HIST1H1C					1.73	2.24
HIST1H1E		5.6				

HIST1H1T			5.2			
HIST1H2BM						2.22
HK1			0.55			
HK2						1.71
HLA-C						0.6
HLA-DMA						3.35
HLA-DMB						1.92
HLA-DOA				1.5		2.48
HLA-DPA1				1.95		
HLA-DPB1				1.74		3.14
HLA-DQA2	6.04					
HLA-G					1.55	
HLF					0.54	0.29
HM13						3.11
HMG4L						1.69
HMGB1		8.01				0.64
HMGCR		0.45				0.56
HMGCS2		0.07				
HMGN4						1.81
HNRPA2B1						2.23
HNRPDL						2.18
HNRPH3		1.99				
HNRPL				1.5		
HNRPM						0.62
HOMER1						1.91
HOOK2			0.51			
HOXB2						1.72
HOXC10					0.55	
HOXC11		3.18				
HOXC4					1.65	
HPCAL1			2.61			1.74
HPGD				1.61		
HPN			3.48			
HPS3						2.82
HRAS			2.02			
HRG			6.26			
HRH1				2.22		
HRK						4.3
HS3ST2						4
HSD17B2		0.19				
HSD3B1			0.1			

HSPA14		0.37	0.34			
HSPA1L			8.7			
HSPA4						3.19
HSPA6						2.82
HSPB1			6.4			
HSPE1						0.59
HSPH1		2.65				1.7
HTATIP			2.88			
HTR2B		0.42				
HTR5A						1.64
HUS1						2.25
ICMT						2.06
ICT1		0.53				
ID4			0.38			
IDH1			0.28			
IDH3A						1.7
IDUA						1.89
IFI30						2.59
IFI44						3.79
IFIH1				1.79		2.53
IFRD1						2.57
IGF2R						2.46
IGFBP2			2.6			1.79
IGFBP5						1.6
IGFBP7		2.21				
IGSF4C						1.66
IGSF6				2.03		2.91
IGSF9						0.47
IHPK2			4.92			0.62
IKBKB			1.66			
IL12RB2						1.8
IL13						0.58
IL15RA					0.31	
IL16						1.76
IL17B						1.7
IL17C					1.57	1.7
IL17RC					1.76	
IL18BP						2.75
IL1F7						0.41
IL1RAP						2.04
IL20RA						0.4

IL21R						2.02
IL22RA1						0.39
IL27RA						1.82
IL2RA						2.3
IL6						1.89
IL6R				1.99		
IL7			0.2			
IMPA1						2
IMPDH2		0.43	0.41			
INDO						3.2
INE1						0.62
ING4		1.66				
ING5						0.55
INPP1				2.03		
INPP4B				1.69		2.65
INSRR						1.54
IPO7				1.5		
IPO9						1.9
IQCA			7.04			
IQCC						1.71
IRX5						0.45
ISLR						0.57
ITCH						1.76
ITGA2B						1.85
ITGA7		0.27	0.13			
ITGAL						2.07
ITGAX						2.92
ITGB1BP1						2.07
ITGB3						1.58
ITGB3BP						1.85
ITGB8						0.52
ITM2B						0.61
ITPKC		0.37				
ITPR3			2.22			
IVL		2.43	2.93			
JAK3	2.25				0.47	2.14
JPH2					0.5	
JUB						0.39
JUNB					0.45	
KAL1		2.96				
KATNAL1						1.53

KBTBD2						2.15
KCNAB3	0.38					
KCNE3						1.7
KCNH4						0.57
KCNIP1	3.3					
KCNJ16						0.66
KCNK12					0.32	
KCNK15					0.62	
KCNK6	0.43					
KCNQ4			4.09			
KCNV1	0.25	0.26				
KCTD12						1.66
KDELR2						2.29
KEAP1		0.42	0.39			
KIAA0103						1.71
KIAA0258			0.26			
KIAA0274						2.17
KIAA0284		0.46				
KIAA0913					0.37	
KIAA1212						1.91
KIAA1305					0.66	
KIAA1529						1.8
KIAA1704						2.21
KIAA1715					0.65	
KIAA1754						0.65
KIAA1900	7.53					
KIAA2010		2.6				
KIF12			3			
KIF1C					0.57	
KIF20A						2.07
KIF22			5.34			
KIF23						1.75
KIF3A		3.6		1.5		1.58
KIF5B						1.87
KIN						2.62
KIRREL						2.67
KLF5						0.28
KLF8			0.14			0.56
KLHL7						1.93
KLK1						0.42
KLK13						1.56

KLK3					0.63	0.55
KLK6						1.76
KLRB1						2.35
KLRC4						2.11
KLRD1						2.93
KLRF1						2.18
KNDC1					0.66	
KNTC1						1.88
KNTC2						3.07
KPNA2						1.95
KPNA4						1.65
KPNB1						2.23
KRT1						0.54
KRT10						0.55
KRT15						0.26
KRT17			7.37			
KRT23						0.48
KRT3					0.41	
KRTAP3-1		0.16				
KYNU				1.57		3.05
LACE1						1.89
LAD1						0.34
LAG3		2.39				1.77
LAIR1						2.64
LAP3						3.85
LAPTM4B	4.55					
LCP2						3.96
LDB1	0.29					
LDHA						2.21
LDHB		3.99				
LEF1						2.09
LEMD2					0.65	
LENG8					0.41	0.57
LFNG						1.5
LGALS1			6.48			
LGALS12		2.31				
LGALS13						2.8
LGALS4		5.52				
LHFPL2						2.73
LHX6				1.52		2.46
LIF						1.53

LILRA1			2.81			
LILRB3						2
LILRB5						1.97
LIMK2			5.06			
LIN7B						0.51
LIPA						2.24
LIPC						0.63
LLGL1						0.57
LMAN2			2.71			
LMNA			28.9			
LMO3					0.51	
LMX1B						1.61
LNPEP						1.71
LPAL2		5.74				
LPIN1		0.24	0.21			2.05
LPXN						2.93
LRCH1			0.53			
LRG1					0.66	
LRP2			0.18			
LRP4						0.28
LRP8						4.61
LRRC14						0.6
LRRFIP2						2.07
LRRN1						0.43
LRRN5					0.46	0.63
LRRN6A			2.43			
LRSAM1		0.51	0.36			
LSAMP						0.63
LSM1						1.86
LSM10						2.66
LSS						0.5
LTB		2.15				
LTBP2					1.51	2.09
LUM	5.82	11.79				2.92
LY6E			2.65			
LY86			6.84			
LY96			0.32			3.04
LYL1						1.58
LYZ		1.86				
LZIC			5.21			
LZTFL1		2.17				

MAD2L1						3.83
MAD2L1BP	0.66					1.67
MAGEA12				0.63		
MAGEA9	0.3	0.09				
MAGEC2						0.59
MAGED2	1.97		7.39			
MAGI1						0.44
MAMDC2		0.21	0.18			
MAML1						0.57
MAN1C1			0.33			
MAN2A1					0.66	
MANBA						1.5
MAP2K1IP1			0.53			2.16
MAP2K7						1.5
MAP3K3					0.59	
MAP4K4						2.97
MAP4K5				1.98		2.07
MAPK11						1.5
MAPK3		0.19				
MAPK8		0.29				
MAPRE1						1.59
MAPT					0.63	0.56
MARCKS						1.61
MARK2		2.04				
MARVELD3		0.18				
MASP1						1.79
MAT2B						1.74
MATN2			0.17			
MAX		0.18				
MAZ						1.65
MB		0.02				
MBD6			9.05			
MCCC2			0.25			1.89
MCF2L						0.5
MCFD2						1.74
MCM7			3.32			
MCM8						1.69
MCOLN3						2.6
MDGA1						0.39
MDH2						1.6
MDM4						1.77

ME2						2.14
MECP2						0.59
MED6						1.62
MED8			0.37			2.17
MEF2B					0.63	0.61
MEF2C						1.79
MELK						2.25
METTL1						1.52
METTL3		0.47	0.42			
MFAP3						1.54
MFI2						4.1
MGAT1		2.47				
MGAT4B			5.09			
MGST2		0.39	0.34			0.6
MIB1						1.74
MICA	0.28					
MID2		0.12				
MIF			4.46			
MINA						1.68
MKKS	0.48		0.41			
MKL2						0.61
MKNK1			0.4			
MKNK2			0.43			0.57
MKRN2			9.71			
MLF1	0.41	0.35	0.38			
MLF2					0.55	
MLH3						1.93
MLL					0.46	0.59
MLL5		0.61				
MLLT1						0.59
MLLT10				1.64		2.03
MLLT6					0.49	
MMP1		18.32				3.88
MMP10		5.94				
MMP11					1.64	
MMP13		4.93				
MMP19						1.5
MMP28					0.56	
MMP7			1.78			
MMRN2					0.55	0.54
MNDA				1.95		3.8

MOBP						2.19
MOCOS			0.34			2.17
MOCS2						2.72
MORF4L1						2.7
MOSPD1						2.09
MPHOSPH10						1.9
MPPE1						1.5
MPV17						1.69
MPZ		0.37				
MPZL1						2.07
MR1						1.79
MRC2			0.44			
MRGPRF					0.57	
MRP63			0.54			
MRPL13						1.84
MRPL16			0.37			
MRPL17						2.3
MRPL19						2.02
MRPL28						1.65
MRPL30						1.84
MRPL32						1.78
MRPL33		0.45				
MRPL35						2.08
MRPL39						1.82
MRPL42						2.01
MRPL44						1.97
MRPL45			5.73			

MRPL46	0.45	0.53				
MRPS10				1.96		1.78
MRPS14	0.38	0.27				1.68
MRPS18B		0.6				
MRPS22		0.51				
MRPS23						2.17
MRPS24	0.37	0.35	0.49			
MRPS30						2.08
MRPS33						1.97
MRPS34					0.45	
MRS2L						2.37
MS4A4A			0.23			3.76
MS4A7						1.71
MSH3						2.35
MSI2						1.68
MSLN						1.52
MSMB		0.12	0.12			
MSTP9	2.97					
MTA1					0.54	
MTA3	0.32					
MTCH2					0.4	1.6
MTM1			0.37			
MTMR1			3.51	1.57		
MTMR9						1.51

MTNR1B					1.96	
MTPN						3.91
MTRF1L						1.73
MTSS1		5.5				
MUC6					0.59	
MX2	0.34	0.35				2.08
MXD3						2.27
MYADM					0.49	
MYB			0.27			0.61
MYBPC1	0.12		0.05			
MYC					0.41	
MYEF2						2.26
MYH14					0.42	0.61
MYH2	0.15	0.07	0.1			
MYH9			4.22			
MYLK				1.94		
MYO15A		0.21				
MYO1B		3.49				
MYO5A				1.73		
MYOG					0.29	
MYOM2		0.52				
MYOM3			0.48			
MYST1			6.86			
MYST4		0.49				

NAB2					0.31	
NAGPA						1.78
NANOG				0.5		0.43
NANOS1			0.17			
NANS						2.29
NAP1L4		4.14				
NAPG						1.99
NASP						2.24
NAV3						2
NBL1			6.04			
NCBP2						2
NCK2						1.59
NCOR1						0.54
NDFIP1						2.31
NDOR1					0.47	
NDRG2		0.33			0.53	
NDUFA1		0.43				
NDUFA2		0.41				
NDUFA3					0.47	
NDUFA4		0.26				
NDUFA6	0.21	0.18	0.22			
NDUFA9						1.55
NDUFB1		0.39				
NDUFB10			2.28			

NDUFB2	0.66	0.61	0.66			
NDUFB4			1.83			
NDUFB5						2.25
NDUFC2						0.47
NDUFS2		0.43				
NDUFS6			1.96			
NDUFV1			2.02			
NEBL			0.3			0.63
NEDD4L				1.97		4.45
NEK1				1.81		
NEU2			2.93			
NEU3		0.21				
NEU4					1.5	1.74
NEUROG1					0.54	
NEUROG3						1.69
NF1					0.38	
NF2		1.7				
NFATC2		0.4				
NFE2L1		0.51			0.57	0.62
NFE2L3						2.76
NFIB		3.69				0.38
NFIC				1.58	1.67	2.44
NFKBIE						1.89
NFKBIL1						1.81

NFS1			0.34			
NFYA						1.53
NIN						0.45
NISCH					0.3	
NIT1			0.56			
NKG7						2.41
NKIRAS2						1.7
NKX2-8						1.95
NLK						1.56
NME4	5.46		2.03			
NME7						2.03
NMI			2.14			
NMT1	0.49					1.55
NMU						0.47
NMUR1					0.31	
NNAT			14.43			
NOL5A		6.89				
NOLA2	0.66					
NOLA3	0.5	0.47				
NOLC1						2.3
NOTCH2		0.36	0.46			
NOX4						3.02
NPAS2			0.22			
NPL						3.06

NPM1					0.61	2.01
NPR1						1.5
NPR3						0.5
NPTX1						0.63
NPY1R						0.33
NQO1		0.47	0.42			
NQO2		0.41				
NR1D1						0.51
NR1H2					0.58	
NR2F6			2.09			
NR4A1					0.43	
NR4A3					1.88	4.67
NRAS						2.6
NRP2						1.78
NT5C3				1.61		1.74
NT5E						2.92
NTF5						0.41
NTN1						1.55
NTRK1		0.4				
NTRK2					0.62	
NTRK3			2.57			
NTS			2.52			
NUCB2						2.19
NUDT14			5.89			

NUDT6						1.54
NUMBL					0.55	
NUP107		2.33	2.06			
NUP93						1.93
NYX					0.57	
OAS3						2.88
OGFR					0.48	
OGT					0.47	0.58
OMG		0.05	0.16			
OPA1						3.68
OPHN1					0.65	
OPN3						3.74
OR10H2					0.39	
OR12D3					0.51	
ORC1L				1.5		2.11
ORMDL2	0.41		0.45			
ORMDL3						0.61
OSBPL2					0.59	0.51
OSBPL6						0.61
OSBPL9						2.09
OSGEPL1						1.61
OSTF1			0.52			
OSTM1						2.24
OXR1					0.59	

OXT					0.38	
P2RX4						1.75
P2RXL1	2.55					
P2RY6						1.61
P4HB			3.18			
PA2G4	0.38					
PABPC1			8.99			0.66
PABPC3			26.98			0.62
PADI4		0.33				
PAIP2						2.12
PANK1		0.41				
PANK2						1.86
PANX1						2.76
PANX3					0.6	
PAPLN						1.5
PAQR6					0.58	0.66
PARK7						1.59
PARP1						1.75
PARP8						1.83
PARVA			3.46			
PARVB		0.46	0.38		0.53	
PASK						1.62
PAX3						1.9
PBEF1						5.08

PBX4			0.41			
PBXIP1			2.56			
PCAF						1.76
PCCB						1.87
PCDH11X					1.57	
PCDH12						1.77
PCMT1						2.31
PCOLCE2	0.39		0.54			
PCSK6		0.35	0.37			
PCTK2						1.84
PDCD4					0.54	
PDCD8						1.87
PDE11A						0.37
PDE2A	1.9					
PDE4DIP				1.61		2.57
PDE6D						2.12
PDE7A						1.63
PDGFC					1.5	
PDHA2						0.66
PDK1		0.53				
PDK2			2.44			
PDPK1				1.68		
PELO		0.34	0.4			
PEPD		0.59				

PERLD1						2
PERP						0.54
PEX11B	0.49		0.37			
PFAS			6.95			
PFKFB1					1.5	
PFKFB4						2
PGAM2			3.69			
PGK1			2.41			2.74
PGM2						0.59
PGM2L1						2.03
PGRMC2						0.62
PHC2		2.81				1.59
PHCA				1.85		
PHF16						0.57
PHF19						2.04
PHF2						1.5
PHKA2			0.42			
PHLDB1			2.71			
PHTF1						2.56
PHTF2						1.63
PHYHIP						0.66
PIAS2					0.66	
PIAS3					0.66	
PIGB						1.87

PIGF						1.73
PIGL			2.76			
PIGR					0.59	
PIK3R3				2.03		2.46
PILRB			0.57			
PINK1		0.4	0.28			
PIP5K2A						2.02
PIP5K2C					0.58	
PIP5K3	3					
PITPNA						1.52
PITPNC1						2.59
PITX1						2.05
PKIA						0.56
PKN2					0.59	0.55
PLA2G2E					1.5	1.64
PLA2G4B						0.6
PLA2G7						3.55
PLAC8						3.37
PLAG1	4.78					
PLCB1						1.5
PLD1				0.63		0.66
PLD3		4.78	4.91			
PLDN		2.45				
PLEC1						1.56

PLEK						3.39
PLEKHA1						0.41
PLEKHB2						2.42
PLEKHC1						1.84
PLEKHG1		0.19				
PLG						0.66
PLOD3						0.63
PLP1						2.42
PLUNC						1.5
PMCH			0.13			
PMCHL1		0.41				
PMFBP1		0.19				
PML						2.66
PMM2						1.97
PMS2L2						1.95
PMS2L3						1.97
PNPT1						2.72
PNRC1						0.63
PNRC2		1.78				
POFUT1				0.42		0.62
POLDIP3					0.64	
POLE			2.95		0.54	
POLE2	3.06					
POLE4						2.46

POLG			2.02			
POLR1D		0.49				
POLR2B						1.73
POLR3K						1.67
POLS	0.41	0.43				
PON2						2.52
POP4			0.59			
POP7		0.49				
PORCN					0.6	
POSTN		14.42				
POU4F1						1.5
PPARBP				1.7		
PPAT				2.27		1.68
PPIB			3.07			
PPIE						1.77
PPIF			3.27			
PPIL5						2.12
PPL					0.62	
PPM1A		0.54	0.36			
PPM1E	3.76					
PPP1CA		1.8				
PPP1R11					0.55	1.81
PPP1R13B		0.47	0.39			
PPP1R14A			8.17			

PPP1R14D					1.59	
PPP1R3B	0.43		0.45			
PPP1R3C					0.35	0.47
PPP2CA						2.01
PPP2R2C						0.55
PPP2R5A		0.33				
PPP2R5C						2
PPP3R1						1.5
PPP5C						2.26
PPT1		2.55				
PPT2						1.81
PRAF2					0.57	
PRAME						4.45
PRDM1			3.42			
PRDM2			0.14			
PRDX1						2.44
PRDX3					0.5	3.09
PRDX4	2.05	2.05				
PRDX5			4.03			
PRDX6	0.53					
PREI3						2.01
PRELP		0.27	0.22			
PRG1						4.35
PRG4		0.13				

PRIM1						1.55
PRKACA			5.67			
PRKCBP1						1.5
PRKCG		0.22				
PRKCH						1.51
PRM1					0.57	0.66
PRND						0.63
PRNP			0.32			
PROCR	0.56					
PRODH			2.5			
PROP1					0.66	
PROS1						2.76
PRPF4	4.03		3.35			
PRSS16					0.37	0.46
PRSS22		6.11				
PRSS23			4.99			
PRX						1.71
PSAP			10.88			
PSCD3						2.54
PSCD4					1.71	3.03
PSCDBP						2.08
PSD4			9.27			
PSMA2						2.08
PSMA4				1.71		3.14

PSMA5						1.85
PSMB8			34.74			2.48
PSMC2						1.59
PSMC6						2.19
PSMD12	2.66					2.77
PSMD14						2.17
PSMD3						0.66
PSMD4						1.64
PSMD8		0.41				
PSME3					0.56	
PSMF1			2.87			
PSORS1C2					0.44	0.66
PSTPIP1						1.71
PTBP1			6.95			
PTGIS						0.4
PTK2						1.59
PTK2B			10.61			
PTMA						0.65
PTOV1			10.51			
PTP4A2						2.78
PTPLA						2.01
PTPN2						2.52
PTPN21						0.28
PTPN4						1.69

PTPN6						1.94
PTPRC						3.98
PTPRF						0.38
PTPRN2				0.53	0.47	
PVR		2.98				
PWP2H						2.08
QTRT1			4.62			
RAB11FIP1		0.64				
RAB14				2.2		0.64
RAB1A			0.38	2.05		3.18
RAB1B			4.39			1.5
RAB2						2.28
RAB26		0.29				
RAB27A						3.12
RAB28		4.25				1.67
RAB2B						1.5
RAB31				1.76		3.11
RAB32						2.32
RAB3D			2.08		0.55	0.45
RAB3IP						0.53
RAB5C						1.88
RAB6B				1.53		
RAB6C		3.22				
RABGAP1L						2.18

RABGEF1						1.84
RABIF						1.73
RABL3						1.68
RAD17						2.12
RAD50						1.9
RAD51L1						1.53
RAGE			5.37			
RALA				1.54		1.69
RALBP1		0.24				0.51
RALGDS						0.52
RAN						2.87
RANBP10	0.16					
RANBP2				1.82		
RAP1A				1.59		2.44
RAP1GDS1						2.64
RAPGEF1					0.52	
RAPGEF5		0.45				
RAPGEFL1						0.31
RARA					0.45	
RARRES1						2.28
RARRES2			3.12			
RASA2	2.84					
RASGRP3						1.79
RASSF1			7.55			

RASSF4						2.21
RBBP4			3.27			2.38
RBBP9	0.35					
RBL1						1.83
RBM10			3.74			
RBM11		0.22				
RBM17				1.58		
RBM18						1.5
RBM21						2.24
RBM4			2.91			
RBMS1						2.96
RBMS2					0.63	
RBMX2						2.74
RBPSUHL	3.67					
RCHY1						2.42
RCL1						0.56
RCOR3						1.64
RDH13			0.43			
RDS			0.16			
RDX						2.6
REC8L1					1.82	
RECQL						1.8
REG1A			0.31			
REL				1.64		

RFC4		1.91				2.35
RFP2						0.56
RFXAP						1.93
RGMA					0.55	0.66
RGS16						2.09
RGS20						3.15
RGS4						1.5
RGS5		1.87				
RHBDL2						0.42
RHEB						2.14
RHO					0.42	0.58
RHOBTB1						0.62
RHOG					0.42	
RHOH						1.68
RHOJ						1.7
RILP			0.51			1.63
RIMS4						1.5
RIN2				0.62		
RIOK1				1.62		1.73
RIPK2						2.47
RIPK5				2.18		
RNASE6						2.1
RNASEH1	0.38	0.44	0.45			1.96
RNF10	2.4					

RNF12						2.08
RNF128			0.15			
RNF135	0.53					
RNF141					0.5	0.51
RNF144						1.78
RNF146		0.11				
RNF167						0.61
RNF32					0.66	
RNF36						1.62
RNF38					0.66	
RNF7		0.49				
ROBO2				1.63		
RPA3	0.43					
RPE						1.79
RPGR			0.17			
RPH3A					0.65	
RPL10A						0.59
RPL11		0.46				0.61
RPL14	0.39	0.36			0.64	0.55
RPL19				0.52		
RPL22						0.55
RPL24						0.52
RPL28					0.57	
RPL31						0.56

RPL32				0.51		0.44
RPL34						0.56
RPL36						0.51
RPL36AL			2.48			
RPL7					0.62	0.57
RPL9		0.29			0.59	0.65
RPLP2						0.55
RPP38			0.49			
RPS11						0.66
RPS13					0.62	0.54
RPS17		0.51				
RPS19			5.18			
RPS2						1.57
RPS25						0.57
RPS26		0.48				
RPS27		0.36				
RPS27A						0.58
RPS28						0.61
RPS29						0.57
RPS5			9.68			
RPS6						0.5
RPS6KA3						2.29
RPS6KA4			2.2			
RPS6KA6		2.08				

RPS6KC1						2.08
RPS8						0.52
RPS9			16.24			
RRAGA		0.47	0.34			
RRAGD			0.26			
RRAS			5.03			0.66
RRAS2						1.57
RRM1		1.62				
RRM2						3.53
RTCD1						1.5
RTN3		0.29	0.32		0.5	0.48
RTN4IP1						1.66
RTTN						1.65
RUFY1			0.37			
RUNX1	2.82	3.25				0.62
RUNX3						1.66
RUSC1						1.5
S100A13			1.81			
S100A8						2.07
SAFB2				1.53		1.79
SAMHD1				2.02		3.39
SAMSN1						10.7
SAP18		0.55				1.62
SARDH					1.5	

SAT						2.65
SBNO1				1.56		1.61
SCAMP1						1.66
SCARB2						3.36
SCEL						0.44
SCFD2	4.51					
SCGB1A1					0.56	
SCGB1D2			0.05			
SCGB2A1		0.07	0.18			
SCGB2A2		0.1				
SCGB3A2					0.57	
SCN1B					1.51	
SCN3B						0.58
SCO1						2.25
SCO2			10.76			
SCP2					0.57	
SCRN3						1.95
SCRT1						1.83
SCT						1.5
SCYE1						1.73
SDC2		5.09				
SDCBP						3.12
SDCCAG8						1.56
SDHB						1.59

SDK1						1.61
SDSL						2.56
SEC24A	2.16					
SEC31L1						0.56
SECTM1						0.59
SEL1L				1.61		
SEMA3B						1.9
SEMA4A					0.57	
SEMA4F						1.83
SEMA6A						3.81
SENP8						1.6
SEPHS2			1.57			
SEPW1			7.39			
SERPINA6						0.58
SERPINB1						2.3
SERPINB8						0.66
SERPINB9	2.72					
SESTD1						2.05
SF3A1			2.48		0.48	
SF3A2			2.06		0.41	
SF3B1		2.76				
SF3B3						0.62
SFRS1						1.73
SFRS12			0.4			

SFRS7					0.56	
SFTPB						1.6
SFTPC					0.63	
SFXN1						1.71
SGCA					0.48	
SGCD						1.61
SGK						2.33
SGTB						1.65
SH2D1A						2.42
SH3BGRL2				1.67		
SH3RF2						0.66
SHANK2	5.31		2.16			
SHB						1.87
SHC1						3.25
SHCBP1						2.77
SIAH1						1.83
SILV			15.3			
SIP1						1.62
SIPA1						2.02
SIPA1L3						0.56
SIRT2					0.63	
SIRT5					0.66	
SIX4				0.5		
SLA2					1.5	1.68

SLAMF8						2.06
SLC12A5						0.65
SLC13A2						1.5
SLC16A14						0.48
SLC16A3						2.11
SLC16A6						3.47
SLC16A7						0.4
SLC16A9		10.97				
SLC17A7		0.55			0.56	
SLC1A2			3.64			
SLC1A3						2.41
SLC1A6				1.5		
SLC20A1		2.72				3.92
SLC22A18		0.35				
SLC22A6		4.29	8.33			
SLC23A2	1.85					
SLC24A3						0.59
SLC25A20	0.41					
SLC25A25			0.52			
SLC25A27						1.67
SLC25A28	0.62					
SLC25A4		0.32				
SLC26A11			0.4			
SLC26A4						1.92

SLC26A7						0.53
SLC2A1						0.55
SLC2A3						1.63
SLC2A4						0.63
SLC2A4RG						0.48
SLC2A5					0.64	
SLC30A1						1.62
SLC30A5						2.74
SLC30A6						1.53
SLC31A2						0.65
SLC35B3						1.81
SLC35F2						0.61
SLC35F5						1.83
SLC38A1			0.5			
SLC38A5			4.98			
SLC38A6						2.39
SLC39A11						1.5
SLC39A2	0.22		0.26			
SLC43A3						3.33
SLC4A1AP	1.82					
SLC5A10						0.63
SLC6A6				0.49		
SLC7A11			0.33			
SLC7A7						1.82

SLC9A3						1.54
SLCO2B1						2.12
SLITRK6						0.41
SLMAP			0.29			
SMAD1						0.55
SMAD2						1.75
SMARCA5						2.59
SMARCC2					0.44	0.66
SMARCE1						2.44
SMOC1					0.66	
SMOX					0.5	
SMPD3	4.43					
SMS			2.14			2.48
SMURF1						0.59
SMYD3			3.95			1.9
SNAPAP	0.6					
SNAPC2			3.12			
SNCA						3.04
SNCG						1.51
SNRP70					0.56	
SNRPB			5.59			
SNRPB2		1.89				
SNRPC			4.97			
SNRPD2			2.5			

SNRPE			1.82			
SNTA1						1.84
SNX10		5.11		2.34		4.49
SNX11			0.32			
SNX13						1.59
SNX14						2.68
SNX17			1.82			
SNX25			0.44			1.64
SNX26					0.46	
SNX4		0.37				
SNX5						1.89
SOCS1					0.48	
SOCS3						2.84
SOD2						2.71
SORT1						1.83
SOX11		5.12	5.97			
SOX17			0.14			
SOX18		3.49				
SOX3						1.81
SOX4			5.03			2.86
SOX8					0.62	
SP100						1.99
SP110						2.23
SP2					0.57	

SPARC		3.45	3.41			
SPARCL1			0.16			
SPATA2		0.17				
SPDEF						1.5
SPESP1	0.33	0.22				
SPINK1						0.61
SPINK2						0.64
SPN					0.43	
SPOP			6.69			0.51
SPP1						16.43
SPR						1.96
SPRY1			0.36			
SPTBN2					0.41	
SPTLC2						2.76
SRA1						1.91
SRC						0.59
SRCRB4D			4.75			
SRD5A1		0.3				
SRP68			4.92			
SRP72						2.11
SRP9						2.83
SRPX		2.25				
SSBP1						0.44
SSH3						0.66

SSNA1						2.01
SSPN		5.49				
SSRP1					0.56	
SSTR1						0.6
SSTR3					0.57	
SSX2IP			1.94			
ST13					0.46	
ST7						2.22
STAG1						1.69
STAG3				0.65		
STAR						0.32
STARD10			3.44		0.63	
STARD3NL						1.93
STARD7		0.62				
STAT1						4.86
STCH						2.42
STK11IP						1.5
STK17A						3.26
STK31						0.66
STK33	0.36					
STK38		3.24	4.6			1.57
STK38L	4.49	3.39	17.6			
STK39	1.59					
STK4				2.09		

STMN1		3.22				
STOML2			2.86			
STRA13		0.52				
STRAP						2.5
STRN3		2.15				
STRN4					0.55	
STX11						1.5
STX18						1.57
STX6						1.94
STX7						2.13
STXBP2					1.87	
SUFU					0.43	0.49
SUGT1		0.59		1.64		2.14
SUHW2					0.52	
SULF2		3.46				
SULT1A3		0.52				
SULT1C2			3.13			
SUMO1		3.35				
SUPT3H						2.66
SUPT4H1		0.35	0.33			
SUPT5H	0.5					
SURF6					0.54	
SUV39H2						1.74
SYAP1					1.55	1.68

SYCP1		3.26				
SYNCRIP	1.93					
SYNE2				2.19		
SYNGR1					0.66	
SYNJ1	2.91					
SYNJ2BP						1.52
SYT12						1.72
SYT7			2.9			
SYT9					0.43	
SYTL2						2.61
TAC1					0.35	0.41
TACC2					0.44	
TACSTD1	7.17	6.96	7.47			0.47
TADA2L						2.02
TAF12	0.36	0.41				
TAF1A						1.91
TAF2						2.36
TAF9						1.9
TAGAP						2.53
TAGLN		4.31		1.88		
TAL2		0.25				
TAP2						0.58
TARBP2						1.71
TAS2R1			0.21			

TBC1D1	2.02					
TBC1D15						1.56
TBC1D17			4.75			
TBC1D7						2.98
TBX2						1.89
TBX21		3.79				
TCAP						1.97
TCF20				1.51		
TCF7L1		2.49				
TCN2			3.6			
TCP11						0.66
TEAD3					0.53	
TECTA				0.49		
TEGT						2.22
TEP1						1.51
TESK2			0.56			
TEX11					1.5	
TEX261					0.46	
TFAP2B						0.43
TFB1M						1.5
TFB2M						3.09
TFEC						3.3
TGIF	2.72					
TGM2			2.73			1.57

THADA			0.31			
THAP8		0.32				
THBS1						1.73
THBS3						0.61
THRA						0.5
THRAP1						1.5
THRAP2						0.59
THRAP4						0.58
THRSP		0.33	0.24			
THSD1			2.13			
THUMPD1				1.65		
THUMPD2			0.11			
THUMPD3			0.31			1.76
THY1	2.21					
TIAM1					0.52	
TICAM2						1.78
TIGD7		6.06				
TIMM17A						1.97
TIMM8A						1.65
TIMM8B		0.33				
TIMP2						2.5
TLE4					0.51	
TLK1						2.29
TLR2						2.17

TLR3	0.23	0.31	0.29			
TLR4						2.22
TLR7						3.26
TLR8						3.3
TM7SF3						2.15
TM7SF4						1.95
TM9SF2						2.05
TMEM14A						2.52
TMEM2						1.95
TMEM25						0.46
TMEM33						2.26
TMEM5				1.5		1.63
TMEM8						1.8
TMLHE		0.12				
TMPO						2
TMSB10	3.39		4.47			
TNFAIP6				1.51		3.24
TNFRSF10B						1.86
TNFRSF19L					0.44	
TNFRSF1B						1.5
TNFRSF25						0.37
TNFRSF6B			7.97			
TNFSF12		0.29				
TNFSF4	0.44		0.35			5.41

TNNI3K	0.05	0.08				
TNNT3			4.66			
TNP1					0.54	0.64
TNPO1	3.06					
TNXB					1.55	1.85
TOMM7		0.45				
TP53BP1						1.73
TP53I11			18.44			
TP53I3						1.85
TP53INP2		0.41				
TP53RK						2.23
TP73L						0.43
TPCN1						2.52
TPI1			7.16			
TPM3						2.35
TPM4						2.25
TPMT						2.25
TPSG1					0.61	0.6
TRAF4						1.66
TRAM2						1.79
TRAPPC3						1.55
TRAPPC6B						1.5
TREM2						2.91
TREX1						2.02

TREX2					1.99	2.21
TRIB1						1.91
TRIB3						1.69
TRIM22						2.18
TRIM29		1.96				
TRIM35						1.55
TRIM37						2.27
TRIM38						2.3
TRIM4					0.5	
TRIM45		0.33				
TRIM7						0.44
TRNT1		4.01				1.94
TRPC4AP		1.98				
TRPM3						1.64
TRPV2						1.96
TRPV5		4.75	9.66			
TRUB1						0.62
TRUB2		0.3				
TSCOT		0.12	0.25			
TSFM	0.49	0.42	0.44			
TSGA14						2.01
TSGA2						0.45
TSNAX						2.46
TST	0.27		0.25			

TTC1	0.56	0.64	0.57			1.8
TTC19		1.5				
TTC8						1.63
TTK						1.81
TTLL1				1.5		
TTR					0.61	0.66
TTTY1					2.05	
TTYH3					0.61	
TUBGCP3						1.62
TUSC4		2.09				
TXN		0.33				
TXNDC4						2.26
TXNDC9						2.43
TXNL4B						2.34
TXNL5			2.02			
TYR						3.12
TYRP1		0.31				
UAP1						3
UBADC1						0.6
UBAP2			0.48			
UBASH3A						1.96
UBD						2.57
UBE2C			3.18			
UBE2G1					0.58	1.73

UBE2G2						0.61
UBE2J1					0.59	
UBE2L6						1.85
UBE2R2					0.63	
UBE2S		2.67	18.43			1.57
UBE2V2						2.04
UBQLN1		0.24				0.64
UBQLN3					0.58	0.66
UBR1						1.63
UBTD1		0.25	0.28			
UBXD4		2.39				
UCHL5						2.72
UCN3					0.62	
UCP2			6.66			2.65
UGCG				2.68		
UGCGL1						2.45
UGCGL2						1.5
UGP2		3.56				
UGT2B4						0.66
ULBP2						2.09
UNC50						1.92
UPF2	0.54					
UPF3B						1.69
UPP1			3.65			2.78

UROD			9			
USH2A				0.61		
USH3A		1.81				
USHBP1						1.73
USP13		0.5		1.51		3.12
USP14						2.66
USP18						2.27
USP34						1.66
USP48				2.14		2.16
USP49	2.06					
USP52	0.19					
USP8						2.28
UXS1			2.01			
VAPA		5.48				
VAV1						2.25
VAV3						0.31
VBP1						2.79
VCY			3.03			
VDAC3						1.99
VEGFB			1.75			
VIPR1						0.66
VIPR2					1.55	
VNN2						2.95
VNN3						1.5

VPS13C		3.64				
VPS29						1.68
VPS33B						1.83
WAC				1.84		
WARS						2.12
WASPIP						3.21
WBP5						3.53
WDFY3						1.76
WDHD1						1.52
WDR12						2.03
WDR13			0.55			
WDR26						1.76
WDR31						1.6
WDR35		6.01				1.5
WDR4		3.18				
WHSC1L1				1.85		
WNT10A						0.54
WNT11					0.56	
WNT16			0.25			0.33
WNT2B						0.55
WNT3						0.29
WNT4						0.29
WRB						1.97
WTAP						2

WWP1			0.39			
WWP2						1.5
XCR1					0.52	
XPC						1.6
XPO1		1.87				
XPO5						1.74
XRCC4						2.02
YAF2		3.69				1.73
YWHAZ						0.64
ZBTB24						2.34
ZBTB5					0.66	
ZCCHC4			0.33			0.62
ZDHHC4			3.58			
ZDHHC9			0.34			
ZFP64						0.6
ZFP95						2.02
ZFYVE16						1.84
ZFYVE21			0.42			
ZFYVE26						0.64
ZFYVE9					0.51	0.54
ZIM3			0.26			
ZMYM1					0.65	
ZMYND10	3.66					
ZNF140						1.93

ZNF185						0.55
ZNF193		3.24				
ZNF225			7.89			
ZNF23						1.66
ZNF232		0.43				
ZNF235						1.57
ZNF26						2.05
ZNF267						2.51
ZNF277			0.36			
ZNF278					0.37	
ZNF281						2.1
ZNF324					0.66	
ZNF330						1.77
ZNF334					0.66	
ZNF350			0.26			
ZNF354A				1.64		
ZNF364						2.06
ZNF37A						1.85
ZNF385					0.6	
ZNF394		0.59				
ZNF395						0.44
ZNF403					0.46	
ZNF406	0.43	0.34				
ZNF408					1.5	1.87

ZNF432			0.47			
ZNF435		2.71				1.56
ZNF45						1.93
ZNF451						1.51
ZNF510			0.28			
ZNF516						0.49
ZNF550						0.58
ZNF553		2.07				
ZNF557			0.31			
ZNF577						0.38
ZNF585A				1.57		
ZNF75						0.6
ZNF8	2.93					
ZNFN1A1						2.64
ZP4						1.83
ZRF1						1.73
ZZZ3						1.96
