# Supplementary material for: A Comparative Study of Gene-Expression Data of Basal Cell Carcinoma and Melanoma Reveals New Insights about the Two Cancers
Source: PLoS One. 2012 Jan 25;7(1):e30750. doi: 10.1371/journal.pone.0030750 (PMC3266277; doi:10.1371/journal.pone.0030750)
Supplement: Table S2 — The enriched pathways by all the cancer types in the study. (PDF) [file pone.0030750.s007.pdf]

Table S2. The enriched pathways by all the cancer types in the study. (each cell in the represents the number of genes differentially expressed in the corresponding cancer type and pathway)

genes	PROSTATE	BREAST	KIDNEY	COLON	STOMACH	LUNG	PANCREASE	SUPERF	MROPHF	NODULAR	DN	RGPM	VGPM
hsa05200:Pathways in cancer	18	18	20	28	29	40	56		16		5		40
hsa04010:MAPK signaling pathway	8			17			28		13	19		10	
hsa05010:Alzheimer's disease									13	8			
hsa05012:Parkinson's disease									13	8			
hsa00190:Oxidative phosphorylation									13	7			
hsa05016:Huntington's disease									12	9			
hsa04510:Focal adhesion	16	15	12	14	22	29	38		10			7	22
hsa04310:Wnt signaling pathway	6			10			19		10				18
hsa04810:Regulation of actin cytoskeleton	9				17		32		9	10			26
hsa04530:Tight junction				9		18	16		8	8			16
hsa03010:Ribosome									6			5	22
hsa04512:ECM-receptor interaction	8	12			16	20	20		6				13
hsa04916:Melanogenesis							13		6	7			12
hsa03320:PPAR signaling pathway		11	11	9		9			6				
hsa04270:Vascular smooth muscle contraction	11			13		17			5	6			
hsa04540:Gap junction				7		9			5	6			
hsa04114:Oocyte meiosis				8			13		5				
hsa04722:Neurotrophin signaling pathway							13		5				
hsa05210:Colorectal cancer	6	5	8	7		10	11		5				
hsa04060:Cytokine-cytokine receptor interaction			14		25	28	34						32
hsa04062:Chemokine signaling pathway			12			18	19					7	29
hsa04142:Lysosome			10	8	10		15						26
hsa04120:Ubiquitin mediated proteolysis												6	23
hsa04144:Endocytosis			11										21
hsa04110:Cell cycle	5	8	9	17	26	23	28						20
hsa00230:Purine metabolism				14	11	17							18
hsa04650:Natural killer cell mediated cytotoxicity			9				16						18
hsa04514:Cell adhesion molecules (CAMs)		9	11		10	18	14						17
hsa04612:Antigen processing and presentation			7										16
hsa04620:Toll-like receptor signaling pathway						10							16
hsa04660:T cell receptor signaling pathway			7			11							16
hsa04670:Leukocyte transendothelial migration		7	12			13	17						15
hsa04012:ErbB signaling pathway				6			15						14
hsa05416:Viral myocarditis		6	5	9		10	11						14
hsa04664:Fc epsilon RI signaling pathway				6		8							12
hsa04666:Fc gamma R-mediated phagocytosis		6	9			11	15						12
hsa04914:Progesterone-mediated oocyte maturation				6	7	10							12
hsa05322:Systemic lupus erythematosus				7									12
hsa00520:Amino sugar and nucleotide sugar metabolism													11

hsa00982:Drug metabolism			6										11
hsa04115:p53 signaling pathway			11	7	13	14	14						11
hsa04210:Apoptosis			6	7			16						11
hsa04621:NOD-like receptor signaling pathway			5				8						11
hsa04640:Hematopoietic cell lineage			6	6		10	13						11
hsa04662:B cell receptor signaling pathway			5				10						11
hsa00980:Metabolism of xenobiotics by cytochrome P450													10
hsa03050:Proteasome					5		7						10
hsa05332:Graft-versus-host disease													10
hsa04672:Intestinal immune network for IgA production				5	6								9
hsa04920:Adipocytokine signaling pathway		6		5									9
hsa05212:Pancreatic cancer	5				8	11	15						9
hsa05217:Basal cell carcinoma					7								9
hsa00010:Glycolysis / Gluconeogenesis		6	10		7	9	11			5			8
hsa04940:Type I diabetes mellitus				5									8
hsa05110:Vibrio cholerae infection						7							8
hsa05320:Autoimmune thyroid disease													8
hsa05330:Allograft rejection													8
hsa00511:Other glycan degradation													7
hsa00591:Linoleic acid metabolism													7
hsa03420:Nucleotide excision repair					5								7
hsa00020:Citrate cycle (TCA cycle)													6
hsa00071:Fatty acid metabolism		6		6									6
hsa00280:Valine, leucine and isoleucine degradation		8	8				6						6
hsa00620:Pyruvate metabolism		8				5							6
hsa00983:Drug metabolism			6										6
hsa03030:DNA replication			5	6	10	8	5						6
hsa04130:SNARE interactions in vesicular transport													6
hsa05310:Asthma													6
hsa05340:Primary immunodeficiency													6
hsa00250:Alanine, aspartate and glutamate metabolism						5							5
hsa00565:Ether lipid metabolism		6		5		7							5
hsa03430:Mismatch repair					6								5
hsa04080:Neuroactive ligand-receptor interaction												7	
hsa04630:Jak-STAT signaling pathway		8				15						7	
hsa04910:Insulin signaling pathway		10					15			9		5	
hsa03040:Spliceosome										8			
hsa04020:Calcium signaling pathway	8			11		16	23			8			
hsa04912:GnRH signaling pathway										7			
hsa05414:Dilated cardiomyopathy	8			7		11	17			6			
hsa04720:Long-term potentiation										5			
hsa04730:Long-term depression				7						5			
hsa00030:Pentose phosphate pathway			7				5						
hsa00040:Pentose and glucuronate interconversions													

hsa00051:Fructose and mannose metabolism			7			7	5						
hsa00052:Galactose metabolism							5						
hsa00240:Pyrimidine metabolism			8	7	10								
hsa00260:Glycine, serine and threonine metabolism			5	5			8						
hsa00270:Cysteine and methionine metabolism							7						
hsa00310:Lysine degradation													
hsa00330:Arginine and proline metabolism				6	5	6							
hsa00340:Histidine metabolism		6											
hsa00350:Tyrosine metabolism			5										
hsa00380:Tryptophan metabolism		6				5							
hsa00410:beta-Alanine metabolism		5	5										
hsa00480:Glutathione metabolism			5	6	5		8						
hsa00500:Starch and sucrose metabolism							6						
hsa00510:N-Glycan biosynthesis						5							
hsa00561:Glycerolipid metabolism		7											
hsa00562:Inositol phosphate metabolism						7							
hsa00564:Glycerophospholipid metabolism		7				7							
hsa00590:Arachidonic acid metabolism				6	5								
hsa00600:Sphingolipid metabolism							5						
hsa00630:Glyoxylate and dicarboxylate metabolism		5											
hsa00640:Propanoate metabolism		8		5			6						
hsa00650:Butanoate metabolism		6											
hsa00910:Nitrogen metabolism				5									
hsa02010:ABC transporters				6		6							
hsa03018:RNA degradation					5								
hsa03410:Base excision repair					6								
hsa04070:Phosphatidylinositol signaling system						8							
hsa04260:Cardiac muscle contraction	5												
hsa04350:TGF-beta signaling pathway	6			9		12							
hsa04360:Axon guidance		9			11	15	27						
hsa04370:VEGF signaling pathway							9						
hsa04520:Adherens junction	6	6				12	16						
hsa04610:Complement and coagulation cascades			9	8			10						
hsa04742:Taste transduction													
hsa04930:Type II diabetes mellitus							6						
hsa04960:Aldosterone-regulated sodium reabsorption		6					6						
hsa05014:Amyotrophic lateral sclerosis (ALS)			5		5								
hsa05020:Prion diseases						5							
hsa05120:Epithelial cell signaling in Helicobacter pylori infection			6				8						
hsa05130:Pathogenic Escherichia coli infection					6	6	11						
hsa05211:Renal cell carcinoma						9	10						
hsa05213:Endometrial cancer							7						
hsa05214:Glioma				6		7	8						
hsa05215:Prostate cancer			6	7		9	11						

hsa05216:Thyroid cancer							6						
hsa05218:Melanoma			6	8	6	9							
hsa05219:Bladder cancer			5	6	8	10	7						
hsa05220:Chronic myeloid leukemia		6		6		8	11						
hsa05221:Acute myeloid leukemia		5					9						
hsa05222:Small cell lung cancer	5			8	11	14	17						
hsa05223:Non-small cell lung cancer					5	7	7						
hsa05410:Hypertrophic cardiomyopathy (HCM)	8			7		11	15						
hsa05412:Arrhythmogenic right ventricular cardiomyopathy (ARVC)	5			6	8	10	19						
