# Supplementary material for: A Comparative Study of Gene-Expression Data of Basal Cell Carcinoma and Melanoma Reveals New Insights about the Two Cancers
Source: PLoS One. 2012 Jan 25;7(1):e30750. doi: 10.1371/journal.pone.0030750 (PMC3266277; doi:10.1371/journal.pone.0030750)
Supplement: Table S3 — The top gene signatures for the skin cancer. (PDF) [file pone.0030750.s008.pdf]

Table S3 (a). The top 1-gene signature for the melanoma. (The 2nd and 3rd column represent the classification accuracy of the gene signature in the training set and independent testing set. The 4th and 5th column represent the prediction result of the urine-secretory [marked by *] and blood-secretory [marked by **])

Melanoma 1-gene signatures				
genes	training accuracy	testing accuracy	urine	blood
OAS3	82.93%	71.43%		**
RALBP1	82.93%	71.43%		
GLA	80.49%	71.43%		
LLGL1	80.49%	71.43%		
SERPINA6	80.49%	71.43%		**
APCS	78.05%	71.43%		**
CD36	78.05%	71.43%		
CYP4B1	78.05%	71.43%		
EXO1	78.05%	71.43%		
GDPD2	78.05%	71.43%		
HOXD9	78.05%	71.43%		
HSPE1	78.05%	71.43%		
KBTBD2	78.05%	71.43%		
PIGR	78.05%	71.43%		
SCEL	78.05%	71.43%		
TP53I3	78.05%	71.43%		
ALDH3A2	75.61%	71.43%		
ARL4A	75.61%	71.43%		
ATP1A2	75.61%	69.84%		
BCL6	75.61%	71.43%		
CD207	75.61%	71.43%		
DLGAP1	75.61%	71.43%		
GATM	75.61%	69.84%		
HAGH	75.61%	71.43%		
HSPA4	75.61%	71.43%		
IFI44	75.61%	71.43%		
MECP2	75.61%	71.43%		
MNDA	75.61%	71.43%		
NOX4	75.61%	71.43%		
PHTF1	75.61%	71.43%		
PKIA	75.61%	71.43%		
RGS20	75.61%	71.43%		
SLC16A6	75.61%	71.43%		
STAR	75.61%	76.19%		**

TNP1	75.61%	71.43%		
WNT4	75.61%	71.43%		**
COX7C	75.61%	71.43%		
RPL34	73.17%	77.78%		
AASDHPPT	73.17%	71.43%		
BEX1	73.17%	71.43%		
BRAP	73.17%	71.43%		
CCL18	73.17%	71.43%	*	**
FCGR2A	73.17%	71.43%		
HEXB	73.17%	71.43%	*	
HMGB1	73.17%	71.43%		**
IFI30	73.17%	71.43%	*	
IVL	73.17%	71.43%		
MAGEC2	73.17%	71.43%		
NT5E	73.17%	71.43%		
PGLYRP4	73.17%	71.43%		**
RAD1	73.17%	71.43%		**
RNF141	73.17%	71.43%		
RUNX3	73.17%	71.43%		
SRC	73.17%	71.43%		
STC1	73.17%	71.43%	*	**
STK24	73.17%	71.43%		
TCP11	73.17%	71.43%		
TFAP2B	73.17%	71.43%		
YWHAB	73.17%	71.43%		

Table S3 (b). The top 2-gene signature for the melanoma. (The 2nd and 3rd column represent the classification accuracy of the gene signature in the training set and independent testing set. The 4th and 5th column represent the prediction result of the urine-secretory [marked by *] and blood-secretory [marked by **])

Melanoma 2-gene signatures				
genes	training accuracy	testing accuracy	urine	blood
GLA_HSPE1	95.12%	71.43%		
GLA_IL13	95.12%	71.43%		
GLA_KBTBD2	95.12%	71.43%		
GLA_LLGL1	95.12%	71.43%		
SERPINA6_VAPA	95.12%	71.43%		
DSP_GLA	92.68%	87.30%		
DSP_IFITM2	92.68%	80.95%		

APCS_LGALS1	92.68%	76.19%		**
LGALS1_LLGL1	92.68%	76.19%		
LLGL1_RNF141	92.68%	76.19%		
CLU_GLA	92.68%	71.43%		
DDB2_IFITM2	92.68%	71.43%		
DNM1_IFITM2	92.68%	71.43%		
EPB41L1_GLA	92.68%	71.43%		
FABP1_GLA	92.68%	71.43%		
GLA_HRSP12	92.68%	71.43%		
GLA_HSPA1L	92.68%	71.43%		
GLA_KCNJ15	92.68%	71.43%		
GLA_MECP2	92.68%	71.43%		
GLA_METAP1	92.68%	71.43%		
GLA_PIGR	92.68%	71.43%		
GLA_PNLIP	92.68%	71.43%		
GLA_SERPINB7	92.68%	71.43%		
GLA_TTR	92.68%	71.43%		
HOXD9_RUNX3	92.68%	71.43%		
IFITM2_TBL2	92.68%	71.43%		
PANX1_RUNX3	92.68%	71.43%		
PMM2_RUNX3	92.68%	71.43%		
RUNX3_WISP3	92.68%	71.43%		
CAPNS1_NOX4	90.24%	82.54%		
CAPNS1_MAGEC2	90.24%	79.37%		
GLA_IMPA2	90.24%	77.78%		
HSPE1_LGALS1	90.24%	76.19%		
KBTBD2_PLAC8	90.24%	76.19%		
APCS_PAK6	90.24%	74.60%		
GLA_GPRASP1	90.24%	74.60%		
GLA_STK39	90.24%	74.60%		
HSPE1_PKIA	90.24%	74.60%		
KBTBD2_LGALS1	90.24%	74.60%		
LGALS1_SRC	90.24%	74.60%		
GLA_RFX2	90.24%	73.02%		
HSPE1_PLAC8	90.24%	73.02%		
HSPE1_TP53I3	90.24%	73.02%		
IVL_SLC10A3	90.24%	73.02%		
LLGL1_TP53I3	90.24%	73.02%		
ADAM17_GATM	90.24%	71.43%		
ANXA5_APCS	90.24%	71.43%		
APCS_EIF4ENIF1	90.24%	71.43%		

APCS_HLA-DMA	90.24%	71.43%		
APCS_IKBKE	90.24%	71.43%		
APCS_LAMC1	90.24%	71.43%		**
APCS_NKG7	90.24%	71.43%		
APCS_OAS3	90.24%	71.43%		**
APCS_PSME1	90.24%	71.43%		
APOL6_GATM	90.24%	71.43%		
AQP6_STAR	90.24%	71.43%		
ARL4A_S100A9	90.24%	71.43%		
ATP5H_RUNX3	90.24%	71.43%		
BEX1_GJB5	90.24%	71.43%		
C11orf24_MNDA	90.24%	71.43%		
C12orf5_SQLE	90.24%	71.43%		
CBFA2T2_EIF4ENIF1	90.24%	71.43%		
CDK4_GLA	90.24%	71.43%		
CHST11_SCEL	90.24%	71.43%		
CKS2_GLA	90.24%	71.43%		
COL17A1_GLA	90.24%	71.43%		
COX7C_CYP4B1	90.24%	71.43%		
CPEB3_GLA	90.24%	71.43%		
CPEB3_LLGL1	90.24%	71.43%		
CPEB3_MNDA	90.24%	71.43%		
CYP4B1_LLGL1	90.24%	71.43%		
CYP4B1_STMN1	90.24%	71.43%		
DDAH1_HEXB	90.24%	71.43%		
DEK_GLA	90.24%	71.43%		
DHX15_HSPE1	90.24%	71.43%		
EIF4ENIF1_LLGL1	90.24%	71.43%		
ESRRA_LLGL1	90.24%	71.43%		
ETF1_PLAC8	90.24%	71.43%		
FURIN_SALL2	90.24%	71.43%		
GATM_KCNAB3	90.24%	71.43%		
GHITM_GLA	90.24%	71.43%		
GLA_HLA-DPB1	90.24%	71.43%		
GLA_KHDRBS3	90.24%	71.43%		
GLA_KRT10	90.24%	71.43%		
GLA_LIN7A	90.24%	71.43%		
GLA_NUP155	90.24%	71.43%		
GLA_PEPD	90.24%	71.43%		
GLA_PKIA	90.24%	71.43%		
GLA_SCO2	90.24%	71.43%		

GLA_SILV	90.24%	71.43%		
GLA_SLC1A3	90.24%	71.43%		
GLA_SPAG5	90.24%	71.43%		
GLA_SPRR1B	90.24%	71.43%		
GLA_STAR	90.24%	71.43%		
GLA_STMN2	90.24%	71.43%		
GLA_TNP1	90.24%	71.43%		
HLA-DMA_LLGL1	90.24%	71.43%		
HLA-DPB1_HSPE1	90.24%	71.43%		
HRASLS_IFITM2	90.24%	71.43%		
HSPE1_PSME1	90.24%	71.43%		
HSPE1_TRPV2	90.24%	71.43%		
IFI30_KBTBD2	90.24%	71.43%		
IFITM2_ITGB3BP	90.24%	71.43%		
IFITM2_MAGEC2	90.24%	71.43%		
IFITM2_ZNF26	90.24%	71.43%		
IL22RA1_PIGR	90.24%	71.43%		
IL6R_KBTBD2	90.24%	71.43%		
INSL5_RUNX3	90.24%	71.43%		
IVL_SERPINA6	90.24%	71.43%		
KBTBD2_MRPS11	90.24%	71.43%		
KIF5C_SH3YL1	90.24%	71.43%		
LETMD1_RUNX3	90.24%	71.43%		
LLGL1_LMO3	90.24%	71.43%		
LLGL1_PEX11B	90.24%	71.43%		
LLGL1_PIM2	90.24%	71.43%		
LLGL1_PLAC8	90.24%	71.43%		
LLGL1_SLAMF7	90.24%	71.43%		
MAGEC2_PSME1	90.24%	71.43%		
MNDA_RUNX3	90.24%	71.43%		
MRPS28_RUNX3	90.24%	71.43%		
NIT1_RUNX3	90.24%	71.43%		
NOX4_SPINK2	90.24%	71.43%		
PIGR_ZFPL1	90.24%	71.43%		
RIMS3_RPL34	90.24%	71.43%		
RNASE6_ZNF24	90.24%	71.43%		
SCEL_SLIT1	90.24%	71.43%		
DSP_RALBP1	87.80%	87.30%		
TP53I3_UCN	87.80%	77.78%		
KLKB1_PLAC8	87.80%	76.19%		
LGALS1_MAGEC2	87.80%	76.19%		

CSTB_PLAC8	87.80%	74.60%		
LLGL1_PKIA	87.80%	74.60%		
AKR1B1_RUNX3	87.80%	73.02%		
APCS_FAM32A	87.80%	73.02%		
APCS_PLAC8	87.80%	73.02%		
CHST2_TP53I3	87.80%	73.02%		
CSTB_SCEL	87.80%	73.02%		
CTSK_RNASE6	87.80%	73.02%	*	
FURIN_PLAC8	87.80%	73.02%		
ACVR1_APCS	87.80%	71.43%		
AGA_GLA	87.80%	71.43%		
AKAP10_APCS	87.80%	71.43%		
APCS_C3AR1	87.80%	71.43%		
APCS_CYP4B1	87.80%	71.43%		
APCS_HPRT1	87.80%	71.43%		
APCS_NES	87.80%	71.43%		
APCS_RPE	87.80%	71.43%		
B3GNT3_SALL2	87.80%	71.43%		
BCL6_MRPS7	87.80%	71.43%		
C20orf117_IVL	87.80%	71.43%		
C9orf125_HSPE1	87.80%	71.43%		
C9orf125_LLGL1	87.80%	71.43%		
CCL13_IFITM2	87.80%	71.43%		
CCL27_IFITM2	87.80%	71.43%		
CCRL1_IFITM2	87.80%	71.43%		
CCRL1_LLGL1	87.80%	71.43%		
CD83_PIGR	87.80%	71.43%		
CHST11_RALBP1	87.80%	71.43%		
CKM_IFITM2	87.80%	71.43%		
COL21A1_KBTBD2	87.80%	71.43%		
CORT_GLA	87.80%	71.43%		
CYB561_TP53I3	87.80%	71.43%		
DDB2_GLA	87.80%	71.43%		
DIAPH3_IFITM2	87.80%	71.43%		
DUSP9_GATM	87.80%	71.43%		
DYRK3_IFITM2	87.80%	71.43%		
EIF4ENIF1_KBTBD2	87.80%	71.43%		
EIF4ENIF1_MNDA	87.80%	71.43%		
FABP4_KBTBD2	87.80%	71.43%		
FCGR2A_KIF5C	87.80%	71.43%		
FCGR2A_TNP1	87.80%	71.43%		

GATM_GFI1B	87.80%	71.43%		
GATM_MUC13	87.80%	71.43%		
GATM_TEX13B	87.80%	71.43%		
GATM_TNC	87.80%	71.43%		
GATM_TRPV6	87.80%	71.43%		
GLA_PRM1	87.80%	71.43%		
GLA_SSB	87.80%	71.43%		
GLA_TYRP1	87.80%	71.43%		
HERC5_RAMP1	87.80%	71.43%		
HERC5_RPL34	87.80%	71.43%		
HIST1H1C_RPL34	87.80%	71.43%		
HOXD9_SALL2	87.80%	71.43%		
HSD17B3_PIGR	87.80%	71.43%		
HSPA6_HSPE1	87.80%	71.43%		
HSPE1_IFI30	87.80%	71.43%		
HSPE1_LIPA	87.80%	71.43%		
HSPE1_MX2	87.80%	71.43%		
HSPE1_NES	87.80%	71.43%		
HSPE1_NKG7	87.80%	71.43%		
HSPE1_OAS3	87.80%	71.43%		
HSPE1_PAK6	87.80%	71.43%		
ID4_IVL	87.80%	71.43%		
IFITM2_IVL	87.80%	71.43%		
IFITM2_PKIA	87.80%	71.43%		
IFITM2_PLUNC	87.80%	71.43%		
IFITM2_SLC1A2	87.80%	71.43%		
IFITM2_SNX5	87.80%	71.43%		
KBTBD2_OAS3	87.80%	71.43%		
KBTBD2_ODF2	87.80%	71.43%		
KBTBD2_PIM2	87.80%	71.43%		
KBTBD2_UBASH3A	87.80%	71.43%		
LCP2_LLGL1	87.80%	71.43%		
LLGL1_OSTF1	87.80%	71.43%		
LRCH1_PIGR	87.80%	71.43%		
MNDA_MPV17	87.80%	71.43%		
MNDA_MXD3	87.80%	71.43%		
MNDA_PIGR	87.80%	71.43%		
OAS3_TBL2	87.80%	71.43%		
P2RY2_TNP1	87.80%	71.43%		
PPRC1_SCEL	87.80%	71.43%		
RAI2_TNP1	87.80%	71.43%		

RUNX3_SAMHD1	87.80%	71.43%		
RUNX3_SCYE1	87.80%	71.43%		
RUNX3_ZNF330	87.80%	71.43%		
SDC2_TP53I3	87.80%	71.43%		
SERPINA6_TP53I3	87.80%	71.43%		
TCP11_YWHAB	87.80%	71.43%		
TNP1_WNT4	87.80%	71.43%		

Table S3 (c). The top 1-gene signature for the BCC. (The 2nd and 3rd column represent the classification accuracy of the gene signature in the training set and independent testing set. The 4th and 5th column represent the prediction result of the urine-secretory [marked by *] and blood-secretory [marked by **])

BCC 1-gene signatures				
genes	Training accuracy	Testing accuracy	urine	blood
CS	0.903226	0.87		
TACSTD1	0.870968	0.87		
EGR1	0.870968	0.8125		**
MAGEA9	0.870968	0.8125		
CPOX	0.870968	0.75		
KEAP1	0.83871	0.8125		
MLF1	0.83871	0.8125		**
PCSK6	0.83871	0.8125		**
PINK1	0.83871	0.8125		
RNASEH1	0.83871	0.8125		
SLC39A2	0.83871	0.8125		**
TTC1	0.83871	0.8125		
CLPTM1	0.83871	0.8125		
ANXA7	0.83871	0.75		
CLIC4	0.83871	0.75		
SNAPC2	0.83871	0.75		**
TNNI2	0.83871	0.75		**
AMOTL2	0.83871	0.6875		
GNAI2	0.83871	0.625		
APOD	0.806452	0.8		**
LMNA	0.806452	0.8		
AKR1C1	0.806452	0.8		
DUSP2	0.806452	0.8		
MYH2	0.806452	0.8		
RBBP4	0.806452	0.8		

BASP1	0.806452	0.8		**
PPM1A	0.806452	0.8		
RAB3D	0.806452	0.8	*	
ATP1A2	0.806452	0.75		
DTX2	0.806452	0.75		
FVT1	0.806452	0.75		
MGST2	0.806452	0.75		
NDUFA6	0.806452	0.75		
NDUFB2	0.806452	0.75		
PRNP	0.806452	0.75		
C21orf25	0.774194	0.75		
FADS2	0.774194	0.75		
B4GALT3	0.774194	0.75		
IMPDH2	0.774194	0.75		
MIF	0.774194	0.75		**
RRAGA	0.774194	0.75		
C9orf58	0.774194	0.75		
CRYAA	0.774194	0.75		
DLGAP4	0.774194	0.75		
ERF	0.774194	0.75		
GNAI3	0.774194	0.75		
GRB7	0.774194	0.75		
LGALS12	0.774194	0.75		**
NBL1	0.774194	0.75		
PPP1R13B	0.774194	0.75		
RAB1B	0.774194	0.75		
RARRES2	0.774194	0.75		**
RPS19	0.774194	0.75		
SMS	0.774194	0.75		
VCY	0.774194	0.75		

Table S3 (d). The top 2-gene signature for the BCC. (The 2nd and 3rd column represent the classification accuracy of the gene signature in the training set and independent testing set. The 4th and 5th column represent the prediction result of the urine-secretory [marked by *] and blood-secretory [marked by **])

BCC 2-gene signatures				
genes	Training accuracy	Testing accuracy	urine	blood
ANXA7_NAP1L4	96.77%	93.75%		
HMGCR_MAGEA9	96.77%	93.75%		

MAGEA9_SOX4	96.77%	81.25%		
COBL_TLR3	93.55%	87.50%		
EGR1_TNNI2	93.55%	87.50%		
FGF20_TLR3	93.55%	87.50%		
ING4_TACSTD1	93.55%	87.50%		
MRPS24_TLR3	93.55%	87.50%		
SFRS16_TACSTD1	93.55%	87.50%		
SLC25A28_TACSTD1	93.55%	87.50%		
ACBD6_CS	93.55%	81.25%		
ANXA7_EGR1	93.55%	81.25%		
TACSTD1_TLR8	93.55%	81.25%		
AMOTL2_MRPS24	93.55%	75.00%		
HMGCR_TLR3	90.32%	87.50%		
BRD4_TACSTD1	90.32%	87.50%		
COL6A2_TACSTD1	90.32%	87.50%		
CS_FRG1	90.32%	87.50%		
SOX4_TACSTD1	90.32%	87.50%		
ANXA7_ARHGAP26	90.32%	87.50%		
ANXA7_ERF	90.32%	87.50%		
ANXA7_MYH2	90.32%	87.50%		
ANXA7_TACSTD1	90.32%	87.50%		
CS_MYH2	90.32%	87.50%		
EGR1_SLC7A11	90.32%	87.50%		
SPARCL1_TACSTD1	90.32%	87.50%		
ANXA7_PCSK6	90.32%	81.25%		
ANXA7_TLR3	90.32%	81.25%		
CAPZA2_TLR3	90.32%	81.25%		
CD3D_TACSTD1	90.32%	81.25%		
HRASLS_TLR3	90.32%	81.25%		
NEU4_TACSTD1	90.32%	81.25%		
AMOTL2_C20orf103	90.32%	75.00%		
ANXA7_CLIC4	90.32%	75.00%		
EGR1_RARRES2	90.32%	75.00%		**
MPHOSPH10_TLR3	90.32%	75.00%		
TACSTD1_TRPC4	90.32%	75.00%		
AMOTL2_POP4	87.10%	81.25%		
ANXA7_CNP	87.10%	81.25%		
CS_RPL36AL	87.10%	81.25%		
ACSL4_EGR1	87.10%	81.25%		
ANXA7_PPP1R13B	87.10%	81.25%		
ELAC2_MAGEA9	87.10%	81.25%		

LDHA_TACSTD1	87.10%	81.25%		
MRP63_TACSTD1	87.10%	81.25%		
OSTF1_TACSTD1	87.10%	81.25%		
RFX2_TACSTD1	87.10%	81.25%		
RPS9_TACSTD1	87.10%	81.25%		
TACSTD1_TNNI2	87.10%	81.25%		**
ABCD3_ANXA7	87.10%	81.25%		
ANXA7_PSMC4	87.10%	81.25%		
COL18A1_EGR1	87.10%	81.25%		**
CS_ELK1	87.10%	81.25%		
EGR1_GNAI3	87.10%	81.25%		
EGR1_MRPS24	87.10%	81.25%		
GALT_TACSTD1	87.10%	81.25%		
MSH2_TLR3	87.10%	81.25%		**
PSMB5_TACSTD1	87.10%	81.25%		
SUPT5H_TACSTD1	87.10%	81.25%		
C20orf103_TLR3	87.10%	81.25%		
COL18A1_CS	87.10%	81.25%		
COL18A1_TACSTD1	87.10%	81.25%		
CSTB_MAGEA9	87.10%	81.25%		
CS_DGCR2	87.10%	81.25%		
CS_MRPS24	87.10%	81.25%		
EGR1_METAP1	87.10%	81.25%		
FABP5_TACSTD1	87.10%	81.25%		
NRN1_TACSTD1	87.10%	81.25%		
ANXA7_MRPS24	87.10%	75.00%		
BTN3A3_EGR1	87.10%	75.00%		
CAPZA2_EGR1	87.10%	75.00%		
COL11A2_EGR1	87.10%	75.00%		**
CSF1R_EGR1	87.10%	75.00%		
EGR1_MYBBP1A	87.10%	75.00%		
EGR1_RFX2	87.10%	75.00%		
EGR1_SFRS11	87.10%	75.00%		
MAGEA9_SPARCL1	87.10%	75.00%		
