# Supplementary material for: A Comparative Study of Gene-Expression Data of Basal Cell Carcinoma and Melanoma Reveals New Insights about the Two Cancers
Source: PLoS One. 2012 Jan 25;7(1):e30750. doi: 10.1371/journal.pone.0030750 (PMC3266277; doi:10.1371/journal.pone.0030750)
Supplement: Table S4 — A summary of the cancer data used in our analysis. (PDF) [file pone.0030750.s009.pdf]

Table S4: A summary of the cancer data used in our analysis

Cancer	GEO dataset ID<br>training / testing data	# reference/ #cancer samples
breast cancer	GSE15852[1] / GSE10810 [2]	43/43 (27/31)
colon cancer	GSE6988 [3] / GSE10950 [4]	28/53 (24/24)
kidney cancer	GSE15641[5] / GSE17816 [6]	23/49 (9/36)
lung cancer	GSE10072 [7] / GSE7670 [8]	49/58 (27/27)
pancreatic cancer	GSE15471 [9] / GSE16515 [10]	39/39 (16/36)
prostate cancer	GSE6606 [11] / GSE3933 [12]	63/65 (47/62)
stomach cancer	GSE2701 [13] / GSE13911 [14]	23/89 (31/38)
Basal Cell Carcinoma	GSE6520 [15] / GSE12542[16]	8/23 (8/8)
Melanoma	GSE12931 [17] / GSE3189[18]	18/34 (7/45)

1. Pau Ni, I.B., et al., *Gene expression patterns distinguish breast carcinomas from normal breast tissues: the Malaysian context.* Pathol Res Pract, 2010. **206**(4): p. 223-8.
2. Pedraza, V., et al., *Gene expression signatures in breast cancer distinguish phenotype characteristics, histologic subtypes, and tumor invasiveness.* Cancer, 2010. **116**(2): p. 486-96.
3. Ki, D.H., et al., *Whole genome analysis for liver metastasis gene signatures in colorectal cancer.* Int J Cancer, 2007. **121**(9): p. 2005-12.
4. Jiang, X., et al., *DACT3 is an epigenetic regulator of Wnt/beta-catenin signaling in colorectal cancer and is a therapeutic target of histone modifications.* Cancer Cell, 2008. **13**(6): p. 529-41.
5. Jones, J., et al., *Gene signatures of progression and metastasis in renal cell cancer.* Clin Cancer Res, 2005. **11**(16): p. 5730-9.
6. Dalgliesh, G.L., et al., *Systematic sequencing of renal carcinoma reveals inactivation of histone modifying genes.* Nature, 2010. **463**(7279): p. 360-3.
7. Landi, M.T., et al., *Gene expression signature of cigarette smoking and its role in lung adenocarcinoma development and survival.* PLoS One, 2008. **3**(2): p. e1651.
8. Su, L.J., et al., *Selection of DDX5 as a novel internal control for Q-RT-PCR from microarray data using a block bootstrap re-sampling scheme.* BMC Genomics, 2007. **8**: p. 140.
9. Badea, L., et al., *Combined gene expression analysis of whole-tissue and microdissected pancreatic ductal adenocarcinoma identifies genes specifically overexpressed in tumor epithelia.* Hepatogastroenterology, 2008. **55**(88): p. 2016-27.
10. Pei, H., et al., *FKBP51 affects cancer cell response to chemotherapy by negatively regulating Akt.* Cancer Cell, 2009. **16**(3): p. 259-66.
11. Chandran, U.R., et al., *Gene expression profiles of prostate cancer reveal involvement of multiple molecular pathways in the metastatic process.* BMC Cancer, 2007. **7**: p. 64.
12. Lapointe, J., et al., *Gene expression profiling identifies clinically relevant subtypes of prostate cancer.* Proc Natl Acad Sci U S A, 2004. **101**(3): p. 811-6.
13. Chen, X., et al., *Variation in gene expression patterns in human gastric cancers.* Molecular biology of the cell, 2003. **14**(8): p. 3208-15.
14. D'Errico, M., et al., *Genome-wide expression profile of sporadic gastric cancers with microsatellite instability.* Eur J Cancer, 2009. **45**(3): p. 461-9.
15. Lo, B.K., et al., *CXCR3/ligands are significantly involved in the tumorigenesis of basal cell carcinomas.* Am J Pathol, 2010. **176**(5): p. 2435-46.
16. Yu, M., et al., *Superficial, nodular, and morpheiform basal-cell carcinomas exhibit distinct gene expression profiles.* J Invest Dermatol, 2008. **128**(7): p. 1797-805.
17. Scatolini, M., et al., *Altered molecular pathways in melanocytic lesions.* Int J Cancer, 2010. **126**(8): p. 1869-81.
18. Talantov, D., et al., *Novel genes associated with malignant melanoma but not benign melanocytic lesions.* Clin Cancer Res, 2005. **11**(20): p. 7234-42.
