# Supplementary material for: A Comparative Study of Gene-Expression Data of Basal Cell Carcinoma and Melanoma Reveals New Insights about the Two Cancers
Source: PLoS One. 2012 Jan 25;7(1):e30750. doi: 10.1371/journal.pone.0030750 (PMC3266277; doi:10.1371/journal.pone.0030750)
Supplement: Table S5 — The common genes shared by the datasets used in this study. (PDF) [file pone.0030750.s010.pdf]

Table S5. The common gene shared by the datasets used in this study.

Gene ID	Gene Name	Gene Symbol	Species
65985	acetoacetyl-CoA synthetase	AACS	Homo sapiens
13	arylacetamide deacetylase (esterase)	AADAC	Homo sapiens
22848	AP2 associated kinase 1	AAK1	Homo sapiens
14	angio-associated, migratory cell protein	AAMP	Homo sapiens
16	alanyl-tRNA synthetase	AARS	Homo sapiens
10157	aminoadipate-semialdehyde synthase	AASS	Homo sapiens
26574	apoptosis antagonizing transcription factor	AATF	Homo sapiens
18	4-aminobutyrate aminotransferase	ABAT	Homo sapiens
19	ATP-binding cassette, sub-family A (ABC1), member 1	ABCA1	Homo sapiens
21	ATP-binding cassette, sub-family A (ABC1), member 3	ABCA3	Homo sapiens
24	ATP-binding cassette, sub-family A (ABC1), member 4	ABCA4	Homo sapiens
23461	ATP-binding cassette, sub-family A (ABC1), member 5	ABCA5	Homo sapiens
10351	ATP-binding cassette, sub-family A (ABC1), member 8	ABCA8	Homo sapiens
5243	ATP-binding cassette, sub-family B (MDR/TAP), member 1	ABCB1	Homo sapiens
5244	ATP-binding cassette, sub-family B (MDR/TAP), member 4	ABCB4	Homo sapiens
10058	ATP-binding cassette, sub-family B (MDR/TAP), member 6	ABCB6	Homo sapiens
22	ATP-binding cassette, sub-family B (MDR/TAP), member 7	ABCB7	Homo sapiens
11194	ATP-binding cassette, sub-family B (MDR/TAP), member 8	ABCB8	Homo sapiens
23457	ATP-binding cassette, sub-family B (MDR/TAP), member 9	ABCB9	Homo sapiens
4363	ATP-binding cassette, sub-family C (CFTR/MRP), member 1	ABCC1	Homo sapiens
89845	ATP-binding cassette, sub-family C (CFTR/MRP), member 10	ABCC10	Homo sapiens
1244	ATP-binding cassette, sub-family C (CFTR/MRP), member 2	ABCC2	Homo sapiens
10057	ATP-binding cassette, sub-family C (CFTR/MRP), member 5	ABCC5	Homo sapiens
6833	ATP-binding cassette, sub-family C (CFTR/MRP), member 8	ABCC8	Homo sapiens
215	ATP-binding cassette, sub-family D (ALD), member 1	ABCD1	Homo sapiens
5826	ATP-binding cassette, sub-family D (ALD), member 4	ABCD4	Homo sapiens
6059	similar to ATP-binding cassette, sub-family E, member 1; ATP-binding cassette, sub-family E (OABP), member 1	ABCE1	Homo sapiens
647150	similar to ATP-binding cassette, sub-family E, member 1; ATP-binding cassette, sub-family E (OABP), member 1	ABCE1	Homo sapiens
55324	ATP-binding cassette, sub-family F (GCN20), member 3	ABCF3	Homo sapiens
9619	ATP-binding cassette, sub-family G (WHITE), member 1	ABCG1	Homo sapiens
9429	ATP-binding cassette, sub-family G (WHITE), member 2	ABCG2	Homo sapiens
64240	ATP-binding cassette, sub-family G (WHITE), member 5	ABCG5	Homo sapiens
171586	abhydrolase domain containing 3	ABHD3	Homo sapiens
63874	abhydrolase domain containing 4	ABHD4	Homo sapiens
51099	abhydrolase domain containing 5	ABHD5	Homo sapiens
10152	abl interactor 2	ABI2	Homo sapiens
3983	actin binding LIM protein 1	ABLIM1	Homo sapiens
22885	actin binding LIM protein family, member 3	ABLIM3	Homo sapiens
25841	ankyrin repeat and BTB (POZ) domain containing 2	ABTB2	Homo sapiens
30	acetyl-Coenzyme A acyltransferase 1	ACAA1	Homo sapiens
10449	hypothetical LOC648603; acetyl-Coenzyme A acyltransferase 2	ACAA2	Homo sapiens
648603	hypothetical LOC648603; acetyl-Coenzyme A acyltransferase 2	ACAA2	Homo sapiens

32	acetyl-Coenzyme A carboxylase beta	ACACB	Homo sapiens
27034	acyl-Coenzyme A dehydrogenase family, member 8	ACAD8	Homo sapiens
33	acyl-Coenzyme A dehydrogenase, long chain	ACADL	Homo sapiens
34	acyl-Coenzyme A dehydrogenase, C-4 to C-12 straight chain	ACADM	Homo sapiens
35	acyl-Coenzyme A dehydrogenase, C-2 to C-3 short chain	ACADS	Homo sapiens
36	acyl-Coenzyme A dehydrogenase, short/branched chain	ACADSB	Homo sapiens
37	acyl-Coenzyme A dehydrogenase, very long chain	ACADVL	Homo sapiens
38	acetyl-Coenzyme A acetyltransferase 1	ACAT1	Homo sapiens
39	acetyl-Coenzyme A acetyltransferase 2	ACAT2	Homo sapiens
41	amiloride-sensitive cation channel 2, neuronal	ACCN2	Homo sapiens
1636	angiotensin I converting enzyme (peptidyl-dipeptidase A) 1	ACE	Homo sapiens
43	acetylcholinesterase (Yt blood group)	ACHE	Homo sapiens
47	ATP citrate lyase	ACLY	Homo sapiens
48	aconitase 1, soluble	ACO1	Homo sapiens
51	acyl-Coenzyme A oxidase 1, palmitoyl	ACOX1	Homo sapiens
8309	acyl-Coenzyme A oxidase 2, branched chain	ACOX2	Homo sapiens
53	acid phosphatase 2, lysosomal	ACP2	Homo sapiens
54	acid phosphatase 5, tartrate resistant	ACP5	Homo sapiens
51205	acid phosphatase 6, lysophosphatidic	ACP6	Homo sapiens
55	acid phosphatase, prostate	ACPP	Homo sapiens
49	acrosin	ACR	Homo sapiens
56	acrosomal vesicle protein 1	ACRV1	Homo sapiens
2181	acyl-CoA synthetase long-chain family member 3	ACSL3	Homo sapiens
2182	acyl-CoA synthetase long-chain family member 4	ACSL4	Homo sapiens
51703	acyl-CoA synthetase long-chain family member 5	ACSL5	Homo sapiens
23305	acyl-CoA synthetase long-chain family member 6	ACSL6	Homo sapiens
59	actin, alpha 2, smooth muscle, aorta	ACTA2	Homo sapiens
72	actin, gamma 2, smooth muscle, enteric	ACTG2	Homo sapiens
86	actin-like 6A	ACTL6A	Homo sapiens
89	actinin, alpha 3	ACTN3	Homo sapiens
10121	ARP1 actin-related protein 1 homolog A, centractin alpha (yeast)	ACTR1A	Homo sapiens
90	activin A receptor, type I	ACVR1	Homo sapiens
91	activin A receptor, type IB	ACVR1B	Homo sapiens
93	activin A receptor, type IIB	ACVR2B	Homo sapiens
94	activin A receptor type II-like 1	ACVRL1	Homo sapiens
97	acylphosphatase 1, erythrocyte (common) type	ACYP1	Homo sapiens
100	adenosine deaminase	ADA	Homo sapiens
102	ADAM metallopeptidase domain 10	ADAM10	Homo sapiens
4185	ADAM metallopeptidase domain 11	ADAM11	Homo sapiens
8038	ADAM metallopeptidase domain 12	ADAM12	Homo sapiens
8751	ADAM metallopeptidase domain 15	ADAM15	Homo sapiens
6868	ADAM metallopeptidase domain 17	ADAM17	Homo sapiens
8749	ADAM metallopeptidase domain 18	ADAM18	Homo sapiens
53616	ADAM metallopeptidase domain 22	ADAM22	Homo sapiens
8745	ADAM metallopeptidase domain 23	ADAM23	Homo sapiens
10863	ADAM metallopeptidase domain 28	ADAM28	Homo sapiens

11085	ADAM metallopeptidase domain 30	ADAM30	Homo sapiens
80332	ADAM metallopeptidase domain 33	ADAM33	Homo sapiens
8754	ADAM metallopeptidase domain 9 (meltrin gamma)	ADAM9	Homo sapiens
27299	ADAM-like, decysin 1	ADAMDEC1	Homo sapiens
9510	ADAM metallopeptidase with thrombospondin type 1 motif, 1	ADAMTS1	Homo sapiens
81792	ADAM metallopeptidase with thrombospondin type 1 motif, 12	ADAMTS12	Homo sapiens
9508	ADAM metallopeptidase with thrombospondin type 1 motif, 3	ADAMTS3	Homo sapiens
11096	ADAM metallopeptidase with thrombospondin type 1 motif, 5	ADAMTS5	Homo sapiens
11174	ADAM metallopeptidase with thrombospondin type 1 motif, 6	ADAMTS6	Homo sapiens
103	adenosine deaminase, RNA-specific	ADAR	Homo sapiens
104	adenosine deaminase, RNA-specific, B1 (RED1 homolog rat)	ADARB1	Homo sapiens
107	adenylate cyclase 1 (brain)	ADCY1	Homo sapiens
108	adenylate cyclase 2 (brain)	ADCY2	Homo sapiens
196883	adenylate cyclase 4	ADCY4	Homo sapiens
113	adenylate cyclase 7	ADCY7	Homo sapiens
115	adenylate cyclase 9	ADCY9	Homo sapiens
118	adducin 1 (alpha)	ADD1	Homo sapiens
119	adducin 2 (beta)	ADD2	Homo sapiens
120	adducin 3 (gamma)	ADD3	Homo sapiens
130	alcohol dehydrogenase 6 (class V)	ADH6	Homo sapiens
51094	adiponectin receptor 1	ADIPOR1	Homo sapiens
132	adenosine kinase	ADK	Homo sapiens
133	adrenomedullin	ADM	Homo sapiens
23394	activity-dependent neuroprotector homeobox	ADNP	Homo sapiens
135	adenosine A2a receptor	ADORA2A	Homo sapiens
136	hypothetical LOC100131909; adenosine A2b receptor	ADORA2B	Homo sapiens
100131909	hypothetical LOC100131909; adenosine A2b receptor	ADORA2B	Homo sapiens
140	adenosine A3 receptor	ADORA3	Homo sapiens
147	adrenergic, alpha-1B-, receptor	ADRA1B	Homo sapiens
146	adrenergic, alpha-1D-, receptor	ADRA1D	Homo sapiens
154	adrenergic, beta-2-, receptor, surface	ADRB2	Homo sapiens
155	adrenergic, beta-3-, receptor	ADRB3	Homo sapiens
156	adrenergic, beta, receptor kinase 1	ADRBK1	Homo sapiens
157	adrenergic, beta, receptor kinase 2	ADRBK2	Homo sapiens
11047	adhesion regulating molecule 1	ADRM1	Homo sapiens
158	adenylosuccinate lyase	ADSL	Homo sapiens
159	adenylosuccinate synthase	ADSS	Homo sapiens
165	AE binding protein 1	AEBP1	Homo sapiens
174	alpha-fetoprotein	AFP	Homo sapiens
175	aspartylglucosaminidase	AGA	Homo sapiens
177	advanced glycosylation end product-specific receptor	AGER	Homo sapiens
178	amylo-1, 6-glucosidase, 4-alpha-glucanotransferase	AGL	Homo sapiens
10555	1-acylglycerol-3-phosphate O-acyltransferase 2 (lysophosphatidic acid acyltransferase, beta)	AGPAT2	Homo sapiens
8540	alkylglycerone phosphate synthase	AGPS	Homo sapiens
10551	anterior gradient homolog 2 (Xenopus laevis)	AGR2	Homo sapiens

183	angiotensinogen (serpin peptidase inhibitor, clade A, member 8)	AGT	Homo sapiens
185	angiotensin II receptor, type 1	AGTR1	Homo sapiens
10768	adenosylhomocysteinase-like 1	AHCYL1	Homo sapiens
196	aryl hydrocarbon receptor	AHR	Homo sapiens
10598	AHA1, activator of heat shock 90kDa protein ATPase homolog 1 (yeast)	AHSA1	Homo sapiens
197	alpha-2-HS-glycoprotein	AHSG	Homo sapiens
199	allograft inflammatory factor 1	AIF1	Homo sapiens
202	absent in melanoma 1	AIM1	Homo sapiens
9447	absent in melanoma 2	AIM2	Homo sapiens
9049	aryl hydrocarbon receptor interacting protein	AIP	Homo sapiens
26289	adenylate kinase 5	AK5	Homo sapiens
8165	A kinase (PRKA) anchor protein 1	AKAP1	Homo sapiens
11216	A kinase (PRKA) anchor protein 10	AKAP10	Homo sapiens
11215	A kinase (PRKA) anchor protein 11	AKAP11	Homo sapiens
9590	A kinase (PRKA) anchor protein 12	AKAP12	Homo sapiens
11214	A kinase (PRKA) anchor protein 13	AKAP13	Homo sapiens
10566	A kinase (PRKA) anchor protein 3	AKAP3	Homo sapiens
9472	A kinase (PRKA) anchor protein 6	AKAP6	Homo sapiens
26993	A kinase (PRKA) anchor protein 8-like	AKAP8L	Homo sapiens
10142	A kinase (PRKA) anchor protein (yotiao) 9	AKAP9	Homo sapiens
10327	aldo-keto reductase family 1, member A1 (aldehyde reductase)	AKR1A1	Homo sapiens
231	aldo-keto reductase family 1, member B1 (aldose reductase)	AKR1B1	Homo sapiens
57016	aldo-keto reductase family 1, member B10 (aldose reductase); aldo-keto reductase family 1, member B10-like	AKR1B10	Homo sapiens
441282	aldo-keto reductase family 1, member B10 (aldose reductase); aldo-keto reductase family 1, member B10-like	AKR1B10	Homo sapiens
1645	aldo-keto reductase family 1, member C1 (dihydrodiol dehydrogenase 1; 20-alpha (3-alpha)-hydroxysteroid dehydrogenase)	AKR1C1	Homo sapiens
1109	aldo-keto reductase family 1, member C4 (chlordecone reductase; 3-alpha hydroxysteroid dehydrogenase, type I; dihydrodiol dehydrogenase 4)	AKR1C4	Homo sapiens
6718	aldo-keto reductase family 1, member D1 (delta 4-3-ketosteroid-5-beta-reductase)	AKR1D1	Homo sapiens
8574	aldo-keto reductase family 7, member A2 (aflatoxin aldehyde reductase)	AKR7A2	Homo sapiens
10000	v-akt murine thymoma viral oncogene homolog 3 (protein kinase B, gamma)	AKT3	Homo sapiens
210	aminolevulinate, delta-, dehydratase	ALAD	Homo sapiens
211	aminolevulinate, delta-, synthase 1	ALAS1	Homo sapiens
212	aminolevulinate, delta-, synthase 2	ALAS2	Homo sapiens
213	albumin	ALB	Homo sapiens
214	hypothetical protein LOC100133690; activated leukocyte cell adhesion molecule	ALCAM	Homo sapiens
100133690	hypothetical protein LOC100133690; activated leukocyte cell adhesion molecule	ALCAM	Homo sapiens
216	aldehyde dehydrogenase 1 family, member A1	ALDH1A1	Homo sapiens
8854	aldehyde dehydrogenase 1 family, member A2	ALDH1A2	Homo sapiens

219	aldehyde dehydrogenase 1 family, member B1	ALDH1B1	Homo sapiens
217	aldehyde dehydrogenase 2 family (mitochondrial)	ALDH2	Homo sapiens
218	aldehyde dehydrogenase 3 family, memberA1	ALDH3A1	Homo sapiens
224	aldehyde dehydrogenase 3 family, member A2	ALDH3A2	Homo sapiens
222	aldehyde dehydrogenase 3 family, member B2	ALDH3B2	Homo sapiens
7915	aldehyde dehydrogenase 5 family, member A1	ALDH5A1	Homo sapiens
4329	aldehyde dehydrogenase 6 family, member A1	ALDH6A1	Homo sapiens
501	aldehyde dehydrogenase 7 family, member A1	ALDH7A1	Homo sapiens
226	aldolase A, fructose-bisphosphate	ALDOA	Homo sapiens
229	aldolase B, fructose-bisphosphate	ALDOB	Homo sapiens
230	aldolase C, fructose-bisphosphate	ALDOC	Homo sapiens
56052	asparagine-linked glycosylation 1, beta-1,4-mannosyltransferase homolog (S. cerevisiae)	ALG1	Homo sapiens
10195	asparagine-linked glycosylation 3, alpha-1,3- mannosyltransferase homolog (S. cerevisiae)	ALG3	Homo sapiens
79053	asparagine-linked glycosylation 8, alpha-1,3-glucosyltransferase homolog (S. cerevisiae)	ALG8	Homo sapiens
239	arachidonate 12-lipoxygenase	ALOX12	Homo sapiens
247	arachidonate 15-lipoxygenase, type B	ALOX15B	Homo sapiens
241	arachidonate 5-lipoxygenase-activating protein	ALOX5AP	Homo sapiens
23600	C1q and tumor necrosis factor related protein 3; alpha-methylacyl-CoA racemase	AMACR	Homo sapiens
114899	C1q and tumor necrosis factor related protein 3; alpha-methylacyl-CoA racemase	AMACR	Homo sapiens
262	adenosylmethionine decarboxylase 1	AMD1	Homo sapiens
267	autocrine motility factor receptor	AMFR	Homo sapiens
154796	angiomotin	AMOT	Homo sapiens
270	adenosine monophosphate deaminase 1 (isoform M)	AMPD1	Homo sapiens
272	adenosine monophosphate deaminase (isoform E)	AMPD3	Homo sapiens
273	amphiphysin	AMPH	Homo sapiens
729198	anaphase promoting complex subunit 10; anaphase promoting complex subunit 10 pseudogene	ANAPC10	Homo sapiens
10393	anaphase promoting complex subunit 10; anaphase promoting complex subunit 10 pseudogene	ANAPC10	Homo sapiens
25847	anaphase promoting complex subunit 13	ANAPC13	Homo sapiens
29882	anaphase promoting complex subunit 2	ANAPC2	Homo sapiens
51433	anaphase promoting complex subunit 5	ANAPC5	Homo sapiens
283	angiogenin, ribonuclease, RNase A family, 5	ANG	Homo sapiens
284	angiopoietin 1	ANGPT1	Homo sapiens
285	angiopoietin 2	ANGPT2	Homo sapiens
27329	angiopoietin-like 3	ANGPTL3	Homo sapiens
51129	angiopoietin-like 4	ANGPTL4	Homo sapiens
287	ankyrin 2, neuronal	ANK2	Homo sapiens
288	ankyrin 3, node of Ranvier (ankyrin G)	ANK3	Homo sapiens
27063	ankyrin repeat domain 1 (cardiac muscle)	ANKRD1	Homo sapiens
23253	ankyrin repeat domain 12	ANKRD12	Homo sapiens
84168	anthrax toxin receptor 1	ANTXR1	Homo sapiens
301	annexin A1	ANXA1	Homo sapiens

11199	annexin A10	ANXA10	Homo sapiens
311	annexin A11	ANXA11	Homo sapiens
306	annexin A3	ANXA3	Homo sapiens
307	annexin A4	ANXA4	Homo sapiens
308	annexin A5	ANXA5	Homo sapiens
310	annexin A7	ANXA7	Homo sapiens
8416	annexin A9	ANXA9	Homo sapiens
314	amine oxidase, copper containing 2 (retina-specific)	AOC2	Homo sapiens
8639	amine oxidase, copper containing 3 (vascular adhesion protein 1)	AOC3	Homo sapiens
316	aldehyde oxidase 1	AOX1	Homo sapiens
162	adaptor-related protein complex 1, beta 1 subunit	AP1B1	Homo sapiens
164	adaptor-related protein complex 1, gamma 1 subunit	AP1G1	Homo sapiens
1174	adaptor-related protein complex 1, sigma 1 subunit	AP1S1	Homo sapiens
161	adaptor-related protein complex 2, alpha 2 subunit	AP2A2	Homo sapiens
1175	adaptor-related protein complex 2, sigma 1 subunit	AP2S1	Homo sapiens
8546	adaptor-related protein complex 3, beta 1 subunit	AP3B1	Homo sapiens
8120	adaptor-related protein complex 3, beta 2 subunit	AP3B2	Homo sapiens
8943	adaptor-related protein complex 3, delta 1 subunit	AP3D1	Homo sapiens
10239	adaptor-related protein complex 3, sigma 2 subunit	AP3S2	Homo sapiens
23431	adaptor-related protein complex 4, epsilon 1 subunit	AP4E1	Homo sapiens
9179	adaptor-related protein complex 4, mu 1 subunit	AP4M1	Homo sapiens
321	amyloid beta (A4) precursor protein-binding, family A, member 2	APBA2	Homo sapiens
9546	amyloid beta (A4) precursor protein-binding, family A, member 3	APBA3	Homo sapiens
324	adenomatous polyposis coli	APC	Homo sapiens
10297	adenomatosis polyposis coli 2	APC2	Homo sapiens
325	amyloid P component, serum	APCS	Homo sapiens
328	APEX nuclease (multifunctional DNA repair enzyme) 1	APEX1	Homo sapiens
27301	APEX nuclease (apurinic/apyrimidinic endonuclease) 2	APEX2	Homo sapiens
642812	API5-like 1; apoptosis inhibitor 5	API5	Homo sapiens
8539	API5-like 1; apoptosis inhibitor 5	API5	Homo sapiens
333	amyloid beta (A4) precursor-like protein 1	APLP1	Homo sapiens
334	amyloid beta (A4) precursor-like protein 2	APLP2	Homo sapiens
335	apolipoprotein A-I	APOA1	Homo sapiens
338	apolipoprotein B (including Ag(x) antigen)	APOB	Homo sapiens
339	apolipoprotein B mRNA editing enzyme, catalytic polypeptide 1	APOBEC1	Homo sapiens
345	apolipoprotein C-III	APOC3	Homo sapiens
346	apolipoprotein C-IV	APOC4	Homo sapiens
347	apolipoprotein D	APOD	Homo sapiens
100129500	hypothetical LOC100129500; apolipoprotein E	APOE	Homo sapiens
348	hypothetical LOC100129500; apolipoprotein E	APOE	Homo sapiens
23780	apolipoprotein L, 2	APOL2	Homo sapiens
55937	apolipoprotein M	APOM	Homo sapiens
351	amyloid beta (A4) precursor protein	APP	Homo sapiens
10513	amyloid beta precursor protein (cytoplasmic tail) binding protein 2	APPBP2	Homo sapiens
353	adenine phosphoribosyltransferase	APRT	Homo sapiens
358	aquaporin 1 (Colton blood group)	AQP1	Homo sapiens

359	aquaporin 2 (collecting duct)	AQP2	Homo sapiens
360	aquaporin 3 (Gill blood group)	AQP3	Homo sapiens
361	aquaporin 4	AQP4	Homo sapiens
363	aquaporin 6, kidney specific	AQP6	Homo sapiens
364	aquaporin 7	AQP7	Homo sapiens
366	aquaporin 9	AQP9	Homo sapiens
367	androgen receptor	AR	Homo sapiens
372	archain 1	ARCN1	Homo sapiens
375	ADP-ribosylation factor 1	ARF1	Homo sapiens
377	ADP-ribosylation factor 3	ARF3	Homo sapiens
378	ADP-ribosylation factor 4	ARF4	Homo sapiens
381	ADP-ribosylation factor 5	ARF5	Homo sapiens
382	ADP-ribosylation factor 6	ARF6	Homo sapiens
10565	ADP-ribosylation factor guanine nucleotide-exchange factor 1(brefeldin A-inhibited)	ARFGEF1	Homo sapiens
10564	ADP-ribosylation factor guanine nucleotide-exchange factor 2 (brefeldin A-inhibited)	ARFGEF2	Homo sapiens
27236	ADP-ribosylation factor interacting protein 1	ARFIP1	Homo sapiens
23647	ADP-ribosylation factor interacting protein 2	ARFIP2	Homo sapiens
10139	ADP-ribosylation factor related protein 1	ARFRP1	Homo sapiens
383	arginase, liver	ARG1	Homo sapiens
384	arginase, type II	ARG2	Homo sapiens
392	Rho GTPase activating protein 1	ARHGAP1	Homo sapiens
23092	Rho GTPase activating protein 26	ARHGAP26	Homo sapiens
394	Rho GTPase activating protein 5	ARHGAP5	Homo sapiens
395	Rho GTPase activating protein 6	ARHGAP6	Homo sapiens
397	Rho GDP dissociation inhibitor (GDI) beta	ARHGDIB	Homo sapiens
398	Rho GDP dissociation inhibitor (GDI) gamma	ARHGDIG	Homo sapiens
9138	Rho guanine nucleotide exchange factor (GEF) 1	ARHGEF1	Homo sapiens
9826	Rho guanine nucleotide exchange factor (GEF) 11	ARHGEF11	Homo sapiens
27237	Rho guanine exchange factor (GEF) 16	ARHGEF16	Homo sapiens
50650	Rho guanine nucleotide exchange factor (GEF) 3	ARHGEF3	Homo sapiens
50649	Rho guanine nucleotide exchange factor (GEF) 4	ARHGEF4	Homo sapiens
7984	Rho guanine nucleotide exchange factor (GEF) 5	ARHGEF5	Homo sapiens
9459	Rac/Cdc42 guanine nucleotide exchange factor (GEF) 6	ARHGEF6	Homo sapiens
8874	Rho guanine nucleotide exchange factor (GEF) 7	ARHGEF7	Homo sapiens
100134381	Cdc42 guanine nucleotide exchange factor (GEF) 9; hypothetical protein LOC100134381	ARHGEF9	Homo sapiens
23229	Cdc42 guanine nucleotide exchange factor (GEF) 9; hypothetical protein LOC100134381	ARHGEF9	Homo sapiens
8289	AT rich interactive domain 1A (SWI-like)	ARID1A	Homo sapiens
25820	ariadne homolog, ubiquitin-conjugating enzyme E2 binding protein, 1 (Drosophila)	ARIH1	Homo sapiens
10425	ariadne homolog 2 (Drosophila)	ARIH2	Homo sapiens
400	ADP-ribosylation factor-like 1	ARL1	Homo sapiens
23568	ADP-ribosylation factor-like 2 binding protein	ARL2BP	Homo sapiens
403	ADP-ribosylation factor-like 3	ARL3	Homo sapiens

10550	ADP-ribosylation-like factor 6 interacting protein 5	ARL6IP5	Homo sapiens
25852	armadillo repeat containing 8	ARMC8	Homo sapiens
405	aryl hydrocarbon receptor nuclear translocator	ARNT	Homo sapiens
406	aryl hydrocarbon receptor nuclear translocator-like	ARNTL	Homo sapiens
10552	actin related protein 2/3 complex, subunit 1A, 41kDa	ARPC1A	Homo sapiens
10109	actin related protein 2/3 complex, subunit 2, 34kDa	ARPC2	Homo sapiens
10092	actin related protein 2/3 complex, subunit 5, 16kDa	ARPC5	Homo sapiens
407	arrestin 3, retinal (X-arrestin)	ARR3	Homo sapiens
410	arylsulfatase A	ARSA	Homo sapiens
411	arylsulfatase B	ARSB	Homo sapiens
415	arylsulfatase E (chondrodysplasia punctata 1)	ARSE	Homo sapiens
416	arylsulfatase F	ARSF	Homo sapiens
421	armadillo repeat gene deletes in velocardiofacial syndrome	ARVCF	Homo sapiens
427	N-acylsphingosine amidohydrolase (acid ceramidase) 1	ASAH1	Homo sapiens
51666	ankyrin repeat and SOCS box-containing 4	ASB4	Homo sapiens
10973	activating signal cointegrator 1 complex subunit 3	ASCC3	Homo sapiens
429	achaete-scute complex homolog 1 (Drosophila)	ASCL1	Homo sapiens
430	achaete-scute complex homolog 2 (Drosophila)	ASCL2	Homo sapiens
432	asialoglycoprotein receptor 1	ASGR1	Homo sapiens
433	asialoglycoprotein receptor 2	ASGR2	Homo sapiens
9070	ash2 (absent, small, or homeotic)-like (Drosophila)	ASH2L	Homo sapiens
434	agouti signaling protein, nonagouti homolog (mouse)	ASIP	Homo sapiens
435	argininosuccinate lyase	ASL	Homo sapiens
8623	acetylserotonin O-methyltransferase-like	ASMTL	Homo sapiens
439	arsA arsenite transporter, ATP-binding, homolog 1 (bacterial)	ASNA1	Homo sapiens
440	asparagine synthetase	ASNS	Homo sapiens
443	aspartoacylase (Canavan disease)	ASPA	Homo sapiens
171023	additional sex combs like 1 (Drosophila)	ASXL1	Homo sapiens
466	activating transcription factor 1	ATF1	Homo sapiens
1386	activating transcription factor 2	ATF2	Homo sapiens
467	activating transcription factor 3	ATF3	Homo sapiens
22809	activating transcription factor 5	ATF5	Homo sapiens
55729	activating transcription factor 7 interacting protein	ATF7IP	Homo sapiens
471	5-aminoimidazole-4-carboxamide ribonucleotide formyltransferase/IMP cyclohydrolase	ATIC	Homo sapiens
651610	similar to Serine-protein kinase ATM (Ataxia telangiectasia mutated) (A-T, mutated); ataxia telangiectasia mutated	ATM	Homo sapiens
472	similar to Serine-protein kinase ATM (Ataxia telangiectasia mutated) (A-T, mutated); ataxia telangiectasia mutated	ATM	Homo sapiens
475	ATX1 antioxidant protein 1 homolog (yeast)	ATOX1	Homo sapiens
477	ATPase, Na+/K+ transporting, alpha 2 (+) polypeptide	ATP1A2	Homo sapiens
483	ATPase, Na+/K+ transporting, beta 3 polypeptide	ATP1B3	Homo sapiens
488	ATPase, Ca++ transporting, cardiac muscle, slow twitch 2	ATP2A2	Homo sapiens
489	ATPase, Ca++ transporting, ubiquitous	ATP2A3	Homo sapiens
490	ATPase, Ca++ transporting, plasma membrane 1	ATP2B1	Homo sapiens
491	ATPase, Ca++ transporting, plasma membrane 2	ATP2B2	Homo sapiens
492	ATPase, Ca++ transporting, plasma membrane 3	ATP2B3	Homo sapiens

493	ATPase, Ca++ transporting, plasma membrane 4	ATP2B4	Homo sapiens
27032	ATPase, Ca++ transporting, type 2C, member 1	ATP2C1	Homo sapiens
495	ATPase, H+/K+ exchanging, alpha polypeptide	ATP4A	Homo sapiens
496	ATPase, H+/K+ exchanging, beta polypeptide	ATP4B	Homo sapiens
509	ATP synthase, H+ transporting, mitochondrial F1 complex, gamma polypeptide 1	ATP5C1	Homo sapiens
513	ATP synthase, H+ transporting, mitochondrial F1 complex, delta subunit	ATP5D	Homo sapiens
515	ATP synthase, H+ transporting, mitochondrial F0 complex, subunit B1	ATP5F1	Homo sapiens
516	ATP synthase, H+ transporting, mitochondrial F0 complex, subunit C1 (subunit 9)	ATP5G1	Homo sapiens
517	ATP synthase, H+ transporting, mitochondrial F0 complex, subunit C2 (subunit 9)	ATP5G2	Homo sapiens
518	ATP synthase, H+ transporting, mitochondrial F0 complex, subunit C3 (subunit 9)	ATP5G3	Homo sapiens
10476	ATP synthase, H+ transporting, mitochondrial F0 complex, subunit d	ATP5H	Homo sapiens
521	ATP synthase, H+ transporting, mitochondrial F0 complex, subunit E	ATP5I	Homo sapiens
522	ATP synthase, H+ transporting, mitochondrial F0 complex, subunit F6	ATP5J	Homo sapiens
9551	ATP synthase, H+ transporting, mitochondrial F0 complex, subunit F2	ATP5J2	Homo sapiens
10632	ATP synthase, H+ transporting, mitochondrial F0 complex, subunit G	ATP5L	Homo sapiens
10159	ATPase, H+ transporting, lysosomal accessory protein 2	ATP6AP2	Homo sapiens
23545	ATPase, H+ transporting, lysosomal V0 subunit a2	ATP6V0A2	Homo sapiens
533	ATPase, H+ transporting, lysosomal 21kDa, V0 subunit b	ATP6V0B	Homo sapiens
9114	ATPase, H+ transporting, lysosomal 38kDa, V0 subunit d1	ATP6V0D1	Homo sapiens
528	ATPase, H+ transporting, lysosomal 42kDa, V1 subunit C1	ATP6V1C1	Homo sapiens
51382	ATPase, H+ transporting, lysosomal 34kDa, V1 subunit D	ATP6V1D	Homo sapiens
9296	ATPase, H+ transporting, lysosomal 14kDa, V1 subunit F	ATP6V1F	Homo sapiens
9550	ATPase, H+ transporting, lysosomal 13kDa, V1 subunit G1	ATP6V1G1	Homo sapiens
534	ATPase, H+ transporting, lysosomal 13kDa, V1 subunit G2	ATP6V1G2	Homo sapiens
51606	ATPase, H+ transporting, lysosomal 50/57kDa, V1 subunit H	ATP6V1H	Homo sapiens
540	ATPase, Cu++ transporting, beta polypeptide	ATP7B	Homo sapiens
10396	ATPase, aminophospholipid transporter (APLT), class I, type 8A, member 1	ATP8A1	Homo sapiens
5205	ATPase, class I, type 8B, member 1	ATP8B1	Homo sapiens
148229	ATPase, class I, type 8B, member 3	ATP8B3	Homo sapiens
10079	ATPase, class II, type 9A	ATP9A	Homo sapiens
374868	ATPase, class II, type 9B	ATP9B	Homo sapiens
651921	ataxia telangiectasia and Rad3 related; similar to ataxia telangiectasia and Rad3 related protein	ATR	Homo sapiens
648152	ataxia telangiectasia and Rad3 related; similar to ataxia telangiectasia and Rad3 related protein	ATR	Homo sapiens
545	ataxia telangiectasia and Rad3 related; similar to ataxia telangiectasia and Rad3 related protein	ATR	Homo sapiens
8455	attractin	ATRN	Homo sapiens
546	alpha thalassemia/mental retardation syndrome X-linked (RAD54 homolog, S. cerevisiae)	ATRX	Homo sapiens
6310	ataxin 1	ATXN1	Homo sapiens
25814	ataxin 10	ATXN10	Homo sapiens

549	AU RNA binding protein/enoyl-Coenzyme A hydratase	AUH	Homo sapiens
9212	aurora kinase B	AURKB	Homo sapiens
6795	aurora kinase C	AURKC	Homo sapiens
10677	advillin	AVIL	Homo sapiens
552	arginine vasopressin receptor 1A	AVPR1A	Homo sapiens
554	arginine vasopressin receptor 2	AVPR2	Homo sapiens
8312	axin 1	AXIN1	Homo sapiens
8313	axin 2	AXIN2	Homo sapiens
558	AXL receptor tyrosine kinase	AXL	Homo sapiens
646282	alpha-2-glycoprotein 1, zinc-binding pseudogene 1; alpha-2-glycoprotein 1, zinc-binding	AZGP1	Homo sapiens
563	alpha-2-glycoprotein 1, zinc-binding pseudogene 1; alpha-2-glycoprotein 1, zinc-binding	AZGP1	Homo sapiens
566	azurocidin 1	AZU1	Homo sapiens
567	beta-2-microglobulin	B2M	Homo sapiens
8707	UDP-Gal:betaGlcNAc beta 1,3-galactosyltransferase, polypeptide 2	B3GALT2	Homo sapiens
26229	beta-1,3-glucuronyltransferase 3 (glucuronosyltransferase I)	B3GAT3	Homo sapiens
11041	UDP-GlcNAc:betaGal beta-1,3-N-acetylglucosaminyltransferase 1; UDP-GlcNAc:betaGal beta-1,3-N-acetylglucosaminyltransferase 2	B3GNT1	Homo sapiens
10678	UDP-GlcNAc:betaGal beta-1,3-N-acetylglucosaminyltransferase 1; UDP-GlcNAc:betaGal beta-1,3-N-acetylglucosaminyltransferase 2	B3GNT1	Homo sapiens
8703	UDP-Gal:betaGlcNAc beta 1,4- galactosyltransferase, polypeptide 3	B4GALT3	Homo sapiens
8702	UDP-Gal:betaGlcNAc beta 1,4- galactosyltransferase, polypeptide 4	B4GALT4	Homo sapiens
25825	beta-site APP-cleaving enzyme 2	BACE2	Homo sapiens
571	BTB and CNC homology 1, basic leucine zipper transcription factor 1	BACH1	Homo sapiens
573	BCL2-associated athanogene	BAG1	Homo sapiens
9531	BCL2-associated athanogene 3	BAG3	Homo sapiens
9530	BCL2-associated athanogene 4	BAG4	Homo sapiens
9529	BCL2-associated athanogene 5	BAG5	Homo sapiens
575	brain-specific angiogenesis inhibitor 1	BAI1	Homo sapiens
576	brain-specific angiogenesis inhibitor 2	BAI2	Homo sapiens
10458	BAI1-associated protein 2	BAIAP2	Homo sapiens
600	BCL2-antagonist/killer 1; BCL2-like 7 pseudogene 1	BAK1	Homo sapiens
578	BCL2-antagonist/killer 1; BCL2-like 7 pseudogene 1	BAK1	Homo sapiens
25805	hypothetical LOC729590; BMP and activin membrane-bound inhibitor homolog (Xenopus laevis)	BAMBI	Homo sapiens
729590	hypothetical LOC729590; BMP and activin membrane-bound inhibitor homolog (Xenopus laevis)	BAMBI	Homo sapiens
8314	BRCA1 associated protein-1 (ubiquitin carboxy-terminal hydrolase)	BAP1	Homo sapiens
580	BRCA1 associated RING domain 1	BARD1	Homo sapiens
8538	BARX homeobox 2	BARX2	Homo sapiens
10409	brain abundant, membrane attached signal protein 1	BASP1	Homo sapiens
7916	HLA-B associated transcript 2	BAT2	Homo sapiens
7917	HLA-B associated transcript 3	BAT3	Homo sapiens
10538	basic leucine zipper transcription factor, ATF-like	BATF	Homo sapiens
581	BCL2-associated X protein	BAX	Homo sapiens
11177	bromodomain adjacent to zinc finger domain, 1A	BAZ1A	Homo sapiens

9031	bromodomain adjacent to zinc finger domain, 1B	BAZ1B	Homo sapiens
8424	butyrobetaine (gamma), 2-oxoglutarate dioxygenase (gamma-butyrobetaine hydroxylase) 1	BBOX1	Homo sapiens
56987	bobby sox homolog (Drosophila)	BBX	Homo sapiens
63827	brevican	BCAN	Homo sapiens
10134	B-cell receptor-associated protein 31	BCAP31	Homo sapiens
8412	breast cancer anti-estrogen resistance 3	BCAR3	Homo sapiens
8537	breast carcinoma amplified sequence 1	BCAS1	Homo sapiens
10286	breast carcinoma amplified sequence 2	BCAS2	Homo sapiens
586	branched chain aminotransferase 1, cytosolic	BCAT1	Homo sapiens
587	branched chain aminotransferase 2, mitochondrial	BCAT2	Homo sapiens
594	branched chain keto acid dehydrogenase E1, beta polypeptide	BCKDHB	Homo sapiens
8915	B-cell CLL/lymphoma 10; hypothetical LOC646626	BCL10	Homo sapiens
646626	B-cell CLL/lymphoma 10; hypothetical LOC646626	BCL10	Homo sapiens
53335	B-cell CLL/lymphoma 11A (zinc finger protein)	BCL11A	Homo sapiens
596	B-cell CLL/lymphoma 2	BCL2	Homo sapiens
597	BCL2-related protein A1	BCL2A1	Homo sapiens
598	BCL2-like 1	BCL2L1	Homo sapiens
10018	BCL2-like 11 (apoptosis facilitator)	BCL2L11	Homo sapiens
599	BCL2-like 2	BCL2L2	Homo sapiens
602	B-cell CLL/lymphoma 3	BCL3	Homo sapiens
604	B-cell CLL/lymphoma 6	BCL6	Homo sapiens
9275	B-cell CLL/lymphoma 7B	BCL7B	Homo sapiens
607	B-cell CLL/lymphoma 9	BCL9	Homo sapiens
54880	BCL6 co-repressor	BCOR	Homo sapiens
617	BCS1-like (yeast)	BCS1L	Homo sapiens
623	bradykinin receptor B1	BDKRB1	Homo sapiens
624	bradykinin receptor B2	BDKRB2	Homo sapiens
627	brain-derived neurotrophic factor	BDNF	Homo sapiens
8678	beclin 1, autophagy related	BECN1	Homo sapiens
10282	hypothetical protein LOC100128542; blocked early in transport 1 homolog (S. cerevisiae)	BET1	Homo sapiens
100128542	hypothetical protein LOC100128542; blocked early in transport 1 homolog (S. cerevisiae)	BET1	Homo sapiens
51283	bifunctional apoptosis regulator	BFAR	Homo sapiens
631	beaded filament structural protein 1, filensin	BFSP1	Homo sapiens
8419	beaded filament structural protein 2, phakinin	BFSP2	Homo sapiens
11243	bone gamma-carboxyglutamate (gla) protein; polyamine-modulated factor 1	BGLAP	Homo sapiens
632	bone gamma-carboxyglutamate (gla) protein; polyamine-modulated factor 1	BGLAP	Homo sapiens
633	biglycan	BGN	Homo sapiens
635	betaine-homocysteine methyltransferase	BHMT	Homo sapiens
636	bicaudal D homolog 1 (Drosophila)	BICD1	Homo sapiens
23299	bicaudal D homolog 2 (Drosophila)	BICD2	Homo sapiens
637	BH3 interacting domain death agonist	BID	Homo sapiens
329	baculoviral IAP repeat-containing 2	BIRC2	Homo sapiens

330	baculoviral IAP repeat-containing 3	BIRC3	Homo sapiens
332	baculoviral IAP repeat-containing 5	BIRC5	Homo sapiens
10904	bladder cancer associated protein	BLCAP	Homo sapiens
640	B lymphoid tyrosine kinase	BLK	Homo sapiens
641	Bloom syndrome, RecQ helicase-like	BLM	Homo sapiens
642	bleomycin hydrolase	BLMH	Homo sapiens
29760	B-cell linker	BLNK	Homo sapiens
2647	biogenesis of lysosomal organelles complex-1, subunit 1	BLOC1S1	Homo sapiens
645	biliverdin reductase B (flavin reductase (NADPH))	BLVRB	Homo sapiens
8548	basic leucine zipper nuclear factor 1	BLZF1	Homo sapiens
649	bone morphogenetic protein 1	BMP1	Homo sapiens
650	bone morphogenetic protein 2	BMP2	Homo sapiens
55589	BMP2 inducible kinase	BMP2K	Homo sapiens
652	bone morphogenetic protein 4	BMP4	Homo sapiens
653	bone morphogenetic protein 5	BMP5	Homo sapiens
655	bone morphogenetic protein 7	BMP7	Homo sapiens
353500	bone morphogenetic protein 8a	BMP8A	Homo sapiens
662	BCL2/adenovirus E1B 19kDa interacting protein 1	BNIP1	Homo sapiens
66037	bol, boule-like (Drosophila)	BOLL	Homo sapiens
669	2,3-bisphosphoglycerate mutase	BPGM	Homo sapiens
671	bactericidal/permeability-increasing protein	BPI	Homo sapiens
673	v-raf murine sarcoma viral oncogene homolog B1	BRAF	Homo sapiens
672	breast cancer 1, early onset	BRCA1	Homo sapiens
675	breast cancer 2, early onset	BRCA2	Homo sapiens
23774	bromodomain containing 1	BRD1	Homo sapiens
6046	bromodomain containing 2	BRD2	Homo sapiens
23476	bromodomain containing 4	BRD4	Homo sapiens
10902	bromodomain containing 8	BRD8	Homo sapiens
676	bromodomain, testis-specific; hCG1811337	BRDT	Homo sapiens
643486	bromodomain, testis-specific; hCG1811337	BRDT	Homo sapiens
9577	brain and reproductive organ-expressed (TNFRSF1A modulator)	BRE	Homo sapiens
2972	BRF1 homolog, subunit of RNA polymerase III transcription initiation factor IIIB (S. cerevisiae)	BRF1	Homo sapiens
683	bone marrow stromal cell antigen 1	BST1	Homo sapiens
684	NPC-A-7; bone marrow stromal cell antigen 2	BST2	Homo sapiens
100129681	NPC-A-7; bone marrow stromal cell antigen 2	BST2	Homo sapiens
55643	BTB (POZ) domain containing 2	BTBD2	Homo sapiens
685	betacellulin	BTC	Homo sapiens
686	biotinidase	BTD	Homo sapiens
689	basic transcription factor 3; basic transcription factor 3, like 1 pseudogene	BTF3	Homo sapiens
690	basic transcription factor 3; basic transcription factor 3, like 1 pseudogene	BTF3	Homo sapiens
694	B-cell translocation gene 1, anti-proliferative	BTG1	Homo sapiens
7832	BTG family, member 2	BTG2	Homo sapiens
54766	B-cell translocation gene 4	BTG4	Homo sapiens
695	Bruton agammaglobulinemia tyrosine kinase	BTK	Homo sapiens

11120	butyrophilin, subfamily 2, member A1	BTN2A1	Homo sapiens
11119	butyrophilin, subfamily 3, member A1	BTN3A1	Homo sapiens
11118	butyrophilin, subfamily 3, member A2	BTN3A2	Homo sapiens
10384	butyrophilin, subfamily 3, member A3	BTN3A3	Homo sapiens
10917	butyrophilin-like 3	BTNL3	Homo sapiens
8945	beta-transducin repeat containing	BTRC	Homo sapiens
699	budding uninhibited by benzimidazoles 1 homolog (yeast)	BUB1	Homo sapiens
701	budding uninhibited by benzimidazoles 1 homolog beta (yeast)	BUB1B	Homo sapiens
9184	budding uninhibited by benzimidazoles 3 homolog (yeast)	BUB3	Homo sapiens
705	bystin-like	BYSL	Homo sapiens
11067	chromosome 10 open reading frame 10	C10orf10	Homo sapiens
10974	chromosome 10 open reading frame 116	C10orf116	Homo sapiens
26148	chromosome 10 open reading frame 12	C10orf12	Homo sapiens
196740	chromosome 10 open reading frame 72	C10orf72	Homo sapiens
9556	chromosome 14 open reading frame 2	C14orf2	Homo sapiens
122616	chromosome 14 open reading frame 79	C14orf79	Homo sapiens
753	chromosome 18 open reading frame 1	C18orf1	Homo sapiens
25941	chromosome 18 open reading frame 10	C18orf10	Homo sapiens
91304	chromosome 19 open reading frame 6	C19orf6	Homo sapiens
9473	chromosome 1 open reading frame 38	C1orf38	Homo sapiens
51430	chromosome 1 open reading frame 9	C1orf9	Homo sapiens
10882	complement component 1, q subcomponent-like 1	C1QL1	Homo sapiens
114898	C1q and tumor necrosis factor related protein 2	C1QTNF2	Homo sapiens
715	complement component 1, r subcomponent	C1R	Homo sapiens
717	complement component 2	C2	Homo sapiens
51526	chromosome 20 open reading frame 111	C20orf111	Homo sapiens
718	similar to Complement C3 precursor; complement component 3; hypothetical protein LOC100133511	C3	Homo sapiens
100133511	similar to Complement C3 precursor; complement component 3; hypothetical protein LOC100133511	C3	Homo sapiens
653879	similar to Complement C3 precursor; complement component 3; hypothetical protein LOC100133511	C3	Homo sapiens
719	complement component 3a receptor 1	C3AR1	Homo sapiens
722	complement component 4 binding protein, alpha	C4BPA	Homo sapiens
725	complement component 4 binding protein, beta	C4BPB	Homo sapiens
727	complement component 5	C5	Homo sapiens
9315	chromosome 5 open reading frame 13	C5orf13	Homo sapiens
56951	chromosome 5 open reading frame 15	C5orf15	Homo sapiens
10826	chromosome 5 open reading frame 4	C5orf4	Homo sapiens
729	complement component 6	C6	Homo sapiens
10665	chromosome 6 open reading frame 10	C6orf10	Homo sapiens
10591	chromosome 6 open reading frame 108	C6orf108	Homo sapiens
221443	chromosome 6 open reading frame 130	C6orf130	Homo sapiens
57150	chromosome 6 open reading frame 162	C6orf162	Homo sapiens
732	complement component 8, beta polypeptide	C8B	Homo sapiens
51490	chromosome 9 open reading frame 114	C9orf114	Homo sapiens
84909	chromosome 9 open reading frame 3	C9orf3	Homo sapiens

770	carbonic anhydrase XI	CA11	Homo sapiens
771	carbonic anhydrase XII	CA12	Homo sapiens
760	carbonic anhydrase II	CA2	Homo sapiens
768	carbonic anhydrase IX	CA9	Homo sapiens
81617	calcium binding protein 39-like	CAB39L	Homo sapiens
9478	calcium binding protein 1	CABP1	Homo sapiens
773	calcium channel, voltage-dependent, P/Q type, alpha 1A subunit	CACNA1A	Homo sapiens
776	calcium channel, voltage-dependent, L type, alpha 1D subunit	CACNA1D	Homo sapiens
777	calcium channel, voltage-dependent, R type, alpha 1E subunit	CACNA1E	Homo sapiens
8912	calcium channel, voltage-dependent, T type, alpha 1H subunit	CACNA1H	Homo sapiens
781	calcium channel, voltage-dependent, alpha 2/delta subunit 1	CACNA2D1	Homo sapiens
9254	calcium channel, voltage-dependent, alpha 2/delta subunit 2	CACNA2D2	Homo sapiens
784	calcium channel, voltage-dependent, beta 3 subunit	CACNB3	Homo sapiens
785	calcium channel, voltage-dependent, beta 4 subunit	CACNB4	Homo sapiens
790	carbamoyl-phosphate synthetase 2, aspartate transcarbamylase, and dihydroorotase	CAD	Homo sapiens
8618	Ca++-dependent secretion activator	CADPS	Homo sapiens
793	calbindin 1, 28kDa	CALB1	Homo sapiens
10203	calcitonin receptor-like	CALCRL	Homo sapiens
800	caldesmon 1	CALD1	Homo sapiens
811	calreticulin	CALR	Homo sapiens
813	calumenin	CALU	Homo sapiens
8536	calcium/calmodulin-dependent protein kinase I	CAMK1	Homo sapiens
815	calcium/calmodulin-dependent protein kinase II alpha	CAMK2A	Homo sapiens
816	calcium/calmodulin-dependent protein kinase II beta	CAMK2B	Homo sapiens
818	calcium/calmodulin-dependent protein kinase II gamma	CAMK2G	Homo sapiens
814	calcium/calmodulin-dependent protein kinase IV	CAMK4	Homo sapiens
10645	calcium/calmodulin-dependent protein kinase kinase 2, beta	CAMKK2	Homo sapiens
819	calcium modulating ligand	CAMLG	Homo sapiens
820	cathelicidin antimicrobial peptide	CAMP	Homo sapiens
23125	calmodulin binding transcription activator 2	CAMTA2	Homo sapiens
821	calnexin	CANX	Homo sapiens
10487	CAP, adenylate cyclase-associated protein 1 (yeast)	CAP1	Homo sapiens
10486	CAP, adenylate cyclase-associated protein, 2 (yeast)	CAP2	Homo sapiens
822	capping protein (actin filament), gelsolin-like	CAPG	Homo sapiens
823	calpain 1, (mu/I) large subunit	CAPN1	Homo sapiens
11132	calpain 10	CAPN10	Homo sapiens
824	calpain 2, (m/II) large subunit	CAPN2	Homo sapiens
726	calpain 5	CAPN5	Homo sapiens
827	calpain 6	CAPN6	Homo sapiens
23473	calpain 7	CAPN7	Homo sapiens
10753	calpain 9	CAPN9	Homo sapiens
826	calpain, small subunit 1	CAPNS1	Homo sapiens
829	capping protein (actin filament) muscle Z-line, alpha 1	CAPZA1	Homo sapiens
833	cysteinyl-tRNA synthetase	CARS	Homo sapiens
22794	cancer susceptibility candidate 3	CASC3	Homo sapiens

8573	calcium/calmodulin-dependent serine protein kinase (MAGUK family)	CASK	Homo sapiens
834	caspase 1, apoptosis-related cysteine peptidase (interleukin 1, beta, convertase)	CASP1	Homo sapiens
843	caspase 10, apoptosis-related cysteine peptidase	CASP10	Homo sapiens
835	caspase 2, apoptosis-related cysteine peptidase	CASP2	Homo sapiens
836	caspase 3, apoptosis-related cysteine peptidase	CASP3	Homo sapiens
840	caspase 7, apoptosis-related cysteine peptidase	CASP7	Homo sapiens
841	caspase 8, apoptosis-related cysteine peptidase	CASP8	Homo sapiens
842	caspase 9, apoptosis-related cysteine peptidase	CASP9	Homo sapiens
845	calsequestrin 2 (cardiac muscle)	CASQ2	Homo sapiens
846	calcium-sensing receptor	CASR	Homo sapiens
831	calpastatin	CAST	Homo sapiens
847	catalase	CAT	Homo sapiens
857	caveolin 1, caveolae protein, 22kDa	CAV1	Homo sapiens
858	caveolin 2	CAV2	Homo sapiens
859	caveolin 3	CAV3	Homo sapiens
10367	calcium binding atopy-related autoantigen 1	CBARA1	Homo sapiens
9139	core-binding factor, runt domain, alpha subunit 2; translocated to, 2	CBFA2T2	Homo sapiens
865	core-binding factor, beta subunit	CBFB	Homo sapiens
867	Cas-Br-M (murine) ecotropic retroviral transforming sequence	CBL	Homo sapiens
868	Cas-Br-M (murine) ecotropic retroviral transforming sequence b	CBLB	Homo sapiens
869	cerebellin 1 precursor	CBLN1	Homo sapiens
873	carbonyl reductase 1	CBR1	Homo sapiens
874	carbonyl reductase 3	CBR3	Homo sapiens
875	cystathionine-beta-synthase	CBS	Homo sapiens
10951	chromobox homolog 1 (HP1 beta homolog Drosophila)	CBX1	Homo sapiens
644101	similar to chromobox homolog 3; chromobox homolog 3 (HP1 gamma homolog, Drosophila)	CBX3	Homo sapiens
11335	similar to chromobox homolog 3; chromobox homolog 3 (HP1 gamma homolog, Drosophila)	CBX3	Homo sapiens
8535	chromobox homolog 4 (Pc class homolog, Drosophila)	CBX4	Homo sapiens
23468	chromobox homolog 5 (HP1 alpha homolog, Drosophila)	CBX5	Homo sapiens
883	cysteine conjugate-beta lyase, cytoplasmic	CCBL1	Homo sapiens
8030	coiled-coil domain containing 6	CCDC6	Homo sapiens
881	calicin	CCIN	Homo sapiens
887	cholecystokinin B receptor	CCKBR	Homo sapiens
6346	chemokine (C-C motif) ligand 1	CCL1	Homo sapiens
6356	chemokine (C-C motif) ligand 11	CCL11	Homo sapiens
6357	chemokine (C-C motif) ligand 13	CCL13	Homo sapiens
6360	chemokine (C-C motif) ligand 16	CCL16	Homo sapiens
6362	chemokine (C-C motif) ligand 18 (pulmonary and activation-regulated)	CCL18	Homo sapiens
6363	chemokine (C-C motif) ligand 19	CCL19	Homo sapiens
6347	chemokine (C-C motif) ligand 2	CCL2	Homo sapiens
6364	chemokine (C-C motif) ligand 20	CCL20	Homo sapiens
6368	chemokine (C-C motif) ligand 23	CCL23	Homo sapiens
6369	chemokine (C-C motif) ligand 24	CCL24	Homo sapiens

6370	chemokine (C-C motif) ligand 25	CCL25	Homo sapiens
10850	chemokine (C-C motif) ligand 27	CCL27	Homo sapiens
56477	chemokine (C-C motif) ligand 28	CCL28	Homo sapiens
6352	chemokine (C-C motif) ligand 5	CCL5	Homo sapiens
6354	chemokine (C-C motif) ligand 7	CCL7	Homo sapiens
6355	chemokine (C-C motif) ligand 8	CCL8	Homo sapiens
8900	cyclin A1	CCNA1	Homo sapiens
890	cyclin A2	CCNA2	Homo sapiens
891	cyclin B1	CCNB1	Homo sapiens
9133	cyclin B2	CCNB2	Homo sapiens
595	cyclin D1	CCND1	Homo sapiens
894	cyclin D2	CCND2	Homo sapiens
896	cyclin D3	CCND3	Homo sapiens
898	cyclin E1	CCNE1	Homo sapiens
899	cyclin F	CCNF	Homo sapiens
900	cyclin G1	CCNG1	Homo sapiens
902	cyclin H	CCNH	Homo sapiens
10983	cyclin I	CCNI	Homo sapiens
904	cyclin T1	CCNT1	Homo sapiens
1230	chemokine (C-C motif) receptor 1	CCR1	Homo sapiens
729230	chemokine (C-C motif) receptor 2	CCR2	Homo sapiens
1234	chemokine (C-C motif) receptor 5	CCR5	Homo sapiens
81669	cyclin L2; chemokine (C-C motif) receptor 6	CCR6	Homo sapiens
1235	cyclin L2; chemokine (C-C motif) receptor 6	CCR6	Homo sapiens
51554	chemokine (C-C motif) receptor-like 1	CCRL1	Homo sapiens
9034	chemokine (C-C motif) receptor-like 2	CCRL2	Homo sapiens
10576	chaperonin containing TCP1, subunit 2 (beta)	CCT2	Homo sapiens
10575	chaperonin containing TCP1, subunit 4 (delta)	CCT4	Homo sapiens
22948	chaperonin containing TCP1, subunit 5 (epsilon)	CCT5	Homo sapiens
908	chaperonin containing TCP1, subunit 6A (zeta 1)	CCT6A	Homo sapiens
10574	chaperonin containing TCP1, subunit 7 (eta)	CCT7	Homo sapiens
929	CD14 molecule	CD14	Homo sapiens
8763	CD164 molecule, sialomucin	CD164	Homo sapiens
930	CD19 molecule	CD19	Homo sapiens
909	CD1a molecule	CD1A	Homo sapiens
910	CD1b molecule	CD1B	Homo sapiens
911	CD1c molecule	CD1C	Homo sapiens
912	CD1d molecule	CD1D	Homo sapiens
914	CD2 molecule	CD2	Homo sapiens
4345	CD200 molecule	CD200	Homo sapiens
30835	CD209 molecule	CD209	Homo sapiens
933	CD22 molecule	CD22	Homo sapiens
10666	CD226 molecule	CD226	Homo sapiens
940	CD28 molecule	CD28	Homo sapiens
23607	CD2-associated protein	CD2AP	Homo sapiens
945	CD33 molecule	CD33	Homo sapiens

947	CD34 molecule	CD34	Homo sapiens
948	CD36 molecule (thrombospondin receptor)	CD36	Homo sapiens
951	CD37 molecule	CD37	Homo sapiens
952	CD38 molecule	CD38	Homo sapiens
915	CD3d molecule, delta (CD3-TCR complex)	CD3D	Homo sapiens
916	CD3e molecule, epsilon (CD3-TCR complex)	CD3E	Homo sapiens
917	CD3g molecule, gamma (CD3-TCR complex)	CD3G	Homo sapiens
920	CD4 molecule	CD4	Homo sapiens
961	CD47 molecule	CD47	Homo sapiens
962	CD48 molecule	CD48	Homo sapiens
921	CD5 molecule	CD5	Homo sapiens
963	CD53 molecule	CD53	Homo sapiens
965	CD58 molecule	CD58	Homo sapiens
966	CD59 molecule, complement regulatory protein	CD59	Homo sapiens
922	CD5 molecule-like	CD5L	Homo sapiens
923	CD6 molecule	CD6	Homo sapiens
967	CD63 molecule	CD63	Homo sapiens
974	CD79b molecule, immunoglobulin-associated beta	CD79B	Homo sapiens
941	CD80 molecule	CD80	Homo sapiens
975	CD81 molecule	CD81	Homo sapiens
9308	CD83 molecule	CD83	Homo sapiens
8832	CD84 molecule	CD84	Homo sapiens
942	CD86 molecule	CD86	Homo sapiens
925	CD8a molecule	CD8A	Homo sapiens
928	CD9 molecule	CD9	Homo sapiens
10225	CD96 molecule	CD96	Homo sapiens
976	CD97 molecule	CD97	Homo sapiens
4267	CD99 molecule	CD99	Homo sapiens
978	cytidine deaminase	CDA	Homo sapiens
8556	CDC14 cell division cycle 14 homolog A (S. cerevisiae)	CDC14A	Homo sapiens
8555	CDC14 cell division cycle 14 homolog B (S. cerevisiae)	CDC14B	Homo sapiens
8881	cell division cycle 16 homolog (S. cerevisiae)	CDC16	Homo sapiens
991	cell division cycle 20 homolog (S. cerevisiae)	CDC20	Homo sapiens
8697	cell division cycle 23 homolog (S. cerevisiae)	CDC23	Homo sapiens
993	cell division cycle 25 homolog A (S. pombe)	CDC25A	Homo sapiens
995	cell division cycle 25 homolog C (S. pombe)	CDC25C	Homo sapiens
996	cell division cycle 27 homolog (S. cerevisiae)	CDC27	Homo sapiens
997	cell division cycle 34 homolog (S. cerevisiae)	CDC34	Homo sapiens
11140	cell division cycle 37 homolog (S. cerevisiae)	CDC37	Homo sapiens
51362	cell division cycle 40 homolog (S. cerevisiae)	CDC40	Homo sapiens
8476	CDC42 binding protein kinase alpha (DMPK-like)	CDC42BPA	Homo sapiens
10602	CDC42 effector protein (Rho GTPase binding) 3	CDC42EP3	Homo sapiens
988	CDC5 cell division cycle 5-like (S. pombe)	CDC5L	Homo sapiens
990	cell division cycle 6 homolog (S. cerevisiae)	CDC6	Homo sapiens
8317	cell division cycle 7 homolog (S. cerevisiae)	CDC7	Homo sapiens
999	cadherin 1, type 1, E-cadherin (epithelial)	CDH1	Homo sapiens

1009	cadherin 11, type 2, OB-cadherin (osteoblast)	CDH11	Homo sapiens
1012	cadherin 13, H-cadherin (heart)	CDH13	Homo sapiens
1013	cadherin 15, type 1, M-cadherin (myotubule)	CDH15	Homo sapiens
1014	cadherin 16, KSP-cadherin	CDH16	Homo sapiens
1015	cadherin 17, LI cadherin (liver-intestine)	CDH17	Homo sapiens
28513	cadherin 19, type 2	CDH19	Homo sapiens
1000	cadherin 2, type 1, N-cadherin (neuronal)	CDH2	Homo sapiens
64405	cadherin-like 22	CDH22	Homo sapiens
60437	cadherin-like 26	CDH26	Homo sapiens
1001	cadherin 3, type 1, P-cadherin (placental)	CDH3	Homo sapiens
1002	cadherin 4, type 1, R-cadherin (retinal)	CDH4	Homo sapiens
1003	cadherin 5, type 2 (vascular endothelium)	CDH5	Homo sapiens
1004	cadherin 6, type 2, K-cadherin (fetal kidney)	CDH6	Homo sapiens
1006	cadherin 8, type 2	CDH8	Homo sapiens
10423	CDP-diacylglycerol--inositol 3-phosphatidyltransferase (phosphatidylinositol synthase)	CDIPT	Homo sapiens
8558	cyclin-dependent kinase 10	CDK10	Homo sapiens
1017	cyclin-dependent kinase 2	CDK2	Homo sapiens
1018	cyclin-dependent kinase 3	CDK3	Homo sapiens
1019	cyclin-dependent kinase 4	CDK4	Homo sapiens
1020	cyclin-dependent kinase 5	CDK5	Homo sapiens
8851	cyclin-dependent kinase 5, regulatory subunit 1 (p35)	CDK5R1	Homo sapiens
8941	cyclin-dependent kinase 5, regulatory subunit 2 (p39)	CDK5R2	Homo sapiens
1021	cyclin-dependent kinase 6	CDK6	Homo sapiens
1022	cyclin-dependent kinase 7	CDK7	Homo sapiens
1025	cyclin-dependent kinase 9	CDK9	Homo sapiens
6792	cyclin-dependent kinase-like 5	CDKL5	Homo sapiens
1026	cyclin-dependent kinase inhibitor 1A (p21, Cip1)	CDKN1A	Homo sapiens
1027	cyclin-dependent kinase inhibitor 1B (p27, Kip1)	CDKN1B	Homo sapiens
1028	cyclin-dependent kinase inhibitor 1C (p57, Kip2)	CDKN1C	Homo sapiens
1029	cyclin-dependent kinase inhibitor 2A (melanoma, p16, inhibits CDK4)	CDKN2A	Homo sapiens
1030	cyclin-dependent kinase inhibitor 2B (p15, inhibits CDK4)	CDKN2B	Homo sapiens
1031	cyclin-dependent kinase inhibitor 2C (p18, inhibits CDK4)	CDKN2C	Homo sapiens
1032	cyclin-dependent kinase inhibitor 2D (p19, inhibits CDK4)	CDKN2D	Homo sapiens
1033	cyclin-dependent kinase inhibitor 3	CDKN3	Homo sapiens
1036	cysteine dioxygenase, type I	CDO1	Homo sapiens
1039	cerebellar degeneration-related protein 2, 62kDa	CDR2	Homo sapiens
1040	CDP-diacylglycerol synthase (phosphatidate cytidylyltransferase) 1	CDS1	Homo sapiens
8760	CDP-diacylglycerol synthase (phosphatidate cytidylyltransferase) 2	CDS2	Homo sapiens
1044	caudal type homeobox 1	CDX1	Homo sapiens
1045	caudal type homeobox 2	CDX2	Homo sapiens
9425	chromodomain protein, Y-like	CDYL	Homo sapiens
634	carcinoembryonic antigen-related cell adhesion molecule 1 (biliary glycoprotein)	CEACAM1	Homo sapiens
1089	carcinoembryonic antigen-related cell adhesion molecule 4	CEACAM4	Homo sapiens
1087	carcinoembryonic antigen-related cell adhesion molecule 7	CEACAM7	Homo sapiens

1051	CCAAT/enhancer binding protein (C/EBP), beta	CEBPB	Homo sapiens
1054	CCAAT/enhancer binding protein (C/EBP), gamma	CEBPG	Homo sapiens
51816	cat eye syndrome chromosome region, candidate 1	CECR1	Homo sapiens
9620	cadherin, EGF LAG seven-pass G-type receptor 1 (flamingo homolog, Drosophila)	CELSR1	Homo sapiens
1952	cadherin, EGF LAG seven-pass G-type receptor 2 (flamingo homolog, Drosophila)	CELSR2	Homo sapiens
1062	centromere protein E, 312kDa	CENPE	Homo sapiens
1063	centromere protein F, 350/400ka (mitosin)	CENPF	Homo sapiens
9350	cerberus 1, cysteine knot superfamily, homolog (Xenopus laevis)	CER1	Homo sapiens
64781	ceramide kinase	CERK	Homo sapiens
8824	carboxylesterase 2 (intestine, liver)	CES2	Homo sapiens
1068	centrin, EF-hand protein, 1	CETN1	Homo sapiens
1070	centrin, EF-hand protein, 3 (CDC31 homolog, yeast)	CETN3	Homo sapiens
10428	craniofacial development protein 1	CFDP1	Homo sapiens
1072	cofilin 1 (non-muscle)	CFL1	Homo sapiens
8837	CASP8 and FADD-like apoptosis regulator	CFLAR	Homo sapiens
1081	glycoprotein hormones, alpha polypeptide	CGA	Homo sapiens
10668	cell growth regulator with ring finger domain 1	CGRRF1	Homo sapiens
9023	cholesterol 25-hydroxylase	CH25H	Homo sapiens
1101	chondroadherin	CHAD	Homo sapiens
8208	chromatin assembly factor 1, subunit B (p60)	CHAF1B	Homo sapiens
1105	chromodomain helicase DNA binding protein 1	CHD1	Homo sapiens
9557	chromodomain helicase DNA binding protein 1-like	CHD1L	Homo sapiens
1106	chromodomain helicase DNA binding protein 2	CHD2	Homo sapiens
1108	chromodomain helicase DNA binding protein 4	CHD4	Homo sapiens
26038	chromodomain helicase DNA binding protein 5	CHD5	Homo sapiens
80205	chromodomain helicase DNA binding protein 9	CHD9	Homo sapiens
1111	CHK1 checkpoint homolog (S. pombe)	CHEK1	Homo sapiens
11200	protein kinase CHK2-like; CHK2 checkpoint homolog (S. pombe); similar to hCG1983233	CHEK2	Homo sapiens
646096	protein kinase CHK2-like; CHK2 checkpoint homolog (S. pombe); similar to hCG1983233	CHEK2	Homo sapiens
100133012	protein kinase CHK2-like; CHK2 checkpoint homolog (S. pombe); similar to hCG1983233	CHEK2	Homo sapiens
1113	chromogranin A (parathyroid secretory protein 1)	CHGA	Homo sapiens
1114	chromogranin B (secretogranin 1)	CHGB	Homo sapiens
1116	chitinase 3-like 1 (cartilage glycoprotein-39)	CHI3L1	Homo sapiens
1117	chitinase 3-like 2	CHI3L2	Homo sapiens
10752	cell adhesion molecule with homology to L1CAM (close homolog of L1)	CHL1	Homo sapiens
1122	choroideremia-like (Rab escort protein 2)	CHML	Homo sapiens
1123	chimerin (chimaerin) 1	CHN1	Homo sapiens
56994	choline phosphotransferase 1	CHPT1	Homo sapiens
8646	chordin	CHRD	Homo sapiens
1136	cholinergic receptor, nicotinic, alpha 3	CHRNA3	Homo sapiens
8534	carbohydrate (keratan sulfate Gal-6) sulfotransferase 1	CHST1	Homo sapiens

9486	carbohydrate sulfotransferase 10	CHST10	Homo sapiens
50515	carbohydrate (chondroitin 4) sulfotransferase 11	CHST11	Homo sapiens
9435	carbohydrate (N-acetylglucosamine-6-O) sulfotransferase 2	CHST2	Homo sapiens
728599	cytokine induced apoptosis inhibitor 1; cytokine induced apoptosis inhibitor 1 pseudogene	CIAPIN1	Homo sapiens
57019	cytokine induced apoptosis inhibitor 1; cytokine induced apoptosis inhibitor 1 pseudogene	CIAPIN1	Homo sapiens
10518	calcium and integrin binding family member 2	CIB2	Homo sapiens
1149	cell death-inducing DFFA-like effector a	CIDEA	Homo sapiens
63924	cell death-inducing DFFA-like effector c	CIDEC	Homo sapiens
8483	cartilage intermediate layer protein, nucleotide pyrophosphohydrolase	CILP	Homo sapiens
1153	cold inducible RNA binding protein	CIRBP	Homo sapiens
11113	citron (rho-interacting, serine/threonine kinase 21)	CIT	Homo sapiens
4435	Cbp/p300-interacting transactivator, with Glu/Asp-rich carboxy-terminal domain, 1	CITED1	Homo sapiens
10370	Cbp/p300-interacting transactivator, with Glu/Asp-rich carboxy-terminal domain, 2	CITED2	Homo sapiens
25792	CDKN1A interacting zinc finger protein 1	CIZ1	Homo sapiens
51192	chemokine-like factor	CKLF	Homo sapiens
1160	creatine kinase, mitochondrial 2 (sarcomeric)	CKMT2	Homo sapiens
23332	cytoplasmic linker associated protein 1	CLASP1	Homo sapiens
23122	cytoplasmic linker associated protein 2	CLASP2	Homo sapiens
9635	chloride channel accessory 2	CLCA2	Homo sapiens
1181	chloride channel 2	CLCN2	Homo sapiens
1182	chloride channel 3	CLCN3	Homo sapiens
1183	chloride channel 4	CLCN4	Homo sapiens
1184	chloride channel 5	CLCN5	Homo sapiens
1185	chloride channel 6	CLCN6	Homo sapiens
1186	chloride channel 7	CLCN7	Homo sapiens
9076	claudin 1	CLDN1	Homo sapiens
9071	claudin 10	CLDN10	Homo sapiens
9069	claudin 12	CLDN12	Homo sapiens
51208	claudin 18	CLDN18	Homo sapiens
9075	claudin 2	CLDN2	Homo sapiens
1364	claudin 4	CLDN4	Homo sapiens
7122	claudin 5	CLDN5	Homo sapiens
1366	claudin 7	CLDN7	Homo sapiens
9080	claudin 9	CLDN9	Homo sapiens
1192	chloride intracellular channel 1	CLIC1	Homo sapiens
1193	chloride intracellular channel 2	CLIC2	Homo sapiens
25932	chloride intracellular channel 4	CLIC4	Homo sapiens
1196	CDC-like kinase 2	CLK2	Homo sapiens
1198	CDC-like kinase 3	CLK3	Homo sapiens
1203	ceroid-lipofuscinosis, neuronal 5	CLN5	Homo sapiens
1207	chloride channel, nucleotide-sensitive, 1A	CLNS1A	Homo sapiens
9575	clock homolog (mouse)	CLOCK	Homo sapiens

8192	ClpP caseinolytic peptidase, ATP-dependent, proteolytic subunit homolog (E. coli)	CLPP	Homo sapiens
1208	colipase, pancreatic	CLPS	Homo sapiens
10845	ClpX caseinolytic peptidase X homolog (E. coli)	CLPX	Homo sapiens
22883	calsyntenin 1	CLSTN1	Homo sapiens
1211	clathrin, light chain (Lca)	CLTA	Homo sapiens
1212	clathrin, light chain (Lcb)	CLTB	Homo sapiens
1191	clusterin	CLU	Homo sapiens
55907	cytidine monophosphate N-acetylneuraminic acid synthetase	CMAS	Homo sapiens
1258	cyclic nucleotide gated channel beta 1	CNGB1	Homo sapiens
10175	cornichon homolog (Drosophila)	CNIH	Homo sapiens
10256	connector enhancer of kinase suppressor of Ras 1	CNKSR1	Homo sapiens
1264	calponin 1, basic, smooth muscle	CNN1	Homo sapiens
1266	calponin 3, acidic	CNN3	Homo sapiens
54805	cyclin M2	CNNM2	Homo sapiens
4848	CCR4-NOT transcription complex, subunit 2	CNOT2	Homo sapiens
4849	CCR4-NOT transcription complex, subunit 3	CNOT3	Homo sapiens
29883	CCR4-NOT transcription complex, subunit 7	CNOT7	Homo sapiens
9337	CCR4-NOT transcription complex, subunit 8	CNOT8	Homo sapiens
1267	2',3'-cyclic nucleotide 3' phosphodiesterase	CNP	Homo sapiens
1271	ciliary neurotrophic factor receptor	CNTFR	Homo sapiens
1272	contactin 1	CNTN1	Homo sapiens
6900	contactin 2 (axonal)	CNTN2	Homo sapiens
27255	contactin 6	CNTN6	Homo sapiens
80347	Coenzyme A synthase	COASY	Homo sapiens
23242	cordon-bleu homolog (mouse)	COBL	Homo sapiens
22837	COBL-like 1	COBLL1	Homo sapiens
1690	coagulation factor C homolog, cochlin (Limulus polyphemus)	COCH	Homo sapiens
22796	component of oligomeric golgi complex 2	COG2	Homo sapiens
10466	component of oligomeric golgi complex 5	COG5	Homo sapiens
1300	collagen, type X, alpha 1	COL10A1	Homo sapiens
1302	collagen, type XI, alpha 2	COL11A2	Homo sapiens
1303	collagen, type XII, alpha 1	COL12A1	Homo sapiens
7373	collagen, type XIV, alpha 1	COL14A1	Homo sapiens
1308	collagen, type XVII, alpha 1	COL17A1	Homo sapiens
80781	collagen, type XVIII, alpha 1	COL18A1	Homo sapiens
1278	collagen, type I, alpha 2	COL1A2	Homo sapiens
81578	collagen, type XXI, alpha 1	COL21A1	Homo sapiens
1281	collagen, type III, alpha 1	COL3A1	Homo sapiens
1282	collagen, type IV, alpha 1	COL4A1	Homo sapiens
1287	collagen, type IV, alpha 5	COL4A5	Homo sapiens
1288	collagen, type IV, alpha 6	COL4A6	Homo sapiens
1289	collagen, type V, alpha 1	COL5A1	Homo sapiens
1290	collagen, type V, alpha 2	COL5A2	Homo sapiens
50509	collagen, type V, alpha 3	COL5A3	Homo sapiens
1291	collagen, type VI, alpha 1	COL6A1	Homo sapiens

1292	collagen, type VI, alpha 2	COL6A2	Homo sapiens
1293	collagen, type VI, alpha 3	COL6A3	Homo sapiens
1294	collagen, type VII, alpha 1	COL7A1	Homo sapiens
1295	collagen, type VIII, alpha 1	COL8A1	Homo sapiens
1297	collagen, type IX, alpha 1	COL9A1	Homo sapiens
1298	collagen, type IX, alpha 2	COL9A2	Homo sapiens
1299	collagen, type IX, alpha 3	COL9A3	Homo sapiens
10584	collectin sub-family member 10 (C-type lectin)	COLEC10	Homo sapiens
150684	copper metabolism (Murr1) domain containing 1	COMMD1	Homo sapiens
1311	cartilage oligomeric matrix protein	COMP	Homo sapiens
1314	coatomer protein complex, subunit alpha	COPA	Homo sapiens
9276	coatomer protein complex, subunit beta 2 (beta prime)	COPB2	Homo sapiens
11316	coatomer protein complex, subunit epsilon	COPE	Homo sapiens
8533	COP9 constitutive photomorphogenic homolog subunit 3 (Arabidopsis)	COPS3	Homo sapiens
10987	COP9 constitutive photomorphogenic homolog subunit 5 (Arabidopsis)	COPS5	Homo sapiens
10980	COP9 constitutive photomorphogenic homolog subunit 6 (Arabidopsis)	COPS6	Homo sapiens
50813	COP9 constitutive photomorphogenic homolog subunit 7A (Arabidopsis)	COPS7A	Homo sapiens
10920	COP9 constitutive photomorphogenic homolog subunit 8 (Arabidopsis)	COPS8	Homo sapiens
10229	coenzyme Q7 homolog, ubiquinone (yeast)	COQ7	Homo sapiens
10699	corin, serine peptidase	CORIN	Homo sapiens
11151	coronin, actin binding protein, 1A	CORO1A	Homo sapiens
7464	coronin, actin binding protein, 2A	CORO2A	Homo sapiens
378708	cortistatin; apoptosis-inducing, TAF9-like domain 1	CORT	Homo sapiens
1325	cortistatin; apoptosis-inducing, TAF9-like domain 1	CORT	Homo sapiens
1353	COX11 homolog, cytochrome c oxidase assembly protein (yeast)	COX11	Homo sapiens
10063	COX17 cytochrome c oxidase assembly homolog (S. cerevisiae)	COX17	Homo sapiens
84701	cytochrome c oxidase subunit IV isoform 2 (lung)	COX4I2	Homo sapiens
9377	cytochrome c oxidase subunit Va	COX5A	Homo sapiens
1329	cytochrome c oxidase subunit Vb	COX5B	Homo sapiens
1337	cytochrome c oxidase subunit VIa polypeptide 1	COX6A1	Homo sapiens
1339	cytochrome c oxidase subunit VIa polypeptide 2	COX6A2	Homo sapiens
1340	cytochrome c oxidase subunit Vib polypeptide 1 (ubiquitous)	COX6B1	Homo sapiens
1345	cytochrome c oxidase subunit VIc	COX6C	Homo sapiens
1346	cytochrome c oxidase subunit VIIa polypeptide 1 (muscle)	COX7A1	Homo sapiens
1347	cytochrome c oxidase subunit VIIa polypeptide 2 (liver)	COX7A2	Homo sapiens
9167	cytochrome c oxidase subunit VIIa polypeptide 2 like	COX7A2L	Homo sapiens
1350	cytochrome c oxidase subunit VIIc	COX7C	Homo sapiens
1356	ceruloplasmin (ferroxidase)	CP	Homo sapiens
1359	carboxypeptidase A3 (mast cell)	CPA3	Homo sapiens
1360	carboxypeptidase B1 (tissue)	CPB1	Homo sapiens
1361	carboxypeptidase B2 (plasma)	CPB2	Homo sapiens
1362	carboxypeptidase D	CPD	Homo sapiens

1363	carboxypeptidase E	CPE	Homo sapiens
1368	carboxypeptidase M	CPM	Homo sapiens
1369	carboxypeptidase N, polypeptide 1	CPN1	Homo sapiens
8895	copine III	CPNE3	Homo sapiens
1371	coproporphyrinogen oxidase	CPOX	Homo sapiens
1373	carbamoyl-phosphate synthetase 1, mitochondrial	CPS1	Homo sapiens
29894	cleavage and polyadenylation specific factor 1, 160kDa	CPSF1	Homo sapiens
10898	cleavage and polyadenylation specific factor 4, 30kDa	CPSF4	Homo sapiens
11052	cleavage and polyadenylation specific factor 6, 68kDa	CPSF6	Homo sapiens
1374	carnitine palmitoyltransferase 1A (liver)	CPT1A	Homo sapiens
1376	carnitine palmitoyltransferase 2	CPT2	Homo sapiens
54504	carboxypeptidase, vitellogenic-like	CPVL	Homo sapiens
8532	carboxypeptidase Z	CPZ	Homo sapiens
1380	complement component (3d/Epstein Barr virus) receptor 2	CR2	Homo sapiens
1382	cellular retinoic acid binding protein 2	CRABP2	Homo sapiens
8738	CASP2 and RIPK1 domain containing adaptor with death domain	CRADD	Homo sapiens
1384	carnitine acetyltransferase	CRAT	Homo sapiens
1385	cAMP responsive element binding protein 1	CREB1	Homo sapiens
9586	cAMP responsive element binding protein 5	CREB5	Homo sapiens
1387	CREB binding protein	CREBBP	Homo sapiens
1389	cAMP responsive element binding protein-like 2	CREBL2	Homo sapiens
78987	cysteine-rich with EGF-like domains 1	CRELD1	Homo sapiens
1390	cAMP responsive element modulator	CREM	Homo sapiens
1392	corticotropin releasing hormone	CRH	Homo sapiens
1393	corticotropin releasing hormone binding protein	CRHBP	Homo sapiens
1397	cysteine-rich protein 2	CRIP2	Homo sapiens
167	cysteine-rich secretory protein 1	CRISP1	Homo sapiens
10321	cysteine-rich secretory protein 3	CRISP3	Homo sapiens
9244	cytokine receptor-like factor 1	CRLF1	Homo sapiens
51379	cytokine receptor-like factor 3	CRLF3	Homo sapiens
1400	collapsin response mediator protein 1	CRMP1	Homo sapiens
1407	cryptochrome 1 (photolyase-like)	CRY1	Homo sapiens
1408	cryptochrome 2 (photolyase-like)	CRY2	Homo sapiens
1409	crystallin, alpha A	CRYAA	Homo sapiens
1410	crystallin, alpha B	CRYAB	Homo sapiens
1411	crystallin, beta A1	CRYBA1	Homo sapiens
1414	crystallin, beta B1	CRYBB1	Homo sapiens
1418	crystallin, gamma A	CRYGA	Homo sapiens
1420	crystallin, gamma C	CRYGC	Homo sapiens
51084	crystallin, lambda 1	CRYL1	Homo sapiens
1428	crystallin, mu	CRYM	Homo sapiens
1429	crystallin, zeta (quinone reductase)	CRYZ	Homo sapiens
1431	citrate synthase	CS	Homo sapiens
8531	cold shock domain protein A; cold shock domain protein A pseudogene 1	CSDA	Homo sapiens
440359	cold shock domain protein A; cold shock domain protein A	CSDA	Homo sapiens

	pseudogene 1		
1434	CSE1 chromosome segregation 1-like (yeast)	CSE1L	Homo sapiens
1436	colony stimulating factor 1 receptor	CSF1R	Homo sapiens
1437	colony stimulating factor 2 (granulocyte-macrophage)	CSF2	Homo sapiens
1440	colony stimulating factor 3 (granulocyte)	CSF3	Homo sapiens
1441	colony stimulating factor 3 receptor (granulocyte)	CSF3R	Homo sapiens
1452	casein kinase 1, alpha 1	CSNK1A1	Homo sapiens
1453	casein kinase 1, delta	CSNK1D	Homo sapiens
1454	casein kinase 1, epsilon	CSNK1E	Homo sapiens
1455	casein kinase 1, gamma 2	CSNK1G2	Homo sapiens
1457	casein kinase 2, alpha 1 polypeptide pseudogene; casein kinase 2, alpha 1 polypeptide	CSNK2A1	Homo sapiens
283106	casein kinase 2, alpha 1 polypeptide pseudogene; casein kinase 2, alpha 1 polypeptide	CSNK2A1	Homo sapiens
1459	casein kinase 2, alpha prime polypeptide	CSNK2A2	Homo sapiens
1460	lymphocyte antigen 6 complex, locus G5B; casein kinase 2, beta polypeptide	CSNK2B	Homo sapiens
58496	lymphocyte antigen 6 complex, locus G5B; casein kinase 2, beta polypeptide	CSNK2B	Homo sapiens
1464	chondroitin sulfate proteoglycan 4	CSPG4	Homo sapiens
10675	chondroitin sulfate proteoglycan 5 (neuroglycan C)	CSPG5	Homo sapiens
1466	cysteine and glycine-rich protein 2	CSRP2	Homo sapiens
8048	cysteine and glycine-rich protein 3 (cardiac LIM protein)	CSRP3	Homo sapiens
1471	cystatin C	CST3	Homo sapiens
1474	cystatin E/M	CST6	Homo sapiens
8530	cystatin F (leukocystatin)	CST7	Homo sapiens
1475	cystatin A (stefin A)	CSTA	Homo sapiens
1477	cleavage stimulation factor, 3' pre-RNA, subunit 1, 50kDa	CSTF1	Homo sapiens
1478	cleavage stimulation factor, 3' pre-RNA, subunit 2, 64kDa	CSTF2	Homo sapiens
1479	cleavage stimulation factor, 3' pre-RNA, subunit 3, 77kDa	CSTF3	Homo sapiens
4253	CTAGE family, member 5 pseudogene; CTAGE family member; CTAGE family, member 4; CTAGE family, member 5	CTAGE5	Homo sapiens
220429	CTAGE family, member 5 pseudogene; CTAGE family member; CTAGE family, member 4; CTAGE family, member 5	CTAGE5	Homo sapiens
643854	CTAGE family, member 5 pseudogene; CTAGE family member; CTAGE family, member 4; CTAGE family, member 5	CTAGE5	Homo sapiens
100128553	CTAGE family, member 5 pseudogene; CTAGE family member; CTAGE family, member 4; CTAGE family, member 5	CTAGE5	Homo sapiens
1487	C-terminal binding protein 1	CTBP1	Homo sapiens
1488	C-terminal binding protein 2	CTBP2	Homo sapiens
1486	chitobiase, di-N-acetyl-	CTBS	Homo sapiens
9150	CTD (carboxy-terminal domain, RNA polymerase II, polypeptide A) phosphatase, subunit 1	CTDP1	Homo sapiens
1489	cardiotrophin 1	CTF1	Homo sapiens
1490	connective tissue growth factor	CTGF	Homo sapiens
1491	cystathionase (cystathionine gamma-lyase)	CTH	Homo sapiens
115908	collagen triple helix repeat containing 1	CTHRC1	Homo sapiens
1496	catenin (cadherin-associated protein), alpha 2	CTNNA2	Homo sapiens

8727	catenin (cadherin-associated protein), alpha-like 1	CTNNAL1	Homo sapiens
1499	catenin (cadherin-associated protein), beta 1, 88kDa	CTNNB1	Homo sapiens
56998	catenin, beta interacting protein 1	CTNNBIP1	Homo sapiens
56259	catenin, beta like 1	CTNNBL1	Homo sapiens
1500	catenin (cadherin-associated protein), delta 1	CTNND1	Homo sapiens
1501	catenin (cadherin-associated protein), delta 2 (neural plakophilin-related arm-repeat protein)	CTNND2	Homo sapiens
1497	cystinosis, nephropathic	CTNS	Homo sapiens
1503	CTP synthase	CTPS	Homo sapiens
1508	cathepsin B	CTSB	Homo sapiens
1075	cathepsin C	CTSC	Homo sapiens
8722	cathepsin F	CTSF	Homo sapiens
1511	cathepsin G	CTSG	Homo sapiens
1512	cathepsin H	CTSH	Homo sapiens
1513	cathepsin K	CTSK	Homo sapiens
1515	cathepsin L2	CTSL2	Homo sapiens
1519	cathepsin O	CTSO	Homo sapiens
1520	cathepsin S	CTSS	Homo sapiens
1522	cathepsin Z	CTSZ	Homo sapiens
2017	cortactin	CTTN	Homo sapiens
8029	cubilin (intrinsic factor-cobalamin receptor)	CUBN	Homo sapiens
8454	cullin 1	CUL1	Homo sapiens
8453	cullin 2	CUL2	Homo sapiens
8452	cullin 3	CUL3	Homo sapiens
8451	cullin 4A	CUL4A	Homo sapiens
8450	cullin 4B	CUL4B	Homo sapiens
8065	cullin 5	CUL5	Homo sapiens
9820	cullin 7	CUL7	Homo sapiens
6376	chemokine (C-X3-C motif) ligand 1	CX3CL1	Homo sapiens
1524	chemokine (C-X3-C motif) receptor 1	CX3CR1	Homo sapiens
1525	coxsackie virus and adenovirus receptor pseudogene 2; coxsackie virus and adenovirus receptor	CXADR	Homo sapiens
646243	coxsackie virus and adenovirus receptor pseudogene 2; coxsackie virus and adenovirus receptor	CXADR	Homo sapiens
3627	chemokine (C-X-C motif) ligand 10	CXCL10	Homo sapiens
6373	chemokine (C-X-C motif) ligand 11	CXCL11	Homo sapiens
10563	chemokine (C-X-C motif) ligand 13	CXCL13	Homo sapiens
9547	chemokine (C-X-C motif) ligand 14	CXCL14	Homo sapiens
58191	chemokine (C-X-C motif) ligand 16	CXCL16	Homo sapiens
2920	chemokine (C-X-C motif) ligand 2	CXCL2	Homo sapiens
2921	chemokine (C-X-C motif) ligand 3	CXCL3	Homo sapiens
6372	chemokine (C-X-C motif) ligand 6 (granulocyte chemotactic protein 2)	CXCL6	Homo sapiens
4283	chemokine (C-X-C motif) ligand 9	CXCL9	Homo sapiens
7852	chemokine (C-X-C motif) receptor 4	CXCR4	Homo sapiens
10663	chemokine (C-X-C motif) receptor 6	CXCR6	Homo sapiens
1534	cytochrome b-561	CYB561	Homo sapiens
1535	cytochrome b-245, alpha polypeptide	CYBA	Homo sapiens

1536	cytochrome b-245, beta polypeptide	CYBB	Homo sapiens
1537	cytochrome c-1	CYC1	Homo sapiens
54205	cytochrome c, somatic	CYCS	Homo sapiens
50626	cysteine/histidine-rich 1	CYHR1	Homo sapiens
84663	chromosome Y open reading frame 15B	CYorf15B	Homo sapiens
1583	cytochrome P450, family 11, subfamily A, polypeptide 1	CYP11A1	Homo sapiens
1584	cytochrome P450, family 11, subfamily B, polypeptide 1	CYP11B1	Homo sapiens
1585	cytochrome P450, family 11, subfamily B, polypeptide 2	CYP11B2	Homo sapiens
1586	cytochrome P450, family 17, subfamily A, polypeptide 1	CYP17A1	Homo sapiens
1588	cytochrome P450, family 19, subfamily A, polypeptide 1	CYP19A1	Homo sapiens
1591	cytochrome P450, family 24, subfamily A, polypeptide 1	CYP24A1	Homo sapiens
1592	cytochrome P450, family 26, subfamily A, polypeptide 1	CYP26A1	Homo sapiens
56603	cytochrome P450, family 26, subfamily B, polypeptide 1	CYP26B1	Homo sapiens
1593	cytochrome P450, family 27, subfamily A, polypeptide 1	CYP27A1	Homo sapiens
1594	cytochrome P450, family 27, subfamily B, polypeptide 1	CYP27B1	Homo sapiens
1555	cytochrome P450, family 2, subfamily B, polypeptide 6	CYP2B6	Homo sapiens
1558	cytochrome P450, family 2, subfamily C, polypeptide 8	CYP2C8	Homo sapiens
1571	cytochrome P450, family 2, subfamily E, polypeptide 1	CYP2E1	Homo sapiens
1573	cytochrome P450, family 2, subfamily J, polypeptide 2	CYP2J2	Homo sapiens
51302	cytochrome P450, family 39, subfamily A, polypeptide 1	CYP39A1	Homo sapiens
1576	cytochrome P450, family 3, subfamily A, polypeptide 4	CYP3A4	Homo sapiens
1577	cytochrome P450, family 3, subfamily A, polypeptide 5	CYP3A5	Homo sapiens
1551	cytochrome P450, family 3, subfamily A, polypeptide 7	CYP3A7	Homo sapiens
10858	cytochrome P450, family 46, subfamily A, polypeptide 1	CYP46A1	Homo sapiens
1579	cytochrome P450, family 4, subfamily A, polypeptide 11	CYP4A11	Homo sapiens
1580	cytochrome P450, family 4, subfamily B, polypeptide 1	CYP4B1	Homo sapiens
57834	cytochrome P450, family 4, subfamily F, polypeptide 11	CYP4F11	Homo sapiens
8529	cytochrome P450, family 4, subfamily F, polypeptide 2	CYP4F2	Homo sapiens
11283	cytochrome P450, family 4, subfamily F, polypeptide 8	CYP4F8	Homo sapiens
1595	cytochrome P450, family 51, subfamily A, polypeptide 1	CYP51A1	Homo sapiens
9420	cytochrome P450, family 7, subfamily B, polypeptide 1	CYP7B1	Homo sapiens
3491	cysteine-rich, angiogenic inducer, 61	CYR61	Homo sapiens
1601	disabled homolog 2, mitogen-responsive phosphoprotein (Drosophila)	DAB2	Homo sapiens
1605	dystroglycan 1 (dystrophin-associated glycoprotein 1)	DAG1	Homo sapiens
1611	death-associated protein	DAP	Homo sapiens
7818	death associated protein 3	DAP3	Homo sapiens
1612	death-associated protein kinase 1	DAPK1	Homo sapiens
23604	death-associated protein kinase 2	DAPK2	Homo sapiens
1613	death-associated protein kinase 3	DAPK3	Homo sapiens
1615	aspartyl-tRNA synthetase	DARS	Homo sapiens
1616	death-domain associated protein	DAXX	Homo sapiens
1618	deleted in azoospermia-like	DAZL	Homo sapiens
1620	deleted in bladder cancer 1	DBC1	Homo sapiens
1622	diazepam binding inhibitor (GABA receptor modulator, acyl-Coenzyme A binding protein)	DBI	Homo sapiens

1629	dihydrolipoamide branched chain transacylase E2	DBT	Homo sapiens
131566	discoidin, CUB and LCCL domain containing 2	DCBLD2	Homo sapiens
1630	deleted in colorectal carcinoma	DCC	Homo sapiens
8642	dachsous 1 (Drosophila)	DCHS1	Homo sapiens
1632	dodecenoyl-Coenzyme A delta isomerase (3,2 trans-enoyl-Coenzyme A isomerase)	DCI	Homo sapiens
1633	deoxycytidine kinase	DCK	Homo sapiens
1638	dopachrome tautomerase (dopachrome delta-isomerase, tyrosine-related protein 2)	DCT	Homo sapiens
1635	dCMP deaminase	DCTD	Homo sapiens
10540	dynactin 2 (p50)	DCTN2	Homo sapiens
10671	dynactin 6	DCTN6	Homo sapiens
1641	doublecortin	DCX	Homo sapiens
51181	dicarbonyl/L-xylulose reductase	DCXR	Homo sapiens
23576	dimethylarginine dimethylaminohydrolase 1	DDAH1	Homo sapiens
23564	dimethylarginine dimethylaminohydrolase 2	DDAH2	Homo sapiens
1643	damage-specific DNA binding protein 2, 48kDa	DDB2	Homo sapiens
1644	dopa decarboxylase (aromatic L-amino acid decarboxylase)	DDC	Homo sapiens
8528	D-aspartate oxidase	DDO	Homo sapiens
780	discoidin domain receptor tyrosine kinase 1	DDR1	Homo sapiens
4921	discoidin domain receptor tyrosine kinase 2	DDR2	Homo sapiens
1652	D-dopachrome tautomerase	DDT	Homo sapiens
1653	DEAD (Asp-Glu-Ala-Asp) box polypeptide 1	DDX1	Homo sapiens
1663	DEAD/H (Asp-Glu-Ala-Asp/His) box polypeptide 11 (CHL1-like helicase homolog, S. cerevisiae)	DDX11	Homo sapiens
10521	DEAD (Asp-Glu-Ala-Asp) box polypeptide 17	DDX17	Homo sapiens
8886	DEAD (Asp-Glu-Ala-Asp) box polypeptide 18	DDX18	Homo sapiens
9188	DEAD (Asp-Glu-Ala-Asp) box polypeptide 21	DDX21	Homo sapiens
9416	DEAD (Asp-Glu-Ala-Asp) box polypeptide 23	DDX23	Homo sapiens
55794	DEAD (Asp-Glu-Ala-Asp) box polypeptide 28	DDX28	Homo sapiens
10212	DEAD (Asp-Glu-Ala-Asp) box polypeptide 39	DDX39	Homo sapiens
1654	DEAD (Asp-Glu-Ala-Asp) box polypeptide 3, X-linked	DDX3X	Homo sapiens
51428	DEAD (Asp-Glu-Ala-Asp) box polypeptide 41	DDX41	Homo sapiens
54555	DEAD (Asp-Glu-Ala-Asp) box polypeptide 49	DDX49	Homo sapiens
1655	DEAD (Asp-Glu-Ala-Asp) box polypeptide 5	DDX5	Homo sapiens
317781	DEAD (Asp-Glu-Ala-Asp) box polypeptide 51	DDX51	Homo sapiens
11056	DEAD (Asp-Glu-Ala-Asp) box polypeptide 52	DDX52	Homo sapiens
10522	deformed epidermal autoregulatory factor 1 (Drosophila)	DEAF1	Homo sapiens
1666	2,4-dienoyl CoA reductase 1, mitochondrial	DECR1	Homo sapiens
9191	death effector domain containing	DEDD	Homo sapiens
1669	defensin, alpha 4, corticostatin	DEFA4	Homo sapiens
1672	defensin, beta 1	DEFB1	Homo sapiens
7913	DEK oncogene	DEK	Homo sapiens
1676	DNA fragmentation factor, 45kDa, alpha polypeptide	DFFA	Homo sapiens
1687	deafness, autosomal dominant 5	DFNA5	Homo sapiens
8694	diacylglycerol O-acyltransferase homolog 1 (mouse)	DGAT1	Homo sapiens
8220	DiGeorge syndrome critical region gene 14	DGCR14	Homo sapiens

8527	diacylglycerol kinase, delta 130kDa	DGKD	Homo sapiens
8525	diacylglycerol kinase, zeta 104kDa	DGKZ	Homo sapiens
1716	deoxyguanosine kinase	DGUOK	Homo sapiens
1718	24-dehydrocholesterol reductase	DHCR24	Homo sapiens
1717	7-dehydrocholesterol reductase	DHCR7	Homo sapiens
1723	dihydroorotate dehydrogenase	DHODH	Homo sapiens
1725	deoxyhypusine synthase	DHPS	Homo sapiens
9249	dehydrogenase/reductase (SDR family) member 3	DHRS3	Homo sapiens
10170	dehydrogenase/reductase (SDR family) member 9	DHRS9	Homo sapiens
1665	DEAH (Asp-Glu-Ala-His) box polypeptide 15	DHX15	Homo sapiens
54505	DEAH (Asp-Glu-Ala-His) box polypeptide 29	DHX29	Homo sapiens
22907	DEAH (Asp-Glu-Ala-His) box polypeptide 30	DHX30	Homo sapiens
1659	DEAH (Asp-Glu-Ala-His) box polypeptide 8	DHX8	Homo sapiens
1660	DEAH (Asp-Glu-Ala-His) box polypeptide 9	DHX9	Homo sapiens
23405	dicer 1, ribonuclease type III	DICER1	Homo sapiens
1733	deiodinase, iodothyronine, type I	DIO1	Homo sapiens
1735	deiodinase, iodothyronine, type III	DIO3	Homo sapiens
1736	dyskeratosis congenita 1, dyskerin	DKC1	Homo sapiens
22943	dickkopf homolog 1 (Xenopus laevis)	DKK1	Homo sapiens
27122	dickkopf homolog 3 (Xenopus laevis)	DKK3	Homo sapiens
10395	deleted in liver cancer 1	DLC1	Homo sapiens
1738	dihydrolipoamide dehydrogenase	DLD	Homo sapiens
9940	deleted in lung and esophageal cancer 1	DLEC1	Homo sapiens
1739	discs, large homolog 1 (Drosophila)	DLG1	Homo sapiens
1740	discs, large homolog 2 (Drosophila)	DLG2	Homo sapiens
1741	discs, large homolog 3 (Drosophila)	DLG3	Homo sapiens
9231	discs, large homolog 5 (Drosophila)	DLG5	Homo sapiens
9229	discs, large (Drosophila) homolog-associated protein 1	DLGAP1	Homo sapiens
9228	discs, large (Drosophila) homolog-associated protein 2	DLGAP2	Homo sapiens
22839	discs, large (Drosophila) homolog-associated protein 4	DLGAP4	Homo sapiens
1746	distal-less homeobox 2	DLX2	Homo sapiens
1748	distal-less homeobox 4	DLX4	Homo sapiens
1749	distal-less homeobox 5	DLX5	Homo sapiens
55929	DNA methyltransferase 1 associated protein 1	DMAP1	Homo sapiens
1756	dystrophin	DMD	Homo sapiens
9988	cyclin D binding myb-like transcription factor 1	DMTF1	Homo sapiens
1770	dynein, axonemal, heavy chain 9	DNAH9	Homo sapiens
10294	DnaJ (Hsp40) homolog, subfamily A, member 2	DNAJA2	Homo sapiens
9093	DnaJ (Hsp40) homolog, subfamily A, member 3	DNAJA3	Homo sapiens
3337	DnaJ (Hsp40) homolog, subfamily B, member 1	DNAJB1	Homo sapiens
51726	DnaJ (Hsp40) homolog, subfamily B, member 11	DNAJB11	Homo sapiens
25822	DnaJ (Hsp40) homolog, subfamily B, member 5	DNAJB5	Homo sapiens
4189	DnaJ (Hsp40) homolog, subfamily B, member 9	DNAJB9	Homo sapiens
23317	DnaJ (Hsp40) homolog, subfamily C, member 13	DNAJC13	Homo sapiens
22826	DnaJ (Hsp40) homolog, subfamily C, member 8	DNAJC8	Homo sapiens
10126	dynein, axonemal, light chain 4	DNAL4	Homo sapiens

1773	deoxyribonuclease I	DNASE1	Homo sapiens
1774	deoxyribonuclease I-like 1	DNASE1L1	Homo sapiens
1776	deoxyribonuclease I-like 3	DNASE1L3	Homo sapiens
1759	dynamin 1	DNM1	Homo sapiens
10059	dynamin 1-like	DNM1L	Homo sapiens
1786	DNA (cytosine-5-)-methyltransferase 1	DNMT1	Homo sapiens
1788	DNA (cytosine-5-)-methyltransferase 3 alpha	DNMT3A	Homo sapiens
1789	DNA (cytosine-5-)-methyltransferase 3 beta	DNMT3B	Homo sapiens
1793	dedicator of cytokinesis 1	DOCK1	Homo sapiens
1794	dedicator of cytokinesis 2	DOCK2	Homo sapiens
1796	docking protein 1-like protein; docking protein 1, 62kDa (downstream of tyrosine kinase 1)	DOK1	Homo sapiens
220077	docking protein 1-like protein; docking protein 1, 62kDa (downstream of tyrosine kinase 1)	DOK1	Homo sapiens
9046	docking protein 2, 56kDa	DOK2	Homo sapiens
55715	docking protein 4	DOK4	Homo sapiens
55816	docking protein 5	DOK5	Homo sapiens
1797	dom-3 homolog Z (C. elegans)	DOM3Z	Homo sapiens
64174	dipeptidase 2	DPEP2	Homo sapiens
5977	D4, zinc and double PHD fingers family 2	DPF2	Homo sapiens
8813	dolichyl-phosphate mannosyltransferase polypeptide 1, catalytic subunit	DPM1	Homo sapiens
8818	dolichyl-phosphate mannosyltransferase polypeptide 2, regulatory subunit	DPM2	Homo sapiens
1805	dermatopontin	DPT	Homo sapiens
1806	dihydropyrimidine dehydrogenase	DPYD	Homo sapiens
1807	dihydropyrimidinase	DPYS	Homo sapiens
1808	dihydropyrimidinase-like 2	DPYSL2	Homo sapiens
1809	dihydropyrimidinase-like 3	DPYSL3	Homo sapiens
1810	down-regulator of transcription 1, TBP-binding (negative cofactor 2)	DR1	Homo sapiens
1813	dopamine receptor D2	DRD2	Homo sapiens
4733	developmentally regulated GTP binding protein 1	DRG1	Homo sapiens
1821	dystrophin related protein 2	DRP2	Homo sapiens
1823	desmocollin 1	DSC1	Homo sapiens
1824	desmocollin 2	DSC2	Homo sapiens
1825	desmocollin 3	DSC3	Homo sapiens
1826	Down syndrome cell adhesion molecule	DSCAM	Homo sapiens
10311	Down syndrome critical region gene 3	DSCR3	Homo sapiens
1829	desmoglein 2	DSG2	Homo sapiens
1830	desmoglein 3 (pemphigus vulgaris antigen)	DSG3	Homo sapiens
1832	desmoplakin	DSP	Homo sapiens
667	dystonin	DST	Homo sapiens
11034	destrin (actin depolymerizing factor)	DSTN	Homo sapiens
1838	dystrobrevin, beta	DTNB	Homo sapiens
1841	deoxythymidylate kinase (thymidylate kinase); similar to Deoxythymidylate kinase (thymidylate kinase)	DTYMK	Homo sapiens
727761	deoxythymidylate kinase (thymidylate kinase); similar to	DTYMK	Homo sapiens

	Deoxythymidylate kinase (thymidylate kinase)		
53905	dual oxidase 1	DUOX1	Homo sapiens
50506	dual oxidase 2	DUOX2	Homo sapiens
1843	dual specificity phosphatase 1	DUSP1	Homo sapiens
11221	dual specificity phosphatase 10	DUSP10	Homo sapiens
8446	dual specificity phosphatase 11 (RNA/RNP complex 1-interacting)	DUSP11	Homo sapiens
11072	dual specificity phosphatase 14	DUSP14	Homo sapiens
1845	dual specificity phosphatase 3	DUSP3	Homo sapiens
1846	dual specificity phosphatase 4	DUSP4	Homo sapiens
1847	dual specificity phosphatase 5	DUSP5	Homo sapiens
1848	dual specificity phosphatase 6	DUSP6	Homo sapiens
1850	dual specificity phosphatase 8	DUSP8	Homo sapiens
1854	deoxyuridine triphosphatase	DUT	Homo sapiens
8215	dishevelled, dsh homolog 1 (Drosophila); dishevelled, dsh homolog 1 (Drosophila)-like 1	DVL1	Homo sapiens
1855	dishevelled, dsh homolog 1 (Drosophila); dishevelled, dsh homolog 1 (Drosophila)-like 1	DVL1	Homo sapiens
1857	dishevelled, dsh homolog 3 (Drosophila)	DVL3	Homo sapiens
1859	dual-specificity tyrosine-(Y)-phosphorylation regulated kinase 1A	DYRK1A	Homo sapiens
8445	dual-specificity tyrosine-(Y)-phosphorylation regulated kinase 2	DYRK2	Homo sapiens
8444	dual-specificity tyrosine-(Y)-phosphorylation regulated kinase 3	DYRK3	Homo sapiens
8798	dual-specificity tyrosine-(Y)-phosphorylation regulated kinase 4	DYRK4	Homo sapiens
1869	E2F transcription factor 1	E2F1	Homo sapiens
1871	E2F transcription factor 3	E2F3	Homo sapiens
1875	E2F transcription factor 5, p130-binding	E2F5	Homo sapiens
10682	emopamil binding protein (sterol isomerase)	EBP	Homo sapiens
1889	endothelin converting enzyme 1	ECE1	Homo sapiens
9718	endothelin converting enzyme 2	ECE2	Homo sapiens
1891	enoyl Coenzyme A hydratase 1, peroxisomal	ECH1	Homo sapiens
1892	enoyl Coenzyme A hydratase, short chain, 1, mitochondrial	ECHS1	Homo sapiens
1893	extracellular matrix protein 1	ECM1	Homo sapiens
1842	extracellular matrix protein 2, female organ and adipocyte specific	ECM2	Homo sapiens
1894	epithelial cell transforming sequence 2 oncogene	ECT2	Homo sapiens
8721	endothelial differentiation-related factor 1	EDF1	Homo sapiens
10085	EGF-like repeats and discoidin I-like domains 3	EDIL3	Homo sapiens
1906	endothelin 1	EDN1	Homo sapiens
1907	endothelin 2	EDN2	Homo sapiens
1908	endothelin 3	EDN3	Homo sapiens
1909	endothelin receptor type A	EDNRA	Homo sapiens
1910	endothelin receptor type B	EDNRB	Homo sapiens
8411	early endosome antigen 1	EEA1	Homo sapiens
8726	embryonic ectoderm development	EED	Homo sapiens
9521	eukaryotic translation elongation factor 1 epsilon 1	EEF1E1	Homo sapiens
30008	EGF-containing fibulin-like extracellular matrix protein 2	EFEMP2	Homo sapiens
80303	EF-hand domain family, member D1	EFHD1	Homo sapiens
1942	ephrin-A1	EFNA1	Homo sapiens

1947	ephrin-B1	EFNB1	Homo sapiens
1948	ephrin-B2	EFNB2	Homo sapiens
1949	ephrin-B3	EFNB3	Homo sapiens
10278	embryonal Fyn-associated substrate	EFS	Homo sapiens
1950	epidermal growth factor (beta-urogastrone)	EGF	Homo sapiens
1958	early growth response 1	EGR1	Homo sapiens
1959	early growth response 2	EGR2	Homo sapiens
1960	early growth response 3	EGR3	Homo sapiens
26298	ets homologous factor	EHF	Homo sapiens
1962	enoyl-Coenzyme A, hydratase/3-hydroxyacyl Coenzyme A dehydrogenase	EHHADH	Homo sapiens
9538	etoposide induced 2.4 mRNA	EI24	Homo sapiens
9086	eukaryotic translation initiation factor 1A, Y-linked	EIF1AY	Homo sapiens
8892	eukaryotic translation initiation factor 2B, subunit 2 beta, 39kDa	EIF2B2	Homo sapiens
8890	eukaryotic translation initiation factor 2B, subunit 4 delta, 67kDa	EIF2B4	Homo sapiens
8893	eukaryotic translation initiation factor 2B, subunit 5 epsilon, 82kDa	EIF2B5	Homo sapiens
27161	eukaryotic translation initiation factor 2C, 2	EIF2C2	Homo sapiens
1965	eukaryotic translation initiation factor 2, subunit 1 alpha, 35kDa	EIF2S1	Homo sapiens
8894	eukaryotic translation initiation factor 2, subunit 2 beta, 38kDa	EIF2S2	Homo sapiens
1968	eukaryotic translation initiation factor 2, subunit 3 gamma, 52kDa	EIF2S3	Homo sapiens
1973	similar to eukaryotic translation initiation factor 4A; small nucleolar RNA, H/ACA box 67; eukaryotic translation initiation factor 4A, isoform 1	EIF4A1	Homo sapiens
26781	similar to eukaryotic translation initiation factor 4A; small nucleolar RNA, H/ACA box 67; eukaryotic translation initiation factor 4A, isoform 1	EIF4A1	Homo sapiens
728698	similar to eukaryotic translation initiation factor 4A; small nucleolar RNA, H/ACA box 67; eukaryotic translation initiation factor 4A, isoform 1	EIF4A1	Homo sapiens
1974	similar to eukaryotic translation initiation factor 4A2; eukaryotic translation initiation factor 4A, isoform 2	EIF4A2	Homo sapiens
286512	similar to eukaryotic translation initiation factor 4A2; eukaryotic translation initiation factor 4A, isoform 2	EIF4A2	Homo sapiens
643873	similar to eukaryotic translation initiation factor 4H; eukaryotic translation initiation factor 4B	EIF4B	Homo sapiens
1975	similar to eukaryotic translation initiation factor 4H; eukaryotic translation initiation factor 4B	EIF4B	Homo sapiens
1978	eukaryotic translation initiation factor 4E binding protein 1	EIF4EBP1	Homo sapiens
1981	eukaryotic translation initiation factor 4 gamma, 1	EIF4G1	Homo sapiens
1983	eukaryotic translation initiation factor 5	EIF5	Homo sapiens
143244	eukaryotic translation initiation factor 5A; eukaryotic translation initiation factor 5A-like 1	EIF5A	Homo sapiens
1984	eukaryotic translation initiation factor 5A; eukaryotic translation initiation factor 5A-like 1	EIF5A	Homo sapiens
56648	eukaryotic translation initiation factor 5A2	EIF5A2	Homo sapiens
1994	ELAV (embryonic lethal, abnormal vision, Drosophila)-like 1 (Hu antigen R)	ELAVL1	Homo sapiens
1996	ELAV (embryonic lethal, abnormal vision, Drosophila)-like 4 (Hu antigen D)	ELAVL4	Homo sapiens

1997	E74-like factor 1 (ets domain transcription factor)	ELF1	Homo sapiens
1998	E74-like factor 2 (ets domain transcription factor)	ELF2	Homo sapiens
1999	E74-like factor 3 (ets domain transcription factor, epithelial-specific)	ELF3	Homo sapiens
2000	E74-like factor 4 (ets domain transcription factor)	ELF4	Homo sapiens
2002	ELK1, member of ETS oncogene family	ELK1	Homo sapiens
2004	ELK3, ETS-domain protein (SRF accessory protein 2)	ELK3	Homo sapiens
2005	ELK4, ETS-domain protein (SRF accessory protein 1)	ELK4	Homo sapiens
8178	elongation factor RNA polymerase II	ELL	Homo sapiens
22936	elongation factor, RNA polymerase II, 2	ELL2	Homo sapiens
80237	elongation factor RNA polymerase II-like 3	ELL3	Homo sapiens
9844	engulfment and cell motility 1	ELMO1	Homo sapiens
2006	elastin	ELN	Homo sapiens
64834	elongation of very long chain fatty acids (FEN1/Elo2, SUR4/Elo3, yeast)-like 1	ELOVL1	Homo sapiens
54898	elongation of very long chain fatty acids (FEN1/Elo2, SUR4/Elo3, yeast)-like 2	ELOVL2	Homo sapiens
60481	ELOVL family member 5, elongation of long chain fatty acids (FEN1/Elo2, SUR4/Elo3-like, yeast)	ELOVL5	Homo sapiens
79071	ELOVL family member 6, elongation of long chain fatty acids (FEN1/Elo2, SUR4/Elo3-like, yeast)	ELOVL6	Homo sapiens
26610	elongation protein 4 homolog (S. cerevisiae)	ELP4	Homo sapiens
2010	emerin	EMD	Homo sapiens
2009	echinoderm microtubule associated protein like 1	EML1	Homo sapiens
24139	echinoderm microtubule associated protein like 2	EML2	Homo sapiens
2013	epithelial membrane protein 2	EMP2	Homo sapiens
2014	epithelial membrane protein 3	EMP3	Homo sapiens
8507	ectodermal-neural cortex (with BTB-like domain)	ENC1	Homo sapiens
2022	endoglin	ENG	Homo sapiens
2023	enolase 1, (alpha)	ENO1	Homo sapiens
2026	enolase 2 (gamma, neuronal)	ENO2	Homo sapiens
2028	glutamyl aminopeptidase (aminopeptidase A)	ENPEP	Homo sapiens
5167	ectonucleotide pyrophosphatase/phosphodiesterase 1	ENPP1	Homo sapiens
5168	ectonucleotide pyrophosphatase/phosphodiesterase 2	ENPP2	Homo sapiens
133121	ectonucleotide pyrophosphatase/phosphodiesterase 6	ENPP6	Homo sapiens
2029	endosulfine alpha	ENSA	Homo sapiens
954	ectonucleoside triphosphate diphosphohydrolase 2	ENTPD2	Homo sapiens
956	ectonucleoside triphosphate diphosphohydrolase 3	ENTPD3	Homo sapiens
957	ectonucleoside triphosphate diphosphohydrolase 5	ENTPD5	Homo sapiens
377841	ectonucleoside triphosphate diphosphohydrolase 8	ENTPD8	Homo sapiens
2033	E1A binding protein p300	EP300	Homo sapiens
57634	E1A binding protein p400	EP400	Homo sapiens
2034	endothelial PAS domain protein 1	EPAS1	Homo sapiens
2035	erythrocyte membrane protein band 4.1 (elliptocytosis 1, RH-linked)	EPB41	Homo sapiens
2036	erythrocyte membrane protein band 4.1-like 1	EPB41L1	Homo sapiens
23136	erythrocyte membrane protein band 4.1-like 3	EPB41L3	Homo sapiens
2038	erythrocyte membrane protein band 4.2	EPB42	Homo sapiens
80314	enhancer of polycomb homolog 1 (Drosophila)	EPC1	Homo sapiens

2041	EPH receptor A1	EPHA1	Homo sapiens
2043	EPH receptor A4	EPHA4	Homo sapiens
2044	EPH receptor A5	EPHA5	Homo sapiens
2048	EPH receptor B2	EPHB2	Homo sapiens
2050	EPH receptor B4	EPHB4	Homo sapiens
2052	epoxide hydrolase 1, microsomal (xenobiotic)	EPHX1	Homo sapiens
7957	epilepsy, progressive myoclonus type 2A, Lafora disease (laforin)	EPM2A	Homo sapiens
22905	epsin 2	EPN2	Homo sapiens
2056	erythropoietin	EPO	Homo sapiens
2057	erythropoietin receptor	EPOR	Homo sapiens
2058	glutamyl-prolyl-tRNA synthetase	EPRS	Homo sapiens
2060	epidermal growth factor receptor pathway substrate 15	EPS15	Homo sapiens
2059	epidermal growth factor receptor pathway substrate 8	EPS8	Homo sapiens
26284	Era G-protein-like 1 (E. coli)	ERAL1	Homo sapiens
2064	v-erb-b2 erythroblastic leukemia viral oncogene homolog 2, neuro/glioblastoma derived oncogene homolog (avian)	ERBB2	Homo sapiens
2065	v-erb-b2 erythroblastic leukemia viral oncogene homolog 3 (avian)	ERBB3	Homo sapiens
2066	v-erb-a erythroblastic leukemia viral oncogene homolog 4 (avian)	ERBB4	Homo sapiens
2067	excision repair cross-complementing rodent repair deficiency, complementation group 1 (includes overlapping antisense sequence)	ERCC1	Homo sapiens
2068	excision repair cross-complementing rodent repair deficiency, complementation group 2	ERCC2	Homo sapiens
2071	excision repair cross-complementing rodent repair deficiency, complementation group 3 (xeroderma pigmentosum group B complementing)	ERCC3	Homo sapiens
2073	excision repair cross-complementing rodent repair deficiency, complementation group 5	ERCC5	Homo sapiens
2069	epiregulin	EREG	Homo sapiens
2078	v-ets erythroblastosis virus E26 oncogene homolog (avian)	ERG	Homo sapiens
2081	endoplasmic reticulum to nucleus signaling 1	ERN1	Homo sapiens
2098	esterase D/formylglutathione hydrolase	ESD	Homo sapiens
11082	endothelial cell-specific molecule 1	ESM1	Homo sapiens
2099	estrogen receptor 1	ESR1	Homo sapiens
2103	estrogen-related receptor beta	ESRRB	Homo sapiens
2104	estrogen-related receptor gamma	ESRRG	Homo sapiens
2107	eukaryotic translation termination factor 1	ETF1	Homo sapiens
2108	electron-transfer-flavoprotein, alpha polypeptide	ETFA	Homo sapiens
2109	electron-transfer-flavoprotein, beta polypeptide	ETFB	Homo sapiens
2110	electron-transferring-flavoprotein dehydrogenase	ETFDH	Homo sapiens
55500	ethanolamine kinase 1	ETNK1	Homo sapiens
55224	ethanolamine kinase 2	ETNK2	Homo sapiens
2114	v-ets erythroblastosis virus E26 oncogene homolog 2 (avian)	ETS2	Homo sapiens
2117	ets variant 3	ETV3	Homo sapiens
2118	ets variant 4	ETV4	Homo sapiens
2120	ets variant 6	ETV6	Homo sapiens
2124	ecotropic viral integration site 2B	EVI2B	Homo sapiens
115704	ecotropic viral integration site 5-like	EVI5L	Homo sapiens

2125	envoplakin	EVPL	Homo sapiens
2128	even-skipped homeobox 1	EVX1	Homo sapiens
2130	similar to Ewing sarcoma breakpoint region 1; Ewing sarcoma breakpoint region 1	EWSR1	Homo sapiens
284685	similar to Ewing sarcoma breakpoint region 1; Ewing sarcoma breakpoint region 1	EWSR1	Homo sapiens
9156	exonuclease 1	EXO1	Homo sapiens
51013	exosome component 1	EXOSC1	Homo sapiens
23016	exosome component 7	EXOSC7	Homo sapiens
2131	exostoses (multiple) 1	EXT1	Homo sapiens
2132	exostoses (multiple) 2	EXT2	Homo sapiens
2135	exostoses (multiple)-like 2	EXTL2	Homo sapiens
2139	eyes absent homolog 2 (Drosophila)	EYA2	Homo sapiens
2140	eyes absent homolog 3 (Drosophila)	EYA3	Homo sapiens
2145	enhancer of zeste homolog 1 (Drosophila)	EZH1	Homo sapiens
2146	enhancer of zeste homolog 2 (Drosophila)	EZH2	Homo sapiens
2159	coagulation factor X	F10	Homo sapiens
2160	coagulation factor XI	F11	Homo sapiens
50848	F11 receptor	F11R	Homo sapiens
2147	coagulation factor II (thrombin)	F2	Homo sapiens
2149	coagulation factor II (thrombin) receptor	F2R	Homo sapiens
2150	coagulation factor II (thrombin) receptor-like 1	F2RL1	Homo sapiens
9002	coagulation factor II (thrombin) receptor-like 3	F2RL3	Homo sapiens
2152	coagulation factor III (thromboplastin, tissue factor)	F3	Homo sapiens
2153	coagulation factor V (proaccelerin, labile factor)	F5	Homo sapiens
2155	coagulation factor VII (serum prothrombin conversion accelerator)	F7	Homo sapiens
2157	coagulation factor VIII, procoagulant component	F8	Homo sapiens
79152	fatty acid 2-hydroxylase	FA2H	Homo sapiens
2166	fatty acid amide hydrolase	FAAH	Homo sapiens
2168	fatty acid binding protein 1, liver	FABP1	Homo sapiens
2167	fatty acid binding protein 4, adipocyte	FABP4	Homo sapiens
728729	fatty acid binding protein 5-like 2; fatty acid binding protein 5 (psoriasis-associated); fatty acid binding protein 5-like 8; fatty acid binding protein 5-like 7; fatty acid binding protein 5-like 9	FABP5	Homo sapiens
729163	fatty acid binding protein 5-like 2; fatty acid binding protein 5 (psoriasis-associated); fatty acid binding protein 5-like 8; fatty acid binding protein 5-like 7; fatty acid binding protein 5-like 9	FABP5	Homo sapiens
642956	fatty acid binding protein 5-like 2; fatty acid binding protein 5 (psoriasis-associated); fatty acid binding protein 5-like 8; fatty acid binding protein 5-like 7; fatty acid binding protein 5-like 9	FABP5	Homo sapiens
2171	fatty acid binding protein 5-like 2; fatty acid binding protein 5 (psoriasis-associated); fatty acid binding protein 5-like 8; fatty acid binding protein 5-like 7; fatty acid binding protein 5-like 9	FABP5	Homo sapiens
728641	fatty acid binding protein 5-like 2; fatty acid binding protein 5 (psoriasis-associated); fatty acid binding protein 5-like 8; fatty acid binding protein 5-like 7; fatty acid binding protein 5-like 9	FABP5	Homo sapiens
2172	fatty acid binding protein 6, ileal	FABP6	Homo sapiens
2173	fatty acid binding protein 7, brain	FABP7	Homo sapiens

8772	Fas (TNFRSF6)-associated via death domain	FADD	Homo sapiens
9415	fatty acid desaturase 2	FADS2	Homo sapiens
3995	fatty acid desaturase 3	FADS3	Homo sapiens
2184	fumarylacetoacetate hydrolase (fumarylacetoacetase)	FAH	Homo sapiens
55179	Fas apoptotic inhibitory molecule	FAIM	Homo sapiens
23017	Fas apoptotic inhibitory molecule 2	FAIM2	Homo sapiens
60343	family with sequence similarity 3, member A	FAM3A	Homo sapiens
55578	family with sequence similarity 48, member A	FAM48A	Homo sapiens
51439	family with sequence similarity 8, member A1	FAM8A1	Homo sapiens
2176	Fanconi anemia, complementation group C	FANCC	Homo sapiens
2189	Fanconi anemia, complementation group G	FANCG	Homo sapiens
55120	Fanconi anemia, complementation group L	FANCL	Homo sapiens
2191	fibroblast activation protein, alpha	FAP	Homo sapiens
10160	FERM, RhoGEF (ARHGEF) and pleckstrin domain protein 1 (chondrocyte-derived)	FARP1	Homo sapiens
2194	fatty acid synthase	FASN	Homo sapiens
2091	fibrillarin	FBL	Homo sapiens
2192	fibulin 1	FBLN1	Homo sapiens
2199	fibulin 2	FBLN2	Homo sapiens
10516	fibulin 5	FBLN5	Homo sapiens
2203	fructose-1,6-bisphosphatase 1	FBP1	Homo sapiens
8789	fructose-1,6-bisphosphatase 2	FBP2	Homo sapiens
25827	F-box and leucine-rich repeat protein 2	FBXL2	Homo sapiens
26235	F-box and leucine-rich repeat protein 4	FBXL4	Homo sapiens
23014	F-box protein 21	FBXO21	Homo sapiens
26271	F-box protein 5	FBXO5	Homo sapiens
26268	F-box protein 9	FBXO9	Homo sapiens
23291	F-box and WD repeat domain containing 11	FBXW11	Homo sapiens
55294	F-box and WD repeat domain containing 7	FBXW7	Homo sapiens
2207	Fc fragment of IgE, high affinity I, receptor for; gamma polypeptide	FCER1G	Homo sapiens
2208	Fc fragment of IgE, low affinity II, receptor for (CD23)	FCER2	Homo sapiens
9103	Fc fragment of IgG, low affinity IIb, receptor (CD32); Fc fragment of IgG, low affinity IIc, receptor for (CD32)	FCGR2B	Homo sapiens
2213	Fc fragment of IgG, low affinity IIb, receptor (CD32); Fc fragment of IgG, low affinity IIc, receptor for (CD32)	FCGR2B	Homo sapiens
2217	Fc fragment of IgG, receptor, transporter, alpha	FCGRT	Homo sapiens
2219	ficolin (collagen/fibrinogen domain containing) 1	FCN1	Homo sapiens
8547	ficolin (collagen/fibrinogen domain containing) 3 (Hakata antigen)	FCN3	Homo sapiens
2222	farnesyl-diphosphate farnesyltransferase 1	FDFT1	Homo sapiens
2224	farnesyl diphosphate synthase (farnesyl pyrophosphate synthetase, dimethylallyltranstransferase, geranyltranstransferase)	FDPS	Homo sapiens
2230	ferredoxin 1	FDX1	Homo sapiens
2232	ferredoxin reductase	FDXR	Homo sapiens
2235	ferrochelatase (protoporphyria)	FECH	Homo sapiens
10116	fem-1 homolog b (C. elegans)	FEM1B	Homo sapiens
2237	flap structure-specific endonuclease 1	FEN1	Homo sapiens
2241	fer (fps/fes related) tyrosine kinase	FER	Homo sapiens

26998	fetuin B	FETUB	Homo sapiens
2243	fibrinogen alpha chain	FGA	Homo sapiens
2245	FYVE, RhoGEF and PH domain containing 1	FGD1	Homo sapiens
2246	fibroblast growth factor 1 (acidic)	FGF1	Homo sapiens
2257	fibroblast growth factor 12	FGF12	Homo sapiens
8817	fibroblast growth factor 18	FGF18	Homo sapiens
2247	fibroblast growth factor 2 (basic)	FGF2	Homo sapiens
26281	fibroblast growth factor 20	FGF20	Homo sapiens
2248	fibroblast growth factor 3 (murine mammary tumor virus integration site (v-int-2) oncogene homolog)	FGF3	Homo sapiens
394217	hypothetical LOC100132771; fibroblast growth factor 7 (keratinocyte growth factor); fibroblast growth factor 7 pseudogene 2	FGF7	Homo sapiens
2252	hypothetical LOC100132771; fibroblast growth factor 7 (keratinocyte growth factor); fibroblast growth factor 7 pseudogene 2	FGF7	Homo sapiens
100132771	hypothetical LOC100132771; fibroblast growth factor 7 (keratinocyte growth factor); fibroblast growth factor 7 pseudogene 2	FGF7	Homo sapiens
2260	fibroblast growth factor receptor 1	FGFR1	Homo sapiens
2261	fibroblast growth factor receptor 3	FGFR3	Homo sapiens
2264	fibroblast growth factor receptor 4	FGFR4	Homo sapiens
2267	fibrinogen-like 1	FGL1	Homo sapiens
10875	fibrinogen-like 2	FGL2	Homo sapiens
2268	Gardner-Rasheed feline sarcoma viral (v-fgr) oncogene homolog	FGR	Homo sapiens
2271	fumarate hydratase	FH	Homo sapiens
2273	four and a half LIM domains 1	FHL1	Homo sapiens
2274	four and a half LIM domains 2	FHL2	Homo sapiens
9158	fibroblast growth factor (acidic) intracellular binding protein	FIBP	Homo sapiens
2277	c-fos induced growth factor (vascular endothelial growth factor D)	FIGF	Homo sapiens
63979	fidgetin-like 1	FIGNL1	Homo sapiens
2286	FK506 binding protein 2, 13kDa	FKBP2	Homo sapiens
2288	FK506 binding protein 4, 59kDa	FKBP4	Homo sapiens
23770	FK506 binding protein 8, 38kDa	FKBP8	Homo sapiens
2314	flightless I homolog (Drosophila)	FLII	Homo sapiens
2316	filamin A, alpha (actin binding protein 280)	FLNA	Homo sapiens
2317	filamin B, beta (actin binding protein 278)	FLNB	Homo sapiens
2318	filamin C, gamma (actin binding protein 280)	FLNC	Homo sapiens
10211	flotillin 1	FLOT1	Homo sapiens
2319	flotillin 2	FLOT2	Homo sapiens
23769	fibronectin leucine rich transmembrane protein 1	FLRT1	Homo sapiens
2322	fms-related tyrosine kinase 3	FLT3	Homo sapiens
2323	fms-related tyrosine kinase 3 ligand	FLT3LG	Homo sapiens
2324	fms-related tyrosine kinase 4	FLT4	Homo sapiens
752	formin-like 1	FMNL1	Homo sapiens
2326	flavin containing monooxygenase 1	FMO1	Homo sapiens
2327	flavin containing monooxygenase 2 (non-functional)	FMO2	Homo sapiens
2329	flavin containing monooxygenase 4	FMO4	Homo sapiens
2330	flavin containing monooxygenase 5	FMO5	Homo sapiens
2331	fibromodulin	FMOD	Homo sapiens

2335	fibronectin 1	FN1	Homo sapiens
23360	formin binding protein 4	FNBP4	Homo sapiens
2342	farnesyltransferase, CAAX box, beta	FNTB	Homo sapiens
2348	folate receptor 1 (adult)	FOLR1	Homo sapiens
2350	folate receptor 2 (fetal)	FOLR2	Homo sapiens
2352	folate receptor 3 (gamma)	FOLR3	Homo sapiens
2353	v-fos FBJ murine osteosarcoma viral oncogene homolog	FOS	Homo sapiens
2354	FBJ murine osteosarcoma viral oncogene homolog B	FOSB	Homo sapiens
8061	FOS-like antigen 1	FOSL1	Homo sapiens
2355	FOS-like antigen 2	FOSL2	Homo sapiens
3170	forkhead box A2	FOXA2	Homo sapiens
3171	forkhead box A3	FOXA3	Homo sapiens
2296	forkhead box C1	FOXC1	Homo sapiens
2306	forkhead box D2	FOXD2	Homo sapiens
2294	forkhead box F1	FOXF1	Homo sapiens
2295	forkhead box F2	FOXF2	Homo sapiens
2299	forkhead box I1	FOXI1	Homo sapiens
2302	forkhead box J1	FOXJ1	Homo sapiens
55810	forkhead box J2	FOXJ2	Homo sapiens
22887	forkhead box J3	FOXJ3	Homo sapiens
2305	forkhead box M1	FOXM1	Homo sapiens
94234	forkhead box Q1	FOXQ1	Homo sapiens
2356	folylpolyglutamate synthase	FPGS	Homo sapiens
51086	TNNI3 interacting kinase; fucose-1-phosphate guanylyltransferase	FPGT	Homo sapiens
8790	TNNI3 interacting kinase; fucose-1-phosphate guanylyltransferase	FPGT	Homo sapiens
2487	frizzled-related protein	FRZB	Homo sapiens
25794	fascin homolog 2, actin-bundling protein, retinal (Strongylocentrotus purpuratus)	FSCN2	Homo sapiens
10468	follistatin	FST	Homo sapiens
10272	follistatin-like 3 (secreted glycoprotein)	FSTL3	Homo sapiens
2508	ferritin, heavy polypeptide 1; ferritin, heavy polypeptide-like 16; similar to ferritin, heavy polypeptide 1; ferritin, heavy polypeptide-like 3 pseudogene	FTH1	Homo sapiens
729009	ferritin, heavy polypeptide 1; ferritin, heavy polypeptide-like 16; similar to ferritin, heavy polypeptide 1; ferritin, heavy polypeptide-like 3 pseudogene	FTH1	Homo sapiens
2495	ferritin, heavy polypeptide 1; ferritin, heavy polypeptide-like 16; similar to ferritin, heavy polypeptide 1; ferritin, heavy polypeptide-like 3 pseudogene	FTH1	Homo sapiens
2498	ferritin, heavy polypeptide 1; ferritin, heavy polypeptide-like 16; similar to ferritin, heavy polypeptide 1; ferritin, heavy polypeptide-like 3 pseudogene	FTH1	Homo sapiens
24140	FtsJ homolog 1 (E. coli)	FTSJ1	Homo sapiens
8880	far upstream element (FUSE) binding protein 1	FUBP1	Homo sapiens
5045	furin (paired basic amino acid cleaving enzyme)	FURIN	Homo sapiens
2521	fusion (involved in t(12;16) in malignant liposarcoma)	FUS	Homo sapiens
2523	fucosyltransferase 1 (galactoside 2-alpha-L-fucosyltransferase, H blood group)	FUT1	Homo sapiens

2530	fucosyltransferase 8 (alpha (1,6) fucosyltransferase)	FUT8	Homo sapiens
2395	frataxin	FXN	Homo sapiens
8087	fragile X mental retardation, autosomal homolog 1	FXR1	Homo sapiens
486	FXYD domain containing ion transport regulator 2	FXYD2	Homo sapiens
5349	FXYD domain containing ion transport regulator 3	FXYD3	Homo sapiens
53827	FXYD domain containing ion transport regulator 5	FXYD5	Homo sapiens
2533	FYN binding protein (FYB-120/130)	FYB	Homo sapiens
2534	FYN oncogene related to SRC, FGR, YES	FYN	Homo sapiens
8321	frizzled homolog 1 (Drosophila)	FZD1	Homo sapiens
2535	frizzled homolog 2 (Drosophila)	FZD2	Homo sapiens
8322	frizzled homolog 4 (Drosophila)	FZD4	Homo sapiens
7855	frizzled homolog 5 (Drosophila)	FZD5	Homo sapiens
8323	frizzled homolog 6 (Drosophila)	FZD6	Homo sapiens
8324	frizzled homolog 7 (Drosophila)	FZD7	Homo sapiens
51343	fizzy/cell division cycle 20 related 1 (Drosophila)	FZR1	Homo sapiens
2538	glucose-6-phosphatase, catalytic subunit	G6PC	Homo sapiens
2539	glucose-6-phosphate dehydrogenase	G6PD	Homo sapiens
2549	GRB2-associated binding protein 1	GAB1	Homo sapiens
9846	GRB2-associated binding protein 2	GAB2	Homo sapiens
11337	GABA(A) receptor-associated protein	GABARAP	Homo sapiens
2551	GA binding protein transcription factor, alpha subunit 60kDa	GABPA	Homo sapiens
126626	GA binding protein transcription factor, beta subunit 2	GABPB2	Homo sapiens
2554	gamma-aminobutyric acid (GABA) A receptor, alpha 1	GABRA1	Homo sapiens
2555	gamma-aminobutyric acid (GABA) A receptor, alpha 2	GABRA2	Homo sapiens
2559	gamma-aminobutyric acid (GABA) A receptor, alpha 6	GABRA6	Homo sapiens
2560	gamma-aminobutyric acid (GABA) A receptor, beta 1	GABRB1	Homo sapiens
2562	gamma-aminobutyric acid (GABA) A receptor, beta 3	GABRB3	Homo sapiens
2563	gamma-aminobutyric acid (GABA) A receptor, delta	GABRD	Homo sapiens
2564	gamma-aminobutyric acid (GABA) A receptor, epsilon	GABRE	Homo sapiens
2566	gamma-aminobutyric acid (GABA) A receptor, gamma 2	GABRG2	Homo sapiens
2568	gamma-aminobutyric acid (GABA) A receptor, pi	GABRP	Homo sapiens
2571	glutamate decarboxylase 1 (brain, 67kDa)	GAD1	Homo sapiens
10912	growth arrest and DNA-damage-inducible, gamma	GADD45G	Homo sapiens
2580	cyclin G associated kinase	GAK	Homo sapiens
51083	galanin prepropeptide	GAL	Homo sapiens
2582	UDP-galactose-4-epimerase	GALE	Homo sapiens
2584	galactokinase 1	GALK1	Homo sapiens
2585	galactokinase 2	GALK2	Homo sapiens
2589	UDP-N-acetyl-alpha-D-galactosamine:polypeptide N-acetylgalactosaminyltransferase 13 (GalNAc-T13); UDP-N-acetyl-alpha-D-galactosamine:polypeptide N-acetylgalactosaminyltransferase 1 (GalNAc-T1)	GALNT1	Homo sapiens
114805	UDP-N-acetyl-alpha-D-galactosamine:polypeptide N-acetylgalactosaminyltransferase 13 (GalNAc-T13); UDP-N-acetyl-alpha-D-galactosamine:polypeptide N-acetylgalactosaminyltransferase 1 (GalNAc-T1)	GALNT1	Homo sapiens
2591	UDP-N-acetyl-alpha-D-galactosamine:polypeptide N-	GALNT3	Homo sapiens

	acetylgalactosaminyltransferase 3 (GalNAc-T3)		
8484	galanin receptor 3	GALR3	Homo sapiens
2592	galactose-1-phosphate uridylyltransferase	GALT	Homo sapiens
2593	guanidinoacetate N-methyltransferase	GAMT	Homo sapiens
2596	growth associated protein 43	GAP43	Homo sapiens
2617	glycyl-tRNA synthetase	GARS	Homo sapiens
2618	phosphoribosylglycinamide formyltransferase, phosphoribosylglycinamide synthetase, phosphoribosylaminoimidazole synthetase	GART	Homo sapiens
10634	growth arrest-specific 2 like 1	GAS2L1	Homo sapiens
2621	similar to growth arrest-specific 6; growth arrest-specific 6	GAS6	Homo sapiens
732446	similar to growth arrest-specific 6; growth arrest-specific 6	GAS6	Homo sapiens
8522	growth arrest-specific 7	GAS7	Homo sapiens
2622	growth arrest-specific 8	GAS8	Homo sapiens
2624	GATA binding protein 2	GATA2	Homo sapiens
2625	GATA binding protein 3	GATA3	Homo sapiens
2626	GATA binding protein 4	GATA4	Homo sapiens
2627	GATA binding protein 6	GATA6	Homo sapiens
2629	glucosidase, beta; acid (includes glucosylceramidase)	GBA	Homo sapiens
57704	glucosidase, beta (bile acid) 2	GBA2	Homo sapiens
8729	golgi-specific brefeldin A resistant guanine nucleotide exchange factor 1	GBF1	Homo sapiens
2633	guanylate binding protein 1, interferon-inducible, 67kDa	GBP1	Homo sapiens
2634	guanylate binding protein 2, interferon-inducible	GBP2	Homo sapiens
2637	gastrulation brain homeobox 2	GBX2	Homo sapiens
25801	grancalcin, EF-hand calcium binding protein	GCA	Homo sapiens
23464	glycine C-acetyltransferase (2-amino-3-ketobutyrate coenzyme A ligase)	GCAT	Homo sapiens
9648	GRIP and coiled-coil domain containing 2	GCC2	Homo sapiens
2639	glutaryl-Coenzyme A dehydrogenase	GCDH	Homo sapiens
2643	GTP cyclohydrolase 1	GCH1	Homo sapiens
2644	GTP cyclohydrolase I feedback regulator	GCHFR	Homo sapiens
2646	glucokinase (hexokinase 4) regulator	GCKR	Homo sapiens
2730	glutamate-cysteine ligase, modifier subunit	GCLM	Homo sapiens
8521	glial cells missing homolog 1 (Drosophila)	GCM1	Homo sapiens
10985	GCN1 general control of amino-acid synthesis 1-like 1 (yeast)	GCN1L1	Homo sapiens
2650	glucosaminyl (N-acetyl) transferase 1, core 2 (beta-1,6-N-acetylglucosaminyltransferase)	GCNT1	Homo sapiens
2651	glucosaminyl (N-acetyl) transferase 2, I-branching enzyme (I blood group)	GCNT2	Homo sapiens
9615	guanine deaminase	GDA	Homo sapiens
2662	growth differentiation factor 10	GDF10	Homo sapiens
9518	growth differentiation factor 15	GDF15	Homo sapiens
8200	growth differentiation factor 5	GDF5	Homo sapiens
2664	GDP dissociation inhibitor 1	GDI1	Homo sapiens
54857	glycerophosphodiester phosphodiesterase domain containing 2	GDPD2	Homo sapiens
2669	GTP binding protein overexpressed in skeletal muscle	GEM	Homo sapiens

25929	gem (nuclear organelle) associated protein 5	GEMIN5	Homo sapiens
2670	glial fibrillary acidic protein	GFAP	Homo sapiens
2671	growth factor, augmenter of liver regeneration	GFER	Homo sapiens
2672	growth factor independent 1 transcription repressor	GFI1	Homo sapiens
9945	glutamine-fructose-6-phosphate transaminase 2	GFPT2	Homo sapiens
2674	GDNF family receptor alpha 1	GFRA1	Homo sapiens
2676	GDNF family receptor alpha 3	GFRA3	Homo sapiens
23062	golgi associated, gamma adaptin ear containing, ARF binding protein 2	GGA2	Homo sapiens
2677	gamma-glutamyl carboxylase	GGCX	Homo sapiens
8836	gamma-glutamyl hydrolase (conjugase, folylpolygammaglutamyl hydrolase)	GGH	Homo sapiens
9453	geranylgeranyl diphosphate synthase 1	GGPS1	Homo sapiens
27069	growth hormone inducible transmembrane protein	GHITM	Homo sapiens
2690	growth hormone receptor	GHR	Homo sapiens
2691	growth hormone releasing hormone	GHRH	Homo sapiens
9815	G protein-coupled receptor kinase interacting ArfGAP 2	GIT2	Homo sapiens
2697	gap junction protein, alpha 1, 43kDa	GJA1	Homo sapiens
2709	gap junction protein, beta 5, 31.1kDa	GJB5	Homo sapiens
10804	gap junction protein, beta 6, 30kDa	GJB6	Homo sapiens
2710	glycerol kinase 3 pseudogene; glycerol kinase	GK	Homo sapiens
2713	glycerol kinase 3 pseudogene; glycerol kinase	GK	Homo sapiens
2712	glycerol kinase 2	GK2	Homo sapiens
56287	gastrokine 1	GKN1	Homo sapiens
2717	galactosidase, alpha	GLA	Homo sapiens
2734	golgi apparatus protein 1	GLG1	Homo sapiens
2737	GLI family zinc finger 3	GLI3	Homo sapiens
11010	GLI pathogenesis-related 1	GLIPR1	Homo sapiens
2739	glyoxalase I	GLO1	Homo sapiens
8001	glycine receptor, alpha 3	GLRA3	Homo sapiens
2743	glycine receptor, beta	GLRB	Homo sapiens
2744	glutaminase	GLS	Homo sapiens
27165	glutaminase 2 (liver, mitochondrial)	GLS2	Homo sapiens
2746	glutamate dehydrogenase 1	GLUD1	Homo sapiens
2752	glutamate-ammonia ligase (glutamine synthetase)	GLUL	Homo sapiens
10249	glycine-N-acyltransferase	GLYAT	Homo sapiens
2760	GM2 ganglioside activator	GM2A	Homo sapiens
2762	GDP-mannose 4,6-dehydratase	GMDS	Homo sapiens
2764	glia maturation factor, beta	GMFB	Homo sapiens
51053	geminin, DNA replication inhibitor	GMNN	Homo sapiens
8833	guanine monphosphate synthetase	GMPS	Homo sapiens
2768	guanine nucleotide binding protein (G protein) alpha 12	GNA12	Homo sapiens
10672	guanine nucleotide binding protein (G protein), alpha 13	GNA13	Homo sapiens
2770	guanine nucleotide binding protein (G protein), alpha inhibiting activity polypeptide 1	GNAI1	Homo sapiens
2771	guanine nucleotide binding protein (G protein), alpha inhibiting activity polypeptide 2	GNAI2	Homo sapiens

2773	guanine nucleotide binding protein (G protein), alpha inhibiting activity polypeptide 3	GNAI3	Homo sapiens
2774	guanine nucleotide binding protein (G protein), alpha activating activity polypeptide, olfactory type	GNAL	Homo sapiens
2775	guanine nucleotide binding protein (G protein), alpha activating activity polypeptide O	GNAO1	Homo sapiens
2776	guanine nucleotide binding protein (G protein), q polypeptide	GNAQ	Homo sapiens
2778	GNAS complex locus	GNAS	Homo sapiens
2781	guanine nucleotide binding protein (G protein), alpha z polypeptide	GNAZ	Homo sapiens
2783	guanine nucleotide binding protein (G protein), beta polypeptide 2	GNB2	Homo sapiens
10681	guanine nucleotide binding protein (G protein), beta 5	GNB5	Homo sapiens
2791	guanine nucleotide binding protein (G protein), gamma 11	GNG11	Homo sapiens
2788	guanine nucleotide binding protein (G protein), gamma 7	GNG7	Homo sapiens
10578	granulysin	GNLY	Homo sapiens
8443	glyceronephosphate O-acyltransferase	GNPAT	Homo sapiens
10007	glucosamine-6-phosphate deaminase 1	GNPDA1	Homo sapiens
2796	gonadotropin-releasing hormone 1 (luteinizing-releasing hormone)	GNRH1	Homo sapiens
2799	glucosamine (N-acetyl)-6-sulfatase	GNS	Homo sapiens
2800	golgi autoantigen, golgin subfamily a, 1	GOLGA1	Homo sapiens
2801	golgi autoantigen, golgin subfamily a, 2	GOLGA2	Homo sapiens
2802	golgi autoantigen, golgin subfamily a, 3	GOLGA3	Homo sapiens
2804	golgin B1, golgi integral membrane protein	GOLGB1	Homo sapiens
9527	golgi SNAP receptor complex member 1	GOSR1	Homo sapiens
9570	golgi SNAP receptor complex member 2	GOSR2	Homo sapiens
2805	glutamic-oxaloacetic transaminase 1, soluble (aspartate aminotransferase 1)	GOT1	Homo sapiens
2806	glutamic-oxaloacetic transaminase 2, mitochondrial (aspartate aminotransferase 2)	GOT2	Homo sapiens
2815	glycoprotein IX (platelet)	GP9	Homo sapiens
10223	glycoprotein A33 (transmembrane)	GPA33	Homo sapiens
8733	glycosylphosphatidylinositol anchor attachment protein 1 homolog (yeast)	GPAA1	Homo sapiens
2719	glypican 3	GPC3	Homo sapiens
2819	glycerol-3-phosphate dehydrogenase 1 (soluble)	GPD1	Homo sapiens
2820	glycerol-3-phosphate dehydrogenase 2 (mitochondrial)	GPD2	Homo sapiens
27238	G patch domain and KOW motifs	GPKOW	Homo sapiens
2823	glycoprotein M6A	GPM6A	Homo sapiens
2824	glycoprotein M6B	GPM6B	Homo sapiens
10457	glycoprotein (transmembrane) nmb	GPNMB	Homo sapiens
4935	G protein-coupled receptor 143	GPR143	Homo sapiens
23432	G protein-coupled receptor 161	GPR161	Homo sapiens
2842	G protein-coupled receptor 19	GPR19	Homo sapiens
2827	G protein-coupled receptor 3	GPR3	Homo sapiens
2859	G protein-coupled receptor 35	GPR35	Homo sapiens
2828	G protein-coupled receptor 4	GPR4	Homo sapiens
10149	G protein-coupled receptor 64	GPR64	Homo sapiens
8477	G protein-coupled receptor 65	GPR65	Homo sapiens

8111	G protein-coupled receptor 68	GPR68	Homo sapiens
51704	G protein-coupled receptor, family C, group 5, member B	GPRC5B	Homo sapiens
63940	G-protein signaling modulator 3 (AGS3-like, C. elegans)	GPSM3	Homo sapiens
2877	glutathione peroxidase 2 (gastrointestinal)	GPX2	Homo sapiens
2878	glutathione peroxidase 3 (plasma)	GPX3	Homo sapiens
2879	glutathione peroxidase 4 (phospholipid hydroperoxidase)	GPX4	Homo sapiens
9402	GRB2-related adaptor protein 2	GRAP2	Homo sapiens
2887	growth factor receptor-bound protein 10	GRB10	Homo sapiens
2888	growth factor receptor-bound protein 14	GRB14	Homo sapiens
2886	growth factor receptor-bound protein 7	GRB7	Homo sapiens
26585	gremlin 1, cysteine knot superfamily, homolog (Xenopus laevis)	GREM1	Homo sapiens
9380	glyoxylate reductase/hydroxypyruvate reductase	GRHPR	Homo sapiens
2891	glutamate receptor, ionotropic, AMPA 2	GRIA2	Homo sapiens
2897	glutamate receptor, ionotropic, kainate 1	GRIK1	Homo sapiens
2899	glutamate receptor, ionotropic, kainate 3	GRIK3	Homo sapiens
2902	glutamate receptor, ionotropic, N-methyl D-aspartate 1	GRIN1	Homo sapiens
2905	glutamate receptor, ionotropic, N-methyl D-aspartate 2C	GRIN2C	Homo sapiens
2906	glutamate receptor, ionotropic, N-methyl D-aspartate 2D	GRIN2D	Homo sapiens
2868	G protein-coupled receptor kinase 4	GRK4	Homo sapiens
2870	G protein-coupled receptor kinase 6	GRK6	Homo sapiens
2913	glutamate receptor, metabotropic 3	GRM3	Homo sapiens
2896	granulin	GRN	Homo sapiens
2922	gastrin-releasing peptide	GRP	Homo sapiens
2926	G-rich RNA sequence binding factor 1	GRSF1	Homo sapiens
2932	glycogen synthase kinase 3 beta	GSK3B	Homo sapiens
2935	G1 to S phase transition 1	GSPT1	Homo sapiens
2936	glutathione reductase	GSR	Homo sapiens
2937	glutathione synthetase	GSS	Homo sapiens
2941	glutathione S-transferase alpha 4	GSTA4	Homo sapiens
2947	glutathione S-transferase mu 3 (brain)	GSTM3	Homo sapiens
2948	glutathione S-transferase mu 4	GSTM4	Homo sapiens
2949	glutathione S-transferase mu 5	GSTM5	Homo sapiens
9446	glutathione S-transferase omega 1	GSTO1	Homo sapiens
2950	glutathione S-transferase pi 1	GSTP1	Homo sapiens
2952	glutathione S-transferase theta 1	GSTT1	Homo sapiens
2954	glutathione transferase zeta 1	GSTZ1	Homo sapiens
2957	general transcription factor IIA, 1, 19/37kDa	GTF2A1	Homo sapiens
2958	general transcription factor IIA, 2, 12kDa	GTF2A2	Homo sapiens
2959	general transcription factor IIB	GTF2B	Homo sapiens
2960	general transcription factor IIE, polypeptide 1, alpha 56kDa	GTF2E1	Homo sapiens
2961	general transcription factor IIE, polypeptide 2, beta 34kDa	GTF2E2	Homo sapiens
2962	general transcription factor IIF, polypeptide 1, 74kDa	GTF2F1	Homo sapiens
2965	general transcription factor IIH, polypeptide 1, 62kDa	GTF2H1	Homo sapiens
2968	general transcription factor IIH, polypeptide 4, 52kDa	GTF2H4	Homo sapiens
9567	GTP binding protein 1	GTPBP1	Homo sapiens
23560	GTP binding protein 4	GTPBP4	Homo sapiens

51512	G-2 and S-phase expressed 1	GTSE1	Homo sapiens
2978	guanylate cyclase activator 1A (retina)	GUCA1A	Homo sapiens
2981	guanylate cyclase activator 2B (uroguanylin)	GUCA2B	Homo sapiens
2983	guanylate cyclase 1, soluble, beta 3	GUCY1B3	Homo sapiens
2984	guanylate cyclase 2C (heat stable enterotoxin receptor)	GUCY2C	Homo sapiens
2987	guanylate kinase 1	GUK1	Homo sapiens
2990	glucuronidase, beta	GUSB	Homo sapiens
8908	glycogenin 2	GYG2	Homo sapiens
2997	glycogen synthase 1 (muscle)	GYS1	Homo sapiens
2998	glycogen synthase 2 (liver)	GYS2	Homo sapiens
3001	granzyme A (granzyme 1, cytotoxic T-lymphocyte-associated serine esterase 3)	GZMA	Homo sapiens
3002	granzyme B (granzyme 2, cytotoxic T-lymphocyte-associated serine esterase 1)	GZMB	Homo sapiens
2999	granzyme H (cathepsin G-like 2, protein h-CCPX)	GZMH	Homo sapiens
3003	granzyme K (granzyme 3; tryptase II)	GZMK	Homo sapiens
3004	granzyme M (lymphocyte met-ase 1)	GZMM	Homo sapiens
3005	H1 histone family, member 0	H1F0	Homo sapiens
9555	H2A histone family, member Y	H2AFY	Homo sapiens
3015	H2A histone family, member Z	H2AFZ	Homo sapiens
23498	3-hydroxyanthranilate 3,4-dioxygenase	HAAO	Homo sapiens
22927	hyaluronan binding protein 4	HABP4	Homo sapiens
3030	hydroxyacyl-Coenzyme A dehydrogenase/3-ketoacyl-Coenzyme A thiolase/enoyl-Coenzyme A hydratase (trifunctional protein), alpha subunit	HADHA	Homo sapiens
3029	hydroxyacylglutathione hydrolase	HAGH	Homo sapiens
3034	histidine ammonia-lyase	HAL	Homo sapiens
54363	hydroxyacid oxidase (glycolate oxidase) 1	HAO1	Homo sapiens
51179	hydroxyacid oxidase 2 (long chain)	HAO2	Homo sapiens
8520	histone acetyltransferase 1	HAT1	Homo sapiens
3043	hemoglobin, beta	HBB	Homo sapiens
10767	HBS1-like (S. cerevisiae)	HBS1L	Homo sapiens
10542	hepatitis B virus x interacting protein	HBXIP	Homo sapiens
3052	holocytochrome c synthase (cytochrome c heme-lyase)	HCCS	Homo sapiens
3054	host cell factor C1 (VP16-accessory protein)	HCFC1	Homo sapiens
3055	hemopoietic cell kinase	HCK	Homo sapiens
3059	hematopoietic cell-specific Lyn substrate 1	HCLS1	Homo sapiens
610	hyperpolarization activated cyclic nucleotide-gated potassium channel 2	HCN2	Homo sapiens
3060	hypocretin (orexin) neuropeptide precursor	HCRT	Homo sapiens
3061	hypocretin (orexin) receptor 1	HCRTR1	Homo sapiens
3062	hypocretin (orexin) receptor 2	HCRTR2	Homo sapiens
3065	histone deacetylase 1	HDAC1	Homo sapiens
3066	histone deacetylase 2	HDAC2	Homo sapiens
8841	histone deacetylase 3	HDAC3	Homo sapiens
9759	histone deacetylase 4	HDAC4	Homo sapiens
10014	histone deacetylase 5	HDAC5	Homo sapiens

10013	histone deacetylase 6	HDAC6	Homo sapiens
8226	haloacid dehalogenase-like hydrolase domain containing 1A	HDHD1A	Homo sapiens
3069	high density lipoprotein binding protein	HDLBP	Homo sapiens
3070	helicase, lymphoid-specific	HELLS	Homo sapiens
3280	hairy and enhancer of split 1, (Drosophila)	HES1	Homo sapiens
8820	HESX homeobox 1	HESX1	Homo sapiens
3073	hexosaminidase A (alpha polypeptide)	HEXA	Homo sapiens
3074	hexosaminidase B (beta polypeptide)	HEXB	Homo sapiens
23462	hairy/enhancer-of-split related with YRPW motif 1	HEY1	Homo sapiens
3077	hemochromatosis	HFE	Homo sapiens
9146	hepatocyte growth factor-regulated tyrosine kinase substrate	HGS	Homo sapiens
3087	hematopoietically expressed homeobox	HHEX	Homo sapiens
26275	3-hydroxyisobutyryl-Coenzyme A hydrolase	HIBCH	Homo sapiens
3090	hypermethylated in cancer 1	HIC1	Homo sapiens
3091	hypoxia inducible factor 1, alpha subunit (basic helix-loop-helix transcription factor)	HIF1A	Homo sapiens
3092	huntingtin interacting protein 1	HIP1	Homo sapiens
28996	homeodomain interacting protein kinase 2; similar to homeodomain interacting protein kinase 2	HIPK2	Homo sapiens
653052	homeodomain interacting protein kinase 2; similar to homeodomain interacting protein kinase 2	HIPK2	Homo sapiens
10114	homeodomain interacting protein kinase 3	HIPK3	Homo sapiens
7290	HIR histone cell cycle regulation defective homolog A (S. cerevisiae)	HIRA	Homo sapiens
8479	HIRA interacting protein 3	HIRIP3	Homo sapiens
3024	histone cluster 1, H1a	HIST1H1A	Homo sapiens
3006	histone cluster 1, H1c	HIST1H1C	Homo sapiens
3096	human immunodeficiency virus type I enhancer binding protein 1	HIVEP1	Homo sapiens
3097	human immunodeficiency virus type I enhancer binding protein 2	HIVEP2	Homo sapiens
3098	hexokinase 1	HK1	Homo sapiens
642546	hexokinase 2 pseudogene; hexokinase 2	HK2	Homo sapiens
3099	hexokinase 2 pseudogene; hexokinase 2	HK2	Homo sapiens
3111	major histocompatibility complex, class II, DO alpha	HLA-DOA	Homo sapiens
3112	major histocompatibility complex, class II, DO beta	HLA-DOB	Homo sapiens
3113	major histocompatibility complex, class II, DP alpha 1	HLA-DPA1	Homo sapiens
3115	major histocompatibility complex, class II, DP beta 1	HLA-DPB1	Homo sapiens
3135	major histocompatibility complex, class I, G	HLA-G	Homo sapiens
3141	holocarboxylase synthetase (biotin-(proprionyl-Coenzyme A-carboxylase (ATP-hydrolysing)) ligase)	HLCS	Homo sapiens
3131	hepatic leukemia factor	HLF	Homo sapiens
10362	high-mobility group 20B	HMG20B	Homo sapiens
100130009	hypothetical LOC100130009; high mobility group AT-hook 1	HMGA1	Homo sapiens
3159	hypothetical LOC100130009; high mobility group AT-hook 1	HMGA1	Homo sapiens
100130561	high-mobility group box 1; high-mobility group box 1-like 10	HMGB1	Homo sapiens
3146	high-mobility group box 1; high-mobility group box 1-like 10	HMGB1	Homo sapiens
3148	high-mobility group box 2	HMGB2	Homo sapiens
729595	similar to high mobility group box 3; high-mobility group box 3	HMGB3	Homo sapiens
646993	similar to high mobility group box 3; high-mobility group box 3	HMGB3	Homo sapiens

3149	similar to high mobility group box 3; high-mobility group box 3	HMGB3	Homo sapiens
729952	similar to high mobility group box 3; high-mobility group box 3	HMGB3	Homo sapiens
3155	3-hydroxymethyl-3-methylglutaryl-Coenzyme A lyase	HMGCL	Homo sapiens
3156	3-hydroxy-3-methylglutaryl-Coenzyme A reductase	HMGCR	Homo sapiens
3158	3-hydroxy-3-methylglutaryl-Coenzyme A synthase 2 (mitochondrial)	HMGCS2	Homo sapiens
10473	high mobility group nucleosomal binding domain 4	HMGN4	Homo sapiens
3162	heme oxygenase (decycling) 1	HMOX1	Homo sapiens
3163	heme oxygenase (decycling) 2	HMOX2	Homo sapiens
3174	hepatocyte nuclear factor 4, gamma	HNF4G	Homo sapiens
3176	histamine N-methyltransferase	HNMT	Homo sapiens
9456	homer homolog 1 (Drosophila)	HOMER1	Homo sapiens
9455	homer homolog 2 (Drosophila)	HOMER2	Homo sapiens
9454	homer homolog 3 (Drosophila)	HOMER3	Homo sapiens
3198	homeobox A1	HOXA1	Homo sapiens
3199	homeobox A2	HOXA2	Homo sapiens
3200	homeobox A3	HOXA3	Homo sapiens
3202	homeobox A5	HOXA5	Homo sapiens
3205	homeobox A9	HOXA9	Homo sapiens
3212	homeobox B2	HOXB2	Homo sapiens
3215	homeobox B5	HOXB5	Homo sapiens
3217	homeobox B7	HOXB7	Homo sapiens
3226	homeobox C10	HOXC10	Homo sapiens
3235	homeobox D9	HOXD9	Homo sapiens
3242	4-hydroxyphenylpyruvate dioxygenase	HPD	Homo sapiens
3248	hydroxyprostaglandin dehydrogenase 15-(NAD)	HPGD	Homo sapiens
3249	hepsin	HPN	Homo sapiens
3251	hypoxanthine phosphoribosyltransferase 1	HPRT1	Homo sapiens
3257	Hermansky-Pudlak syndrome 1	HPS1	Homo sapiens
11234	Hermansky-Pudlak syndrome 5	HPS5	Homo sapiens
3263	hemopexin	HPX	Homo sapiens
3265	v-Ha-ras Harvey rat sarcoma viral oncogene homolog	HRAS	Homo sapiens
3273	histidine-rich glycoprotein	HRG	Homo sapiens
3269	histamine receptor H1	HRH1	Homo sapiens
8739	harakiri, BCL2 interacting protein (contains only BH3 domain)	HRK	Homo sapiens
10247	heat-responsive protein 12	HRSP12	Homo sapiens
9653	heparan sulfate 2-O-sulfotransferase 1	HS2ST1	Homo sapiens
9957	heparan sulfate (glucosamine) 3-O-sulfotransferase 1	HS3ST1	Homo sapiens
3290	hydroxysteroid (11-beta) dehydrogenase 1	HSD11B1	Homo sapiens
3292	hydroxysteroid (17-beta) dehydrogenase 1	HSD17B1	Homo sapiens
3294	hydroxysteroid (17-beta) dehydrogenase 2	HSD17B2	Homo sapiens
3293	hydroxysteroid (17-beta) dehydrogenase 3	HSD17B3	Homo sapiens
7923	hydroxysteroid (17-beta) dehydrogenase 8	HSD17B8	Homo sapiens
3283	hydroxy-delta-5-steroid dehydrogenase, 3 beta- and steroid delta-isomerase 1	HSD3B1	Homo sapiens
3298	heat shock transcription factor 2	HSF2	Homo sapiens
11077	heat shock transcription factor 2 binding protein	HSF2BP	Homo sapiens

3305	heat shock 70kDa protein 1-like	HSPA1L	Homo sapiens
3308	heat shock 70kDa protein 4	HSPA4	Homo sapiens
400750	hypothetical gene supported by AF216292; NM_005347; heat shock 70kDa protein 5 (glucose-regulated protein, 78kDa)	HSPA5	Homo sapiens
3309	hypothetical gene supported by AF216292; NM_005347; heat shock 70kDa protein 5 (glucose-regulated protein, 78kDa)	HSPA5	Homo sapiens
653553	heat shock 27kDa protein-like 2 pseudogene; heat shock 27kDa protein 1	HSPB1	Homo sapiens
3315	heat shock 27kDa protein-like 2 pseudogene; heat shock 27kDa protein 1	HSPB1	Homo sapiens
8988	heat shock 27kDa protein 3	HSPB3	Homo sapiens
644745	heat shock 60kDa protein 1 (chaperonin) pseudogene 5; heat shock 60kDa protein 1 (chaperonin) pseudogene 6; heat shock 60kDa protein 1 (chaperonin) pseudogene 1; heat shock 60kDa protein 1 (chaperonin) pseudogene 4; heat shock 60kDa protein 1 (chaperonin)	HSPD1	Homo sapiens
345041	heat shock 60kDa protein 1 (chaperonin) pseudogene 5; heat shock 60kDa protein 1 (chaperonin) pseudogene 6; heat shock 60kDa protein 1 (chaperonin) pseudogene 1; heat shock 60kDa protein 1 (chaperonin) pseudogene 4; heat shock 60kDa protein 1 (chaperonin)	HSPD1	Homo sapiens
645548	heat shock 60kDa protein 1 (chaperonin) pseudogene 5; heat shock 60kDa protein 1 (chaperonin) pseudogene 6; heat shock 60kDa protein 1 (chaperonin) pseudogene 1; heat shock 60kDa protein 1 (chaperonin) pseudogene 4; heat shock 60kDa protein 1 (chaperonin)	HSPD1	Homo sapiens
643300	heat shock 60kDa protein 1 (chaperonin) pseudogene 5; heat shock 60kDa protein 1 (chaperonin) pseudogene 6; heat shock 60kDa protein 1 (chaperonin) pseudogene 1; heat shock 60kDa protein 1 (chaperonin) pseudogene 4; heat shock 60kDa protein 1 (chaperonin)	HSPD1	Homo sapiens
3329	heat shock 60kDa protein 1 (chaperonin) pseudogene 5; heat shock 60kDa protein 1 (chaperonin) pseudogene 6; heat shock 60kDa protein 1 (chaperonin) pseudogene 1; heat shock 60kDa protein 1 (chaperonin) pseudogene 4; heat shock 60kDa protein 1 (chaperonin)	HSPD1	Homo sapiens
3339	heparan sulfate proteoglycan 2	HSPG2	Homo sapiens
10553	HIV-1 Tat interactive protein 2, 30kDa	HTATIP2	Homo sapiens
3356	5-hydroxytryptamine (serotonin) receptor 2A	HTR2A	Homo sapiens
3357	5-hydroxytryptamine (serotonin) receptor 2B	HTR2B	Homo sapiens
3358	5-hydroxytryptamine (serotonin) receptor 2C	HTR2C	Homo sapiens
3359	5-hydroxytryptamine (serotonin) receptor 3A	HTR3A	Homo sapiens
3362	5-hydroxytryptamine (serotonin) receptor 6	HTR6	Homo sapiens
3364	HUS1 checkpoint homolog (S. pombe)	HUS1	Homo sapiens
3373	hyaluronoglucosaminidase 1	HYAL1	Homo sapiens
8692	hyaluronoglucosaminidase 2	HYAL2	Homo sapiens
10525	hypoxia up-regulated 1	HYOU1	Homo sapiens
3376	isoleucyl-tRNA synthetase	IARS	Homo sapiens
25998	inhibitor of Bruton agammaglobulinemia tyrosine kinase	IBTK	Homo sapiens
3382	islet cell autoantigen 1, 69kDa	ICA1	Homo sapiens
3385	intercellular adhesion molecule 3	ICAM3	Homo sapiens
7087	intercellular adhesion molecule 5, telencephalin	ICAM5	Homo sapiens
23463	isoprenylcysteine carboxyl methyltransferase	ICMT	Homo sapiens
29851	inducible T-cell co-stimulator	ICOS	Homo sapiens

3397	inhibitor of DNA binding 1, dominant negative helix-loop-helix protein	ID1	Homo sapiens
3399	inhibitor of DNA binding 3, dominant negative helix-loop-helix protein	ID3	Homo sapiens
3400	inhibitor of DNA binding 4, dominant negative helix-loop-helix protein	ID4	Homo sapiens
3416	insulin-degrading enzyme	IDE	Homo sapiens
3417	isocitrate dehydrogenase 1 (NADP+), soluble	IDH1	Homo sapiens
3418	isocitrate dehydrogenase 2 (NADP+), mitochondrial	IDH2	Homo sapiens
3419	isocitrate dehydrogenase 3 (NAD+) alpha	IDH3A	Homo sapiens
3421	isocitrate dehydrogenase 3 (NAD+) gamma	IDH3G	Homo sapiens
3423	iduronate 2-sulfatase	IDS	Homo sapiens
3425	iduronidase, alpha-L-	IDUA	Homo sapiens
8870	immediate early response 3	IER3	Homo sapiens
10437	interferon, gamma-inducible protein 30	IFI30	Homo sapiens
64135	interferon induced with helicase C domain 1	IFIH1	Homo sapiens
24138	interferon-induced protein with tetratricopeptide repeats 5	IFIT5	Homo sapiens
8519	interferon induced transmembrane protein 1 (9-27)	IFITM1	Homo sapiens
3454	interferon (alpha, beta and omega) receptor 1	IFNAR1	Homo sapiens
3458	interferon, gamma	IFNG	Homo sapiens
3459	interferon gamma receptor 1	IFNGR1	Homo sapiens
3460	interferon gamma receptor 2 (interferon gamma transducer 1)	IFNGR2	Homo sapiens
3475	interferon-related developmental regulator 1	IFRD1	Homo sapiens
7866	interferon-related developmental regulator 2	IFRD2	Homo sapiens
280655	chromosome 14 open reading frame 19; immunoglobulin (CD79A) binding protein 1	IGBP1	Homo sapiens
3476	chromosome 14 open reading frame 19; immunoglobulin (CD79A) binding protein 1	IGBP1	Homo sapiens
3479	insulin-like growth factor 1 (somatomedin C)	IGF1	Homo sapiens
3482	insulin-like growth factor 2 receptor	IGF2R	Homo sapiens
3484	insulin-like growth factor binding protein 1	IGFBP1	Homo sapiens
3486	insulin-like growth factor binding protein 3	IGFBP3	Homo sapiens
3488	insulin-like growth factor binding protein 5	IGFBP5	Homo sapiens
3489	insulin-like growth factor binding protein 6	IGFBP6	Homo sapiens
3490	insulin-like growth factor binding protein 7	IGFBP7	Homo sapiens
3500	immunoglobulin heavy constant gamma 1 (G1m marker); immunoglobulin heavy constant mu; immunoglobulin heavy variable 3-7; immunoglobulin heavy constant gamma 3 (G3m marker); immunoglobulin heavy variable 3-11 (gene/pseudogene); immunoglobulin heavy variable 4-31; immunoglobulin heavy locus	IGHM	Homo sapiens
3492	immunoglobulin heavy constant gamma 1 (G1m marker); immunoglobulin heavy constant mu; immunoglobulin heavy variable 3-7; immunoglobulin heavy constant gamma 3 (G3m marker); immunoglobulin heavy variable 3-11 (gene/pseudogene); immunoglobulin heavy variable 4-31; immunoglobulin heavy locus	IGHM	Homo sapiens
28396	immunoglobulin heavy constant gamma 1 (G1m marker); immunoglobulin heavy constant mu; immunoglobulin heavy variable 3-7; immunoglobulin heavy constant gamma 3 (G3m marker); immunoglobulin heavy variable 3-11 (gene/pseudogene);	IGHM	Homo sapiens

	immunoglobulin heavy variable 4-31; immunoglobulin heavy locus		
3502	immunoglobulin heavy constant gamma 1 (G1m marker); immunoglobulin heavy constant mu; immunoglobulin heavy variable 3-7; immunoglobulin heavy constant gamma 3 (G3m marker); immunoglobulin heavy variable 3-11 (gene/pseudogene); immunoglobulin heavy variable 4-31; immunoglobulin heavy locus	IGHM	Homo sapiens
3507	immunoglobulin heavy constant gamma 1 (G1m marker); immunoglobulin heavy constant mu; immunoglobulin heavy variable 3-7; immunoglobulin heavy constant gamma 3 (G3m marker); immunoglobulin heavy variable 3-11 (gene/pseudogene); immunoglobulin heavy variable 4-31; immunoglobulin heavy locus	IGHM	Homo sapiens
28450	immunoglobulin heavy constant gamma 1 (G1m marker); immunoglobulin heavy constant mu; immunoglobulin heavy variable 3-7; immunoglobulin heavy constant gamma 3 (G3m marker); immunoglobulin heavy variable 3-11 (gene/pseudogene); immunoglobulin heavy variable 4-31; immunoglobulin heavy locus	IGHM	Homo sapiens
28452	immunoglobulin heavy constant gamma 1 (G1m marker); immunoglobulin heavy constant mu; immunoglobulin heavy variable 3-7; immunoglobulin heavy constant gamma 3 (G3m marker); immunoglobulin heavy variable 3-11 (gene/pseudogene); immunoglobulin heavy variable 4-31; immunoglobulin heavy locus	IGHM	Homo sapiens
3508	immunoglobulin mu binding protein 2	IGHMBP2	Homo sapiens
3543	immunoglobulin lambda-like polypeptide 1	IGLL1	Homo sapiens
3321	immunoglobulin superfamily, member 3	IGSF3	Homo sapiens
644456	similar to CG18005; IK cytokine, down-regulator of HLA II	IK	Homo sapiens
3550	similar to CG18005; IK cytokine, down-regulator of HLA II	IK	Homo sapiens
8518	inhibitor of kappa light polypeptide gene enhancer in B-cells, kinase complex-associated protein	IKBKAP	Homo sapiens
3551	inhibitor of kappa light polypeptide gene enhancer in B-cells, kinase beta	IKBKB	Homo sapiens
3588	interleukin 10 receptor, beta	IL10RB	Homo sapiens
3589	interleukin 11	IL11	Homo sapiens
3590	interleukin 11 receptor, alpha	IL11RA	Homo sapiens
3592	interleukin 12A (natural killer cell stimulatory factor 1, cytotoxic lymphocyte maturation factor 1, p35)	IL12A	Homo sapiens
3595	interleukin 12 receptor, beta 2	IL12RB2	Homo sapiens
3596	interleukin 13	IL13	Homo sapiens
3598	interleukin 13 receptor, alpha 2	IL13RA2	Homo sapiens
3600	interleukin 15	IL15	Homo sapiens
3601	interleukin 15 receptor, alpha	IL15RA	Homo sapiens
3603	interleukin 16 (lymphocyte chemoattractant factor)	IL16	Homo sapiens
3606	interleukin 18 (interferon-gamma-inducing factor)	IL18	Homo sapiens
8809	interleukin 18 receptor 1	IL18R1	Homo sapiens
8807	interleukin 18 receptor accessory protein	IL18RAP	Homo sapiens
29949	interleukin 19	IL19	Homo sapiens
3553	interleukin 1, beta	IL1B	Homo sapiens
3554	interleukin 1 receptor, type I	IL1R1	Homo sapiens
7850	interleukin 1 receptor, type II	IL1R2	Homo sapiens
3556	interleukin 1 receptor accessory protein	IL1RAP	Homo sapiens

9173	interleukin 1 receptor-like 1	IL1RL1	Homo sapiens
3557	interleukin 1 receptor antagonist	IL1RN	Homo sapiens
51561	interleukin 23, alpha subunit p19	IL23A	Homo sapiens
9466	interleukin 27 receptor, alpha	IL27RA	Homo sapiens
3559	interleukin 2 receptor, alpha	IL2RA	Homo sapiens
3560	interleukin 2 receptor, beta	IL2RB	Homo sapiens
3561	interleukin 2 receptor, gamma (severe combined immunodeficiency)	IL2RG	Homo sapiens
133396	interleukin 31 receptor A	IL31RA	Homo sapiens
3566	interleukin 4 receptor	IL4R	Homo sapiens
3569	interleukin 6 (interferon, beta 2)	IL6	Homo sapiens
3570	interleukin 6 receptor	IL6R	Homo sapiens
3572	interleukin 6 signal transducer (gp130, oncostatin M receptor)	IL6ST	Homo sapiens
3574	interleukin 7	IL7	Homo sapiens
3575	interleukin 7 receptor	IL7R	Homo sapiens
3608	interleukin enhancer binding factor 2, 45kDa	ILF2	Homo sapiens
10994	ilvB (bacterial acetolactate synthase)-like	ILVBL	Homo sapiens
10989	inner membrane protein, mitochondrial (mitofilin)	IMMT	Homo sapiens
3612	inositol(myo)-1(or 4)-monophosphatase 1	IMPA1	Homo sapiens
3613	inositol(myo)-1(or 4)-monophosphatase 2	IMPA2	Homo sapiens
3614	IMP (inosine monophosphate) dehydrogenase 1	IMPDH1	Homo sapiens
3615	IMP (inosine monophosphate) dehydrogenase 2	IMPDH2	Homo sapiens
9118	internexin neuronal intermediate filament protein, alpha	INA	Homo sapiens
54556	inhibitor of growth family, member 3	ING3	Homo sapiens
51147	inhibitor of growth family, member 4	ING4	Homo sapiens
84289	inhibitor of growth family, member 5	ING5	Homo sapiens
3625	inhibin, beta B	INHBB	Homo sapiens
3628	inositol polyphosphate-1-phosphatase	INPP1	Homo sapiens
3631	inositol polyphosphate-4-phosphatase, type I, 107kDa	INPP4A	Homo sapiens
56623	inositol polyphosphate-5-phosphatase, 72 kDa	INPP5E	Homo sapiens
3636	inositol polyphosphate phosphatase-like 1	INPPL1	Homo sapiens
3638	insulin induced gene 1	INSIG1	Homo sapiens
3641	insulin-like 4 (placenta)	INSL4	Homo sapiens
3642	insulinoma-associated 1	INSM1	Homo sapiens
3643	insulin receptor	INSR	Homo sapiens
27130	inversin	INVS	Homo sapiens
9670	importin 13	IPO13	Homo sapiens
9657	IQ motif containing B1	IQCB1	Homo sapiens
23288	IQ motif containing E	IQCE	Homo sapiens
8826	IQ motif containing GTPase activating protein 1	IQGAP1	Homo sapiens
10788	IQ motif containing GTPase activating protein 2	IQGAP2	Homo sapiens
3654	interleukin-1 receptor-associated kinase 1	IRAK1	Homo sapiens
11213	interleukin-1 receptor-associated kinase 3	IRAK3	Homo sapiens
3660	interferon regulatory factor 2	IRF2	Homo sapiens
3662	interferon regulatory factor 4	IRF4	Homo sapiens
3663	interferon regulatory factor 5	IRF5	Homo sapiens
3664	interferon regulatory factor 6	IRF6	Homo sapiens

3665	interferon regulatory factor 7	IRF7	Homo sapiens
3667	insulin receptor substrate 1	IRS1	Homo sapiens
8660	insulin receptor substrate 2	IRS2	Homo sapiens
10265	iroquois homeobox 5	IRX5	Homo sapiens
3670	ISL LIM homeobox 1	ISL1	Homo sapiens
3671	immunoglobulin superfamily containing leucine-rich repeat	ISLR	Homo sapiens
83737	itchy E3 ubiquitin protein ligase homolog (mouse)	ITCH	Homo sapiens
8515	integrin, alpha 10	ITGA10	Homo sapiens
3673	integrin, alpha 2 (CD49B, alpha 2 subunit of VLA-2 receptor)	ITGA2	Homo sapiens
3674	integrin, alpha 2b (platelet glycoprotein IIb of IIb/IIIa complex, antigen CD41)	ITGA2B	Homo sapiens
3675	integrin, alpha 3 (antigen CD49C, alpha 3 subunit of VLA-3 receptor)	ITGA3	Homo sapiens
3676	integrin, alpha 4 (antigen CD49D, alpha 4 subunit of VLA-4 receptor)	ITGA4	Homo sapiens
3678	integrin, alpha 5 (fibronectin receptor, alpha polypeptide)	ITGA5	Homo sapiens
3679	integrin, alpha 7	ITGA7	Homo sapiens
8516	integrin, alpha 8	ITGA8	Homo sapiens
3680	integrin, alpha 9	ITGA9	Homo sapiens
3682	integrin, alpha E (antigen CD103, human mucosal lymphocyte antigen 1; alpha polypeptide)	ITGAE	Homo sapiens
3683	integrin, alpha L (antigen CD11A (p180), lymphocyte function-associated antigen 1; alpha polypeptide)	ITGAL	Homo sapiens
3684	integrin, alpha M (complement component 3 receptor 3 subunit)	ITGAM	Homo sapiens
3685	integrin, alpha V (vitronectin receptor, alpha polypeptide, antigen CD51)	ITGAV	Homo sapiens
3687	integrin, alpha X (complement component 3 receptor 4 subunit)	ITGAX	Homo sapiens
9270	integrin beta 1 binding protein 1	ITGB1BP1	Homo sapiens
3689	integrin, beta 2 (complement component 3 receptor 3 and 4 subunit)	ITGB2	Homo sapiens
3690	integrin, beta 3 (platelet glycoprotein IIIa, antigen CD61)	ITGB3	Homo sapiens
23421	integrin beta 3 binding protein (beta3-endonexin)	ITGB3BP	Homo sapiens
3691	integrin, beta 4	ITGB4	Homo sapiens
3693	integrin, beta 5	ITGB5	Homo sapiens
3694	integrin, beta 6	ITGB6	Homo sapiens
3695	integrin, beta 7	ITGB7	Homo sapiens
3696	integrin, beta 8	ITGB8	Homo sapiens
9358	integrin, beta-like 1 (with EGF-like repeat domains)	ITGBL1	Homo sapiens
3697	inter-alpha (globulin) inhibitor H1	ITIH1	Homo sapiens
3698	inter-alpha (globulin) inhibitor H2	ITIH2	Homo sapiens
3699	inter-alpha (globulin) inhibitor H3	ITIH3	Homo sapiens
3700	inter-alpha (globulin) inhibitor H4 (plasma Kallikrein-sensitive glycoprotein)	ITIH4	Homo sapiens
9452	integral membrane protein 2A	ITM2A	Homo sapiens
9445	integral membrane protein 2B	ITM2B	Homo sapiens
81618	integral membrane protein 2C	ITM2C	Homo sapiens
3705	inositol 1,3,4-triphosphate 5/6 kinase	ITPK1	Homo sapiens
3706	inositol 1,4,5-trisphosphate 3-kinase A	ITPKA	Homo sapiens
3708	inositol 1,4,5-triphosphate receptor, type 1	ITPR1	Homo sapiens
3710	inositol 1,4,5-triphosphate receptor, type 3	ITPR3	Homo sapiens

50618	intersectin 2	ITSN2	Homo sapiens
3712	isovaleryl Coenzyme A dehydrogenase	IVD	Homo sapiens
3713	involucrin	IVL	Homo sapiens
10625	influenza virus NS1A binding protein	IVNS1ABP	Homo sapiens
3716	Janus kinase 1	JAK1	Homo sapiens
3718	Janus kinase 3	JAK3	Homo sapiens
58494	junctional adhesion molecule 2	JAM2	Homo sapiens
83700	junctional adhesion molecule 3	JAM3	Homo sapiens
221037	jumonji domain containing 1C	JMJD1C	Homo sapiens
57338	junctophilin 3	JPH3	Homo sapiens
8690	jerky homolog-like (mouse)	JRKL	Homo sapiens
10899	jumping translocation breakpoint	JTB	Homo sapiens
84962	jub, ajuba homolog (Xenopus laevis)	JUB	Homo sapiens
3726	jun B proto-oncogene	JUNB	Homo sapiens
3728	junction plakoglobin	JUP	Homo sapiens
3730	Kallmann syndrome 1 sequence	KAL1	Homo sapiens
3735	lysyl-tRNA synthetase	KARS	Homo sapiens
11104	katanin p60 (ATPase-containing) subunit A 1	KATNA1	Homo sapiens
10300	katanin p80 (WD repeat containing) subunit B 1	KATNB1	Homo sapiens
3736	potassium voltage-gated channel, shaker-related subfamily, member 1 (episodic ataxia with myokymia)	KCNA1	Homo sapiens
3739	potassium voltage-gated channel, shaker-related subfamily, member 4	KCNA4	Homo sapiens
3741	potassium voltage-gated channel, shaker-related subfamily, member 5	KCNA5	Homo sapiens
7881	potassium voltage-gated channel, shaker-related subfamily, beta member 1	KCNAB1	Homo sapiens
3746	potassium voltage-gated channel, Shaw-related subfamily, member 1	KCNC1	Homo sapiens
3750	potassium voltage-gated channel, Shal-related subfamily, member 1	KCND1	Homo sapiens
3754	potassium voltage-gated channel, subfamily F, member 1	KCNF1	Homo sapiens
3755	potassium voltage-gated channel, subfamily G, member 1	KCNG1	Homo sapiens
3757	potassium voltage-gated channel, subfamily H (eag-related), member 2	KCNH2	Homo sapiens
3758	potassium inwardly-rectifying channel, subfamily J, member 1	KCNJ1	Homo sapiens
3766	potassium inwardly-rectifying channel, subfamily J, member 10	KCNJ10	Homo sapiens
3772	potassium inwardly-rectifying channel, subfamily J, member 15	KCNJ15	Homo sapiens
3760	potassium inwardly-rectifying channel, subfamily J, member 3	KCNJ3	Homo sapiens
3761	potassium inwardly-rectifying channel, subfamily J, member 4	KCNJ4	Homo sapiens
3764	potassium inwardly-rectifying channel, subfamily J, member 8	KCNJ8	Homo sapiens
3775	potassium channel, subfamily K, member 1	KCNK1	Homo sapiens
3778	potassium large conductance calcium-activated channel, subfamily M, alpha member 1	KCNMA1	Homo sapiens
3779	potassium large conductance calcium-activated channel, subfamily M, beta member 1	KCNMB1	Homo sapiens
3782	potassium intermediate/small conductance calcium-activated channel, subfamily N, member 3	KCNN3	Homo sapiens
3783	potassium intermediate/small conductance calcium-activated channel, subfamily N, member 4	KCNN4	Homo sapiens

3785	potassium voltage-gated channel, KQT-like subfamily, member 2	KCNQ2	Homo sapiens
3786	potassium voltage-gated channel, KQT-like subfamily, member 3	KCNQ3	Homo sapiens
3787	potassium voltage-gated channel, delayed-rectifier, subfamily S, member 1	KCNS1	Homo sapiens
3790	potassium voltage-gated channel, delayed-rectifier, subfamily S, member 3	KCNS3	Homo sapiens
10945	KDEL (Lys-Asp-Glu-Leu) endoplasmic reticulum protein retention receptor 1	KDELR1	Homo sapiens
11014	KDEL (Lys-Asp-Glu-Leu) endoplasmic reticulum protein retention receptor 2	KDELR2	Homo sapiens
3791	kinase insert domain receptor (a type III receptor tyrosine kinase)	KDR	Homo sapiens
3792	Kell blood group, metallo-endopeptidase	KEL	Homo sapiens
10657	KH domain containing, RNA binding, signal transduction associated 1	KHDRBS1	Homo sapiens
10656	KH domain containing, RNA binding, signal transduction associated 3	KHDRBS3	Homo sapiens
3795	ketohexokinase (fructokinase)	KHK	Homo sapiens
8570	KH-type splicing regulatory protein	KHSRP	Homo sapiens
23506	KIAA0240	KIAA0240	Homo sapiens
9856	KIAA0319	KIAA0319	Homo sapiens
23376	KIAA0776	KIAA0776	Homo sapiens
26128	KIAA1279	KIAA1279	Homo sapiens
23303	kinesin family member 13B	KIF13B	Homo sapiens
547	kinesin family member 1A	KIF1A	Homo sapiens
10749	kinesin family member 1C	KIF1C	Homo sapiens
3835	kinesin family member 22	KIF22	Homo sapiens
9493	kinesin family member 23	KIF23	Homo sapiens
11004	kinesin family member 2C	KIF2C	Homo sapiens
11127	kinesin family member 3A	KIF3A	Homo sapiens
3797	kinesin family member 3C	KIF3C	Homo sapiens
3799	kinesin family member 5B	KIF5B	Homo sapiens
22920	kinesin-associated protein 3	KIFAP3	Homo sapiens
3833	kinesin family member C1	KIFC1	Homo sapiens
3801	kinesin family member C3	KIFC3	Homo sapiens
22944	KIN, antigenic determinant of recA protein homolog (mouse)	KIN	Homo sapiens
652799	similar to Mast/stem cell growth factor receptor precursor (SCFR) (Proto-oncogene tyrosine-protein kinase Kit) (c-kit) (CD117 antigen); v-kit Hardy-Zuckerman 4 feline sarcoma viral oncogene homolog	KIT	Homo sapiens
653882	similar to Mast/stem cell growth factor receptor precursor (SCFR) (Proto-oncogene tyrosine-protein kinase Kit) (c-kit) (CD117 antigen); v-kit Hardy-Zuckerman 4 feline sarcoma viral oncogene homolog	KIT	Homo sapiens
3815	similar to Mast/stem cell growth factor receptor precursor (SCFR) (Proto-oncogene tyrosine-protein kinase Kit) (c-kit) (CD117 antigen); v-kit Hardy-Zuckerman 4 feline sarcoma viral oncogene homolog	KIT	Homo sapiens
4254	KIT ligand	KITLG	Homo sapiens
10661	Kruppel-like factor 1 (erythroid)	KLF1	Homo sapiens
11278	Kruppel-like factor 12	KLF12	Homo sapiens
28999	Kruppel-like factor 15	KLF15	Homo sapiens
688	Kruppel-like factor 5 (intestinal)	KLF5	Homo sapiens
8609	Kruppel-like factor 7 (ubiquitous)	KLF7	Homo sapiens

116138	kelch domain containing 3	KLHDC3	Homo sapiens
5655	kallikrein-related peptidase 10	KLK10	Homo sapiens
11012	kallikrein-related peptidase 11	KLK11	Homo sapiens
26085	kallikrein-related peptidase 13	KLK13	Homo sapiens
3817	kallikrein-related peptidase 2	KLK2	Homo sapiens
354	kallikrein-related peptidase 3	KLK3	Homo sapiens
5653	kallikrein-related peptidase 6	KLK6	Homo sapiens
5650	kallikrein-related peptidase 7	KLK7	Homo sapiens
3818	kallikrein B, plasma (Fletcher factor) 1	KLKB1	Homo sapiens
3820	killer cell lectin-like receptor subfamily B, member 1	KLRB1	Homo sapiens
10219	killer cell lectin-like receptor subfamily G, member 1	KLRG1	Homo sapiens
8564	kynurenine 3-monooxygenase (kynurenine 3-hydroxylase)	KMO	Homo sapiens
3827	kininogen 1	KNG1	Homo sapiens
9735	kinetochore associated 1	KNTC1	Homo sapiens
3839	karyopherin alpha 3 (importin alpha 4)	KPNA3	Homo sapiens
3841	karyopherin alpha 5 (importin alpha 6)	KPNA5	Homo sapiens
23633	karyopherin alpha 6 (importin alpha 7)	KPNA6	Homo sapiens
3858	keratin 10	KRT10	Homo sapiens
729682	keratin 17; keratin 17 pseudogene 3	KRT17	Homo sapiens
3872	keratin 17; keratin 17 pseudogene 3	KRT17	Homo sapiens
3875	keratin 18; keratin 18 pseudogene 26; keratin 18 pseudogene 19	KRT18	Homo sapiens
339781	keratin 18; keratin 18 pseudogene 26; keratin 18 pseudogene 19	KRT18	Homo sapiens
729634	keratin 18; keratin 18 pseudogene 26; keratin 18 pseudogene 19	KRT18	Homo sapiens
54474	keratin 20	KRT20	Homo sapiens
3851	keratin 4	KRT4	Homo sapiens
3855	keratin 7	KRT7	Homo sapiens
3895	kinectin 1 (kinesin receptor)	KTN1	Homo sapiens
8942	kynureninase (L-kynurenine hydrolase)	KYNU	Homo sapiens
3898	ladinin 1	LAD1	Homo sapiens
3902	lymphocyte-activation gene 3	LAG3	Homo sapiens
3903	leukocyte-associated immunoglobulin-like receptor 1	LAIR1	Homo sapiens
3904	leukocyte-associated immunoglobulin-like receptor 2	LAIR2	Homo sapiens
3908	laminin, alpha 2	LAMA2	Homo sapiens
3909	laminin, alpha 3	LAMA3	Homo sapiens
3910	laminin, alpha 4	LAMA4	Homo sapiens
3911	laminin, alpha 5	LAMA5	Homo sapiens
3912	laminin, beta 1	LAMB1	Homo sapiens
3913	laminin, beta 2 (laminin S)	LAMB2	Homo sapiens
3915	laminin, gamma 1 (formerly LAMB2)	LAMC1	Homo sapiens
3918	laminin, gamma 2	LAMC2	Homo sapiens
10314	LanC lantibiotic synthetase component C-like 1 (bacterial)	LANCL1	Homo sapiens
23395	leucyl-tRNA synthetase 2, mitochondrial	LARS2	Homo sapiens
3927	LIM and SH3 protein 1	LASP1	Homo sapiens
79603	LAG1 homolog, ceramide synthase 4	LASS4	Homo sapiens
253782	LAG1 homolog, ceramide synthase 6	LASS6	Homo sapiens
27040	linker for activation of T cells	LAT	Homo sapiens

3930	lamin B receptor	LBR	Homo sapiens
3931	lecithin-cholesterol acyltransferase	LCAT	Homo sapiens
3932	lymphocyte-specific protein tyrosine kinase	LCK	Homo sapiens
3934	lipocalin 2	LCN2	Homo sapiens
3937	lymphocyte cytosolic protein 2 (SH2 domain containing leukocyte protein of 76kDa)	LCP2	Homo sapiens
8861	LIM domain binding 1	LDB1	Homo sapiens
9079	LIM domain binding 2	LDB2	Homo sapiens
3939	lactate dehydrogenase A	LDHA	Homo sapiens
3945	lactate dehydrogenase B	LDHB	Homo sapiens
11061	leukocyte cell derived chemotaxin 1	LECT1	Homo sapiens
51176	lymphoid enhancer-binding factor 1	LEF1	Homo sapiens
3952	leptin	LEP	Homo sapiens
3953	leptin receptor	LEPR	Homo sapiens
64175	leucine proline-enriched proteoglycan (leprecan) 1	LEPRE1	Homo sapiens
23484	leptin receptor overlapping transcript-like 1	LEPROTL1	Homo sapiens
3955	LFNG O-fucosylpeptide 3-beta-N-acetylglucosaminyltransferase	LFNG	Homo sapiens
3956	lectin, galactoside-binding, soluble, 1	LGALS1	Homo sapiens
85329	lectin, galactoside-binding, soluble, 12	LGALS12	Homo sapiens
29124	lectin, galactoside-binding, soluble, 13	LGALS13	Homo sapiens
3958	lectin, galactoside-binding, soluble, 3	LGALS3	Homo sapiens
3959	lectin, galactoside-binding, soluble, 3 binding protein	LGALS3BP	Homo sapiens
3960	lectin, galactoside-binding, soluble, 4	LGALS4	Homo sapiens
3965	lectin, galactoside-binding, soluble, 9	LGALS9	Homo sapiens
9211	leucine-rich, glioma inactivated 1	LGI1	Homo sapiens
5641	legumain	LGMN	Homo sapiens
3972	luteinizing hormone beta polypeptide	LHB	Homo sapiens
3975	LIM homeobox 1	LHX1	Homo sapiens
11019	lipoic acid synthetase	LIAS	Homo sapiens
3976	leukemia inhibitory factor (cholinergic differentiation factor)	LIF	Homo sapiens
3977	leukemia inhibitory factor receptor alpha	LIFR	Homo sapiens
3978	ligase I, DNA, ATP-dependent	LIG1	Homo sapiens
3980	ligase III, DNA, ATP-dependent	LIG3	Homo sapiens
3981	ligase IV, DNA, ATP-dependent	LIG4	Homo sapiens
3984	LIM domain kinase 1	LIMK1	Homo sapiens
3987	LIM and senescent cell antigen-like domains 1	LIMS1	Homo sapiens
8825	lin-7 homolog A (C. elegans)	LIN7A	Homo sapiens
3988	lipase A, lysosomal acid, cholesterol esterase	LIPA	Homo sapiens
3990	lipase, hepatic	LIPC	Homo sapiens
3991	lipase, hormone-sensitive	LIPE	Homo sapiens
9388	lipase, endothelial	LIPG	Homo sapiens
9516	lipopolysaccharide-induced TNF factor	LITAF	Homo sapiens
3996	lethal giant larvae homolog 1 (Drosophila)	LLGL1	Homo sapiens
3998	lectin, mannose-binding, 1	LMAN1	Homo sapiens
29995	LIM and cysteine-rich domains 1	LMCD1	Homo sapiens
4000	lamin A/C	LMNA	Homo sapiens

4004	LIM domain only 1 (rhombotin 1)	LMO1	Homo sapiens
4005	LIM domain only 2 (rhombotin-like 1)	LMO2	Homo sapiens
4008	LIM domain 7	LMO7	Homo sapiens
22853	lemur tyrosine kinase 2	LMTK2	Homo sapiens
4010	LIM homeobox transcription factor 1, beta	LMX1B	Homo sapiens
4012	leucyl/cystinyl aminopeptidase	LNPEP	Homo sapiens
4015	lysyl oxidase	LOX	Homo sapiens
4017	lysyl oxidase-like 2	LOXL2	Homo sapiens
23266	latrophilin 2	LPHN2	Homo sapiens
23284	latrophilin 3	LPHN3	Homo sapiens
23175	lipin 1	LPIN1	Homo sapiens
9663	lipin 2	LPIN2	Homo sapiens
4023	lipoprotein lipase	LPL	Homo sapiens
9404	leupaxin	LPXN	Homo sapiens
987	LPS-responsive vesicle trafficking, beach and anchor containing	LRBA	Homo sapiens
23143	leucine-rich repeats and calponin homology (CH) domain containing 1	LRCH1	Homo sapiens
4033	lymphoid-restricted membrane protein	LRMP	Homo sapiens
4035	low density lipoprotein-related protein 1 (alpha-2-macroglobulin receptor)	LRP1	Homo sapiens
26020	low density lipoprotein receptor-related protein 10	LRP10	Homo sapiens
4036	low density lipoprotein-related protein 2	LRP2	Homo sapiens
4041	low density lipoprotein receptor-related protein 5	LRP5	Homo sapiens
4040	low density lipoprotein receptor-related protein 6	LRP6	Homo sapiens
7804	low density lipoprotein receptor-related protein 8, apolipoprotein e receptor	LRP8	Homo sapiens
4043	low density lipoprotein receptor-related protein associated protein 1	LRPAP1	Homo sapiens
10128	leucine-rich PPR-motif containing	LRPPRC	Homo sapiens
10234	leucine rich repeat containing 17	LRRC17	Homo sapiens
23639	leucine rich repeat containing 6	LRRC6	Homo sapiens
9208	leucine rich repeat (in FLII) interacting protein 1	LRRFIP1	Homo sapiens
4045	limbic system-associated membrane protein	LSAMP	Homo sapiens
27257	LSM1 homolog, U6 small nuclear RNA associated (S. cerevisiae)	LSM1	Homo sapiens
84967	LSM10, U7 small nuclear RNA associated	LSM10	Homo sapiens
647302	LSM3 homolog, U6 small nuclear RNA associated (S. cerevisiae); similar to Lsm3 protein	LSM3	Homo sapiens
27258	LSM3 homolog, U6 small nuclear RNA associated (S. cerevisiae); similar to Lsm3 protein	LSM3	Homo sapiens
25804	LSM4 homolog, U6 small nuclear RNA associated (S. cerevisiae)	LSM4	Homo sapiens
23658	LSM5 homolog, U6 small nuclear RNA associated (S. cerevisiae)	LSM5	Homo sapiens
11157	LSM6 homolog, U6 small nuclear RNA associated (S. cerevisiae)	LSM6	Homo sapiens
4047	lanosterol synthase (2,3-oxidosqualene-lanosterol cyclase)	LSS	Homo sapiens
7940	leukocyte specific transcript 1	LST1	Homo sapiens
4048	leukotriene A4 hydrolase	LTA4H	Homo sapiens
4050	lymphotoxin beta (TNF superfamily, member 3)	LTB	Homo sapiens
4052	latent transforming growth factor beta binding protein 1	LTBP1	Homo sapiens
4053	latent transforming growth factor beta binding protein 2	LTBP2	Homo sapiens

4055	lymphotoxin beta receptor (TNFR superfamily, member 3)	LTBR	Homo sapiens
4056	leukotriene C4 synthase	LTC4S	Homo sapiens
4057	lactotransferrin	LTF	Homo sapiens
4058	leukocyte receptor tyrosine kinase	LTK	Homo sapiens
4060	lumican	LUM	Homo sapiens
7798	leucine zipper protein 1	LUZP1	Homo sapiens
4061	lymphocyte antigen 6 complex, locus E	LY6E	Homo sapiens
80740	lymphocyte antigen 6 complex, locus G6C	LY6G6C	Homo sapiens
9936	CD302 molecule; lymphocyte antigen 75	LY75	Homo sapiens
4065	CD302 molecule; lymphocyte antigen 75	LY75	Homo sapiens
4063	lymphocyte antigen 9	LY9	Homo sapiens
23643	lymphocyte antigen 96	LY96	Homo sapiens
4067	v-yes-1 Yamaguchi sarcoma viral related oncogene homolog	LYN	Homo sapiens
653639	lysophospholipase II pseudogene 1; lysophospholipase II	LYPLA2	Homo sapiens
11313	lysophospholipase II pseudogene 1; lysophospholipase II	LYPLA2	Homo sapiens
4069	lysozyme (renal amyloidosis)	LYZ	Homo sapiens
57151	lysozyme-like 6	LYZL6	Homo sapiens
4074	mannose-6-phosphate receptor (cation dependent)	M6PR	Homo sapiens
4085	MAD2 mitotic arrest deficient-like 1 (yeast)	MAD2L1	Homo sapiens
8174	mucosal vascular addressin cell adhesion molecule 1	MADCAM1	Homo sapiens
8567	MAP-kinase activating death domain	MADD	Homo sapiens
4099	myelin associated glycoprotein	MAG	Homo sapiens
4109	melanoma antigen family A, 10	MAGEA10	Homo sapiens
4107	melanoma antigen family A, 8	MAGEA8	Homo sapiens
4113	melanoma antigen family B, 2	MAGEB2	Homo sapiens
4114	melanoma antigen family B, 3	MAGEB3	Homo sapiens
10916	melanoma antigen family D, 2	MAGED2	Homo sapiens
154043	membrane associated guanylate kinase, WW and PDZ domain containing 1; CNKSR family member 3	MAGI1	Homo sapiens
9223	membrane associated guanylate kinase, WW and PDZ domain containing 1; CNKSR family member 3	MAGI1	Homo sapiens
4118	mal, T-cell differentiation protein	MAL	Homo sapiens
10892	mucosa associated lymphoid tissue lymphoma translocation gene 1	MALT1	Homo sapiens
9794	mastermind-like 1 (Drosophila)	MAML1	Homo sapiens
84441	mastermind-like 2 (Drosophila)	MAML2	Homo sapiens
4121	mannosidase, alpha, class 1A, member 1	MAN1A1	Homo sapiens
10905	mannosidase, alpha, class 1A, member 2	MAN1A2	Homo sapiens
4124	mannosidase, alpha, class 2A, member 1	MAN2A1	Homo sapiens
4122	mannosidase, alpha, class 2A, member 2	MAN2A2	Homo sapiens
4123	mannosidase, alpha, class 2C, member 1	MAN2C1	Homo sapiens
4126	mannosidase, beta A, lysosomal	MANBA	Homo sapiens
4128	monoamine oxidase A	MAOA	Homo sapiens
4131	microtubule-associated protein 1B	MAP1B	Homo sapiens
4133	microtubule-associated protein 2	MAP2	Homo sapiens
5604	mitogen-activated protein kinase kinase 1	MAP2K1	Homo sapiens
5605	mitogen-activated protein kinase kinase 2 pseudogene; mitogen-activated protein kinase kinase 2	MAP2K2	Homo sapiens

407835	mitogen-activated protein kinase kinase 2 pseudogene; mitogen-activated protein kinase kinase 2	MAP2K2	Homo sapiens
5606	mitogen-activated protein kinase kinase 3	MAP2K3	Homo sapiens
6416	mitogen-activated protein kinase kinase 4	MAP2K4	Homo sapiens
5608	mitogen-activated protein kinase kinase 6	MAP2K6	Homo sapiens
5609	mitogen-activated protein kinase kinase 7	MAP2K7	Homo sapiens
4296	mitogen-activated protein kinase kinase kinase 11	MAP3K11	Homo sapiens
7786	mitogen-activated protein kinase kinase kinase 12	MAP3K12	Homo sapiens
9020	mitogen-activated protein kinase kinase kinase 14	MAP3K14	Homo sapiens
4216	mitogen-activated protein kinase kinase kinase 4	MAP3K4	Homo sapiens
4217	mitogen-activated protein kinase kinase kinase 5	MAP3K5	Homo sapiens
6885	mitogen-activated protein kinase kinase kinase 7	MAP3K7	Homo sapiens
1326	mitogen-activated protein kinase kinase kinase 8	MAP3K8	Homo sapiens
4134	microtubule-associated protein 4	MAP4	Homo sapiens
11184	mitogen-activated protein kinase kinase kinase kinase 1	MAP4K1	Homo sapiens
5871	mitogen-activated protein kinase kinase kinase kinase 2	MAP4K2	Homo sapiens
9448	mitogen-activated protein kinase kinase kinase kinase 4	MAP4K4	Homo sapiens
11183	mitogen-activated protein kinase kinase kinase kinase 5	MAP4K5	Homo sapiens
5602	mitogen-activated protein kinase 10	MAPK10	Homo sapiens
5600	mitogen-activated protein kinase 11	MAPK11	Homo sapiens
5603	mitogen-activated protein kinase 13	MAPK13	Homo sapiens
1432	mitogen-activated protein kinase 14	MAPK14	Homo sapiens
5595	hypothetical LOC100271831; mitogen-activated protein kinase 3	MAPK3	Homo sapiens
100271831	hypothetical LOC100271831; mitogen-activated protein kinase 3	MAPK3	Homo sapiens
5597	mitogen-activated protein kinase 6	MAPK6	Homo sapiens
5598	mitogen-activated protein kinase 7	MAPK7	Homo sapiens
5599	mitogen-activated protein kinase 8	MAPK8	Homo sapiens
9479	mitogen-activated protein kinase 8 interacting protein 1	MAPK8IP1	Homo sapiens
23162	mitogen-activated protein kinase 8 interacting protein 3	MAPK8IP3	Homo sapiens
79109	mitogen-activated protein kinase associated protein 1	MAPKAP1	Homo sapiens
9261	mitogen-activated protein kinase-activated protein kinase 2	MAPKAPK2	Homo sapiens
7867	mitogen-activated protein kinase-activated protein kinase 3	MAPKAPK3	Homo sapiens
8550	mitogen-activated protein kinase-activated protein kinase 5	MAPKAPK5	Homo sapiens
22919	microtubule-associated protein, RP/EB family, member 1	MAPRE1	Homo sapiens
10982	microtubule-associated protein, RP/EB family, member 2	MAPRE2	Homo sapiens
22924	microtubule-associated protein, RP/EB family, member 3	MAPRE3	Homo sapiens
4137	microtubule-associated protein tau	MAPT	Homo sapiens
4082	myristoylated alanine-rich protein kinase C substrate	MARCKS	Homo sapiens
8685	macrophage receptor with collagenous structure	MARCO	Homo sapiens
4140	MAP/microtubule affinity-regulating kinase 3	MARK3	Homo sapiens
4141	methionyl-tRNA synthetase	MARS	Homo sapiens
5648	mannan-binding lectin serine peptidase 1 (C4/C2 activating component of Ra-reactive factor)	MASP1	Homo sapiens
10747	mannan-binding lectin serine peptidase 2	MASP2	Homo sapiens
4143	methionine adenosyltransferase I, alpha	MAT1A	Homo sapiens
4146	matrilin 1, cartilage matrix protein	MATN1	Homo sapiens

4147	matrilin 2	MATN2	Homo sapiens
4149	MYC associated factor X	MAX	Homo sapiens
4151	myoglobin	MB	Homo sapiens
4152	methyl-CpG binding domain protein 1	MBD1	Homo sapiens
8932	methyl-CpG binding domain protein 2	MBD2	Homo sapiens
53615	methyl-CpG binding domain protein 3	MBD3	Homo sapiens
8930	methyl-CpG binding domain protein 4	MBD4	Homo sapiens
4154	muscleblind-like (Drosophila)	MBNL1	Homo sapiens
10150	muscleblind-like 2 (Drosophila)	MBNL2	Homo sapiens
4155	myelin basic protein	MBP	Homo sapiens
8720	membrane-bound transcription factor peptidase, site 1	MBTPS1	Homo sapiens
4163	mutated in colorectal cancers	MCC	Homo sapiens
4168	MCF.2 cell line derived transforming sequence	MCF2	Homo sapiens
23263	MCF.2 cell line derived transforming sequence-like	MCF2L	Homo sapiens
4170	myeloid cell leukemia sequence 1 (BCL2-related)	MCL1	Homo sapiens
4173	minichromosome maintenance complex component 4	MCM4	Homo sapiens
4174	minichromosome maintenance complex component 5	MCM5	Homo sapiens
4175	minichromosome maintenance complex component 6	MCM6	Homo sapiens
4176	minichromosome maintenance complex component 7	MCM7	Homo sapiens
10445	microspherule protein 1	MCRS1	Homo sapiens
28985	malignant T cell amplified sequence 1	MCTS1	Homo sapiens
4190	malate dehydrogenase 1, NAD (soluble)	MDH1	Homo sapiens
4191	malate dehydrogenase 2, NAD (mitochondrial)	MDH2	Homo sapiens
4192	midkine (neurite growth-promoting factor 2)	MDK	Homo sapiens
4193	Mdm2 p53 binding protein homolog (mouse)	MDM2	Homo sapiens
4194	Mdm4 p53 binding protein homolog (mouse)	MDM4	Homo sapiens
4199	malic enzyme 1, NADP(+)-dependent, cytosolic	ME1	Homo sapiens
4200	malic enzyme 2, NAD(+)-dependent, mitochondrial	ME2	Homo sapiens
10873	malic enzyme 3, NADP(+)-dependent, mitochondrial	ME3	Homo sapiens
4204	methyl CpG binding protein 2 (Rett syndrome)	MECP2	Homo sapiens
10001	mediator complex subunit 6	MED6	Homo sapiens
4205	myocyte enhancer factor 2A	MEF2A	Homo sapiens
4208	myocyte enhancer factor 2C	MEF2C	Homo sapiens
4209	myocyte enhancer factor 2D	MEF2D	Homo sapiens
4211	Meis homeobox 1	MEIS1	Homo sapiens
4212	Meis homeobox 2	MEIS2	Homo sapiens
9833	maternal embryonic leucine zipper kinase	MELK	Homo sapiens
10461	c-mer proto-oncogene tyrosine kinase	MERTK	Homo sapiens
4232	mesoderm specific transcript homolog (mouse)	MEST	Homo sapiens
4233	met proto-oncogene (hepatocyte growth factor receptor)	MET	Homo sapiens
10988	methionyl aminopeptidase 2	METAP2	Homo sapiens
4234	methyltransferase like 1	METTL1	Homo sapiens
4236	microfibrillar-associated protein 1	MFAP1	Homo sapiens
4239	microfibrillar-associated protein 4	MFAP4	Homo sapiens
4241	antigen p97 (melanoma associated) identified by monoclonal antibodies 133.2 and 96.5	MFI2	Homo sapiens

55669	mitofusin 1	MFN1	Homo sapiens
9927	mitofusin 2	MFN2	Homo sapiens
4242	MFNG O-fucosylpeptide 3-beta-N-acetylglucosaminyltransferase	MFNG	Homo sapiens
4245	mannosyl (alpha-1,3-)-glycoprotein beta-1,2-N-acetylglucosaminyltransferase	MGAT1	Homo sapiens
4247	mannosyl (alpha-1,6-)-glycoprotein beta-1,2-N-acetylglucosaminyltransferase	MGAT2	Homo sapiens
11343	monoglyceride lipase	MGLL	Homo sapiens
4255	O-6-methylguanine-DNA methyltransferase	MGMT	Homo sapiens
4256	matrix Gla protein	MGP	Homo sapiens
4258	microsomal glutathione S-transferase 2	MGST2	Homo sapiens
4259	microsomal glutathione S-transferase 3	MGST3	Homo sapiens
4276	MHC class I polypeptide-related sequence A	MICA	Homo sapiens
11043	midline 2	MID2	Homo sapiens
4282	macrophage migration inhibitory factor (glycosylation-inhibiting factor)	MIF	Homo sapiens
9562	multiple inositol polyphosphate histidine phosphatase, 1	MINPP1	Homo sapiens
4286	microphthalmia-associated transcription factor	MITF	Homo sapiens
4288	antigen identified by monoclonal antibody Ki-67	MKI67	Homo sapiens
57496	MKL/myocardin-like 2	MKL2	Homo sapiens
4289	muskelin 1, intracellular mediator containing kelch motifs	MKLN1	Homo sapiens
8569	MAP kinase interacting serine/threonine kinase 1	MKNK1	Homo sapiens
2872	MAP kinase interacting serine/threonine kinase 2	MKNK2	Homo sapiens
7681	makorin ring finger protein 3	MKRN3	Homo sapiens
2315	melan-A	MLANA	Homo sapiens
23209	megalencephalic leukoencephalopathy with subcortical cysts 1	MLC1	Homo sapiens
8079	myeloid leukemia factor 2	MLF2	Homo sapiens
4292	mutL homolog 1, colon cancer, nonpolyposis type 2 (E. coli)	MLH1	Homo sapiens
27030	mutL homolog 3 (E. coli)	MLH3	Homo sapiens
4297	myeloid/lymphoid or mixed-lineage leukemia (trithorax homolog, Drosophila)	MLL	Homo sapiens
8085	myeloid/lymphoid or mixed-lineage leukemia 2	MLL2	Homo sapiens
55904	myeloid/lymphoid or mixed-lineage leukemia 5 (trithorax homolog, Drosophila)	MLL5	Homo sapiens
8028	myeloid/lymphoid or mixed-lineage leukemia (trithorax homolog, Drosophila); translocated to, 10	MLLT10	Homo sapiens
730031	similar to Afadin (Protein AF-6); myeloid/lymphoid or mixed-lineage leukemia (trithorax homolog, Drosophila); translocated to, 4	MLLT4	Homo sapiens
4301	similar to Afadin (Protein AF-6); myeloid/lymphoid or mixed-lineage leukemia (trithorax homolog, Drosophila); translocated to, 4	MLLT4	Homo sapiens
4295	motilin	MLN	Homo sapiens
4311	membrane metallo-endopeptidase	MME	Homo sapiens
4312	matrix metallopeptidase 1 (interstitial collagenase)	MMP1	Homo sapiens
4319	matrix metallopeptidase 10 (stromelysin 2)	MMP10	Homo sapiens
4320	matrix metallopeptidase 11 (stromelysin 3)	MMP11	Homo sapiens
4321	matrix metallopeptidase 12 (macrophage elastase)	MMP12	Homo sapiens
4322	matrix metallopeptidase 13 (collagenase 3)	MMP13	Homo sapiens
4323	matrix metallopeptidase 14 (membrane-inserted)	MMP14	Homo sapiens

4324	matrix metallopeptidase 15 (membrane-inserted)	MMP15	Homo sapiens
4326	matrix metallopeptidase 17 (membrane-inserted)	MMP17	Homo sapiens
4327	matrix metallopeptidase 19	MMP19	Homo sapiens
4313	matrix metallopeptidase 2 (gelatinase A, 72kDa gelatinase, 72kDa type IV collagenase)	MMP2	Homo sapiens
79148	matrix metallopeptidase 28	MMP28	Homo sapiens
4316	matrix metallopeptidase 7 (matrilysin, uterine)	MMP7	Homo sapiens
4318	matrix metallopeptidase 9 (gelatinase B, 92kDa gelatinase, 92kDa type IV collagenase)	MMP9	Homo sapiens
4330	meningioma (disrupted in balanced translocation) 1	MN1	Homo sapiens
4331	menage a trois homolog 1, cyclin H assembly factor (Xenopus laevis)	MNAT1	Homo sapiens
4332	myeloid cell nuclear differentiation antigen	MNDA	Homo sapiens
64112	modulator of apoptosis 1	MOAP1	Homo sapiens
4336	myelin-associated oligodendrocyte basic protein	MOBP	Homo sapiens
27304	molybdenum cofactor synthesis 3	MOCS3	Homo sapiens
9643	mortality factor 4 like 2	MORF4L2	Homo sapiens
56180	motile sperm domain containing 1	MOSPD1	Homo sapiens
9526	mannose-P-dolichol utilization defect 1	MPDU1	Homo sapiens
8777	multiple PDZ domain protein	MPDZ	Homo sapiens
4350	N-methylpurine-DNA glycosylase	MPG	Homo sapiens
10200	M-phase phosphoprotein 6	MPHOSPH6	Homo sapiens
10198	M-phase phosphoprotein 9	MPHOSPH9	Homo sapiens
4351	mannose phosphate isomerase	MPI	Homo sapiens
4353	myeloperoxidase	MPO	Homo sapiens
4354	membrane protein, palmitoylated 1, 55kDa	MPP1	Homo sapiens
4355	membrane protein, palmitoylated 2 (MAGUK p55 subfamily member 2)	MPP2	Homo sapiens
4356	membrane protein, palmitoylated 3 (MAGUK p55 subfamily member 3)	MPP3	Homo sapiens
51678	membrane protein, palmitoylated 6 (MAGUK p55 subfamily member 6)	MPP6	Homo sapiens
65258	metallophosphoesterase 1	MPPE1	Homo sapiens
4357	mercaptopyruvate sulfurtransferase	MPST	Homo sapiens
4358	MpV17 mitochondrial inner membrane protein	MPV17	Homo sapiens
4359	myelin protein zero	MPZ	Homo sapiens
9019	myelin protein zero-like 1	MPZL1	Homo sapiens
3140	major histocompatibility complex, class I-related	MR1	Homo sapiens
4361	MRE11 meiotic recombination 11 homolog A (S. cerevisiae)	MRE11A	Homo sapiens
6182	mitochondrial ribosomal protein L12	MRPL12	Homo sapiens
10573	mitochondrial ribosomal protein L28	MRPL28	Homo sapiens
9553	mitochondrial ribosomal protein L33	MRPL33	Homo sapiens
64963	mitochondrial ribosomal protein S11	MRPS11	Homo sapiens
6183	mitochondrial ribosomal protein S12	MRPS12	Homo sapiens
931	membrane-spanning 4-domains, subfamily A, member 1	MS4A1	Homo sapiens
2206	membrane-spanning 4-domains, subfamily A, member 2 (Fc fragment of IgE, high affinity I, receptor for; beta polypeptide)	MS4A2	Homo sapiens
4436	mutS homolog 2, colon cancer, nonpolyposis type 1 (E. coli)	MSH2	Homo sapiens

4437	mutS homolog 3 (E. coli)	MSH3	Homo sapiens
10232	mesothelin	MSLN	Homo sapiens
4477	microseminoprotein, beta-	MSMB	Homo sapiens
4486	macrophage stimulating 1 receptor (c-met-related tyrosine kinase)	MST1R	Homo sapiens
4487	msh homeobox 1	MSX1	Homo sapiens
4507	methylthioadenosine phosphorylase	MTAP	Homo sapiens
4515	mature T-cell proliferation 1 neighbor; mature T-cell proliferation 1	MTCP1	Homo sapiens
100272147	mature T-cell proliferation 1 neighbor; mature T-cell proliferation 1	MTCP1	Homo sapiens
4522	methylenetetrahydrofolate dehydrogenase (NADP+ dependent) 1, methenyltetrahydrofolate cyclohydrolase, formyltetrahydrofolate synthetase	MTHFD1	Homo sapiens
10797	methylenetetrahydrofolate dehydrogenase (NADP+ dependent) 2, methenyltetrahydrofolate cyclohydrolase	MTHFD2	Homo sapiens
4524	5,10-methylenetetrahydrofolate reductase (NADPH)	MTHFR	Homo sapiens
4528	mitochondrial translational initiation factor 2	MTIF2	Homo sapiens
8776	myotubularin related protein 1	MTMR1	Homo sapiens
8898	myotubularin related protein 2	MTMR2	Homo sapiens
9110	myotubularin related protein 4	MTMR4	Homo sapiens
9788	metastasis suppressor 1	MTSS1	Homo sapiens
10651	metaxin 2	MTX2	Homo sapiens
4585	mucin 4, cell surface associated	MUC4	Homo sapiens
4589	mucin 7, secreted	MUC7	Homo sapiens
4593	muscle, skeletal, receptor tyrosine kinase	MUSK	Homo sapiens
4594	methylmalonyl Coenzyme A mutase	MUT	Homo sapiens
4595	mutY homolog (E. coli)	MUTYH	Homo sapiens
4597	mevalonate (diphospho) decarboxylase	MVD	Homo sapiens
4599	myxovirus (influenza virus) resistance 1, interferon-inducible protein p78 (mouse)	MX1	Homo sapiens
4600	myxovirus (influenza virus) resistance 2 (mouse)	MX2	Homo sapiens
10608	MAX dimerization protein 4	MXD4	Homo sapiens
4601	MAX interactor 1	MXI1	Homo sapiens
4602	v-myb myeloblastosis viral oncogene homolog (avian)	MYB	Homo sapiens
4605	v-myb myeloblastosis viral oncogene homolog (avian)-like 2	MYBL2	Homo sapiens
4609	v-myc myelocytomatosis viral oncogene homolog (avian)	MYC	Homo sapiens
23077	MYC binding protein 2	MYCBP2	Homo sapiens
4610	v-myc myelocytomatosis viral oncogene homolog 1, lung carcinoma derived (avian)	MYCL1	Homo sapiens
4613	v-myc myelocytomatosis viral related oncogene, neuroblastoma derived (avian)	MYCN	Homo sapiens
4615	myeloid differentiation primary response gene (88)	MYD88	Homo sapiens
50804	myelin expression factor 2	MYEF2	Homo sapiens
4628	myosin, heavy chain 10, non-muscle	MYH10	Homo sapiens
4629	myosin, heavy chain 11, smooth muscle	MYH11	Homo sapiens
4627	myosin, heavy chain 9, non-muscle	MYH9	Homo sapiens
4633	myosin, light chain 2, regulatory, cardiac, slow	MYL2	Homo sapiens
4637	myosin, light chain 6, alkali, smooth muscle and non-muscle	MYL6	Homo sapiens
29116	myosin regulatory light chain interacting protein	MYLIP	Homo sapiens

4638	myosin light chain kinase	MYLK	Homo sapiens
4651	myosin X	MYO10	Homo sapiens
4430	myosin IB	MYO1B	Homo sapiens
4642	myosin ID	MYO1D	Homo sapiens
4643	myosin IE	MYO1E	Homo sapiens
4644	myosin VA (heavy chain 12, myoxin)	MYO5A	Homo sapiens
4646	myosin VI	MYO6	Homo sapiens
4647	myosin VIIA	MYO7A	Homo sapiens
4653	myocilin, trabecular meshwork inducible glucocorticoid response	MYOC	Homo sapiens
4656	myogenin (myogenic factor 4)	MYOG	Homo sapiens
9172	myomesin (M-protein) 2, 165kDa	MYOM2	Homo sapiens
84148	MYST histone acetyltransferase 1	MYST1	Homo sapiens
7994	MYST histone acetyltransferase (monocytic leukemia) 3	MYST3	Homo sapiens
23522	MYST histone acetyltransferase (monocytic leukemia) 4	MYST4	Homo sapiens
23040	myelin transcription factor 1-like	MYT1L	Homo sapiens
4664	NGFI-A binding protein 1 (EGR1 binding protein 1)	NAB1	Homo sapiens
4665	NGFI-A binding protein 2 (EGR1 binding protein 2)	NAB2	Homo sapiens
4668	N-acetylgalactosaminidase, alpha-	NAGA	Homo sapiens
51172	N-acetylglucosamine-1-phosphodiester alpha-N-acetylglucosaminidase	NAGPA	Homo sapiens
54187	N-acetylneuraminic acid synthase	NANS	Homo sapiens
4676	nucleosome assembly protein 1-like 4	NAP1L4	Homo sapiens
8774	N-ethylmaleimide-sensitive factor attachment protein, gamma	NAPG	Homo sapiens
4677	asparaginyl-tRNA synthetase	NARS	Homo sapiens
4678	nuclear autoantigenic sperm protein (histone-binding)	NASP	Homo sapiens
10	N-acetyltransferase 2 (arylamine N-acetyltransferase)	NAT2	Homo sapiens
9027	N-acetyltransferase 8 (GCN5-related, putative); N-acetyltransferase 8B (GCN5-related, putative, gene/pseudogene)	NAT8	Homo sapiens
51471	N-acetyltransferase 8 (GCN5-related, putative); N-acetyltransferase 8B (GCN5-related, putative, gene/pseudogene)	NAT8	Homo sapiens
652725	neuron navigator 3; similar to neuron navigator 3	NAV3	Homo sapiens
89795	neuron navigator 3; similar to neuron navigator 3	NAV3	Homo sapiens
4681	neuroblastoma, suppression of tumorigenicity 1	NBL1	Homo sapiens
4684	neural cell adhesion molecule 1	NCAM1	Homo sapiens
4686	nuclear cap binding protein subunit 1, 80kDa	NCBP1	Homo sapiens
22916	nuclear cap binding protein subunit 2, 20kDa	NCBP2	Homo sapiens
4688	neutrophil cytosolic factor 2	NCF2	Homo sapiens
4689	neutrophil cytosolic factor 4, 40kDa	NCF4	Homo sapiens
4690	NCK adaptor protein 1	NCK1	Homo sapiens
8440	NCK adaptor protein 2	NCK2	Homo sapiens
51517	NCK interacting protein with SH3 domain	NCKIPSD	Homo sapiens
4691	nucleolin	NCL	Homo sapiens
8648	nuclear receptor coactivator 1	NCOA1	Homo sapiens
10499	nuclear receptor coactivator 2	NCOA2	Homo sapiens
8031	nuclear receptor coactivator 4	NCOA4	Homo sapiens
9611	nuclear receptor co-repressor 1	NCOR1	Homo sapiens
80762	Nedd4 family interacting protein 1	NDFIP1	Homo sapiens

4693	Norrie disease (pseudoglioma)	NDP	Homo sapiens
10397	N-myc downstream regulated 1	NDRG1	Homo sapiens
8509	N-deacetylase/N-sulfotransferase (heparan glucosaminyl) 2	NDST2	Homo sapiens
4694	NADH dehydrogenase (ubiquinone) 1 alpha subcomplex, 1, 7.5kDa	NDUFA1	Homo sapiens
4695	NADH dehydrogenase (ubiquinone) 1 alpha subcomplex, 2, 8kDa	NDUFA2	Homo sapiens
4696	NADH dehydrogenase (ubiquinone) 1 alpha subcomplex, 3, 9kDa	NDUFA3	Homo sapiens
4697	NADH dehydrogenase (ubiquinone) 1 alpha subcomplex, 4, 9kDa	NDUFA4	Homo sapiens
4700	NADH dehydrogenase (ubiquinone) 1 alpha subcomplex, 6, 14kDa	NDUFA6	Homo sapiens
4701	NADH dehydrogenase (ubiquinone) 1 alpha subcomplex, 7, 14.5kDa	NDUFA7	Homo sapiens
4704	NADH dehydrogenase (ubiquinone) 1 alpha subcomplex, 9, 39kDa	NDUFA9	Homo sapiens
4706	NADH dehydrogenase (ubiquinone) 1, alpha/beta subcomplex, 1, 8kDa	NDUFAB1	Homo sapiens
4707	NADH dehydrogenase (ubiquinone) 1 beta subcomplex, 1, 7kDa	NDUFB1	Homo sapiens
4716	NADH dehydrogenase (ubiquinone) 1 beta subcomplex, 10, 22kDa	NDUFB10	Homo sapiens
4708	NADH dehydrogenase (ubiquinone) 1 beta subcomplex, 2, 8kDa	NDUFB2	Homo sapiens
402175	hypothetical gene supported by AF044957; NM_004547; NADH dehydrogenase (ubiquinone) 1 beta subcomplex, 4, 15kDa	NDUFB4	Homo sapiens
4710	hypothetical gene supported by AF044957; NM_004547; NADH dehydrogenase (ubiquinone) 1 beta subcomplex, 4, 15kDa	NDUFB4	Homo sapiens
4711	NADH dehydrogenase (ubiquinone) 1 beta subcomplex, 5, 16kDa	NDUFB5	Homo sapiens
4712	NADH dehydrogenase (ubiquinone) 1 beta subcomplex, 6, 17kDa	NDUFB6	Homo sapiens
4713	NADH dehydrogenase (ubiquinone) 1 beta subcomplex, 7, 18kDa	NDUFB7	Homo sapiens
4714	NADH dehydrogenase (ubiquinone) 1 beta subcomplex, 8, 19kDa	NDUFB8	Homo sapiens
4718	NADH dehydrogenase (ubiquinone) 1, subcomplex unknown, 2, 14.5kDa	NDUFC2	Homo sapiens
4719	NADH dehydrogenase (ubiquinone) Fe-S protein 1, 75kDa (NADH-coenzyme Q reductase)	NDUFS1	Homo sapiens
4720	NADH dehydrogenase (ubiquinone) Fe-S protein 2, 49kDa (NADH-coenzyme Q reductase)	NDUFS2	Homo sapiens
4724	NADH dehydrogenase (ubiquinone) Fe-S protein 4, 18kDa (NADH-coenzyme Q reductase)	NDUFS4	Homo sapiens
4726	NADH dehydrogenase (ubiquinone) Fe-S protein 6, 13kDa (NADH-coenzyme Q reductase)	NDUFS6	Homo sapiens
4723	NADH dehydrogenase (ubiquinone) flavoprotein 1, 51kDa	NDUFV1	Homo sapiens
10529	nebulette	NEBL	Homo sapiens
4734	neural precursor cell expressed, developmentally down-regulated 4	NEDD4	Homo sapiens
100128775	neural precursor cell expressed, developmentally down-regulated 8; similar to neural precursor cell expressed, developmentally down-regulated gene 8	NEDD8	Homo sapiens
4738	neural precursor cell expressed, developmentally down-regulated 8; similar to neural precursor cell expressed, developmentally down-regulated gene 8	NEDD8	Homo sapiens
4744	neurofilament, heavy polypeptide	NEFH	Homo sapiens
4751	NIMA (never in mitosis gene a)-related kinase 2	NEK2	Homo sapiens
4752	NIMA (never in mitosis gene a)-related kinase 3	NEK3	Homo sapiens
91754	NIMA (never in mitosis gene a)- related kinase 9	NEK9	Homo sapiens
4753	NEL-like 2 (chicken)	NELL2	Homo sapiens
4756	neogenin homolog 1 (chicken)	NEO1	Homo sapiens
10763	nestin	NES	Homo sapiens

10276	neuroepithelial cell transforming 1	NET1	Homo sapiens
10825	sialidase 3 (membrane sialidase)	NEU3	Homo sapiens
9148	neuralized homolog (Drosophila)	NEURL	Homo sapiens
50674	neurogenin 3	NEUROG3	Homo sapiens
4763	neurofibromin 1	NF1	Homo sapiens
4771	neurofibromin 2 (merlin)	NF2	Homo sapiens
10725	nuclear factor of activated T-cells 5, tonicity-responsive	NFAT5	Homo sapiens
4772	nuclear factor of activated T-cells, cytoplasmic, calcineurin-dependent 1	NFATC1	Homo sapiens
4773	nuclear factor of activated T-cells, cytoplasmic, calcineurin-dependent 2	NFATC2	Homo sapiens
4775	nuclear factor of activated T-cells, cytoplasmic, calcineurin-dependent 3	NFATC3	Homo sapiens
4778	nuclear factor (erythroid-derived 2), 45kDa	NFE2	Homo sapiens
4780	nuclear factor (erythroid-derived 2)-like 2	NFE2L2	Homo sapiens
9603	nuclear factor (erythroid-derived 2)-like 3	NFE2L3	Homo sapiens
4782	nuclear factor I/C (CCAAT-binding transcription factor)	NFIC	Homo sapiens
4783	nuclear factor, interleukin 3 regulated	NFIL3	Homo sapiens
4784	nuclear factor I/X (CCAAT-binding transcription factor)	NFIX	Homo sapiens
4790	nuclear factor of kappa light polypeptide gene enhancer in B-cells 1	NFKB1	Homo sapiens
4792	nuclear factor of kappa light polypeptide gene enhancer in B-cells inhibitor, alpha	NFKBIA	Homo sapiens
4794	nuclear factor of kappa light polypeptide gene enhancer in B-cells inhibitor, epsilon	NFKBIE	Homo sapiens
4795	nuclear factor of kappa light polypeptide gene enhancer in B-cells inhibitor-like 1	NFKBIL1	Homo sapiens
4798	nuclear factor related to kappaB binding protein	NFRKB	Homo sapiens
4800	nuclear transcription factor Y, alpha	NFYA	Homo sapiens
4802	nuclear transcription factor Y, gamma	NFYC	Homo sapiens
4804	nerve growth factor receptor (TNFR superfamily, member 16)	NGFR	Homo sapiens
4807	nescient helix loop helix 1	NHLH1	Homo sapiens
4809	NHP2 non-histone chromosome protein 2-like 1 (S. cerevisiae)	NHP2L1	Homo sapiens
22795	nidogen 2 (osteonidogen)	NID2	Homo sapiens
4814	ninjurin 1	NINJ1	Homo sapiens
25836	Nipped-B homolog (Drosophila)	NIPBL	Homo sapiens
8508	nipsnap homolog 1 (C. elegans)	NIPSNAP1	Homo sapiens
11188	nischarin	NISCH	Homo sapiens
4817	nitrilase 1	NIT1	Homo sapiens
4818	natural killer cell group 7 sequence	NKG7	Homo sapiens
4820	natural killer-tumor recognition sequence	NKTR	Homo sapiens
4828	neuromedin B	NMB	Homo sapiens
654364	non-metastatic cells 1, protein (NM23A) expressed in; NME1-NME2 readthrough transcript; non-metastatic cells 2, protein (NM23B) expressed in	NME1	Homo sapiens
4830	non-metastatic cells 1, protein (NM23A) expressed in; NME1-NME2 readthrough transcript; non-metastatic cells 2, protein (NM23B) expressed in	NME1	Homo sapiens
4831	non-metastatic cells 1, protein (NM23A) expressed in; NME1-NME2	NME1	Homo sapiens

	readthrough transcript; non-metastatic cells 2, protein (NM23B) expressed in		
4832	non-metastatic cells 3, protein expressed in	NME3	Homo sapiens
4833	non-metastatic cells 4, protein expressed in	NME4	Homo sapiens
8382	non-metastatic cells 5, protein expressed in (nucleoside-diphosphate kinase)	NME5	Homo sapiens
10201	non-metastatic cells 6, protein expressed in (nucleoside-diphosphate kinase)	NME6	Homo sapiens
29922	non-metastatic cells 7, protein expressed in (nucleoside-diphosphate kinase)	NME7	Homo sapiens
9111	N-myc (and STAT) interactor	NMI	Homo sapiens
4836	N-myristoyltransferase 1	NMT1	Homo sapiens
10874	neuromedin U	NMU	Homo sapiens
4826	neuronatin	NNAT	Homo sapiens
4837	nicotinamide N-methyltransferase	NNMT	Homo sapiens
23530	nicotinamide nucleotide transhydrogenase	NNT	Homo sapiens
8715	nucleolar protein 4	NOL4	Homo sapiens
51406	nucleolar protein 7, 27kDa	NOL7	Homo sapiens
9221	nucleolar and coiled-body phosphoprotein 1	NOLC1	Homo sapiens
4841	non-POU domain containing, octamer-binding	NONO	Homo sapiens
4842	nitric oxide synthase 1 (neuronal)	NOS1	Homo sapiens
4853	Notch homolog 2 (Drosophila)	NOTCH2	Homo sapiens
4854	Notch homolog 3 (Drosophila)	NOTCH3	Homo sapiens
4855	Notch homolog 4 (Drosophila)	NOTCH4	Homo sapiens
4856	nephroblastoma overexpressed gene	NOV	Homo sapiens
50507	NADPH oxidase 4	NOX4	Homo sapiens
4862	neuronal PAS domain protein 2	NPAS2	Homo sapiens
64067	neuronal PAS domain protein 3	NPAS3	Homo sapiens
4864	Niemann-Pick disease, type C1	NPC1	Homo sapiens
8620	neuropeptide FF-amide peptide precursor	NPFF	Homo sapiens
4867	nephronophthisis 1 (juvenile)	NPHP1	Homo sapiens
4868	nephrosis 1, congenital, Finnish type (nephrin)	NPHS1	Homo sapiens
729686	nucleophosmin 1 (nucleolar phosphoprotein B23, numatrin) pseudogene 21; hypothetical LOC100131044; similar to nucleophosmin 1; nucleophosmin (nucleolar phosphoprotein B23, numatrin)	NPM1	Homo sapiens
4869	nucleophosmin 1 (nucleolar phosphoprotein B23, numatrin) pseudogene 21; hypothetical LOC100131044; similar to nucleophosmin 1; nucleophosmin (nucleolar phosphoprotein B23, numatrin)	NPM1	Homo sapiens
399804	nucleophosmin 1 (nucleolar phosphoprotein B23, numatrin) pseudogene 21; hypothetical LOC100131044; similar to nucleophosmin 1; nucleophosmin (nucleolar phosphoprotein B23, numatrin)	NPM1	Homo sapiens
100131044	nucleophosmin 1 (nucleolar phosphoprotein B23, numatrin) pseudogene 21; hypothetical LOC100131044; similar to nucleophosmin 1; nucleophosmin (nucleolar phosphoprotein B23, numatrin)	NPM1	Homo sapiens
729342	nucleophosmin 1 (nucleolar phosphoprotein B23, numatrin)	NPM1	Homo sapiens

	pseudogene 21; hypothetical LOC100131044; similar to nucleophosmin 1; nucleophosmin (nucleolar phosphoprotein B23, numatrin)		
440577	nucleophosmin 1 (nucleolar phosphoprotein B23, numatrin) pseudogene 21; hypothetical LOC100131044; similar to nucleophosmin 1; nucleophosmin (nucleolar phosphoprotein B23, numatrin)	NPM1	Homo sapiens
100129237	nucleophosmin 1 (nucleolar phosphoprotein B23, numatrin) pseudogene 21; hypothetical LOC100131044; similar to nucleophosmin 1; nucleophosmin (nucleolar phosphoprotein B23, numatrin)	NPM1	Homo sapiens
4879	natriuretic peptide precursor B	NPPB	Homo sapiens
4881	natriuretic peptide receptor A/guanylate cyclase A (atrionatriuretic peptide receptor A)	NPR1	Homo sapiens
4882	natriuretic peptide receptor B/guanylate cyclase B (atrionatriuretic peptide receptor B)	NPR2	Homo sapiens
4883	natriuretic peptide receptor C/guanylate cyclase C (atrionatriuretic peptide receptor C)	NPR3	Homo sapiens
4885	neuronal pentraxin II	NPTX2	Homo sapiens
23467	neuronal pentraxin receptor	NPTXR	Homo sapiens
4886	neuropeptide Y receptor Y1	NPY1R	Homo sapiens
1728	NAD(P)H dehydrogenase, quinone 1	NQO1	Homo sapiens
190	nuclear receptor subfamily 0, group B, member 1	NR0B1	Homo sapiens
8431	nuclear receptor subfamily 0, group B, member 2	NR0B2	Homo sapiens
9572	nuclear receptor subfamily 1, group D, member 1	NR1D1	Homo sapiens
7376	nuclear receptor subfamily 1, group H, member 2	NR1H2	Homo sapiens
10062	nuclear receptor subfamily 1, group H, member 3	NR1H3	Homo sapiens
9971	nuclear receptor subfamily 1, group H, member 4	NR1H4	Homo sapiens
8856	nuclear receptor subfamily 1, group I, member 2	NR1I2	Homo sapiens
7181	nuclear receptor subfamily 2, group C, member 1	NR2C1	Homo sapiens
7182	nuclear receptor subfamily 2, group C, member 2	NR2C2	Homo sapiens
7025	nuclear receptor subfamily 2, group F, member 1	NR2F1	Homo sapiens
2063	nuclear receptor subfamily 2, group F, member 6	NR2F6	Homo sapiens
2908	nuclear receptor subfamily 3, group C, member 1 (glucocorticoid receptor)	NR3C1	Homo sapiens
4306	nuclear receptor subfamily 3, group C, member 2	NR3C2	Homo sapiens
3164	nuclear receptor subfamily 4, group A, member 1	NR4A1	Homo sapiens
4929	nuclear receptor subfamily 4, group A, member 2	NR4A2	Homo sapiens
8013	nuclear receptor subfamily 4, group A, member 3	NR4A3	Homo sapiens
4893	neuroblastoma RAS viral (v-ras) oncogene homolog	NRAS	Homo sapiens
4897	neuronal cell adhesion molecule	NRCAM	Homo sapiens
4898	nardilysin (N-arginine dibasic convertase)	NRD1	Homo sapiens
4899	nuclear respiratory factor 1	NRF1	Homo sapiens
3084	neuregulin 1	NRG1	Homo sapiens
4900	neurogranin (protein kinase C substrate, RC3)	NRGN	Homo sapiens
4901	neural retina leucine zipper	NRL	Homo sapiens
8829	neuropilin 1	NRP1	Homo sapiens
8828	neuropilin 2	NRP2	Homo sapiens

9378	neurexin 1	NRXN1	Homo sapiens
9369	neurexin 3	NRXN3	Homo sapiens
50814	NAD(P) dependent steroid dehydrogenase-like	NSDHL	Homo sapiens
4905	N-ethylmaleimide-sensitive factor	NSF	Homo sapiens
55968	NSFL1 (p97) cofactor (p47)	NSFL1C	Homo sapiens
8439	neutral sphingomyelinase (N-SMase) activation associated factor	NSMAF	Homo sapiens
22978	5'-nucleotidase, cytosolic II	NT5C2	Homo sapiens
51251	5'-nucleotidase, cytosolic III	NT5C3	Homo sapiens
4907	5'-nucleotidase, ecto (CD73)	NT5E	Homo sapiens
4913	nth endonuclease III-like 1 (E. coli)	NTHL1	Homo sapiens
9423	netrin 1	NTN1	Homo sapiens
4914	neurotrophic tyrosine kinase, receptor, type 1	NTRK1	Homo sapiens
4915	neurotrophic tyrosine kinase, receptor, type 2	NTRK2	Homo sapiens
4922	neurotensin	NTS	Homo sapiens
23620	neurotensin receptor 2	NTSR2	Homo sapiens
4682	nucleotide binding protein 1 (MinD homolog, E. coli)	NUBP1	Homo sapiens
4925	nucleobindin 2	NUCB2	Homo sapiens
4521	nudix (nucleoside diphosphate linked moiety X)-type motif 1	NUDT1	Homo sapiens
11164	nudix (nucleoside diphosphate linked moiety X)-type motif 5	NUDT5	Homo sapiens
26747	nuclear fragile X mental retardation protein interacting protein 1	NUFIP1	Homo sapiens
4926	nuclear mitotic apparatus protein 1	NUMA1	Homo sapiens
8650	numb homolog (Drosophila)	NUMB	Homo sapiens
9972	nucleoporin 153kDa	NUP153	Homo sapiens
9631	nucleoporin 155kDa	NUP155	Homo sapiens
8021	nucleoporin 214kDa	NUP214	Homo sapiens
23636	nucleoporin 62kDa	NUP62	Homo sapiens
4927	nucleoporin 88kDa	NUP88	Homo sapiens
4928	nucleoporin 98kDa	NUP98	Homo sapiens
11097	nucleoporin like 2	NUPL2	Homo sapiens
55916	nuclear transport factor 2-like export factor 2	NXT2	Homo sapiens
4938	2',5'-oligoadenylate synthetase 1, 40/46kDa	OAS1	Homo sapiens
4942	ornithine aminotransferase (gyrate atrophy)	OAT	Homo sapiens
4947	ornithine decarboxylase antizyme 2	OAZ2	Homo sapiens
4948	oculocutaneous albinism II	OCA2	Homo sapiens
4953	ornithine decarboxylase 1	ODC1	Homo sapiens
4956	outer dense fiber of sperm tails 1	ODF1	Homo sapiens
4957	outer dense fiber of sperm tails 2	ODF2	Homo sapiens
8481	oral-facial-digital syndrome 1	OFD1	Homo sapiens
11054	opioid growth factor receptor	OGFR	Homo sapiens
8473	O-linked N-acetylglucosamine (GlcNAc) transferase (UDP-N-acetylglucosamine:polypeptide-N-acetylglucosaminyl transferase)	OGT	Homo sapiens
10439	olfactomedin 1	OLFM1	Homo sapiens
10562	olfactomedin 4	OLFM4	Homo sapiens
283298	olfactomedin-like 1	OLFML1	Homo sapiens
169611	olfactomedin-like 2A	OLFML2A	Homo sapiens
10215	oligodendrocyte lineage transcription factor 2	OLIG2	Homo sapiens

4973	oxidized low density lipoprotein (lectin-like) receptor 1	OLR1	Homo sapiens
4974	oligodendrocyte myelin glycoprotein	OMG	Homo sapiens
3175	one cut homeobox 1	ONECUT1	Homo sapiens
9480	one cut homeobox 2	ONECUT2	Homo sapiens
4976	optic atrophy 1 (autosomal dominant)	OPA1	Homo sapiens
4978	opioid binding protein/cell adhesion molecule-like	OPCML	Homo sapiens
4986	opioid receptor, kappa 1	OPRK1	Homo sapiens
10133	optineurin	OPTN	Homo sapiens
23595	origin recognition complex, subunit 3-like (yeast)	ORC3L	Homo sapiens
5000	origin recognition complex, subunit 4-like (yeast)	ORC4L	Homo sapiens
5001	origin recognition complex, subunit 5-like (yeast)	ORC5L	Homo sapiens
29095	ORM1-like 2 (S. cerevisiae)	ORMDL2	Homo sapiens
94103	ORM1-like 3 (S. cerevisiae)	ORMDL3	Homo sapiens
5007	oxysterol binding protein	OSBP	Homo sapiens
114884	oxysterol binding protein-like 10	OSBPL10	Homo sapiens
114885	oxysterol binding protein-like 11	OSBPL11	Homo sapiens
9885	oxysterol binding protein-like 2	OSBPL2	Homo sapiens
26031	oxysterol binding protein-like 3	OSBPL3	Homo sapiens
114880	oxysterol binding protein-like 6	OSBPL6	Homo sapiens
114882	oxysterol binding protein-like 8	OSBPL8	Homo sapiens
114883	oxysterol binding protein-like 9	OSBPL9	Homo sapiens
55644	O-sialoglycoprotein endopeptidase	OSGEP	Homo sapiens
9180	oncostatin M receptor	OSMR	Homo sapiens
26578	osteoclast stimulating factor 1	OSTF1	Homo sapiens
5009	ornithine carbamoyltransferase	OTC	Homo sapiens
23440	orthopedia homeobox	OTP	Homo sapiens
55611	OTU domain, ubiquitin aldehyde binding 1	OTUB1	Homo sapiens
5018	oxidase (cytochrome c) assembly 1-like	OXA1L	Homo sapiens
5019	3-oxoacid CoA transferase 1	OXCT1	Homo sapiens
5023	purinergic receptor P2X, ligand-gated ion channel, 1	P2RX1	Homo sapiens
5025	purinergic receptor P2X, ligand-gated ion channel, 4	P2RX4	Homo sapiens
5026	purinergic receptor P2X, ligand-gated ion channel, 5	P2RX5	Homo sapiens
27334	purinergic receptor P2Y, G-protein coupled, 10	P2RY10	Homo sapiens
5029	purinergic receptor P2Y, G-protein coupled, 2	P2RY2	Homo sapiens
5033	prolyl 4-hydroxylase, alpha polypeptide I	P4HA1	Homo sapiens
8974	prolyl 4-hydroxylase, alpha polypeptide II	P4HA2	Homo sapiens
5034	prolyl 4-hydroxylase, beta polypeptide	P4HB	Homo sapiens
647033	proliferation-associated 2G4, 38kDa; proliferation-associated 2G4 pseudogene 4	PA2G4	Homo sapiens
5036	proliferation-associated 2G4, 38kDa; proliferation-associated 2G4 pseudogene 4	PA2G4	Homo sapiens
341315	poly(A) binding protein, cytoplasmic pseudogene 5; poly(A) binding protein, cytoplasmic 1	PABPC1	Homo sapiens
26986	poly(A) binding protein, cytoplasmic pseudogene 5; poly(A) binding protein, cytoplasmic 1	PABPC1	Homo sapiens
5042	poly(A) binding protein, cytoplasmic 3	PABPC3	Homo sapiens
8106	poly(A) binding protein, nuclear 1	PABPN1	Homo sapiens

11240	peptidyl arginine deiminase, type II	PADI2	Homo sapiens
5047	progestagen-associated endometrial protein	PAEP	Homo sapiens
5048	platelet-activating factor acetylhydrolase, isoform Ib, subunit 1 (45kDa)	PAFAH1B1	Homo sapiens
5050	platelet-activating factor acetylhydrolase, isoform Ib, subunit 3 (29kDa)	PAFAH1B3	Homo sapiens
5053	phenylalanine hydroxylase	PAH	Homo sapiens
10606	phosphoribosylaminoimidazole carboxylase, phosphoribosylaminoimidazole succinocarboxamide synthetase	PAICS	Homo sapiens
51247	poly(A) binding protein interacting protein 2	PAIP2	Homo sapiens
5058	p21 protein (Cdc42/Rac)-activated kinase 1	PAK1	Homo sapiens
5062	p21 protein (Cdc42/Rac)-activated kinase 2	PAK2	Homo sapiens
5063	p21 protein (Cdc42/Rac)-activated kinase 3	PAK3	Homo sapiens
5064	paralemmin	PALM	Homo sapiens
5066	peptidylglycine alpha-amidating monooxygenase	PAM	Homo sapiens
53354	pantothenate kinase 1	PANK1	Homo sapiens
80025	pantothenate kinase 2	PANK2	Homo sapiens
24145	pannexin 1	PANX1	Homo sapiens
10914	poly(A) polymerase alpha	PAPOLA	Homo sapiens
493913	PAPPA antisense RNA (non-protein coding); pregnancy-associated plasma protein A, pappalysin 1	PAPPA	Homo sapiens
5069	PAPPA antisense RNA (non-protein coding); pregnancy-associated plasma protein A, pappalysin 1	PAPPA	Homo sapiens
9061	3'-phosphoadenosine 5'-phosphosulfate synthase 1	PAPSS1	Homo sapiens
50855	par-6 partitioning defective 6 homolog alpha (C. elegans)	PARD6A	Homo sapiens
142	poly (ADP-ribose) polymerase 1	PARP1	Homo sapiens
10038	poly (ADP-ribose) polymerase 2	PARP2	Homo sapiens
29780	parvin, beta	PARVB	Homo sapiens
23178	PAS domain containing serine/threonine kinase	PASK	Homo sapiens
5074	PRKC, apoptosis, WT1, regulator	PAWR	Homo sapiens
5075	paired box 1	PAX1	Homo sapiens
5076	paired box 2	PAX2	Homo sapiens
5077	paired box 3	PAX3	Homo sapiens
7849	paired box 8	PAX8	Homo sapiens
5083	paired box 9	PAX9	Homo sapiens
5087	pre-B-cell leukemia homeobox 1	PBX1	Homo sapiens
57326	pre-B-cell leukemia homeobox interacting protein 1	PBXIP1	Homo sapiens
5093	poly(rC) binding protein 1	PCBP1	Homo sapiens
5094	poly(rC) binding protein 2	PCBP2	Homo sapiens
5095	propionyl Coenzyme A carboxylase, alpha polypeptide	PCCA	Homo sapiens
5096	propionyl Coenzyme A carboxylase, beta polypeptide	PCCB	Homo sapiens
5097	protocadherin 1	PCDH1	Homo sapiens
27328	protocadherin 11 X-linked	PCDH11X	Homo sapiens
51294	protocadherin 12	PCDH12	Homo sapiens
5100	protocadherin 8	PCDH8	Homo sapiens
56127	protocadherin beta 10; protocadherin beta 9	PCDHB10	Homo sapiens
56126	protocadherin beta 10; protocadherin beta 9	PCDHB10	Homo sapiens

5106	phosphoenolpyruvate carboxykinase 2 (mitochondrial)	PCK2	Homo sapiens
5108	pericentriolar material 1	PCM1	Homo sapiens
5110	protein-L-isoaspartate (D-aspartate) O-methyltransferase	PCMT1	Homo sapiens
5111	proliferating cell nuclear antigen	PCNA	Homo sapiens
22990	pecanex homolog (Drosophila)	PCNX	Homo sapiens
5118	procollagen C-endopeptidase enhancer	PCOLCE	Homo sapiens
26577	procollagen C-endopeptidase enhancer 2	PCOLCE2	Homo sapiens
5121	Purkinje cell protein 4	PCP4	Homo sapiens
5122	proprotein convertase subtilisin/kexin type 1	PCSK1	Homo sapiens
5126	proprotein convertase subtilisin/kexin type 2	PCSK2	Homo sapiens
5125	proprotein convertase subtilisin/kexin type 5	PCSK5	Homo sapiens
5046	proprotein convertase subtilisin/kexin type 6	PCSK6	Homo sapiens
5130	phosphate cytidylyltransferase 1, choline, alpha	PCYT1A	Homo sapiens
9468	phosphate cytidylyltransferase 1, choline, beta	PCYT1B	Homo sapiens
645181	PDGFA associated protein 1; similar to PDGFA associated protein 1	PDAP1	Homo sapiens
11333	PDGFA associated protein 1; similar to PDGFA associated protein 1	PDAP1	Homo sapiens
11235	programmed cell death 10	PDCD10	Homo sapiens
27250	programmed cell death 4 (neoplastic transformation inhibitor)	PDCD4	Homo sapiens
9141	programmed cell death 5	PDCD5	Homo sapiens
57491	aryl-hydrocarbon receptor repressor; programmed cell death 6	PDCD6	Homo sapiens
10016	aryl-hydrocarbon receptor repressor; programmed cell death 6	PDCD6	Homo sapiens
10846	phosphodiesterase 10A	PDE10A	Homo sapiens
50940	phosphodiesterase 11A	PDE11A	Homo sapiens
5136	phosphodiesterase 1A, calmodulin-dependent	PDE1A	Homo sapiens
5137	phosphodiesterase 1C, calmodulin-dependent 70kDa	PDE1C	Homo sapiens
5138	phosphodiesterase 2A, cGMP-stimulated	PDE2A	Homo sapiens
5140	phosphodiesterase 3B, cGMP-inhibited	PDE3B	Homo sapiens
5141	phosphodiesterase 4A, cAMP-specific (phosphodiesterase E2 dunce homolog, Drosophila)	PDE4A	Homo sapiens
5144	phosphodiesterase 4D, cAMP-specific (phosphodiesterase E3 dunce homolog, Drosophila)	PDE4D	Homo sapiens
652164	hypothetical protein LOC100134230; similar to KIAA0454 protein; similar to phosphodiesterase 4D interacting protein isoform 2; phosphodiesterase 4D interacting protein	PDE4DIP	Homo sapiens
100134230	hypothetical protein LOC100134230; similar to KIAA0454 protein; similar to phosphodiesterase 4D interacting protein isoform 2; phosphodiesterase 4D interacting protein	PDE4DIP	Homo sapiens
727927	hypothetical protein LOC100134230; similar to KIAA0454 protein; similar to phosphodiesterase 4D interacting protein isoform 2; phosphodiesterase 4D interacting protein	PDE4DIP	Homo sapiens
9659	hypothetical protein LOC100134230; similar to KIAA0454 protein; similar to phosphodiesterase 4D interacting protein isoform 2; phosphodiesterase 4D interacting protein	PDE4DIP	Homo sapiens
8654	phosphodiesterase 5A, cGMP-specific	PDE5A	Homo sapiens
5145	phosphodiesterase 6A, cGMP-specific, rod, alpha	PDE6A	Homo sapiens
5147	phosphodiesterase 6D, cGMP-specific, rod, delta	PDE6D	Homo sapiens
5151	phosphodiesterase 8A	PDE8A	Homo sapiens
8622	phosphodiesterase 8B	PDE8B	Homo sapiens

5152	phosphodiesterase 9A	PDE9A	Homo sapiens
56034	platelet derived growth factor C	PDGFC	Homo sapiens
5156	platelet-derived growth factor receptor, alpha polypeptide	PDGFRA	Homo sapiens
5157	platelet-derived growth factor receptor-like	PDGFRL	Homo sapiens
5160	pyruvate dehydrogenase (lipoamide) alpha 1	PDHA1	Homo sapiens
5161	pyruvate dehydrogenase (lipoamide) alpha 2	PDHA2	Homo sapiens
5163	pyruvate dehydrogenase kinase, isozyme 1	PDK1	Homo sapiens
5164	pyruvate dehydrogenase kinase, isozyme 2	PDK2	Homo sapiens
5165	pyruvate dehydrogenase kinase, isozyme 3	PDK3	Homo sapiens
5166	pyruvate dehydrogenase kinase, isozyme 4	PDK4	Homo sapiens
27295	PDZ and LIM domain 3	PDLIM3	Homo sapiens
8572	PDZ and LIM domain 4	PDLIM4	Homo sapiens
5173	prodynorphin	PDYN	Homo sapiens
8682	phosphoprotein enriched in astrocytes 15	PEA15	Homo sapiens
5175	platelet/endothelial cell adhesion molecule	PECAM1	Homo sapiens
57162	pellino homolog 1 (Drosophila)	PELI1	Homo sapiens
10400	phosphatidylethanolamine N-methyltransferase	PEMT	Homo sapiens
5179	proenkephalin	PENK	Homo sapiens
5184	peptidase D	PEPD	Homo sapiens
5187	period homolog 1 (Drosophila)	PER1	Homo sapiens
8864	period homolog 2 (Drosophila)	PER2	Homo sapiens
64065	PERP, TP53 apoptosis effector	PERP	Homo sapiens
23481	pescadillo homolog 1, containing BRCT domain (zebrafish)	PES1	Homo sapiens
5188	PET112-like (yeast)	PET112L	Homo sapiens
5189	peroxisomal biogenesis factor 1	PEX1	Homo sapiens
8800	peroxisomal biogenesis factor 11 alpha	PEX11A	Homo sapiens
8799	peroxisomal biogenesis factor 11 beta	PEX11B	Homo sapiens
5193	peroxisomal biogenesis factor 12	PEX12	Homo sapiens
5194	peroxisomal biogenesis factor 13	PEX13	Homo sapiens
5195	peroxisomal biogenesis factor 14	PEX14	Homo sapiens
5824	peroxisomal biogenesis factor 19	PEX19	Homo sapiens
5830	peroxisomal biogenesis factor 5	PEX5	Homo sapiens
5190	peroxisomal biogenesis factor 6	PEX6	Homo sapiens
5191	peroxisomal biogenesis factor 7	PEX7	Homo sapiens
5196	platelet factor 4	PF4	Homo sapiens
5198	phosphoribosylformylglycinamidine synthase	PFAS	Homo sapiens
5207	6-phosphofructo-2-kinase/fructose-2,6-biphosphatase 1	PFKFB1	Homo sapiens
5208	6-phosphofructo-2-kinase/fructose-2,6-biphosphatase 2	PFKFB2	Homo sapiens
5210	6-phosphofructo-2-kinase/fructose-2,6-biphosphatase 4	PFKFB4	Homo sapiens
5213	phosphofructokinase, muscle	PFKM	Homo sapiens
5214	phosphofructokinase, platelet	PFKP	Homo sapiens
5216	profilin 1	PFN1	Homo sapiens
5224	phosphoglycerate mutase 2 (muscle)	PGAM2	Homo sapiens
5225	progastricsin (pepsinogen C)	PGC	Homo sapiens
5226	phosphogluconate dehydrogenase	PGD	Homo sapiens
5228	placental growth factor	PGF	Homo sapiens

5229	protein geranylgeranyltransferase type I, beta subunit	PGGT1B	Homo sapiens
5230	phosphoglycerate kinase 1	PGK1	Homo sapiens
25796	6-phosphogluconolactonase	PGLS	Homo sapiens
5236	phosphoglucomutase 1	PGM1	Homo sapiens
55276	phosphoglucomutase 2	PGM2	Homo sapiens
5239	phosphoglucomutase 5	PGM5	Homo sapiens
5241	progesterone receptor	PGR	Homo sapiens
10857	progesterone receptor membrane component 1	PGRMC1	Homo sapiens
10424	progesterone receptor membrane component 2	PGRMC2	Homo sapiens
5245	prohibitin	PHB	Homo sapiens
1912	polyhomeotic homolog 2 (Drosophila)	PHC2	Homo sapiens
5252	PHD finger protein 1	PHF1	Homo sapiens
9678	PHD finger protein 14	PHF14	Homo sapiens
5253	PHD finger protein 2	PHF2	Homo sapiens
23469	PHD finger protein 3	PHF3	Homo sapiens
55023	pleckstrin homology domain interacting protein	PHIP	Homo sapiens
646780	phosphorylase kinase, alpha 1 pseudogene 1; phosphorylase kinase, alpha 1 (muscle)	PHKA1	Homo sapiens
5255	phosphorylase kinase, alpha 1 pseudogene 1; phosphorylase kinase, alpha 1 (muscle)	PHKA1	Homo sapiens
5256	phosphorylase kinase, alpha 2 (liver)	PHKA2	Homo sapiens
5257	phosphorylase kinase, beta	PHKB	Homo sapiens
5261	phosphorylase kinase, gamma 2 (testis)	PHKG2	Homo sapiens
7262	pleckstrin homology-like domain, family A, member 2	PHLDA2	Homo sapiens
23612	pleckstrin homology-like domain, family A, member 3	PHLDA3	Homo sapiens
8929	paired-like homeobox 2b	PHOX2B	Homo sapiens
10745	putative homeodomain transcription factor 1	PHTF1	Homo sapiens
57157	putative homeodomain transcription factor 2	PHTF2	Homo sapiens
5264	phytanoyl-CoA 2-hydroxylase	PHYH	Homo sapiens
5266	peptidase inhibitor 3, skin-derived	PI3	Homo sapiens
8554	protein inhibitor of activated STAT, 1	PIAS1	Homo sapiens
9063	protein inhibitor of activated STAT, 2	PIAS2	Homo sapiens
10401	protein inhibitor of activated STAT, 3	PIAS3	Homo sapiens
8301	phosphatidylinositol binding clathrin assembly protein	PICALM	Homo sapiens
5277	phosphatidylinositol glycan anchor biosynthesis, class A	PIGA	Homo sapiens
9488	phosphatidylinositol glycan anchor biosynthesis, class B	PIGB	Homo sapiens
5279	phosphatidylinositol glycan anchor biosynthesis, class C	PIGC	Homo sapiens
5281	phosphatidylinositol glycan anchor biosynthesis, class F	PIGF	Homo sapiens
9487	phosphatidylinositol glycan anchor biosynthesis, class L	PIGL	Homo sapiens
9091	phosphatidylinositol glycan anchor biosynthesis, class Q	PIGQ	Homo sapiens
5284	polymeric immunoglobulin receptor	PIGR	Homo sapiens
5287	phosphoinositide-3-kinase, class 2, beta polypeptide	PIK3C2B	Homo sapiens
5288	phosphoinositide-3-kinase, class 2, gamma polypeptide	PIK3C2G	Homo sapiens
5289	phosphoinositide-3-kinase, class 3	PIK3C3	Homo sapiens
5290	phosphoinositide-3-kinase, catalytic, alpha polypeptide	PIK3CA	Homo sapiens
5291	phosphoinositide-3-kinase, catalytic, beta polypeptide	PIK3CB	Homo sapiens

5294	phosphoinositide-3-kinase, catalytic, gamma polypeptide	PIK3CG	Homo sapiens
5295	phosphoinositide-3-kinase, regulatory subunit 1 (alpha)	PIK3R1	Homo sapiens
5296	phosphoinositide-3-kinase, regulatory subunit 2 (beta)	PIK3R2	Homo sapiens
8503	phosphoinositide-3-kinase, regulatory subunit 3 (gamma)	PIK3R3	Homo sapiens
30849	phosphoinositide-3-kinase, regulatory subunit 4	PIK3R4	Homo sapiens
5304	prolactin-induced protein	PIP	Homo sapiens
8394	phosphatidylinositol-4-phosphate 5-kinase, type I, alpha	PIP5K1A	Homo sapiens
8395	phosphatidylinositol-4-phosphate 5-kinase, type I, beta	PIP5K1B	Homo sapiens
23396	phosphatidylinositol-4-phosphate 5-kinase, type I, gamma	PIP5K1C	Homo sapiens
8544	pirin (iron-binding nuclear protein)	PIR	Homo sapiens
23761	phosphatidylserine decarboxylase	PISD	Homo sapiens
5306	phosphatidylinositol transfer protein, alpha	PITPNA	Homo sapiens
23760	phosphatidylinositol transfer protein, beta	PITPNB	Homo sapiens
9600	phosphatidylinositol transfer protein, membrane-associated 1	PITPNM1	Homo sapiens
5308	paired-like homeodomain 2	PITX2	Homo sapiens
9271	piwi-like 1 (Drosophila)	PIWIL1	Homo sapiens
55124	hydroxy-delta-5-steroid dehydrogenase, 3 beta- and steroid delta-isomerase 7; piwi-like 2 (Drosophila)	PIWIL2	Homo sapiens
80270	hydroxy-delta-5-steroid dehydrogenase, 3 beta- and steroid delta-isomerase 7; piwi-like 2 (Drosophila)	PIWIL2	Homo sapiens
5311	polycystic kidney disease 2 (autosomal dominant)	PKD2	Homo sapiens
5569	protein kinase (cAMP-dependent, catalytic) inhibitor alpha	PKIA	Homo sapiens
11142	protein kinase (cAMP-dependent, catalytic) inhibitor gamma	PKIG	Homo sapiens
5313	pyruvate kinase, liver and RBC	PKLR	Homo sapiens
652797	similar to Pyruvate kinase, isozymes M1/M2 (Pyruvate kinase muscle isozyme) (Cytosolic thyroid hormone-binding protein) (CTHBP) (THBP1); pyruvate kinase, muscle	PKM2	Homo sapiens
5315	similar to Pyruvate kinase, isozymes M1/M2 (Pyruvate kinase muscle isozyme) (Cytosolic thyroid hormone-binding protein) (CTHBP) (THBP1); pyruvate kinase, muscle	PKM2	Homo sapiens
5585	protein kinase N1	PKN1	Homo sapiens
5586	protein kinase N2	PKN2	Homo sapiens
5316	PBX/knotted 1 homeobox 1	PKNOX1	Homo sapiens
11187	plakophilin 3	PKP3	Homo sapiens
8502	plakophilin 4	PKP4	Homo sapiens
51365	phospholipase A1 member A	PLA1A	Homo sapiens
5320	phospholipase A2, group IIA (platelets, synovial fluid)	PLA2G2A	Homo sapiens
30814	phospholipase A2, group IIE	PLA2G2E	Homo sapiens
5321	phospholipase A2, group IVA (cytosolic, calcium-dependent)	PLA2G4A	Homo sapiens
8681	JMJD7-PLA2G4B readthrough transcript; phospholipase A2, group IVB (cytosolic); jumonji domain containing 7	PLA2G4B	Homo sapiens
100137047	JMJD7-PLA2G4B readthrough transcript; phospholipase A2, group IVB (cytosolic); jumonji domain containing 7	PLA2G4B	Homo sapiens
100137049	JMJD7-PLA2G4B readthrough transcript; phospholipase A2, group IVB (cytosolic); jumonji domain containing 7	PLA2G4B	Homo sapiens
8605	phospholipase A2, group IVC (cytosolic, calcium-independent)	PLA2G4C	Homo sapiens
5322	phospholipase A2, group V	PLA2G5	Homo sapiens
8398	phospholipase A2, group VI (cytosolic, calcium-independent)	PLA2G6	Homo sapiens

7941	phospholipase A2, group VII (platelet-activating factor acetylhydrolase, plasma)	PLA2G7	Homo sapiens
22925	phospholipase A2 receptor 1, 180kDa	PLA2R1	Homo sapiens
5324	pleiomorphic adenoma gene 1	PLAG1	Homo sapiens
152845	pleiomorphic adenoma gene-like 2; similar to pleiomorphic adenoma gene-like 2	PLAGL2	Homo sapiens
5326	pleiomorphic adenoma gene-like 2; similar to pleiomorphic adenoma gene-like 2	PLAGL2	Homo sapiens
5327	plasminogen activator, tissue	PLAT	Homo sapiens
5328	plasminogen activator, urokinase	PLAU	Homo sapiens
5329	plasminogen activator, urokinase receptor	PLAUR	Homo sapiens
23236	phospholipase C, beta 1 (phosphoinositide-specific)	PLCB1	Homo sapiens
5330	phospholipase C, beta 2	PLCB2	Homo sapiens
5332	phospholipase C, beta 4	PLCB4	Homo sapiens
5333	phospholipase C, delta 1	PLCD1	Homo sapiens
5335	phospholipase C, gamma 1	PLCG1	Homo sapiens
5336	phospholipase C, gamma 2 (phosphatidylinositol-specific)	PLCG2	Homo sapiens
23228	phospholipase C-like 2	PLCL2	Homo sapiens
5337	phospholipase D1, phosphatidylcholine-specific	PLD1	Homo sapiens
5338	phospholipase D2	PLD2	Homo sapiens
23646	phospholipase D family, member 3	PLD3	Homo sapiens
5341	pleckstrin	PLEK	Homo sapiens
59338	pleckstrin homology domain containing, family A (phosphoinositide binding specific) member 1	PLEKHA1	Homo sapiens
58473	pleckstrin homology domain containing, family B (evectins) member 1	PLEKHB1	Homo sapiens
5340	plasminogen	PLG	Homo sapiens
5347	polo-like kinase 1 (Drosophila)	PLK1	Homo sapiens
10769	polo-like kinase 2 (Drosophila)	PLK2	Homo sapiens
1263	polo-like kinase 3 (Drosophila)	PLK3	Homo sapiens
5352	procollagen-lysine, 2-oxoglutarate 5-dioxygenase 2	PLOD2	Homo sapiens
8985	procollagen-lysine, 2-oxoglutarate 5-dioxygenase 3	PLOD3	Homo sapiens
5354	proteolipid protein 1	PLP1	Homo sapiens
5355	proteolipid protein 2 (colonic epithelium-enriched)	PLP2	Homo sapiens
5358	plastin 3 (T isoform)	PLS3	Homo sapiens
5359	phospholipid scramblase 1	PLSCR1	Homo sapiens
5360	phospholipid transfer protein	PLTP	Homo sapiens
55558	plexin A3	PLXNA3	Homo sapiens
5364	plexin B1	PLXNB1	Homo sapiens
5365	plexin B3	PLXNB3	Homo sapiens
10154	plexin C1	PLXNC1	Homo sapiens
652346	promyelocytic leukemia; similar to promyelocytic leukemia protein isoform 1	PML	Homo sapiens
5371	promyelocytic leukemia; similar to promyelocytic leukemia protein isoform 1	PML	Homo sapiens
5372	phosphomannomutase 1	PMM1	Homo sapiens
5376	peripheral myelin protein 22	PMP22	Homo sapiens
9512	peptidase (mitochondrial processing) beta	PMPCB	Homo sapiens

5378	PMS1 postmeiotic segregation increased 1 (S. cerevisiae)	PMS1	Homo sapiens
10654	phosphomevalonate kinase	PMVK	Homo sapiens
5407	pancreatic lipase-related protein 1	PNLIPRP1	Homo sapiens
5408	pancreatic lipase-related protein 2	PNLIPRP2	Homo sapiens
5409	phenylethanolamine N-methyltransferase	PNMT	Homo sapiens
5411	pinin, desmosome associated protein	PNN	Homo sapiens
5368	prepronociceptin	PNOC	Homo sapiens
87178	polyribonucleotide nucleotidyltransferase 1	PNPT1	Homo sapiens
10957	proline-rich nuclear receptor coactivator 1	PNRC1	Homo sapiens
55629	proline-rich nuclear receptor coactivator 2; similar to hCG1728885	PNRC2	Homo sapiens
100131261	proline-rich nuclear receptor coactivator 2; similar to hCG1728885	PNRC2	Homo sapiens
5420	podocalyxin-like	PODXL	Homo sapiens
5423	polymerase (DNA directed), beta	POLB	Homo sapiens
5424	polymerase (DNA directed), delta 1, catalytic subunit 125kDa	POLD1	Homo sapiens
5425	polymerase (DNA directed), delta 2, regulatory subunit 50kDa	POLD2	Homo sapiens
10714	polymerase (DNA-directed), delta 3, accessory subunit	POLD3	Homo sapiens
57804	polymerase (DNA-directed), delta 4	POLD4	Homo sapiens
84271	polymerase (DNA-directed), delta interacting protein 3	POLDIP3	Homo sapiens
5427	polymerase (DNA directed), epsilon 2 (p59 subunit)	POLE2	Homo sapiens
11232	polymerase (DNA directed), gamma 2, accessory subunit	POLG2	Homo sapiens
5430	polymerase (RNA) II (DNA directed) polypeptide A, 220kDa	POLR2A	Homo sapiens
5431	polymerase (RNA) II (DNA directed) polypeptide B, 140kDa	POLR2B	Homo sapiens
5435	polymerase (RNA) II (DNA directed) polypeptide F	POLR2F	Homo sapiens
5436	polymerase (RNA) II (DNA directed) polypeptide G	POLR2G	Homo sapiens
5437	polymerase (RNA) II (DNA directed) polypeptide H	POLR2H	Homo sapiens
5439	polymerase (RNA) II (DNA directed) polypeptide J, 13.3kDa	POLR2J	Homo sapiens
5440	polymerase (RNA) II (DNA directed) polypeptide K, 7.0kDa	POLR2K	Homo sapiens
10623	polymerase (RNA) III (DNA directed) polypeptide C (62kD)	POLR3C	Homo sapiens
661	polymerase (RNA) III (DNA directed) polypeptide D, 44kDa	POLR3D	Homo sapiens
5442	polymerase (RNA) mitochondrial (DNA directed)	POLRMT	Homo sapiens
5445	paraoxonase 2	PON2	Homo sapiens
10775	processing of precursor 4, ribonuclease P/MRP subunit (S. cerevisiae)	POP4	Homo sapiens
10248	processing of precursor 7, ribonuclease P/MRP subunit (S. cerevisiae)	POP7	Homo sapiens
10631	periostin, osteoblast specific factor	POSTN	Homo sapiens
5450	POU class 2 associating factor 1	POU2AF1	Homo sapiens
5451	POU class 2 homeobox 1	POU2F1	Homo sapiens
5454	POU class 3 homeobox 2	POU3F2	Homo sapiens
5456	POU class 3 homeobox 4	POU3F4	Homo sapiens
5457	POU class 4 homeobox 1	POU4F1	Homo sapiens
8613	phosphatidic acid phosphatase type 2B	PPAP2B	Homo sapiens
8612	phosphatidic acid phosphatase type 2C	PPAP2C	Homo sapiens
5468	peroxisome proliferator-activated receptor gamma	PPARG	Homo sapiens
5471	phosphoribosyl pyrophosphate amidotransferase	PPAT	Homo sapiens
5473	pro-platelet basic protein (chemokine (C-X-C motif) ligand 7)	PPBP	Homo sapiens
5475	protein phosphatase, EF-hand calcium binding domain 1	PPEF1	Homo sapiens
8500	protein tyrosine phosphatase, receptor type, f polypeptide (PTPRF),	PPFIA1	Homo sapiens

	interacting protein (liprin), alpha 1		
8541	protein tyrosine phosphatase, receptor type, f polypeptide (PTPRF), interacting protein (liprin), alpha 3	PPFIA3	Homo sapiens
8496	PTPRF interacting protein, binding protein 1 (liprin beta 1)	PPFIBP1	Homo sapiens
8495	PTPRF interacting protein, binding protein 2 (liprin beta 2)	PPFIBP2	Homo sapiens
5479	peptidylprolyl isomerase B (cyclophilin B)	PPIB	Homo sapiens
5480	peptidylprolyl isomerase C (cyclophilin C)	PPIC	Homo sapiens
5481	peptidylprolyl isomerase D	PPID	Homo sapiens
10450	peptidylprolyl isomerase E (cyclophilin E)	PPIE	Homo sapiens
10105	peptidylprolyl isomerase F	PPIF	Homo sapiens
9360	peptidylprolyl isomerase G (cyclophilin G)	PPIG	Homo sapiens
10465	peptidylprolyl isomerase H (cyclophilin H)	PPIH	Homo sapiens
23759	peptidylprolyl isomerase (cyclophilin)-like 2	PPIL2	Homo sapiens
5493	periplakin	PPL	Homo sapiens
5494	protein phosphatase 1A (formerly 2C), magnesium-dependent, alpha isoform	PPM1A	Homo sapiens
5495	protein phosphatase 1B (formerly 2C), magnesium-dependent, beta isoform	PPM1B	Homo sapiens
8493	protein phosphatase 1D magnesium-dependent, delta isoform	PPM1D	Homo sapiens
9647	protein phosphatase 1F (PP2C domain containing)	PPM1F	Homo sapiens
5498	protoporphyrinogen oxidase	PPOX	Homo sapiens
5499	protein phosphatase 1, catalytic subunit, alpha isoform	PPP1CA	Homo sapiens
5501	protein phosphatase 1, catalytic subunit, gamma isoform	PPP1CC	Homo sapiens
5514	protein phosphatase 1, regulatory (inhibitor) subunit 10	PPP1R10	Homo sapiens
6992	protein phosphatase 1, regulatory (inhibitor) subunit 11	PPP1R11	Homo sapiens
4659	protein phosphatase 1, regulatory (inhibitor) subunit 12A	PPP1R12A	Homo sapiens
4660	protein phosphatase 1, regulatory (inhibitor) subunit 12B	PPP1R12B	Homo sapiens
23368	protein phosphatase 1, regulatory (inhibitor) subunit 13B	PPP1R13B	Homo sapiens
23645	protein phosphatase 1, regulatory (inhibitor) subunit 15A	PPP1R15A	Homo sapiens
26051	protein phosphatase 1, regulatory (inhibitor) subunit 16B	PPP1R16B	Homo sapiens
5504	protein phosphatase 1, regulatory (inhibitor) subunit 2	PPP1R2	Homo sapiens
5506	protein phosphatase 1, regulatory (inhibitor) subunit 3A	PPP1R3A	Homo sapiens
5510	protein phosphatase 1, regulatory (inhibitor) subunit 7	PPP1R7	Homo sapiens
5515	protein phosphatase 2 (formerly 2A), catalytic subunit, alpha isoform	PPP2CA	Homo sapiens
5516	protein phosphatase 2 (formerly 2A), catalytic subunit, beta isoform	PPP2CB	Homo sapiens
5518	protein phosphatase 2 (formerly 2A), regulatory subunit A, alpha isoform	PPP2R1A	Homo sapiens
5520	protein phosphatase 2 (formerly 2A), regulatory subunit B, alpha isoform	PPP2R2A	Homo sapiens
5524	protein phosphatase 2A activator, regulatory subunit 4	PPP2R4	Homo sapiens
5525	protein phosphatase 2, regulatory subunit B', alpha isoform	PPP2R5A	Homo sapiens
5526	protein phosphatase 2, regulatory subunit B', beta isoform	PPP2R5B	Homo sapiens
5527	protein phosphatase 2, regulatory subunit B', gamma isoform	PPP2R5C	Homo sapiens
5529	protein phosphatase 2, regulatory subunit B', epsilon isoform	PPP2R5E	Homo sapiens
5530	protein phosphatase 3 (formerly 2B), catalytic subunit, alpha isoform	PPP3CA	Homo sapiens
5532	protein phosphatase 3 (formerly 2B), catalytic subunit, beta isoform	PPP3CB	Homo sapiens
5533	protein phosphatase 3 (formerly 2B), catalytic subunit, gamma	PPP3CC	Homo sapiens

	isoform		
5534	protein phosphatase 3 (formerly 2B), regulatory subunit B, alpha isoform	PPP3R1	Homo sapiens
9989	protein phosphatase 4, regulatory subunit 1	PPP4R1	Homo sapiens
5537	protein phosphatase 6, catalytic subunit	PPP6C	Homo sapiens
5538	palmitoyl-protein thioesterase 1	PPT1	Homo sapiens
10084	polyglutamine binding protein 1	PQBP1	Homo sapiens
11230	PRA1 domain family, member 2	PRAF2	Homo sapiens
23532	preferentially expressed antigen in melanoma	PRAME	Homo sapiens
5546	papillary renal cell carcinoma (translocation-associated)	PRCC	Homo sapiens
5547	prolylcarboxypeptidase (angiotensinase C)	PRCP	Homo sapiens
639	PR domain containing 1, with ZNF domain	PRDM1	Homo sapiens
63976	PR domain containing 16	PRDM16	Homo sapiens
7799	PR domain containing 2, with ZNF domain	PRDM2	Homo sapiens
5052	peroxiredoxin 1	PRDX1	Homo sapiens
10935	peroxiredoxin 3	PRDX3	Homo sapiens
10549	peroxiredoxin 4	PRDX4	Homo sapiens
25824	peroxiredoxin 5	PRDX5	Homo sapiens
9588	peroxiredoxin 6	PRDX6	Homo sapiens
5549	proline/arginine-rich end leucine-rich repeat protein	PRELP	Homo sapiens
5550	prolyl endopeptidase	PREP	Homo sapiens
5551	perforin 1 (pore forming protein)	PRF1	Homo sapiens
5553	proteoglycan 2, bone marrow (natural killer cell activator, eosinophil granule major basic protein)	PRG2	Homo sapiens
5557	primase, DNA, polypeptide 1 (49kDa)	PRIM1	Homo sapiens
5562	protein kinase, AMP-activated, alpha 1 catalytic subunit	PRKAA1	Homo sapiens
5563	protein kinase, AMP-activated, alpha 2 catalytic subunit	PRKAA2	Homo sapiens
5565	protein kinase, AMP-activated, beta 2 non-catalytic subunit	PRKAB2	Homo sapiens
5566	protein kinase, cAMP-dependent, catalytic, alpha	PRKACA	Homo sapiens
5567	protein kinase, cAMP-dependent, catalytic, beta	PRKACB	Homo sapiens
5571	protein kinase, AMP-activated, gamma 1 non-catalytic subunit	PRKAG1	Homo sapiens
5576	protein kinase, cAMP-dependent, regulatory, type II, alpha	PRKAR2A	Homo sapiens
5577	protein kinase, cAMP-dependent, regulatory, type II, beta	PRKAR2B	Homo sapiens
5578	protein kinase C, alpha	PRKCA	Homo sapiens
5580	protein kinase C, delta	PRKCD	Homo sapiens
5581	protein kinase C, epsilon	PRKCE	Homo sapiens
5582	protein kinase C, gamma	PRKCG	Homo sapiens
5583	protein kinase C, eta	PRKCH	Homo sapiens
5584	protein kinase C, iota	PRKCI	Homo sapiens
5590	protein kinase C, zeta	PRKCZ	Homo sapiens
731751	similar to protein kinase, DNA-activated, catalytic polypeptide; protein kinase, DNA-activated, catalytic polypeptide	PRKDC	Homo sapiens
5591	similar to protein kinase, DNA-activated, catalytic polypeptide; protein kinase, DNA-activated, catalytic polypeptide	PRKDC	Homo sapiens
5593	protein kinase, cGMP-dependent, type II	PRKG2	Homo sapiens
5612	protein-kinase, interferon-inducible double stranded RNA dependent inhibitor, repressor of (P58 repressor)	PRKRIR	Homo sapiens

5617	prolactin	PRL	Homo sapiens
5618	prolactin receptor	PRLR	Homo sapiens
5619	protamine 1	PRM1	Homo sapiens
5621	prion protein	PRNP	Homo sapiens
5624	protein C (inactivator of coagulation factors Va and VIIIa)	PROC	Homo sapiens
10544	protein C receptor, endothelial (EPCR)	PROCR	Homo sapiens
5625	proline dehydrogenase (oxidase) 1	PRODH	Homo sapiens
8842	prominin 1	PROM1	Homo sapiens
5626	PROP paired-like homeobox 1	PROP1	Homo sapiens
5627	protein S (alpha)	PROS1	Homo sapiens
11212	proline synthetase co-transcribed homolog (bacterial)	PROSC	Homo sapiens
8858	protein Z, vitamin K-dependent plasma glycoprotein	PROZ	Homo sapiens
8559	PRP18 pre-mRNA processing factor 18 homolog (S. cerevisiae)	PRPF18	Homo sapiens
9129	PRP3 pre-mRNA processing factor 3 homolog (S. cerevisiae)	PRPF3	Homo sapiens
26121	PRP31 pre-mRNA processing factor 31 homolog (S. cerevisiae)	PRPF31	Homo sapiens
9128	PRP4 pre-mRNA processing factor 4 homolog (yeast)	PRPF4	Homo sapiens
8899	similar to hCG1820375; PRP4 pre-mRNA processing factor 4 homolog B (yeast)	PRPF4B	Homo sapiens
653155	similar to hCG1820375; PRP4 pre-mRNA processing factor 4 homolog B (yeast)	PRPF4B	Homo sapiens
5630	peripherin	PRPH	Homo sapiens
5634	phosphoribosyl pyrophosphate synthetase 2	PRPS2	Homo sapiens
5635	phosphoribosyl pyrophosphate synthetase-associated protein 1	PRPSAP1	Homo sapiens
5636	phosphoribosyl pyrophosphate synthetase-associated protein 2	PRPSAP2	Homo sapiens
5638	proline rich Gla (G-carboxyglutamic acid) 1	PRRG1	Homo sapiens
5639	proline rich Gla (G-carboxyglutamic acid) 2	PRRG2	Homo sapiens
5396	paired related homeobox 1	PRRX1	Homo sapiens
8492	protease, serine, 12 (neurotrypsin, motopsin)	PRSS12	Homo sapiens
10279	protease, serine, 16 (thymus)	PRSS16	Homo sapiens
11098	protease, serine, 23	PRSS23	Homo sapiens
5660	prosaposin	PSAP	Homo sapiens
5662	pleckstrin and Sec7 domain containing	PSD	Homo sapiens
23550	pleckstrin and Sec7 domain containing 4	PSD4	Homo sapiens
5663	presenilin 1	PSEN1	Homo sapiens
5664	presenilin 2 (Alzheimer disease 4)	PSEN2	Homo sapiens
11168	PC4 and SFRS1 interacting protein 1	PSIP1	Homo sapiens
5681	protein serine kinase H1	PSKH1	Homo sapiens
5682	proteasome (prosome, macropain) subunit, alpha type, 1	PSMA1	Homo sapiens
5683	proteasome (prosome, macropain) subunit, alpha type, 2	PSMA2	Homo sapiens
5684	proteasome (prosome, macropain) subunit, alpha type, 3	PSMA3	Homo sapiens
5685	proteasome (prosome, macropain) subunit, alpha type, 4	PSMA4	Homo sapiens
5686	proteasome (prosome, macropain) subunit, alpha type, 5	PSMA5	Homo sapiens
5687	proteasome (prosome, macropain) subunit, alpha type, 6	PSMA6	Homo sapiens
5688	proteasome (prosome, macropain) subunit, alpha type, 7	PSMA7	Homo sapiens
5689	proteasome (prosome, macropain) subunit, beta type, 1	PSMB1	Homo sapiens
5691	proteasome (prosome, macropain) subunit, beta type, 3	PSMB3	Homo sapiens

5693	proteasome (prosome, macropain) subunit, beta type, 5	PSMB5	Homo sapiens
5695	proteasome (prosome, macropain) subunit, beta type, 7	PSMB7	Homo sapiens
5696	proteasome (prosome, macropain) subunit, beta type, 8 (large multifunctional peptidase 7)	PSMB8	Homo sapiens
5701	proteasome (prosome, macropain) 26S subunit, ATPase, 2	PSMC2	Homo sapiens
5702	proteasome (prosome, macropain) 26S subunit, ATPase, 3	PSMC3	Homo sapiens
652826	similar to 26S protease regulatory subunit 6B (MIP224) (MB67-interacting protein) (TAT-binding protein 7) (TBP-7); proteasome (prosome, macropain) 26S subunit, ATPase, 4	PSMC4	Homo sapiens
5704	similar to 26S protease regulatory subunit 6B (MIP224) (MB67-interacting protein) (TAT-binding protein 7) (TBP-7); proteasome (prosome, macropain) 26S subunit, ATPase, 4	PSMC4	Homo sapiens
5705	proteasome (prosome, macropain) 26S subunit, ATPase, 5	PSMC5	Homo sapiens
5706	proteasome (prosome, macropain) 26S subunit, ATPase, 6	PSMC6	Homo sapiens
5707	proteasome (prosome, macropain) 26S subunit, non-ATPase, 1	PSMD1	Homo sapiens
5716	proteasome (prosome, macropain) 26S subunit, non-ATPase, 10	PSMD10	Homo sapiens
5717	proteasome (prosome, macropain) 26S subunit, non-ATPase, 11	PSMD11	Homo sapiens
5718	proteasome (prosome, macropain) 26S subunit, non-ATPase, 12	PSMD12	Homo sapiens
5719	proteasome (prosome, macropain) 26S subunit, non-ATPase, 13	PSMD13	Homo sapiens
10213	proteasome (prosome, macropain) 26S subunit, non-ATPase, 14	PSMD14	Homo sapiens
5709	proteasome (prosome, macropain) 26S subunit, non-ATPase, 3	PSMD3	Homo sapiens
5710	proteasome (prosome, macropain) 26S subunit, non-ATPase, 4	PSMD4	Homo sapiens
5711	proteasome (prosome, macropain) 26S subunit, non-ATPase, 5	PSMD5	Homo sapiens
5714	proteasome (prosome, macropain) 26S subunit, non-ATPase, 8	PSMD8	Homo sapiens
5721	proteasome (prosome, macropain) activator subunit 2 (PA28 beta)	PSME2	Homo sapiens
10197	proteasome (prosome, macropain) activator subunit 3 (PA28 gamma; Ki)	PSME3	Homo sapiens
23198	proteasome (prosome, macropain) activator subunit 4	PSME4	Homo sapiens
9491	proteasome (prosome, macropain) inhibitor subunit 1 (PI31)	PSMF1	Homo sapiens
9051	proline-serine-threonine phosphatase interacting protein 1	PSTPIP1	Homo sapiens
5725	polypyrimidine tract binding protein 1	PTBP1	Homo sapiens
9791	phosphatidylserine synthase 1	PTDSS1	Homo sapiens
5730	prostaglandin D2 synthase, hematopoietic; prostaglandin D2 synthase 21kDa (brain)	PTGDS	Homo sapiens
27306	prostaglandin D2 synthase, hematopoietic; prostaglandin D2 synthase 21kDa (brain)	PTGDS	Homo sapiens
5732	prostaglandin E receptor 2 (subtype EP2), 53kDa	PTGER2	Homo sapiens
5734	prostaglandin E receptor 4 (subtype EP4)	PTGER4	Homo sapiens
5739	prostaglandin I2 (prostacyclin) receptor (IP)	PTGIR	Homo sapiens
5740	prostaglandin I2 (prostacyclin) synthase	PTGIS	Homo sapiens
5742	prostaglandin-endoperoxide synthase 1 (prostaglandin G/H synthase and cyclooxygenase)	PTGS1	Homo sapiens
5743	prostaglandin-endoperoxide synthase 2 (prostaglandin G/H synthase and cyclooxygenase)	PTGS2	Homo sapiens
5744	parathyroid hormone-like hormone	PTHLH	Homo sapiens
5747	PTK2 protein tyrosine kinase 2	PTK2	Homo sapiens
2185	PTK2B protein tyrosine kinase 2 beta	PTK2B	Homo sapiens
5754	PTK7 protein tyrosine kinase 7	PTK7	Homo sapiens

7803	protein tyrosine phosphatase type IVA, member 1	PTP4A1	Homo sapiens
11156	protein tyrosine phosphatase type IVA, member 3	PTP4A3	Homo sapiens
9200	protein tyrosine phosphatase-like (proline instead of catalytic arginine), member A	PTPLA	Homo sapiens
5770	protein tyrosine phosphatase, non-receptor type 1	PTPN1	Homo sapiens
442113	protein tyrosine phosphatase, non-receptor type 11; similar to protein tyrosine phosphatase, non-receptor type 11	PTPN11	Homo sapiens
5781	protein tyrosine phosphatase, non-receptor type 11; similar to protein tyrosine phosphatase, non-receptor type 11	PTPN11	Homo sapiens
344593	protein tyrosine phosphatase, non-receptor type 11; similar to protein tyrosine phosphatase, non-receptor type 11	PTPN11	Homo sapiens
5782	protein tyrosine phosphatase, non-receptor type 12	PTPN12	Homo sapiens
5783	protein tyrosine phosphatase, non-receptor type 13 (APO-1/CD95 (Fas)-associated phosphatase)	PTPN13	Homo sapiens
5771	protein tyrosine phosphatase, non-receptor type 2	PTPN2	Homo sapiens
11099	protein tyrosine phosphatase, non-receptor type 21	PTPN21	Homo sapiens
26191	protein tyrosine phosphatase, non-receptor type 22 (lymphoid)	PTPN22	Homo sapiens
5774	protein tyrosine phosphatase, non-receptor type 3	PTPN3	Homo sapiens
5775	protein tyrosine phosphatase, non-receptor type 4 (megakaryocyte)	PTPN4	Homo sapiens
5777	protein tyrosine phosphatase, non-receptor type 6	PTPN6	Homo sapiens
5780	protein tyrosine phosphatase, non-receptor type 9	PTPN9	Homo sapiens
5787	protein tyrosine phosphatase, receptor type, B	PTPRB	Homo sapiens
5788	protein tyrosine phosphatase, receptor type, C	PTPRC	Homo sapiens
5790	protein tyrosine phosphatase, receptor type, C-associated protein	PTPRCAP	Homo sapiens
5789	protein tyrosine phosphatase, receptor type, D	PTPRD	Homo sapiens
5791	protein tyrosine phosphatase, receptor type, E	PTPRE	Homo sapiens
5792	protein tyrosine phosphatase, receptor type, F	PTPRF	Homo sapiens
5795	protein tyrosine phosphatase, receptor type, J	PTPRJ	Homo sapiens
5796	protein tyrosine phosphatase, receptor type, K	PTPRK	Homo sapiens
5798	protein tyrosine phosphatase, receptor type, N	PTPRN	Homo sapiens
5799	protein tyrosine phosphatase, receptor type, N polypeptide 2	PTPRN2	Homo sapiens
5801	protein tyrosine phosphatase, receptor type, R	PTPRR	Homo sapiens
5802	protein tyrosine phosphatase, receptor type, S	PTPRS	Homo sapiens
11122	protein tyrosine phosphatase, receptor type, T	PTPRT	Homo sapiens
10076	protein tyrosine phosphatase, receptor type, U	PTPRU	Homo sapiens
284119	polymerase I and transcript release factor	PTRF	Homo sapiens
5805	6-pyruvoyltetrahydropterin synthase	PTS	Homo sapiens
754	pituitary tumor-transforming 1 interacting protein	PTTG1IP	Homo sapiens
5813	purine-rich element binding protein A	PURA	Homo sapiens
80324	pseudouridylate synthase 1	PUS1	Homo sapiens
5816	parvalbumin	PVALB	Homo sapiens
5817	poliovirus receptor	PVR	Homo sapiens
29108	PYD and CARD domain containing	PYCARD	Homo sapiens
5836	phosphorylase, glycogen, liver	PYGL	Homo sapiens
5837	phosphorylase, glycogen, muscle	PYGM	Homo sapiens
5858	pregnancy-zone protein	PZP	Homo sapiens
5860	quinoid dihydropteridine reductase	QDPR	Homo sapiens

9444	quaking homolog, KH domain RNA binding (mouse)	QKI	Homo sapiens
25797	glutaminyl-peptide cyclotransferase	QPCT	Homo sapiens
23475	quinolinate phosphoribosyltransferase	QPRT	Homo sapiens
8766	RAB11A, member RAS oncogene family	RAB11A	Homo sapiens
9230	RAB11B, member RAS oncogene family	RAB11B	Homo sapiens
9727	RAB11 family interacting protein 3 (class II)	RAB11FIP3	Homo sapiens
51552	RAB14, member RAS oncogene family	RAB14	Homo sapiens
5861	RAB1A, member RAS oncogene family	RAB1A	Homo sapiens
57111	RAB25, member RAS oncogene family	RAB25	Homo sapiens
5873	RAB27A, member RAS oncogene family	RAB27A	Homo sapiens
5874	RAB27B, member RAS oncogene family	RAB27B	Homo sapiens
27314	RAB30, member RAS oncogene family	RAB30	Homo sapiens
11031	RAB31, member RAS oncogene family	RAB31	Homo sapiens
10981	RAB32, member RAS oncogene family	RAB32	Homo sapiens
9363	RAB33A, member RAS oncogene family	RAB33A	Homo sapiens
100130711	similar to hCG1778032; RAB35, member RAS oncogene family	RAB35	Homo sapiens
11021	similar to hCG1778032; RAB35, member RAS oncogene family	RAB35	Homo sapiens
9609	RAB36, member RAS oncogene family	RAB36	Homo sapiens
5864	RAB3A, member RAS oncogene family	RAB3A	Homo sapiens
10966	RAB40B, member RAS oncogene family	RAB40B	Homo sapiens
53916	RAB4B, member RAS oncogene family	RAB4B	Homo sapiens
5868	RAB5A, member RAS oncogene family	RAB5A	Homo sapiens
5878	RAB5C, member RAS oncogene family	RAB5C	Homo sapiens
5870	RAB6C, member RAS oncogene family; RAB6A, member RAS oncogene family; hypothetical LOC100130819; RAB6C-like	RAB6A	Homo sapiens
100130819	RAB6C, member RAS oncogene family; RAB6A, member RAS oncogene family; hypothetical LOC100130819; RAB6C-like	RAB6A	Homo sapiens
150786	RAB6C, member RAS oncogene family; RAB6A, member RAS oncogene family; hypothetical LOC100130819; RAB6C-like	RAB6A	Homo sapiens
84084	RAB6C, member RAS oncogene family; RAB6A, member RAS oncogene family; hypothetical LOC100130819; RAB6C-like	RAB6A	Homo sapiens
8934	RAB7, member RAS oncogene family-like 1	RAB7L1	Homo sapiens
4218	RAB8A, member RAS oncogene family	RAB8A	Homo sapiens
23637	RAB GTPase activating protein 1	RABGAP1	Homo sapiens
9910	RAB GTPase activating protein 1-like	RABGAP1L	Homo sapiens
5876	Rab geranylgeranyltransferase, beta subunit	RABGGTB	Homo sapiens
5877	RAB interacting factor	RABIF	Homo sapiens
5879	ras-related C3 botulinum toxin substrate 1 (rho family, small GTP binding protein Rac1)	RAC1	Homo sapiens
5880	ras-related C3 botulinum toxin substrate 2 (rho family, small GTP binding protein Rac2)	RAC2	Homo sapiens
5810	RAD1 homolog (S. pombe)	RAD1	Homo sapiens
5884	RAD17 homolog (S. pombe)	RAD17	Homo sapiens
5885	RAD21 homolog (S. pombe)	RAD21	Homo sapiens
5887	RAD23 homolog B (S. cerevisiae)	RAD23B	Homo sapiens
10111	RAD50 homolog (S. cerevisiae)	RAD50	Homo sapiens
5889	RAD51 homolog C (S. cerevisiae)	RAD51C	Homo sapiens

5890	RAD51-like 1 (S. cerevisiae)	RAD51L1	Homo sapiens
5893	RAD52 homolog (S. cerevisiae)	RAD52	Homo sapiens
8438	RAD54-like (S. cerevisiae)	RAD54L	Homo sapiens
8480	RAE1 RNA export 1 homolog (S. pombe)	RAE1	Homo sapiens
5894	v-raf-1 murine leukemia viral oncogene homolog 1	RAF1	Homo sapiens
5891	renal tumor antigen	RAGE	Homo sapiens
26064	retinoic acid induced 14	RAI14	Homo sapiens
5898	v-ral simian leukemia viral oncogene homolog A (ras related)	RALA	Homo sapiens
5899	v-ral simian leukemia viral oncogene homolog B (ras related; GTP binding protein)	RALB	Homo sapiens
100129773	hypothetical LOC100129773; ralA binding protein 1	RALBP1	Homo sapiens
10928	hypothetical LOC100129773; ralA binding protein 1	RALBP1	Homo sapiens
5900	ral guanine nucleotide dissociation stimulator	RALGDS	Homo sapiens
22913	RNA binding protein, autoantigenic (hnRNP-associated with lethal yellow homolog (mouse))	RALY	Homo sapiens
10267	receptor (G protein-coupled) activity modifying protein 1	RAMP1	Homo sapiens
5901	RAN, member RAS oncogene family	RAN	Homo sapiens
8498	RAN binding protein 3	RANBP3	Homo sapiens
26953	RAN binding protein 6	RANBP6	Homo sapiens
5905	Ran GTPase activating protein 1	RANGAP1	Homo sapiens
5906	RAP1A, member of RAS oncogene family	RAP1A	Homo sapiens
5910	RAP1, GTP-GDP dissociation stimulator 1	RAP1GDS1	Homo sapiens
5911	RAP2A, member of RAS oncogene family	RAP2A	Homo sapiens
5912	RAP2B, member of RAS oncogene family	RAP2B	Homo sapiens
2889	Rap guanine nucleotide exchange factor (GEF) 1	RAPGEF1	Homo sapiens
10411	Rap guanine nucleotide exchange factor (GEF) 3	RAPGEF3	Homo sapiens
11069	Rap guanine nucleotide exchange factor (GEF) 4	RAPGEF4	Homo sapiens
65059	Ras association (RalGDS/AF-6) and pleckstrin homology domains 1	RAPH1	Homo sapiens
5914	retinoic acid receptor, alpha	RARA	Homo sapiens
5918	retinoic acid receptor responder (tazarotene induced) 1	RARRES1	Homo sapiens
5919	retinoic acid receptor responder (tazarotene induced) 2	RARRES2	Homo sapiens
5920	retinoic acid receptor responder (tazarotene induced) 3	RARRES3	Homo sapiens
5917	arginyl-tRNA synthetase	RARS	Homo sapiens
5921	RAS p21 protein activator (GTPase activating protein) 1	RASA1	Homo sapiens
9462	RAS protein activator like 2	RASAL2	Homo sapiens
5923	Ras protein-specific guanine nucleotide-releasing factor 1	RASGRF1	Homo sapiens
10125	RAS guanyl releasing protein 1 (calcium and DAG-regulated)	RASGRP1	Homo sapiens
25780	RAS guanyl releasing protein 3 (calcium and DAG-regulated)	RASGRP3	Homo sapiens
11186	Ras association (RalGDS/AF-6) domain family member 1	RASSF1	Homo sapiens
9770	Ras association (RalGDS/AF-6) domain family member 2	RASSF2	Homo sapiens
5928	hypothetical LOC642954; retinoblastoma binding protein 4	RBBP4	Homo sapiens
642954	hypothetical LOC642954; retinoblastoma binding protein 4	RBBP4	Homo sapiens
5929	retinoblastoma binding protein 5	RBBP5	Homo sapiens
5931	retinoblastoma binding protein 7	RBBP7	Homo sapiens
5932	retinoblastoma binding protein 8	RBBP8	Homo sapiens
5933	retinoblastoma-like 1 (p107)	RBL1	Homo sapiens

8241	RNA binding motif protein 10	RBM10	Homo sapiens
84991	RNA binding motif protein 17	RBM17	Homo sapiens
5935	RNA binding motif (RNP1, RRM) protein 3	RBM3	Homo sapiens
5936	RNA binding motif protein 14; RNA binding motif protein 4	RBM4	Homo sapiens
10432	RNA binding motif protein 14; RNA binding motif protein 4	RBM4	Homo sapiens
9939	RNA binding motif protein 8A	RBM8A	Homo sapiens
23543	RNA binding motif protein 9	RBM9	Homo sapiens
27303	RNA binding motif, single stranded interacting protein	RBMS3	Homo sapiens
11030	RNA binding protein with multiple splicing	RBPMS	Homo sapiens
9986	RCE1 homolog, prenyl protein peptidase (S. cerevisiae)	RCE1	Homo sapiens
25898	ring finger and CHY zinc finger domain containing 1	RCHY1	Homo sapiens
5955	reticulocalbin 2, EF-hand calcium binding domain	RCN2	Homo sapiens
7936	RD RNA binding protein	RDBP	Homo sapiens
51109	retinol dehydrogenase 11 (all-trans/9-cis/11-cis)	RDH11	Homo sapiens
5962	radixin	RDX	Homo sapiens
8434	reversion-inducing-cysteine-rich protein with kazal motifs	RECK	Homo sapiens
9401	RecQ protein-like 4	RECQL4	Homo sapiens
5969	regenerating islet-derived 1 alpha; regenerating islet-derived 1 pseudogene	REG1A	Homo sapiens
5967	regenerating islet-derived 1 alpha; regenerating islet-derived 1 pseudogene	REG1A	Homo sapiens
5966	v-rel reticuloendotheliosis viral oncogene homolog (avian)	REL	Homo sapiens
5972	renin	REN	Homo sapiens
9185	RALBP1 associated Eps domain containing 2	REPS2	Homo sapiens
5980	REV3-like, catalytic subunit of DNA polymerase zeta (yeast)	REV3L	Homo sapiens
5982	replication factor C (activator 1) 2, 40kDa	RFC2	Homo sapiens
5983	replication factor C (activator 1) 3, 38kDa	RFC3	Homo sapiens
5984	replication factor C (activator 1) 4, 37kDa	RFC4	Homo sapiens
55312	riboflavin kinase	RFK	Homo sapiens
5990	regulatory factor X, 2 (influences HLA class II expression)	RFX2	Homo sapiens
5991	regulatory factor X, 3 (influences HLA class II expression)	RFX3	Homo sapiens
5993	regulatory factor X, 5 (influences HLA class II expression)	RFX5	Homo sapiens
8625	regulatory factor X-associated ankyrin-containing protein	RFXANK	Homo sapiens
5994	regulatory factor X-associated protein	RFXAP	Homo sapiens
23179	ral guanine nucleotide dissociation stimulator-like 1	RGL1	Homo sapiens
5863	ral guanine nucleotide dissociation stimulator-like 2	RGL2	Homo sapiens
9104	regucalcin (senescence marker protein-30)	RGN	Homo sapiens
5995	retinal G protein coupled receptor	RGR	Homo sapiens
8786	regulator of G-protein signaling 11	RGS11	Homo sapiens
6002	regulator of G-protein signaling 12	RGS12	Homo sapiens
6003	regulator of G-protein signaling 13	RGS13	Homo sapiens
6004	regulator of G-protein signaling 16	RGS16	Homo sapiens
10287	regulator of G-protein signaling 19	RGS19	Homo sapiens
5997	regulator of G-protein signaling 2, 24kDa	RGS2	Homo sapiens
8601	regulator of G-protein signaling 20	RGS20	Homo sapiens
5998	regulator of G-protein signaling 3	RGS3	Homo sapiens

5999	regulator of G-protein signaling 4	RGS4	Homo sapiens
8490	regulator of G-protein signaling 5	RGS5	Homo sapiens
9628	regulator of G-protein signaling 6	RGS6	Homo sapiens
6000	regulator of G-protein signaling 7	RGS7	Homo sapiens
6005	Rh-associated glycoprotein	RHAG	Homo sapiens
64285	rhomboid 5 homolog 1 (Drosophila)	RHBDF1	Homo sapiens
6009	Ras homolog enriched in brain	RHEB	Homo sapiens
388	ras homolog gene family, member B	RHOB	Homo sapiens
391	ras homolog gene family, member G (rho G)	RHOG	Homo sapiens
399	ras homolog gene family, member H	RHOH	Homo sapiens
26150	RIB43A domain with coiled-coils 2	RIBC2	Homo sapiens
9610	Ras and Rab interactor 1	RIN1	Homo sapiens
6015	ring finger protein 1	RING1	Homo sapiens
8780	RIO kinase 3 (yeast)	RIOK3	Homo sapiens
8737	receptor (TNFRSF)-interacting serine-threonine kinase 1	RIPK1	Homo sapiens
8767	receptor-interacting serine-threonine kinase 2	RIPK2	Homo sapiens
6014	Ras-like without CAAX 2	RIT2	Homo sapiens
6017	retinaldehyde binding protein 1	RLBP1	Homo sapiens
6013	relaxin 1	RLN1	Homo sapiens
6019	relaxin 2	RLN2	Homo sapiens
6035	ribonuclease, RNase A family, 1 (pancreatic)	RNASE1	Homo sapiens
6036	ribonuclease, RNase A family, 2 (liver, eosinophil-derived neurotoxin)	RNASE2	Homo sapiens
6039	ribonuclease, RNase A family, k6	RNASE6	Homo sapiens
10535	ribonuclease H2, subunit A	RNASEH2A	Homo sapiens
27289	Rho family GTPase 1	RND1	Homo sapiens
9921	ring finger protein 10	RNF10	Homo sapiens
51652	vacuolar protein sorting 24 homolog (S. cerevisiae); ring finger protein 103	RNF103	Homo sapiens
7844	vacuolar protein sorting 24 homolog (S. cerevisiae); ring finger protein 103	RNF103	Homo sapiens
79589	ring finger protein 128	RNF128	Homo sapiens
11342	ring finger protein 13	RNF13	Homo sapiens
11236	ring finger protein 139	RNF139	Homo sapiens
9604	ring finger protein 14	RNF14	Homo sapiens
26001	ring finger protein 167	RNF167	Homo sapiens
9810	ring finger protein 40	RNF40	Homo sapiens
10193	ring finger protein 41	RNF41	Homo sapiens
22838	ring finger protein 44	RNF44	Homo sapiens
6049	ring finger protein (C3H2C3 type) 6	RNF6	Homo sapiens
9616	ring finger protein 7	RNF7	Homo sapiens
642132	roundabout, axon guidance receptor, homolog 1 (Drosophila); similar to roundabout 1 isoform b	ROBO1	Homo sapiens
6091	roundabout, axon guidance receptor, homolog 1 (Drosophila); similar to roundabout 1 isoform b	ROBO1	Homo sapiens
6092	roundabout, axon guidance receptor, homolog 2 (Drosophila)	ROBO2	Homo sapiens
6093	similar to Rho-associated, coiled-coil containing protein kinase 1; Rho-associated, coiled-coil containing protein kinase 1	ROCK1	Homo sapiens

727758	similar to Rho-associated, coiled-coil containing protein kinase 1; Rho-associated, coiled-coil containing protein kinase 1	ROCK1	Homo sapiens
9991	ROD1 regulator of differentiation 1 (S. pombe)	ROD1	Homo sapiens
6094	retinal outer segment membrane protein 1	ROM1	Homo sapiens
4920	receptor tyrosine kinase-like orphan receptor 2	ROR2	Homo sapiens
6095	RAR-related orphan receptor A	RORA	Homo sapiens
6097	RAR-related orphan receptor C	RORC	Homo sapiens
6102	retinitis pigmentosa 2 (X-linked recessive)	RP2	Homo sapiens
6117	replication protein A1, 70kDa	RPA1	Homo sapiens
6118	replication protein A2, 32kDa	RPA2	Homo sapiens
6119	replication protein A3, 14kDa	RPA3	Homo sapiens
6120	rcRPE; ribulose-5-phosphate-3-epimerase	RPE	Homo sapiens
729020	rcRPE; ribulose-5-phosphate-3-epimerase	RPE	Homo sapiens
6103	retinitis pigmentosa GTPase regulator	RPGR	Homo sapiens
22934	ribose 5-phosphate isomerase A	RPIA	Homo sapiens
6135	ribosomal protein L11	RPL11	Homo sapiens
100129424	ribosomal protein L19; ribosomal protein L19 pseudogene 12	RPL19	Homo sapiens
6143	ribosomal protein L19; ribosomal protein L19 pseudogene 12	RPL19	Homo sapiens
6155	ribosomal protein L27	RPL27	Homo sapiens
6158	ribosomal protein L28	RPL28	Homo sapiens
6166	ribosomal protein L36a-like	RPL36AL	Homo sapiens
6184	ribophorin I	RPN1	Homo sapiens
6185	ribophorin II	RPN2	Homo sapiens
11102	ribonuclease P/MRP 14kDa subunit	RPP14	Homo sapiens
10556	ribonuclease P/MRP 30kDa subunit	RPP30	Homo sapiens
645630	ribosomal protein S13 pseudogene 8; ribosomal protein S13; ribosomal protein S13 pseudogene 2	RPS13	Homo sapiens
6207	ribosomal protein S13 pseudogene 8; ribosomal protein S13; ribosomal protein S13 pseudogene 2	RPS13	Homo sapiens
729236	ribosomal protein S13 pseudogene 8; ribosomal protein S13; ribosomal protein S13 pseudogene 2	RPS13	Homo sapiens
6208	ribosomal protein S14	RPS14	Homo sapiens
6228	ribosomal protein S23	RPS23	Homo sapiens
6229	ribosomal protein S24	RPS24	Homo sapiens
392256	ribosomal protein S26 pseudogene 38; ribosomal protein S26 pseudogene 39; ribosomal protein S26 pseudogene 35; ribosomal protein S26 pseudogene 31; ribosomal protein S26 pseudogene 20; ribosomal protein S26 pseudogene 54; ribosomal protein S26 pseudogene 2; ribosomal protein S26 pseudogene 53; ribosomal protein S26 pseudogene 25; ribosomal protein S26 pseudogene 50; ribosomal protein S26 pseudogene 6; ribosomal protein S26 pseudogene 8; ribosomal protein S26	RPS26	Homo sapiens
646753	ribosomal protein S26 pseudogene 38; ribosomal protein S26 pseudogene 39; ribosomal protein S26 pseudogene 35; ribosomal protein S26 pseudogene 31; ribosomal protein S26 pseudogene 20; ribosomal protein S26 pseudogene 54; ribosomal protein S26 pseudogene 2; ribosomal protein S26 pseudogene 53; ribosomal protein S26 pseudogene 25; ribosomal protein S26 pseudogene 50; ribosomal protein S26 pseudogene 6; ribosomal protein S26	RPS26	Homo sapiens

	pseudogene 8; ribosomal protein S26		
644166	ribosomal protein S26 pseudogene 38; ribosomal protein S26 pseudogene 39; ribosomal protein S26 pseudogene 35; ribosomal protein S26 pseudogene 31; ribosomal protein S26 pseudogene 20; ribosomal protein S26 pseudogene 54; ribosomal protein S26 pseudogene 2; ribosomal protein S26 pseudogene 53; ribosomal protein S26 pseudogene 25; ribosomal protein S26 pseudogene 50; ribosomal protein S26 pseudogene 6; ribosomal protein S26 pseudogene 8; ribosomal protein S26	RPS26	Homo sapiens
100129552	ribosomal protein S26 pseudogene 38; ribosomal protein S26 pseudogene 39; ribosomal protein S26 pseudogene 35; ribosomal protein S26 pseudogene 31; ribosomal protein S26 pseudogene 20; ribosomal protein S26 pseudogene 54; ribosomal protein S26 pseudogene 2; ribosomal protein S26 pseudogene 53; ribosomal protein S26 pseudogene 25; ribosomal protein S26 pseudogene 50; ribosomal protein S26 pseudogene 6; ribosomal protein S26 pseudogene 8; ribosomal protein S26	RPS26	Homo sapiens
6231	ribosomal protein S26 pseudogene 38; ribosomal protein S26 pseudogene 39; ribosomal protein S26 pseudogene 35; ribosomal protein S26 pseudogene 31; ribosomal protein S26 pseudogene 20; ribosomal protein S26 pseudogene 54; ribosomal protein S26 pseudogene 2; ribosomal protein S26 pseudogene 53; ribosomal protein S26 pseudogene 25; ribosomal protein S26 pseudogene 50; ribosomal protein S26 pseudogene 6; ribosomal protein S26 pseudogene 8; ribosomal protein S26	RPS26	Homo sapiens
100128168	ribosomal protein S26 pseudogene 38; ribosomal protein S26 pseudogene 39; ribosomal protein S26 pseudogene 35; ribosomal protein S26 pseudogene 31; ribosomal protein S26 pseudogene 20; ribosomal protein S26 pseudogene 54; ribosomal protein S26 pseudogene 2; ribosomal protein S26 pseudogene 53; ribosomal protein S26 pseudogene 25; ribosomal protein S26 pseudogene 50; ribosomal protein S26 pseudogene 6; ribosomal protein S26 pseudogene 8; ribosomal protein S26	RPS26	Homo sapiens
644934	ribosomal protein S26 pseudogene 38; ribosomal protein S26 pseudogene 39; ribosomal protein S26 pseudogene 35; ribosomal protein S26 pseudogene 31; ribosomal protein S26 pseudogene 20; ribosomal protein S26 pseudogene 54; ribosomal protein S26 pseudogene 2; ribosomal protein S26 pseudogene 53; ribosomal protein S26 pseudogene 25; ribosomal protein S26 pseudogene 50; ribosomal protein S26 pseudogene 6; ribosomal protein S26 pseudogene 8; ribosomal protein S26	RPS26	Homo sapiens
645979	ribosomal protein S26 pseudogene 38; ribosomal protein S26 pseudogene 39; ribosomal protein S26 pseudogene 35; ribosomal protein S26 pseudogene 31; ribosomal protein S26 pseudogene 20; ribosomal protein S26 pseudogene 54; ribosomal protein S26 pseudogene 2; ribosomal protein S26 pseudogene 53; ribosomal protein S26 pseudogene 25; ribosomal protein S26 pseudogene 50; ribosomal protein S26 pseudogene 6; ribosomal protein S26 pseudogene 8; ribosomal protein S26	RPS26	Homo sapiens
100131971	ribosomal protein S26 pseudogene 38; ribosomal protein S26 pseudogene 39; ribosomal protein S26 pseudogene 35; ribosomal protein S26 pseudogene 31; ribosomal protein S26 pseudogene 20; ribosomal protein S26 pseudogene 54; ribosomal protein S26	RPS26	Homo sapiens

	pseudogene 2; ribosomal protein S26 pseudogene 53; ribosomal protein S26 pseudogene 25; ribosomal protein S26 pseudogene 50; ribosomal protein S26 pseudogene 6; ribosomal protein S26 pseudogene 8; ribosomal protein S26		
644191	ribosomal protein S26 pseudogene 38; ribosomal protein S26 pseudogene 39; ribosomal protein S26 pseudogene 35; ribosomal protein S26 pseudogene 31; ribosomal protein S26 pseudogene 20; ribosomal protein S26 pseudogene 54; ribosomal protein S26 pseudogene 2; ribosomal protein S26 pseudogene 53; ribosomal protein S26 pseudogene 25; ribosomal protein S26 pseudogene 50; ribosomal protein S26 pseudogene 6; ribosomal protein S26 pseudogene 8; ribosomal protein S26	RPS26	Homo sapiens
441377	ribosomal protein S26 pseudogene 38; ribosomal protein S26 pseudogene 39; ribosomal protein S26 pseudogene 35; ribosomal protein S26 pseudogene 31; ribosomal protein S26 pseudogene 20; ribosomal protein S26 pseudogene 54; ribosomal protein S26 pseudogene 2; ribosomal protein S26 pseudogene 53; ribosomal protein S26 pseudogene 25; ribosomal protein S26 pseudogene 50; ribosomal protein S26 pseudogene 6; ribosomal protein S26 pseudogene 8; ribosomal protein S26	RPS26	Homo sapiens
728823	ribosomal protein S26 pseudogene 38; ribosomal protein S26 pseudogene 39; ribosomal protein S26 pseudogene 35; ribosomal protein S26 pseudogene 31; ribosomal protein S26 pseudogene 20; ribosomal protein S26 pseudogene 54; ribosomal protein S26 pseudogene 2; ribosomal protein S26 pseudogene 53; ribosomal protein S26 pseudogene 25; ribosomal protein S26 pseudogene 50; ribosomal protein S26 pseudogene 6; ribosomal protein S26 pseudogene 8; ribosomal protein S26	RPS26	Homo sapiens
728937	ribosomal protein S26 pseudogene 38; ribosomal protein S26 pseudogene 39; ribosomal protein S26 pseudogene 35; ribosomal protein S26 pseudogene 31; ribosomal protein S26 pseudogene 20; ribosomal protein S26 pseudogene 54; ribosomal protein S26 pseudogene 2; ribosomal protein S26 pseudogene 53; ribosomal protein S26 pseudogene 25; ribosomal protein S26 pseudogene 50; ribosomal protein S26 pseudogene 6; ribosomal protein S26 pseudogene 8; ribosomal protein S26	RPS26	Homo sapiens
728590	ribosomal protein S27a pseudogene 12; ribosomal protein S27a; ribosomal protein S27a pseudogene 11; ribosomal protein S27a pseudogene 16	RPS27A	Homo sapiens
100130446	ribosomal protein S27a pseudogene 12; ribosomal protein S27a; ribosomal protein S27a pseudogene 11; ribosomal protein S27a pseudogene 16	RPS27A	Homo sapiens
643358	ribosomal protein S27a pseudogene 12; ribosomal protein S27a; ribosomal protein S27a pseudogene 11; ribosomal protein S27a pseudogene 16	RPS27A	Homo sapiens
6233	ribosomal protein S27a pseudogene 12; ribosomal protein S27a; ribosomal protein S27a pseudogene 11; ribosomal protein S27a pseudogene 16	RPS27A	Homo sapiens
440991	ribosomal protein S3 pseudogene 3; ribosomal protein S3	RPS3	Homo sapiens
6188	ribosomal protein S3 pseudogene 3; ribosomal protein S3	RPS3	Homo sapiens
6192	ribosomal protein S4, Y-linked 1	RPS4Y1	Homo sapiens
6193	ribosomal protein S5	RPS5	Homo sapiens

729389	ribosomal protein S6 pseudogene 25; ribosomal protein S6; ribosomal protein S6 pseudogene 1	RPS6	Homo sapiens
440086	ribosomal protein S6 pseudogene 25; ribosomal protein S6; ribosomal protein S6 pseudogene 1	RPS6	Homo sapiens
6194	ribosomal protein S6 pseudogene 25; ribosomal protein S6; ribosomal protein S6 pseudogene 1	RPS6	Homo sapiens
6195	ribosomal protein S6 kinase, 90kDa, polypeptide 1	RPS6KA1	Homo sapiens
6196	ribosomal protein S6 kinase, 90kDa, polypeptide 2; hypothetical LOC100127984	RPS6KA2	Homo sapiens
100127984	ribosomal protein S6 kinase, 90kDa, polypeptide 2; hypothetical LOC100127984	RPS6KA2	Homo sapiens
8986	ribosomal protein S6 kinase, 90kDa, polypeptide 4	RPS6KA4	Homo sapiens
6198	ribosomal protein S6 kinase, 70kDa, polypeptide 1	RPS6KB1	Homo sapiens
6199	ribosomal protein S6 kinase, 70kDa, polypeptide 2	RPS6KB2	Homo sapiens
6203	ribosomal protein S9; ribosomal protein S9 pseudogene 4	RPS9	Homo sapiens
388556	ribosomal protein S9; ribosomal protein S9 pseudogene 4	RPS9	Homo sapiens
9125	RCD1 required for cell differentiation1 homolog (S. pombe)	RQCD1	Homo sapiens
10670	Ras-related GTP binding A	RRAGA	Homo sapiens
10325	Ras-related GTP binding B	RRAGB	Homo sapiens
58528	Ras-related GTP binding D	RRAGD	Homo sapiens
6237	related RAS viral (r-ras) oncogene homolog	RRAS	Homo sapiens
100133211	related RAS viral (r-ras) oncogene homolog 2; similar to related RAS viral (r-ras) oncogene homolog 2	RRAS2	Homo sapiens
22800	related RAS viral (r-ras) oncogene homolog 2; similar to related RAS viral (r-ras) oncogene homolog 2	RRAS2	Homo sapiens
6238	ribosome binding protein 1 homolog 180kDa (dog)	RRBP1	Homo sapiens
6240	ribonucleotide reductase M1	RRM1	Homo sapiens
6241	ribonucleotide reductase M2 polypeptide	RRM2	Homo sapiens
50484	ribonucleotide reductase M2 B (TP53 inducible)	RRM2B	Homo sapiens
54700	RRN3 RNA polymerase I transcription factor homolog (S. cerevisiae)	RRN3	Homo sapiens
54665	round spermatid basic protein 1	RSBN1	Homo sapiens
6251	Ras suppressor protein 1	RSU1	Homo sapiens
8634	RNA terminal phosphate cyclase domain 1	RTCD1	Homo sapiens
6242	rhotekin	RTKN	Homo sapiens
6252	reticulon 1	RTN1	Homo sapiens
6253	reticulon 2	RTN2	Homo sapiens
57142	reticulon 4	RTN4	Homo sapiens
861	runt-related transcription factor 1	RUNX1	Homo sapiens
860	runt-related transcription factor 2	RUNX2	Homo sapiens
864	runt-related transcription factor 3	RUNX3	Homo sapiens
8607	RuvB-like 1 (E. coli)	RUVBL1	Homo sapiens
6258	retinoid X receptor, gamma	RXRG	Homo sapiens
23429	RING1 and YY1 binding protein	RYBP	Homo sapiens
6263	ryanodine receptor 3	RYR3	Homo sapiens
6271	S100 calcium binding protein A1	S100A1	Homo sapiens
729659	S100 calcium binding protein A11; S100 calcium binding protein A11 pseudogene	S100A11	Homo sapiens
6282	S100 calcium binding protein A11; S100 calcium binding protein A11	S100A11	Homo sapiens

	pseudogene		
6283	S100 calcium binding protein A12	S100A12	Homo sapiens
6284	S100 calcium binding protein A13	S100A13	Homo sapiens
6273	S100 calcium binding protein A2	S100A2	Homo sapiens
6275	S100 calcium binding protein A4	S100A4	Homo sapiens
6277	S100 calcium binding protein A6	S100A6	Homo sapiens
6279	S100 calcium binding protein A8	S100A8	Homo sapiens
6280	S100 calcium binding protein A9	S100A9	Homo sapiens
6285	S100 calcium binding protein B	S100B	Homo sapiens
26278	spastic ataxia of Charlevoix-Saguenay (sacsin)	SACS	Homo sapiens
25939	SAM domain and HD domain 1	SAMHD1	Homo sapiens
8819	Sin3A-associated protein, 30kDa	SAP30	Homo sapiens
1757	sarcosine dehydrogenase	SARDH	Homo sapiens
6301	seryl-tRNA synthetase	SARS	Homo sapiens
9092	squamous cell carcinoma antigen recognized by T cells	SART1	Homo sapiens
9733	squamous cell carcinoma antigen recognized by T cells 3	SART3	Homo sapiens
6304	SATB homeobox 1	SATB1	Homo sapiens
23314	SATB homeobox 2	SATB2	Homo sapiens
100133234	SET binding factor 1; SET binding factor 1 pseudogene 1	SBF1	Homo sapiens
6305	SET binding factor 1; SET binding factor 1 pseudogene 1	SBF1	Homo sapiens
6307	sterol-C4-methyl oxidase-like	SC4MOL	Homo sapiens
6309	sterol-C5-desaturase (ERG3 delta-5-desaturase homolog, S. cerevisiae)-like	SC5DL	Homo sapiens
9522	secretory carrier membrane protein 1	SCAMP1	Homo sapiens
54581	SCAN domain containing 2 pseudogene	SCAND2	Homo sapiens
949	scavenger receptor class B, member 1	SCARB1	Homo sapiens
950	scavenger receptor class B, member 2	SCARB2	Homo sapiens
6319	stearoyl-CoA desaturase (delta-9-desaturase)	SCD	Homo sapiens
23256	sec1 family domain containing 1	SCFD1	Homo sapiens
7857	secretogranin II (chromogranin C)	SCG2	Homo sapiens
7356	secretoglobin, family 1A, member 1 (uteroglobin)	SCGB1A1	Homo sapiens
4246	secretoglobin, family 2A, member 1	SCGB2A1	Homo sapiens
4250	secretoglobin, family 2A, member 2	SCGB2A2	Homo sapiens
10590	secretagogin, EF-hand calcium binding protein	SCGN	Homo sapiens
29970	schwannomin interacting protein 1	SCHIP1	Homo sapiens
85477	scinderin	SCIN	Homo sapiens
10389	sex comb on midleg-like 2 (Drosophila)	SCML2	Homo sapiens
6324	sodium channel, voltage-gated, type I, beta	SCN1B	Homo sapiens
6335	sodium channel, voltage-gated, type IX, alpha subunit	SCN9A	Homo sapiens
6337	sodium channel, nonvoltage-gated 1 alpha	SCNN1A	Homo sapiens
6338	sodium channel, nonvoltage-gated 1, beta	SCNN1B	Homo sapiens
6339	sodium channel, nonvoltage-gated 1, delta	SCNN1D	Homo sapiens
6342	sterol carrier protein 2	SCP2	Homo sapiens
6382	syndecan 1	SDC1	Homo sapiens
6383	syndecan 2	SDC2	Homo sapiens
9672	syndecan 3	SDC3	Homo sapiens

6385	syndecan 4	SDC4	Homo sapiens
6386	syndecan binding protein (syntenin)	SDCBP	Homo sapiens
6388	stromal cell-derived factor 2	SDF2	Homo sapiens
6389	succinate dehydrogenase complex, subunit A, flavoprotein (Fp)	SDHA	Homo sapiens
6390	succinate dehydrogenase complex, subunit B, iron sulfur (Ip)	SDHB	Homo sapiens
6391	succinate dehydrogenase complex, subunit C, integral membrane protein, 15kDa	SDHC	Homo sapiens
730351	sidekick homolog 1, cell adhesion molecule (chicken); hypothetical LOC730351	SDK1	Homo sapiens
221935	sidekick homolog 1, cell adhesion molecule (chicken); hypothetical LOC730351	SDK1	Homo sapiens
10993	serine dehydratase	SDS	Homo sapiens
10483	Sec23 homolog B (S. cerevisiae)	SEC23B	Homo sapiens
11196	SEC23 interacting protein	SEC23IP	Homo sapiens
10427	SEC24 family, member B (S. cerevisiae)	SEC24B	Homo sapiens
9871	SEC24 family, member D (S. cerevisiae)	SEC24D	Homo sapiens
10952	Sec61 beta subunit	SEC61B	Homo sapiens
23480	Sec61 gamma subunit	SEC61G	Homo sapiens
6398	secreted and transmembrane 1	SECTM1	Homo sapiens
6400	sel-1 suppressor of lin-12-like (C. elegans)	SEL1L	Homo sapiens
6401	selectin E	SELE	Homo sapiens
6402	selectin L	SELL	Homo sapiens
6403	selectin P (granule membrane protein 140kDa, antigen CD62)	SELP	Homo sapiens
10371	sema domain, immunoglobulin domain (Ig), short basic domain, secreted, (semaphorin) 3A	SEMA3A	Homo sapiens
10512	sema domain, immunoglobulin domain (Ig), short basic domain, secreted, (semaphorin) 3C	SEMA3C	Homo sapiens
6405	sema domain, immunoglobulin domain (Ig), short basic domain, secreted, (semaphorin) 3F	SEMA3F	Homo sapiens
10505	sema domain, immunoglobulin domain (Ig), transmembrane domain (TM) and short cytoplasmic domain, (semaphorin) 4F	SEMA4F	Homo sapiens
57556	sema domain, transmembrane domain (TM), and cytoplasmic domain, (semaphorin) 6A	SEMA6A	Homo sapiens
6406	semenogelin I	SEMG1	Homo sapiens
6407	semenogelin II	SEMG2	Homo sapiens
205564	SUMO1/sentrin specific peptidase 5	SENP5	Homo sapiens
26054	SUMO1/sentrin specific peptidase 6	SENP6	Homo sapiens
22928	selenophosphate synthetase 2	SEPHS2	Homo sapiens
6414	selenoprotein P, plasma, 1	SEPP1	Homo sapiens
6415	selenoprotein W, 1	SEPW1	Homo sapiens
12	serpin peptidase inhibitor, clade A (alpha-1 antiproteinase, antitrypsin), member 3	SERPINA3	Homo sapiens
5267	serpin peptidase inhibitor, clade A (alpha-1 antiproteinase, antitrypsin), member 4	SERPINA4	Homo sapiens
5104	serpin peptidase inhibitor, clade A (alpha-1 antiproteinase, antitrypsin), member 5	SERPINA5	Homo sapiens
866	serpin peptidase inhibitor, clade A (alpha-1 antiproteinase, antitrypsin), member 6	SERPINA6	Homo sapiens
1992	serpin peptidase inhibitor, clade B (ovalbumin), member 1	SERPINB1	Homo sapiens

5055	serpin peptidase inhibitor, clade B (ovalbumin), member 2	SERPINB2	Homo sapiens
5269	serpin peptidase inhibitor, clade B (ovalbumin), member 6	SERPINB6	Homo sapiens
8710	serpin peptidase inhibitor, clade B (ovalbumin), member 7	SERPINB7	Homo sapiens
5271	serpin peptidase inhibitor, clade B (ovalbumin), member 8	SERPINB8	Homo sapiens
5272	serpin peptidase inhibitor, clade B (ovalbumin), member 9	SERPINB9	Homo sapiens
462	serpin peptidase inhibitor, clade C (antithrombin), member 1	SERPINC1	Homo sapiens
5176	serpin peptidase inhibitor, clade F (alpha-2 antiplasmin, pigment epithelium derived factor), member 1	SERPINF1	Homo sapiens
710	serpin peptidase inhibitor, clade G (C1 inhibitor), member 1	SERPING1	Homo sapiens
871	serpin peptidase inhibitor, clade H (heat shock protein 47), member 1, (collagen binding protein 1)	SERPINH1	Homo sapiens
5274	serpin peptidase inhibitor, clade I (neuroserpin), member 1	SERPINI1	Homo sapiens
27244	sestrin 1	SESN1	Homo sapiens
9869	SET domain, bifurcated 1	SETDB1	Homo sapiens
23544	seizure related 6 homolog (mouse)-like	SEZ6L	Homo sapiens
7536	splicing factor 1	SF1	Homo sapiens
10291	splicing factor 3a, subunit 1, 120kDa	SF3A1	Homo sapiens
8175	splicing factor 3a, subunit 2, 66kDa	SF3A2	Homo sapiens
23451	splicing factor 3b, subunit 1, 155kDa	SF3B1	Homo sapiens
10992	splicing factor 3b, subunit 2, 145kDa	SF3B2	Homo sapiens
23450	splicing factor 3b, subunit 3, 130kDa	SF3B3	Homo sapiens
10262	splicing factor 3b, subunit 4, 49kDa	SF3B4	Homo sapiens
51460	Scm-like with four mbt domains 1	SFMBT1	Homo sapiens
6421	splicing factor proline/glutamine-rich (polypyrimidine tract binding protein associated)	SFPQ	Homo sapiens
6422	secreted frizzled-related protein 1	SFRP1	Homo sapiens
6424	secreted frizzled-related protein 4	SFRP4	Homo sapiens
6425	secreted frizzled-related protein 5	SFRP5	Homo sapiens
9295	splicing factor, arginine/serine-rich 11	SFRS11	Homo sapiens
140890	splicing factor, arginine/serine-rich 12	SFRS12	Homo sapiens
9169	splicing factor, arginine/serine-rich 2, interacting protein	SFRS2IP	Homo sapiens
6430	splicing factor, arginine/serine-rich 5	SFRS5	Homo sapiens
8683	splicing factor, arginine/serine-rich 9	SFRS9	Homo sapiens
6439	surfactant protein B	SFTPB	Homo sapiens
6440	surfactant protein C	SFTPC	Homo sapiens
6441	surfactant protein D	SFTPD	Homo sapiens
6442	sarcoglycan, alpha (50kDa dystrophin-associated glycoprotein)	SGCA	Homo sapiens
6444	sarcoglycan, delta (35kDa dystrophin-associated glycoprotein)	SGCD	Homo sapiens
8910	sarcoglycan, epsilon	SGCE	Homo sapiens
6445	sarcoglycan, gamma (35kDa dystrophin-associated glycoprotein)	SGCG	Homo sapiens
8879	sphingosine-1-phosphate lyase 1	SGPL1	Homo sapiens
4068	SH2 domain protein 1A	SH2D1A	Homo sapiens
9047	SH2 domain protein 2A	SH2D2A	Homo sapiens
6450	SH3 domain binding glutamic acid-rich protein	SH3BGR	Homo sapiens
6452	SH3-domain binding protein 2	SH3BP2	Homo sapiens
6455	SH3-domain GRB2-like 1	SH3GL1	Homo sapiens
6456	SH3-domain GRB2-like 2	SH3GL2	Homo sapiens

22941	SH3 and multiple ankyrin repeat domains 2	SHANK2	Homo sapiens
6462	sex hormone-binding globulin	SHBG	Homo sapiens
6464	SHC (Src homology 2 domain containing) transforming protein 1	SHC1	Homo sapiens
53358	SHC (Src homology 2 domain containing) transforming protein 3	SHC3	Homo sapiens
7979	split hand/foot malformation (ectrodactyly) type 1	SHFM1	Homo sapiens
6470	serine hydroxymethyltransferase 1 (soluble)	SHMT1	Homo sapiens
6477	seven in absentia homolog 1 (Drosophila)	SIAH1	Homo sapiens
6478	seven in absentia homolog 2 (Drosophila)	SIAH2	Homo sapiens
946	sialic acid binding Ig-like lectin 6	SIGLEC6	Homo sapiens
6490	silver homolog (mouse)	SILV	Homo sapiens
6493	single-minded homolog 2 (Drosophila)	SIM2	Homo sapiens
8487	survival of motor neuron protein interacting protein 1	SIP1	Homo sapiens
6494	signal-induced proliferation-associated 1	SIPA1	Homo sapiens
22933	sirtuin (silent mating type information regulation 2 homolog) 2 (S. cerevisiae)	SIRT2	Homo sapiens
23408	sirtuin (silent mating type information regulation 2 homolog) 5 (S. cerevisiae)	SIRT5	Homo sapiens
6495	SIX homeobox 1	SIX1	Homo sapiens
51804	SIX homeobox 4	SIX4	Homo sapiens
6498	SKI-like oncogene	SKIL	Homo sapiens
6502	S-phase kinase-associated protein 2 (p45)	SKP2	Homo sapiens
6503	Src-like-adaptor	SLA	Homo sapiens
84174	Src-like-adaptor 2	SLA2	Homo sapiens
6504	signaling lymphocytic activation molecule family member 1	SLAMF1	Homo sapiens
57823	SLAM family member 7	SLAMF7	Homo sapiens
7884	stem-loop binding protein	SLBP	Homo sapiens
6554	solute carrier family 10 (sodium/bile acid cotransporter family), member 1	SLC10A1	Homo sapiens
6555	solute carrier family 10 (sodium/bile acid cotransporter family), member 2	SLC10A2	Homo sapiens
6556	solute carrier family 11 (proton-coupled divalent metal ion transporters), member 1	SLC11A1	Homo sapiens
4891	solute carrier family 11 (proton-coupled divalent metal ion transporters), member 2	SLC11A2	Homo sapiens
6558	solute carrier family 12 (sodium/potassium/chloride transporters), member 2	SLC12A2	Homo sapiens
64849	solute carrier family 13 (sodium-dependent dicarboxylate transporter), member 3	SLC13A3	Homo sapiens
6563	solute carrier family 14 (urea transporter), member 1 (Kidd blood group)	SLC14A1	Homo sapiens
6564	solute carrier family 15 (oligopeptide transporter), member 1	SLC15A1	Homo sapiens
6566	solute carrier family 16, member 1 (monocarboxylic acid transporter 1)	SLC16A1	Homo sapiens
6567	solute carrier family 16, member 2 (monocarboxylic acid transporter 8)	SLC16A2	Homo sapiens
9123	solute carrier family 16, member 3 (monocarboxylic acid transporter 4)	SLC16A3	Homo sapiens
9122	solute carrier family 16, member 4 (monocarboxylic acid transporter 5)	SLC16A4	Homo sapiens

100133772	similar to MCT; solute carrier family 16, member 5 (monocarboxylic acid transporter 6)	SLC16A5	Homo sapiens
9121	similar to MCT; solute carrier family 16, member 5 (monocarboxylic acid transporter 6)	SLC16A5	Homo sapiens
440459	solute carrier family 16, member 6 (monocarboxylic acid transporter 7); similar to solute carrier family 16, member 6	SLC16A6	Homo sapiens
9120	solute carrier family 16, member 6 (monocarboxylic acid transporter 7); similar to solute carrier family 16, member 6	SLC16A6	Homo sapiens
9194	solute carrier family 16, member 7 (monocarboxylic acid transporter 2)	SLC16A7	Homo sapiens
6568	solute carrier family 17 (sodium phosphate), member 1	SLC17A1	Homo sapiens
10246	solute carrier family 17 (sodium phosphate), member 2	SLC17A2	Homo sapiens
10786	solute carrier family 17 (sodium phosphate), member 3	SLC17A3	Homo sapiens
10050	solute carrier family 17 (sodium phosphate), member 4	SLC17A4	Homo sapiens
10560	solute carrier family 19 (thiamine transporter), member 2	SLC19A2	Homo sapiens
6505	solute carrier family 1 (neuronal/epithelial high affinity glutamate transporter, system Xag), member 1	SLC1A1	Homo sapiens
6507	solute carrier family 1 (glial high affinity glutamate transporter), member 3	SLC1A3	Homo sapiens
6509	solute carrier family 1 (glutamate/neutral amino acid transporter), member 4	SLC1A4	Homo sapiens
6510	solute carrier family 1 (neutral amino acid transporter), member 5	SLC1A5	Homo sapiens
6574	solute carrier family 20 (phosphate transporter), member 1	SLC20A1	Homo sapiens
6575	solute carrier family 20 (phosphate transporter), member 2	SLC20A2	Homo sapiens
6580	solute carrier family 22 (organic cation transporter), member 1	SLC22A1	Homo sapiens
9389	solute carrier family 22, member 14	SLC22A14	Homo sapiens
5002	solute carrier family 22, member 18	SLC22A18	Homo sapiens
6582	solute carrier family 22 (organic cation transporter), member 2	SLC22A2	Homo sapiens
6581	solute carrier family 22 (extraneuronal monoamine transporter), member 3	SLC22A3	Homo sapiens
6584	solute carrier family 22 (organic cation/carnitine transporter), member 5	SLC22A5	Homo sapiens
9356	solute carrier family 22 (organic anion transporter), member 6	SLC22A6	Homo sapiens
9962	solute carrier family 23 (nucleobase transporters), member 2	SLC23A2	Homo sapiens
10165	solute carrier family 25, member 13 (citrin)	SLC25A13	Homo sapiens
9016	solute carrier family 25 (mitochondrial carrier, brain), member 14	SLC25A14	Homo sapiens
10478	solute carrier family 25 (mitochondrial carrier; peroxisomal membrane protein, 34kDa), member 17	SLC25A17	Homo sapiens
788	solute carrier family 25 (carnitine/acylcarnitine translocase), member 20	SLC25A20	Homo sapiens
5250	solute carrier family 25 (mitochondrial carrier; phosphate carrier), member 3	SLC25A3	Homo sapiens
291	solute carrier family 25 (mitochondrial carrier; adenine nucleotide translocator), member 4	SLC25A4	Homo sapiens
1836	solute carrier family 26 (sulfate transporter), member 2	SLC26A2	Homo sapiens
1811	solute carrier family 26, member 3	SLC26A3	Homo sapiens
5172	solute carrier family 26, member 4	SLC26A4	Homo sapiens
11001	solute carrier family 27 (fatty acid transporter), member 2	SLC27A2	Homo sapiens
11000	solute carrier family 27 (fatty acid transporter), member 3	SLC27A3	Homo sapiens

9154	solute carrier family 28 (sodium-coupled nucleoside transporter), member 1	SLC28A1	Homo sapiens
3177	solute carrier family 29 (nucleoside transporters), member 2	SLC29A2	Homo sapiens
6513	solute carrier family 2 (facilitated glucose transporter), member 1	SLC2A1	Homo sapiens
6514	solute carrier family 2 (facilitated glucose transporter), member 2	SLC2A2	Homo sapiens
6515	solute carrier family 2 (facilitated glucose transporter), member 3	SLC2A3	Homo sapiens
6517	solute carrier family 2 (facilitated glucose transporter), member 4	SLC2A4	Homo sapiens
6518	solute carrier family 2 (facilitated glucose/fructose transporter), member 5	SLC2A5	Homo sapiens
7781	solute carrier family 30 (zinc transporter), member 3	SLC30A3	Homo sapiens
7782	solute carrier family 30 (zinc transporter), member 4	SLC30A4	Homo sapiens
10463	solute carrier family 30 (zinc transporter), member 9	SLC30A9	Homo sapiens
1317	solute carrier family 31 (copper transporters), member 1	SLC31A1	Homo sapiens
1318	solute carrier family 31 (copper transporters), member 2	SLC31A2	Homo sapiens
9197	solute carrier family 33 (acetyl-CoA transporter), member 1	SLC33A1	Homo sapiens
10559	solute carrier family 35 (CMP-sialic acid transporter), member A1	SLC35A1	Homo sapiens
23443	solute carrier family 35 (UDP-N-acetylglucosamine (UDP-GlcNAc) transporter), member A3	SLC35A3	Homo sapiens
10237	solute carrier family 35, member B1	SLC35B1	Homo sapiens
11046	solute carrier family 35, member D2	SLC35D2	Homo sapiens
10991	solute carrier family 38, member 3	SLC38A3	Homo sapiens
25800	solute carrier family 39 (zinc transporter), member 6	SLC39A6	Homo sapiens
7922	solute carrier family 39 (zinc transporter), member 7	SLC39A7	Homo sapiens
64116	solute carrier family 39 (zinc transporter), member 8	SLC39A8	Homo sapiens
8501	solute carrier family 43, member 1	SLC43A1	Homo sapiens
6508	solute carrier family 4, anion exchanger, member 3	SLC4A3	Homo sapiens
8671	solute carrier family 4, sodium bicarbonate cotransporter, member 4	SLC4A4	Homo sapiens
9497	solute carrier family 4, sodium bicarbonate cotransporter, member 7	SLC4A7	Homo sapiens
9498	solute carrier family 4, sodium bicarbonate cotransporter, member 8	SLC4A8	Homo sapiens
6523	solute carrier family 5 (sodium/glucose cotransporter), member 1	SLC5A1	Homo sapiens
6539	solute carrier family 6 (neurotransmitter transporter, betaine/GABA), member 12	SLC6A12	Homo sapiens
6530	solute carrier family 6 (neurotransmitter transporter, noradrenalin), member 2	SLC6A2	Homo sapiens
6531	solute carrier family 6 (neurotransmitter transporter, dopamine), member 3	SLC6A3	Homo sapiens
6532	solute carrier family 6 (neurotransmitter transporter, serotonin), member 4	SLC6A4	Homo sapiens
9152	solute carrier family 6 (neurotransmitter transporter, glycine), member 5	SLC6A5	Homo sapiens
6533	solute carrier family 6 (neurotransmitter transporter, taurine), member 6	SLC6A6	Homo sapiens
6535	solute carrier family 6 (neurotransmitter transporter, creatine), member 8	SLC6A8	Homo sapiens
6541	solute carrier family 7 (cationic amino acid transporter, y+ system), member 1	SLC7A1	Homo sapiens
6542	solute carrier family 7 (cationic amino acid transporter, y+ system), member 2	SLC7A2	Homo sapiens
8140	solute carrier family 7 (cationic amino acid transporter, y+ system),	SLC7A5	Homo sapiens

	member 5		
23428	solute carrier family 7 (cationic amino acid transporter, y+ system), member 8	SLC7A8	Homo sapiens
6548	solute carrier family 9 (sodium/hydrogen exchanger), member 1	SLC9A1	Homo sapiens
9351	solute carrier family 9 (sodium/hydrogen exchanger), member 3 regulator 2	SLC9A3R2	Homo sapiens
10479	solute carrier family 9 (sodium/hydrogen exchanger), member 6	SLC9A6	Homo sapiens
23315	solute carrier family 9 (sodium/hydrogen exchanger), member 8	SLC9A8	Homo sapiens
6579	solute carrier organic anion transporter family, member 1A2	SLCO1A2	Homo sapiens
6578	solute carrier organic anion transporter family, member 2A1	SLCO2A1	Homo sapiens
11309	solute carrier organic anion transporter family, member 2B1	SLCO2B1	Homo sapiens
28232	solute carrier organic anion transporter family, member 3A1	SLCO3A1	Homo sapiens
6585	slit homolog 1 (Drosophila)	SLIT1	Homo sapiens
9353	slit homolog 2 (Drosophila)	SLIT2	Homo sapiens
6586	slit homolog 3 (Drosophila)	SLIT3	Homo sapiens
6590	secretory leukocyte peptidase inhibitor	SLPI	Homo sapiens
4086	SMAD family member 1	SMAD1	Homo sapiens
4087	SMAD family member 2	SMAD2	Homo sapiens
4088	SMAD family member 3	SMAD3	Homo sapiens
4090	SMAD family member 5	SMAD5	Homo sapiens
4091	SMAD family member 6	SMAD6	Homo sapiens
4092	SMAD family member 7	SMAD7	Homo sapiens
6594	SWI/SNF related, matrix associated, actin dependent regulator of chromatin, subfamily a, member 1	SMARCA1	Homo sapiens
6595	SWI/SNF related, matrix associated, actin dependent regulator of chromatin, subfamily a, member 2	SMARCA2	Homo sapiens
6597	SWI/SNF related, matrix associated, actin dependent regulator of chromatin, subfamily a, member 4	SMARCA4	Homo sapiens
8467	SWI/SNF related, matrix associated, actin dependent regulator of chromatin, subfamily a, member 5	SMARCA5	Homo sapiens
6599	SWI/SNF related, matrix associated, actin dependent regulator of chromatin, subfamily c, member 1	SMARCC1	Homo sapiens
6601	SWI/SNF related, matrix associated, actin dependent regulator of chromatin, subfamily c, member 2	SMARCC2	Homo sapiens
6602	SWI/SNF related, matrix associated, actin dependent regulator of chromatin, subfamily d, member 1	SMARCD1	Homo sapiens
6603	SWI/SNF related, matrix associated, actin dependent regulator of chromatin, subfamily d, member 2	SMARCD2	Homo sapiens
6604	SWI/SNF related, matrix associated, actin dependent regulator of chromatin, subfamily d, member 3	SMARCD3	Homo sapiens
6605	SWI/SNF related, matrix associated, actin dependent regulator of chromatin, subfamily e, member 1	SMARCE1	Homo sapiens
10285	survival motor neuron domain containing 1	SMNDC1	Homo sapiens
6608	smoothened homolog (Drosophila)	SMO	Homo sapiens
64093	SPARC related modular calcium binding 1	SMOC1	Homo sapiens
54498	spermine oxidase	SMOX	Homo sapiens
6609	sphingomyelin phosphodiesterase 1, acid lysosomal	SMPD1	Homo sapiens
55512	sphingomyelin phosphodiesterase 3, neutral membrane (neutral sphingomyelinase II)	SMPD3	Homo sapiens

10924	sphingomyelin phosphodiesterase, acid-like 3A	SMPDL3A	Homo sapiens
27293	sphingomyelin phosphodiesterase, acid-like 3B	SMPDL3B	Homo sapiens
6525	smoothelin	SMTN	Homo sapiens
57154	SMAD specific E3 ubiquitin protein ligase 1	SMURF1	Homo sapiens
64750	SMAD specific E3 ubiquitin protein ligase 2	SMURF2	Homo sapiens
56950	SET and MYND domain containing 2	SMYD2	Homo sapiens
10322	SMYD family member 5	SMYD5	Homo sapiens
6591	snail homolog 2 (Drosophila)	SNAI2	Homo sapiens
8773	synaptosomal-associated protein, 23kDa	SNAP23	Homo sapiens
6616	synaptosomal-associated protein, 25kDa	SNAP25	Homo sapiens
6618	small nuclear RNA activating complex, polypeptide 2, 45kDa	SNAPC2	Homo sapiens
6621	small nuclear RNA activating complex, polypeptide 4, 190kDa	SNAPC4	Homo sapiens
6622	synuclein, alpha (non A4 component of amyloid precursor)	SNCA	Homo sapiens
6620	synuclein, beta	SNCB	Homo sapiens
6623	synuclein, gamma (breast cancer-specific protein 1)	SNCG	Homo sapiens
8303	stannin	SNN	Homo sapiens
6626	small nuclear ribonucleoprotein polypeptide A	SNRPA	Homo sapiens
6628	small nuclear ribonucleoprotein polypeptides B and B1	SNRPB	Homo sapiens
6629	small nuclear ribonucleoprotein polypeptide B''	SNRPB2	Homo sapiens
6631	small nuclear ribonucleoprotein polypeptide C	SNRPC	Homo sapiens
6632	small nuclear ribonucleoprotein D1 polypeptide 16kDa; hypothetical protein LOC100129492	SNRPD1	Homo sapiens
100129492	small nuclear ribonucleoprotein D1 polypeptide 16kDa; hypothetical protein LOC100129492	SNRPD1	Homo sapiens
119358	small nuclear ribonucleoprotein D2 polypeptide 16.5kDa; similar to hCG2040270	SNRPD2	Homo sapiens
6633	small nuclear ribonucleoprotein D2 polypeptide 16.5kDa; similar to hCG2040270	SNRPD2	Homo sapiens
6634	small nuclear ribonucleoprotein D3 polypeptide 18kDa	SNRPD3	Homo sapiens
6635	small nuclear ribonucleoprotein polypeptide E-like 1; small nuclear ribonucleoprotein polypeptide E; similar to hCG23490	SNRPE	Homo sapiens
414153	small nuclear ribonucleoprotein polypeptide E-like 1; small nuclear ribonucleoprotein polypeptide E; similar to hCG23490	SNRPE	Homo sapiens
100130109	small nuclear ribonucleoprotein polypeptide E-like 1; small nuclear ribonucleoprotein polypeptide E; similar to hCG23490	SNRPE	Homo sapiens
6638	small nuclear ribonucleoprotein polypeptide N; SNRPN upstream reading frame	SNRPN	Homo sapiens
8926	small nuclear ribonucleoprotein polypeptide N; SNRPN upstream reading frame	SNRPN	Homo sapiens
6640	syntrophin, alpha 1 (dystrophin-associated protein A1, 59kDa, acidic component)	SNTA1	Homo sapiens
6641	syntrophin, beta 1 (dystrophin-associated protein A1, 59kDa, basic component 1)	SNTB1	Homo sapiens
6645	syntrophin, beta 2 (dystrophin-associated protein A1, 59kDa, basic component 2)	SNTB2	Homo sapiens
6642	sorting nexin 1	SNX1	Homo sapiens
23161	sorting nexin 13	SNX13	Homo sapiens
9784	sorting nexin 17	SNX17	Homo sapiens
8724	sorting nexin 3	SNX3	Homo sapiens

8723	sorting nexin 4	SNX4	Homo sapiens
6646	sterol O-acyltransferase 1	SOAT1	Homo sapiens
8651	suppressor of cytokine signaling 1	SOCS1	Homo sapiens
8835	suppressor of cytokine signaling 2	SOCS2	Homo sapiens
9021	suppressor of cytokine signaling 3	SOCS3	Homo sapiens
9306	suppressor of cytokine signaling 6	SOCS6	Homo sapiens
30837	suppressor of cytokine signaling 7	SOCS7	Homo sapiens
6647	superoxide dismutase 1, soluble	SOD1	Homo sapiens
6648	superoxide dismutase 2, mitochondrial	SOD2	Homo sapiens
10580	sorbin and SH3 domain containing 1	SORBS1	Homo sapiens
6272	sortilin 1	SORT1	Homo sapiens
6654	son of sevenless homolog 1 (Drosophila)	SOS1	Homo sapiens
6656	SRY (sex determining region Y)-box 1	SOX1	Homo sapiens
6663	SRY (sex determining region Y)-box 10	SOX10	Homo sapiens
6664	SRY (sex determining region Y)-box 11	SOX11	Homo sapiens
64321	SRY (sex determining region Y)-box 17	SOX17	Homo sapiens
54345	SRY (sex determining region Y)-box 18	SOX18	Homo sapiens
6657	SRY (sex determining region Y)-box 2	SOX2	Homo sapiens
6658	SRY (sex determining region Y)-box 3	SOX3	Homo sapiens
11063	SRY (sex determining region Y)-box 30	SOX30	Homo sapiens
6659	SRY (sex determining region Y)-box 4	SOX4	Homo sapiens
30812	SRY (sex determining region Y)-box 8	SOX8	Homo sapiens
6662	SRY (sex determining region Y)-box 9	SOX9	Homo sapiens
6667	Sp1 transcription factor	SP1	Homo sapiens
6672	SP100 nuclear antigen	SP100	Homo sapiens
3431	SP110 nuclear body protein	SP110	Homo sapiens
11262	SP140 nuclear body protein	SP140	Homo sapiens
6668	Sp2 transcription factor	SP2	Homo sapiens
6671	Sp4 transcription factor	SP4	Homo sapiens
53340	sperm autoantigenic protein 17	SPA17	Homo sapiens
6674	sperm associated antigen 1	SPAG1	Homo sapiens
10615	sperm associated antigen 5	SPAG5	Homo sapiens
9576	sperm associated antigen 6	SPAG6	Homo sapiens
9552	sperm associated antigen 7	SPAG7	Homo sapiens
26206	sperm associated antigen 8	SPAG8	Homo sapiens
6677	sperm adhesion molecule 1 (PH-20 hyaluronidase, zona pellucida binding)	SPAM1	Homo sapiens
6678	secreted protein, acidic, cysteine-rich (osteonectin)	SPARC	Homo sapiens
8404	SPARC-like 1 (hevin)	SPARCL1	Homo sapiens
9825	spermatogenesis associated 2	SPATA2	Homo sapiens
25803	SAM pointed domain containing ets transcription factor	SPDEF	Homo sapiens
23111	spastic paraplegia 20 (Troyer syndrome)	SPG20	Homo sapiens
6687	spastic paraplegia 7 (pure and complicated autosomal recessive)	SPG7	Homo sapiens
8877	sphingosine kinase 1	SPHK1	Homo sapiens
6688	spleen focus forming virus (SFFV) proviral integration oncogene spi1	SPI1	Homo sapiens
6689	Spi-B transcription factor (Spi-1/PU.1 related)	SPIB	Homo sapiens

6691	serine peptidase inhibitor, Kazal type 2 (acrosin-trypsin inhibitor)	SPINK2	Homo sapiens
6692	serine peptidase inhibitor, Kunitz type 1	SPINT1	Homo sapiens
10653	serine peptidase inhibitor, Kunitz type, 2	SPINT2	Homo sapiens
6693	sialophorin	SPN	Homo sapiens
9806	sparc/osteonectin, cwcv and kazal-like domains proteoglycan (testican) 2	SPOCK2	Homo sapiens
10418	spondin 1, extracellular matrix protein	SPON1	Homo sapiens
8405	speckle-type POZ protein	SPOP	Homo sapiens
6696	secreted phosphoprotein 1	SPP1	Homo sapiens
200734	sprouty-related, EVH1 domain containing 2	SPRED2	Homo sapiens
6699	small proline-rich protein 1B (cornifin)	SPRR1B	Homo sapiens
10252	sprouty homolog 1, antagonist of FGF signaling (Drosophila)	SPRY1	Homo sapiens
10253	sprouty homolog 2 (Drosophila)	SPRY2	Homo sapiens
6709	spectrin, alpha, non-erythrocytic 1 (alpha-fodrin)	SPTAN1	Homo sapiens
6711	spectrin, beta, non-erythrocytic 1	SPTBN1	Homo sapiens
10558	serine palmitoyltransferase, long chain base subunit 1	SPTLC1	Homo sapiens
9517	serine palmitoyltransferase, long chain base subunit 2	SPTLC2	Homo sapiens
6713	squalene epoxidase	SQLE	Homo sapiens
10011	steroid receptor RNA activator 1	SRA1	Homo sapiens
6714	v-src sarcoma (Schmidt-Ruppin A-2) viral oncogene homolog (avian)	SRC	Homo sapiens
6715	steroid-5-alpha-reductase, alpha polypeptide 1 (3-oxo-5 alpha-steroid delta 4-dehydrogenase alpha 1)	SRD5A1	Homo sapiens
6716	steroid-5-alpha-reductase, alpha polypeptide 2 (3-oxo-5 alpha-steroid delta 4-dehydrogenase alpha 2)	SRD5A2	Homo sapiens
6720	sterol regulatory element binding transcription factor 1	SREBF1	Homo sapiens
6721	sterol regulatory element binding transcription factor 2	SREBF2	Homo sapiens
6722	serum response factor (c-fos serum response element-binding transcription factor)	SRF	Homo sapiens
6717	sorcin	SRI	Homo sapiens
6723	spermidine synthase	SRM	Homo sapiens
6729	similar to signal recognition particle 54kDa; signal recognition particle 54kDa	SRP54	Homo sapiens
650638	similar to signal recognition particle 54kDa; signal recognition particle 54kDa	SRP54	Homo sapiens
6731	signal recognition particle 72kDa	SRP72	Homo sapiens
653226	signal recognition particle 9-like 1; signal recognition particle 9kDa	SRP9	Homo sapiens
6726	signal recognition particle 9-like 1; signal recognition particle 9kDa	SRP9	Homo sapiens
6732	SFRS protein kinase 1	SRPK1	Homo sapiens
6733	SFRS protein kinase 2	SRPK2	Homo sapiens
8406	sushi-repeat-containing protein, X-linked	SRPX	Homo sapiens
27286	sushi-repeat-containing protein, X-linked 2	SRPX2	Homo sapiens
10250	serine/arginine repetitive matrix 1	SRRM1	Homo sapiens
23524	serine/arginine repetitive matrix 2; hypothetical LOC100132779	SRRM2	Homo sapiens
100132779	serine/arginine repetitive matrix 2; hypothetical LOC100132779	SRRM2	Homo sapiens
6760	synovial sarcoma translocation, chromosome 18	SS18	Homo sapiens
26039	synovial sarcoma translocation gene on chromosome 18-like 1	SS18L1	Homo sapiens
6741	Sjogren syndrome antigen B (autoantigen La)	SSB	Homo sapiens

6742	single-stranded DNA binding protein 1	SSBP1	Homo sapiens
23635	single-stranded DNA binding protein 2	SSBP2	Homo sapiens
6744	sperm specific antigen 2	SSFA2	Homo sapiens
8636	Sjogren syndrome nuclear autoantigen 1	SSNA1	Homo sapiens
8082	sarcospan (Kras oncogene-associated gene)	SSPN	Homo sapiens
6745	signal sequence receptor, alpha	SSR1	Homo sapiens
6746	signal sequence receptor, beta (translocon-associated protein beta)	SSR2	Homo sapiens
6749	structure specific recognition protein 1	SSRP1	Homo sapiens
6750	somatostatin	SST	Homo sapiens
6751	somatostatin receptor 1	SSTR1	Homo sapiens
6753	somatostatin receptor 3	SSTR3	Homo sapiens
10214	synovial sarcoma, X breakpoint 3	SSX3	Homo sapiens
6768	suppression of tumorigenicity 14 (colon carcinoma)	ST14	Homo sapiens
9705	suppression of tumorigenicity 18 (breast carcinoma) (zinc finger protein)	ST18	Homo sapiens
10735	stromal antigen 2	STAG2	Homo sapiens
8027	signal transducing adaptor molecule (SH3 domain and ITAM motif) 1	STAM	Homo sapiens
10254	signal transducing adaptor molecule (SH3 domain and ITAM motif) 2	STAM2	Homo sapiens
10617	STAM binding protein	STAMBP	Homo sapiens
6770	steroidogenic acute regulatory protein	STAR	Homo sapiens
90627	StAR-related lipid transfer (START) domain containing 13	STARD13	Homo sapiens
10948	StAR-related lipid transfer (START) domain containing 3	STARD3	Homo sapiens
56910	StAR-related lipid transfer (START) domain containing 7	STARD7	Homo sapiens
6772	signal transducer and activator of transcription 1, 91kDa	STAT1	Homo sapiens
6773	signal transducer and activator of transcription 2, 113kDa	STAT2	Homo sapiens
6774	signal transducer and activator of transcription 3 (acute-phase response factor)	STAT3	Homo sapiens
6775	signal transducer and activator of transcription 4	STAT4	Homo sapiens
6776	signal transducer and activator of transcription 5A	STAT5A	Homo sapiens
6777	signal transducer and activator of transcription 5B	STAT5B	Homo sapiens
27067	staufen, RNA binding protein, homolog 2 (Drosophila)	STAU2	Homo sapiens
6781	stanniocalcin 1	STC1	Homo sapiens
10963	stress-induced-phosphoprotein 1	STIP1	Homo sapiens
6793	serine/threonine kinase 10	STK10	Homo sapiens
6794	serine/threonine kinase 11	STK11	Homo sapiens
9263	serine/threonine kinase 17a	STK17A	Homo sapiens
9262	serine/threonine kinase 17b	STK17B	Homo sapiens
8428	serine/threonine kinase 24 (STE20 homolog, yeast)	STK24	Homo sapiens
10494	serine/threonine kinase 25 (STE20 homolog, yeast)	STK25	Homo sapiens
6788	serine/threonine kinase 3 (STE20 homolog, yeast)	STK3	Homo sapiens
11329	serine/threonine kinase 38	STK38	Homo sapiens
23012	serine/threonine kinase 38 like	STK38L	Homo sapiens
27347	serine threonine kinase 39 (STE20/SPS1 homolog, yeast)	STK39	Homo sapiens
6789	serine/threonine kinase 4	STK4	Homo sapiens
11075	stathmin-like 2	STMN2	Homo sapiens
50861	stathmin-like 3	STMN3	Homo sapiens

9399	stomatin (EPB72)-like 1	STOML1	Homo sapiens
201254	stimulated by retinoic acid 13 homolog (mouse)	STRA13	Homo sapiens
11171	serine/threonine kinase receptor associated protein	STRAP	Homo sapiens
29966	striatin, calmodulin binding protein 3	STRN3	Homo sapiens
412	steroid sulfatase (microsomal), isozyme S	STS	Homo sapiens
8676	syntaxin 11	STX11	Homo sapiens
8675	syntaxin 16	STX16	Homo sapiens
6804	syntaxin 1A (brain)	STX1A	Homo sapiens
10228	syntaxin 6	STX6	Homo sapiens
8417	syntaxin 7	STX7	Homo sapiens
6812	syntaxin binding protein 1	STXBP1	Homo sapiens
6814	syntaxin binding protein 3	STXBP3	Homo sapiens
8803	succinate-CoA ligase, ADP-forming, beta subunit	SUCLA2	Homo sapiens
51684	suppressor of fused homolog (Drosophila)	SUFU	Homo sapiens
23213	sulfatase 1	SULF1	Homo sapiens
6819	sulfotransferase family, cytosolic, 1C, member 2	SULT1C2	Homo sapiens
6820	sulfotransferase family, cytosolic, 2B, member 1	SULT2B1	Homo sapiens
25830	sulfotransferase family 4A, member 1	SULT4A1	Homo sapiens
474338	SMT3 suppressor of mif two 3 homolog 1 (S. cerevisiae); SUMO1 pseudogene 3	SUMO1	Homo sapiens
7341	SMT3 suppressor of mif two 3 homolog 1 (S. cerevisiae); SUMO1 pseudogene 3	SUMO1	Homo sapiens
6821	sulfite oxidase	SUOX	Homo sapiens
11198	suppressor of Ty 16 homolog (S. cerevisiae); suppressor of Ty 16 homolog (S. cerevisiae) pseudogene	SUPT16H	Homo sapiens
400011	suppressor of Ty 16 homolog (S. cerevisiae); suppressor of Ty 16 homolog (S. cerevisiae) pseudogene	SUPT16H	Homo sapiens
6827	suppressor of Ty 4 homolog 1 (S. cerevisiae)	SUPT4H1	Homo sapiens
6829	suppressor of Ty 5 homolog (S. cerevisiae)	SUPT5H	Homo sapiens
6830	suppressor of Ty 6 homolog (S. cerevisiae)	SUPT6H	Homo sapiens
6832	suppressor of var1, 3-like 1 (S. cerevisiae)	SUPV3L1	Homo sapiens
6834	surfeit 1	SURF1	Homo sapiens
6835	surfeit 2	SURF2	Homo sapiens
6840	supervillin	SVIL	Homo sapiens
6847	synaptonemal complex protein 1	SYCP1	Homo sapiens
10388	synaptonemal complex protein 2	SYCP2	Homo sapiens
6850	spleen tyrosine kinase	SYK	Homo sapiens
6853	synapsin I	SYN1	Homo sapiens
6854	synapsin II	SYN2	Homo sapiens
10492	synaptotagmin binding, cytoplasmic RNA interacting protein	SYNCRIP	Homo sapiens
9145	synaptogyrin 1	SYNGR1	Homo sapiens
9143	synaptogyrin 3	SYNGR3	Homo sapiens
8867	synaptojanin 1	SYNJ1	Homo sapiens
8871	synaptojanin 2	SYNJ2	Homo sapiens
6855	synaptophysin	SYP	Homo sapiens
6857	synaptotagmin I	SYT1	Homo sapiens
6861	synaptotagmin V	SYT5	Homo sapiens

6862	T, brachyury homolog (mouse)	T	Homo sapiens
6863	tachykinin, precursor 1	TAC1	Homo sapiens
6867	transforming, acidic coiled-coil containing protein 1	TACC1	Homo sapiens
6865	tachykinin receptor 2	TACR2	Homo sapiens
6872	TAF1 RNA polymerase II, TATA box binding protein (TBP)-associated factor, 250kDa	TAF1	Homo sapiens
6882	TAF11 RNA polymerase II, TATA box binding protein (TBP)-associated factor, 28kDa	TAF11	Homo sapiens
6883	TAF12 RNA polymerase II, TATA box binding protein (TBP)-associated factor, 20kDa	TAF12	Homo sapiens
8148	TAF15 RNA polymerase II, TATA box binding protein (TBP)-associated factor, 68kDa	TAF15	Homo sapiens
9015	TATA box binding protein (TBP)-associated factor, RNA polymerase I, A, 48kDa	TAF1A	Homo sapiens
9014	TATA box binding protein (TBP)-associated factor, RNA polymerase I, B, 63kDa	TAF1B	Homo sapiens
9013	TATA box binding protein (TBP)-associated factor, RNA polymerase I, C, 110kDa	TAF1C	Homo sapiens
6873	TAF2 RNA polymerase II, TATA box binding protein (TBP)-associated factor, 150kDa	TAF2	Homo sapiens
6877	TAF5 RNA polymerase II, TATA box binding protein (TBP)-associated factor, 100kDa	TAF5	Homo sapiens
27097	TAF5-like RNA polymerase II, p300/CBP-associated factor (PCAF)-associated factor, 65kDa	TAF5L	Homo sapiens
6878	TAF6 RNA polymerase II, TATA box binding protein (TBP)-associated factor, 80kDa	TAF6	Homo sapiens
10629	TAF6-like RNA polymerase II, p300/CBP-associated factor (PCAF)-associated factor, 65kDa	TAF6L	Homo sapiens
6880	TAF9 RNA polymerase II, TATA box binding protein (TBP)-associated factor, 32kDa	TAF9	Homo sapiens
6888	transaldolase 1	TALDO1	Homo sapiens
10010	TRAF family member-associated NFKB activator	TANK	Homo sapiens
6891	transporter 2, ATP-binding cassette, sub-family B (MDR/TAP)	TAP2	Homo sapiens
6892	TAP binding protein (tapasin)	TAPBP	Homo sapiens
6894	TAR (HIV-1) RNA binding protein 1	TARBP1	Homo sapiens
6895	TAR (HIV-1) RNA binding protein 2	TARBP2	Homo sapiens
23435	TAR DNA binding protein	TARDBP	Homo sapiens
6897	threonyl-tRNA synthetase	TARS	Homo sapiens
8887	Tax1 (human T-cell leukemia virus type I) binding protein 1	TAX1BP1	Homo sapiens
30851	Tax1 (human T-cell leukemia virus type I) binding protein 3	TAX1BP3	Homo sapiens
6901	tafazzin	TAZ	Homo sapiens
23216	TBC1 (tre-2/USP6, BUB2, cdc16) domain family, member 1	TBC1D1	Homo sapiens
64786	TBC1 domain family, member 15	TBC1D15	Homo sapiens
79735	TBC1 domain family, member 17	TBC1D17	Homo sapiens
55357	TBC1 domain family, member 2	TBC1D2	Homo sapiens
9779	TBC1 domain family, member 5	TBC1D5	Homo sapiens
51256	TBC1 domain family, member 7	TBC1D7	Homo sapiens
6902	tubulin folding cofactor A	TBCA	Homo sapiens
6903	tubulin folding cofactor C	TBCC	Homo sapiens

6905	tubulin folding cofactor E	TBCE	Homo sapiens
6907	transducin (beta)-like 1X-linked	TBL1X	Homo sapiens
26608	transducin (beta)-like 2	TBL2	Homo sapiens
6908	TATA box binding protein	TBP	Homo sapiens
9519	TBP-like 1	TBPL1	Homo sapiens
9095	T-box 19	TBX19	Homo sapiens
6909	T-box 2	TBX2	Homo sapiens
30009	T-box 21	TBX21	Homo sapiens
6926	T-box 3	TBX3	Homo sapiens
6915	thromboxane A2 receptor	TBXA2R	Homo sapiens
6916	thromboxane A synthase 1 (platelet)	TBXAS1	Homo sapiens
6919	transcription elongation factor A (SII), 2	TCEA2	Homo sapiens
100132973	similar to elongin C; transcription elongation factor B (SIII), polypeptide 1 (15kDa, elongin C)	TCEB1	Homo sapiens
6921	similar to elongin C; transcription elongation factor B (SIII), polypeptide 1 (15kDa, elongin C)	TCEB1	Homo sapiens
6923	transcription elongation factor B (SIII), polypeptide 2 (18kDa, elongin B)	TCEB2	Homo sapiens
6924	transcription elongation factor B (SIII), polypeptide 3 (110kDa, elongin A)	TCEB3	Homo sapiens
10915	transcription elongation regulator 1	TCERG1	Homo sapiens
6938	transcription factor 12	TCF12	Homo sapiens
6942	transcription factor 20 (AR1)	TCF20	Homo sapiens
6925	transcription factor 4	TCF4	Homo sapiens
6932	transcription factor 7 (T-cell specific, HMG-box)	TCF7	Homo sapiens
83439	transcription factor 7-like 1 (T-cell specific, HMG-box)	TCF7L1	Homo sapiens
6934	transcription factor 7-like 2 (T-cell specific, HMG-box)	TCF7L2	Homo sapiens
10312	T-cell, immune regulator 1, ATPase, H+ transporting, lysosomal V0 subunit A3	TCIRG1	Homo sapiens
6947	transcobalamin I (vitamin B12 binding protein, R binder family)	TCN1	Homo sapiens
6948	transcobalamin II; macrocytic anemia	TCN2	Homo sapiens
6949	Treacher Collins-Franceschetti syndrome 1	TCOF1	Homo sapiens
6999	tryptophan 2,3-dioxygenase	TDO2	Homo sapiens
23424	tudor domain containing 7	TDRD7	Homo sapiens
7003	TEA domain family member 1 (SV40 transcriptional enhancer factor)	TEAD1	Homo sapiens
8463	TEA domain family member 2	TEAD2	Homo sapiens
7004	TEA domain family member 4	TEAD4	Homo sapiens
7007	tectorin alpha	TECTA	Homo sapiens
7010	TEK tyrosine kinase, endothelial	TEK	Homo sapiens
27285	tektin 2 (testicular)	TEKT2	Homo sapiens
23371	tensin like C1 domain containing phosphatase (tensin 2)	TENC1	Homo sapiens
7014	telomeric repeat binding factor 2	TERF2	Homo sapiens
54386	telomeric repeat binding factor 2, interacting protein	TERF2IP	Homo sapiens
7015	telomerase reverse transcriptase	TERT	Homo sapiens
7016	testis-specific kinase 1	TESK1	Homo sapiens
10420	testis-specific kinase 2	TESK2	Homo sapiens
56159	testis expressed 11	TEX11	Homo sapiens

113419	testis expressed 261	TEX261	Homo sapiens
7018	transferrin	TF	Homo sapiens
7019	transcription factor A, mitochondrial	TFAM	Homo sapiens
7020	transcription factor AP-2 alpha (activating enhancer binding protein 2 alpha)	TFAP2A	Homo sapiens
7021	transcription factor AP-2 beta (activating enhancer binding protein 2 beta)	TFAP2B	Homo sapiens
7023	transcription factor AP-4 (activating enhancer binding protein 4)	TFAP4	Homo sapiens
64216	transcription factor B2, mitochondrial	TFB2M	Homo sapiens
7024	transcription factor CP2	TFCP2	Homo sapiens
29842	transcription factor CP2-like 1	TFCP2L1	Homo sapiens
7027	transcription factor Dp-1	TFDP1	Homo sapiens
7030	transcription factor binding to IGHM enhancer 3	TFE3	Homo sapiens
24144	tuftelin interacting protein 11	TFIP11	Homo sapiens
7035	tissue factor pathway inhibitor (lipoprotein-associated coagulation inhibitor)	TFPI	Homo sapiens
7980	tissue factor pathway inhibitor 2	TFPI2	Homo sapiens
7038	thyroglobulin	TG	Homo sapiens
23483	TDP-glucose 4,6-dehydratase	TGDS	Homo sapiens
7039	transforming growth factor, alpha	TGFA	Homo sapiens
7041	transforming growth factor beta 1 induced transcript 1	TGFB1I1	Homo sapiens
7042	transforming growth factor, beta 2	TGFB2	Homo sapiens
7043	transforming growth factor, beta 3	TGFB3	Homo sapiens
7045	transforming growth factor, beta-induced, 68kDa	TGFBI	Homo sapiens
7046	transforming growth factor, beta receptor 1	TGFBR1	Homo sapiens
7048	transforming growth factor, beta receptor II (70/80kDa)	TGFBR2	Homo sapiens
7049	transforming growth factor, beta receptor III	TGFBR3	Homo sapiens
9392	transforming growth factor, beta receptor associated protein 1	TGFBRAP1	Homo sapiens
60436	TGFB-induced factor homeobox 2	TGIF2	Homo sapiens
7051	transglutaminase 1 (K polypeptide epidermal type I, protein-glutamine-gamma-glutamyltransferase)	TGM1	Homo sapiens
7052	transglutaminase 2 (C polypeptide, protein-glutamine-gamma-glutamyltransferase)	TGM2	Homo sapiens
7053	transglutaminase 3 (E polypeptide, protein-glutamine-gamma-glutamyltransferase)	TGM3	Homo sapiens
7047	transglutaminase 4 (prostate)	TGM4	Homo sapiens
10618	trans-golgi network protein 2	TGOLN2	Homo sapiens
7054	tyrosine hydroxylase	TH	Homo sapiens
57215	THAP domain containing 11	THAP11	Homo sapiens
7057	thrombospondin 1	THBS1	Homo sapiens
7059	thrombospondin 3	THBS3	Homo sapiens
7060	thrombospondin 4	THBS4	Homo sapiens
9984	THO complex 1	THOC1	Homo sapiens
57187	THO complex 2	THOC2	Homo sapiens
7064	thimet oligopeptidase 1	THOP1	Homo sapiens
7066	thrombopoietin	THPO	Homo sapiens
7067	thyroid hormone receptor, alpha (erythroblastic leukemia viral (v-erb-a) oncogene homolog, avian)	THRA	Homo sapiens

7068	thyroid hormone receptor, beta (erythroblastic leukemia viral (v-erb-a) oncogene homolog 2, avian)	THRB	Homo sapiens
7069	thyroid hormone responsive (SPOT14 homolog, rat)	THRSP	Homo sapiens
7070	Thy-1 cell surface antigen	THY1	Homo sapiens
7072	TIA1 cytotoxic granule-associated RNA binding protein	TIA1	Homo sapiens
7073	TIA1 cytotoxic granule-associated RNA binding protein-like 1	TIAL1	Homo sapiens
7074	T-cell lymphoma invasion and metastasis 1	TIAM1	Homo sapiens
8914	timeless homolog (Drosophila)	TIMELESS	Homo sapiens
10440	translocase of inner mitochondrial membrane 17 homolog A (yeast)	TIMM17A	Homo sapiens
10245	translocase of inner mitochondrial membrane 17 homolog B (yeast)	TIMM17B	Homo sapiens
10469	translocase of inner mitochondrial membrane 44 homolog (yeast)	TIMM44	Homo sapiens
7076	TIMP metallopeptidase inhibitor 1	TIMP1	Homo sapiens
7077	TIMP metallopeptidase inhibitor 2	TIMP2	Homo sapiens
7078	TIMP metallopeptidase inhibitor 3	TIMP3	Homo sapiens
7082	tight junction protein 1 (zona occludens 1)	TJP1	Homo sapiens
9414	tight junction protein 2 (zona occludens 2)	TJP2	Homo sapiens
7084	thymidine kinase 2, mitochondrial	TK2	Homo sapiens
7086	transketolase	TKT	Homo sapiens
8277	transketolase-like 1	TKTL1	Homo sapiens
7088	similar to transducin-like enhancer of split 1 (E(sp1) homolog, Drosophila); transducin-like enhancer of split 1 (E(sp1) homolog, Drosophila)	TLE1	Homo sapiens
645381	similar to transducin-like enhancer of split 1 (E(sp1) homolog, Drosophila); transducin-like enhancer of split 1 (E(sp1) homolog, Drosophila)	TLE1	Homo sapiens
7090	transducin-like enhancer of split 3 (E(sp1) homolog, Drosophila)	TLE3	Homo sapiens
7091	transducin-like enhancer of split 4 (E(sp1) homolog, Drosophila)	TLE4	Homo sapiens
9874	tousled-like kinase 1	TLK1	Homo sapiens
11011	tousled-like kinase 2	TLK2	Homo sapiens
7093	tolloid-like 2	TLL2	Homo sapiens
83660	talin 2	TLN2	Homo sapiens
7097	toll-like receptor 2	TLR2	Homo sapiens
7098	toll-like receptor 3	TLR3	Homo sapiens
7099	toll-like receptor 4	TLR4	Homo sapiens
51284	toll-like receptor 7	TLR7	Homo sapiens
51311	toll-like receptor 8	TLR8	Homo sapiens
4071	transmembrane 4 L six family member 1	TM4SF1	Homo sapiens
7104	transmembrane 4 L six family member 4	TM4SF4	Homo sapiens
9032	transmembrane 4 L six family member 5	TM4SF5	Homo sapiens
7108	transmembrane 7 superfamily member 2	TM7SF2	Homo sapiens
10548	transmembrane 9 superfamily member 1	TM9SF1	Homo sapiens
9375	transmembrane 9 superfamily member 2	TM9SF2	Homo sapiens
3371	tenascin C	TNC	Homo sapiens
7124	tumor necrosis factor (TNF superfamily, member 2)	TNF	Homo sapiens
7126	tumor necrosis factor, alpha-induced protein 1 (endothelial)	TNFAIP1	Homo sapiens
7127	tumor necrosis factor, alpha-induced protein 2	TNFAIP2	Homo sapiens
7128	tumor necrosis factor, alpha-induced protein 3	TNFAIP3	Homo sapiens

7130	tumor necrosis factor, alpha-induced protein 6	TNFAIP6	Homo sapiens
8797	tumor necrosis factor receptor superfamily, member 10a	TNFRSF10A	Homo sapiens
8795	tumor necrosis factor receptor superfamily, member 10b	TNFRSF10B	Homo sapiens
8793	tumor necrosis factor receptor superfamily, member 10d, decoy with truncated death domain	TNFRSF10D	Homo sapiens
4982	tumor necrosis factor receptor superfamily, member 11b	TNFRSF11B	Homo sapiens
51330	tumor necrosis factor receptor superfamily, member 12A	TNFRSF12A	Homo sapiens
8764	tumor necrosis factor receptor superfamily, member 14 (herpesvirus entry mediator)	TNFRSF14	Homo sapiens
608	tumor necrosis factor receptor superfamily, member 17	TNFRSF17	Homo sapiens
7133	tumor necrosis factor receptor superfamily, member 1B	TNFRSF1B	Homo sapiens
27242	tumor necrosis factor receptor superfamily, member 21	TNFRSF21	Homo sapiens
8718	tumor necrosis factor receptor superfamily, member 25	TNFRSF25	Homo sapiens
8771	tumor necrosis factor receptor superfamily, member 6b, decoy; regulator of telomere elongation helicase 1	TNFRSF6B	Homo sapiens
51750	tumor necrosis factor receptor superfamily, member 6b, decoy; regulator of telomere elongation helicase 1	TNFRSF6B	Homo sapiens
943	tumor necrosis factor receptor superfamily, member 8	TNFRSF8	Homo sapiens
8743	tumor necrosis factor (ligand) superfamily, member 10	TNFSF10	Homo sapiens
8600	tumor necrosis factor (ligand) superfamily, member 11	TNFSF11	Homo sapiens
8741	TNFSF12-TNFSF13 readthrough transcript; tumor necrosis factor (ligand) superfamily, member 12; tumor necrosis factor (ligand) superfamily, member 13	TNFSF12	Homo sapiens
407977	TNFSF12-TNFSF13 readthrough transcript; tumor necrosis factor (ligand) superfamily, member 12; tumor necrosis factor (ligand) superfamily, member 13	TNFSF12	Homo sapiens
8742	TNFSF12-TNFSF13 readthrough transcript; tumor necrosis factor (ligand) superfamily, member 12; tumor necrosis factor (ligand) superfamily, member 13	TNFSF12	Homo sapiens
10673	tumor necrosis factor (ligand) superfamily, member 13b	TNFSF13B	Homo sapiens
7292	tumor necrosis factor (ligand) superfamily, member 4	TNFSF4	Homo sapiens
10318	TNFAIP3 interacting protein 1	TNIP1	Homo sapiens
8658	tankyrase, TRF1-interacting ankyrin-related ADP-ribose polymerase	TNKS	Homo sapiens
7134	troponin C type 1 (slow)	TNNC1	Homo sapiens
7135	troponin I type 1 (skeletal, slow)	TNNI1	Homo sapiens
7136	troponin I type 2 (skeletal, fast)	TNNI2	Homo sapiens
51086	TNNI3 interacting kinase; fucose-1-phosphate guanylyltransferase	TNNI3K	Homo sapiens
8790	TNNI3 interacting kinase; fucose-1-phosphate guanylyltransferase	TNNI3K	Homo sapiens
7139	troponin T type 2 (cardiac)	TNNT2	Homo sapiens
7140	troponin T type 3 (skeletal, fast)	TNNT3	Homo sapiens
7141	transition protein 1 (during histone to protamine replacement)	TNP1	Homo sapiens
3842	transportin 1	TNPO1	Homo sapiens
30000	transportin 2	TNPO2	Homo sapiens
7146	tenascin XB; tenascin XA pseudogene	TNXB	Homo sapiens
7148	tenascin XB; tenascin XA pseudogene	TNXB	Homo sapiens
10140	transducer of ERBB2, 1	TOB1	Homo sapiens
10766	transducer of ERBB2, 2	TOB2	Homo sapiens
10040	target of myb1 (chicken)-like 1	TOM1L1	Homo sapiens

10452	translocase of outer mitochondrial membrane 40 homolog (yeast)	TOMM40	Homo sapiens
54543	translocase of outer mitochondrial membrane 7 homolog (yeast)	TOMM7	Homo sapiens
9868	translocase of outer mitochondrial membrane 70 homolog A (S. cerevisiae)	TOMM70A	Homo sapiens
7150	topoisomerase (DNA) I	TOP1	Homo sapiens
7153	topoisomerase (DNA) II alpha 170kDa	TOP2A	Homo sapiens
7156	topoisomerase (DNA) III alpha	TOP3A	Homo sapiens
8940	topoisomerase (DNA) III beta	TOP3B	Homo sapiens
11073	topoisomerase (DNA) II binding protein 1	TOPBP1	Homo sapiens
10210	topoisomerase I binding, arginine/serine-rich	TOPORS	Homo sapiens
27348	torsin family 1, member B (torsin B)	TOR1B	Homo sapiens
7157	tumor protein p53	TP53	Homo sapiens
7158	tumor protein p53 binding protein 1	TP53BP1	Homo sapiens
7159	tumor protein p53 binding protein, 2	TP53BP2	Homo sapiens
9537	tumor protein p53 inducible protein 11	TP53I11	Homo sapiens
9540	tumor protein p53 inducible protein 3	TP53I3	Homo sapiens
94241	tumor protein p53 inducible nuclear protein 1	TP53INP1	Homo sapiens
112858	TP53 regulating kinase	TP53RK	Homo sapiens
7162	trophoblast glycoprotein	TPBG	Homo sapiens
7165	tumor protein D52-like 2	TPD52L2	Homo sapiens
7167	TPI1 pseudogene; triosephosphate isomerase 1	TPI1	Homo sapiens
729708	TPI1 pseudogene; triosephosphate isomerase 1	TPI1	Homo sapiens
7168	tropomyosin 1 (alpha)	TPM1	Homo sapiens
7169	tropomyosin 2 (beta)	TPM2	Homo sapiens
7170	tropomyosin 3	TPM3	Homo sapiens
7174	tripeptidyl peptidase II	TPP2	Homo sapiens
7175	translocated promoter region (to activated MET oncogene)	TPR	Homo sapiens
8460	tyrosylprotein sulfotransferase 1	TPST1	Homo sapiens
8459	tyrosylprotein sulfotransferase 2	TPST2	Homo sapiens
22974	TPX2, microtubule-associated, homolog (Xenopus laevis)	TPX2	Homo sapiens
8717	TNFRSF1A-associated via death domain	TRADD	Homo sapiens
7185	TNF receptor-associated factor 1	TRAF1	Homo sapiens
9618	TNF receptor-associated factor 4	TRAF4	Homo sapiens
7188	TNF receptor-associated factor 5	TRAF5	Homo sapiens
7189	TNF receptor-associated factor 6	TRAF6	Homo sapiens
23471	translocation associated membrane protein 1	TRAM1	Homo sapiens
10131	TNF receptor-associated protein 1	TRAP1	Homo sapiens
27095	trafficking protein particle complex 3	TRAPPC3	Homo sapiens
11181	trehalase (brush-border membrane glycoprotein)	TREH	Homo sapiens
10221	tribbles homolog 1 (Drosophila)	TRIB1	Homo sapiens
57761	tribbles homolog 3 (Drosophila)	TRIB3	Homo sapiens
10107	tripartite motif-containing 10	TRIM10	Homo sapiens
10626	tripartite motif-containing 16	TRIM16	Homo sapiens
10155	tripartite motif-containing 28	TRIM28	Homo sapiens
23650	tripartite motif-containing 29	TRIM29	Homo sapiens
10612	tripartite motif-containing 3	TRIM3	Homo sapiens

11074	tripartite motif-containing 31	TRIM31	Homo sapiens
22954	tripartite motif-containing 32	TRIM32	Homo sapiens
51592	tripartite motif-containing 33	TRIM33	Homo sapiens
23087	tripartite motif-containing 35	TRIM35	Homo sapiens
4591	tripartite motif-containing 37	TRIM37	Homo sapiens
54765	tripartite motif-containing 44	TRIM44	Homo sapiens
7204	triple functional domain (PTPRF interacting)	TRIO	Homo sapiens
9322	thyroid hormone receptor interactor 10	TRIP10	Homo sapiens
341378	hypothetical LOC341378; thyroid hormone receptor interactor 11	TRIP11	Homo sapiens
9321	hypothetical LOC341378; thyroid hormone receptor interactor 11	TRIP11	Homo sapiens
9320	thyroid hormone receptor interactor 12	TRIP12	Homo sapiens
9319	thyroid hormone receptor interactor 13	TRIP13	Homo sapiens
9325	thyroid hormone receptor interactor 4	TRIP4	Homo sapiens
7205	thyroid hormone receptor interactor 6	TRIP6	Homo sapiens
55621	TRM1 tRNA methyltransferase 1 homolog (S. cerevisiae)	TRMT1	Homo sapiens
10024	trophinin associated protein (tastin)	TROAP	Homo sapiens
8989	transient receptor potential cation channel, subfamily A, member 1	TRPA1	Homo sapiens
7222	transient receptor potential cation channel, subfamily C, member 3	TRPC3	Homo sapiens
26133	transient receptor potential cation channel, subfamily C, member 4 associated protein	TRPC4AP	Homo sapiens
7225	transient receptor potential cation channel, subfamily C, member 6	TRPC6	Homo sapiens
4308	transient receptor potential cation channel, subfamily M, member 1	TRPM1	Homo sapiens
7227	trichorhinophalangeal syndrome I	TRPS1	Homo sapiens
55503	transient receptor potential cation channel, subfamily V, member 6	TRPV6	Homo sapiens
8295	transformation/transcription domain-associated protein	TRRAP	Homo sapiens
7248	tuberous sclerosis 1	TSC1	Homo sapiens
7249	tuberous sclerosis 2	TSC2	Homo sapiens
10102	Ts translation elongation factor, mitochondrial	TSFM	Homo sapiens
7251	tumor susceptibility gene 101	TSG101	Homo sapiens
7247	translin	TSN	Homo sapiens
7263	thiosulfate sulfurtransferase (rhodanese)	TST	Homo sapiens
7264	tissue specific transplantation antigen P35B	TSTA3	Homo sapiens
7265	tetratricopeptide repeat domain 1	TTC1	Homo sapiens
51112	tetratricopeptide repeat domain 15	TTC15	Homo sapiens
7267	tetratricopeptide repeat domain 3; tetratricopeptide repeat domain 3-like	TTC3	Homo sapiens
286495	tetratricopeptide repeat domain 3; tetratricopeptide repeat domain 3-like	TTC3	Homo sapiens
8458	transcription termination factor, RNA polymerase II	TTF2	Homo sapiens
7272	TTK protein kinase	TTK	Homo sapiens
25809	tubulin tyrosine ligase-like family, member 1	TTLL1	Homo sapiens
7273	titin	TTN	Homo sapiens
7276	transthyretin	TTR	Homo sapiens
51567	TRAF and TNF receptor associated protein	TTRAP	Homo sapiens
442308	tubulin, beta; similar to tubulin, beta 5; tubulin, beta pseudogene 2; tubulin, beta pseudogene 1	TUBB	Homo sapiens
203068	tubulin, beta; similar to tubulin, beta 5; tubulin, beta pseudogene 2;	TUBB	Homo sapiens

	tubulin, beta pseudogene 1		
92755	tubulin, beta; similar to tubulin, beta 5; tubulin, beta pseudogene 2; tubulin, beta pseudogene 1	TUBB	Homo sapiens
647000	tubulin, beta; similar to tubulin, beta 5; tubulin, beta pseudogene 2; tubulin, beta pseudogene 1	TUBB	Homo sapiens
10844	tubulin, gamma complex associated protein 2	TUBGCP2	Homo sapiens
10426	tubulin, gamma complex associated protein 3	TUBGCP3	Homo sapiens
7287	tubby like protein 1	TULP1	Homo sapiens
11334	tumor suppressor candidate 2	TUSC2	Homo sapiens
7291	twist homolog 1 (Drosophila)	TWIST1	Homo sapiens
7294	TXK tyrosine kinase	TXK	Homo sapiens
7295	thioredoxin	TXN	Homo sapiens
25828	thioredoxin 2	TXN2	Homo sapiens
10190	thioredoxin domain containing 9	TXNDC9	Homo sapiens
9352	thioredoxin-like 1	TXNL1	Homo sapiens
10907	thioredoxin-like 4A	TXNL4A	Homo sapiens
54957	thioredoxin-like 4B	TXNL4B	Homo sapiens
100130902	thioredoxin reductase 1; hypothetical LOC100130902	TXNRD1	Homo sapiens
7296	thioredoxin reductase 1; hypothetical LOC100130902	TXNRD1	Homo sapiens
7298	thymidylate synthetase	TYMS	Homo sapiens
7306	tyrosinase-related protein 1	TYRP1	Homo sapiens
7307	U2 small nuclear RNA auxiliary factor 1	U2AF1	Homo sapiens
11338	U2 small nuclear RNA auxiliary factor 2	U2AF2	Homo sapiens
55075	uveal autoantigen with coiled-coil domains and ankyrin repeats	UACA	Homo sapiens
10537	ubiquitin D	UBD	Homo sapiens
7319	ubiquitin-conjugating enzyme E2A (RAD6 homolog)	UBE2A	Homo sapiens
7320	ubiquitin-conjugating enzyme E2B (RAD6 homolog)	UBE2B	Homo sapiens
11065	ubiquitin-conjugating enzyme E2C	UBE2C	Homo sapiens
7321	ubiquitin-conjugating enzyme E2D 1 (UBC4/5 homolog, yeast)	UBE2D1	Homo sapiens
7322	ubiquitin-conjugating enzyme E2D 2 (UBC4/5 homolog, yeast)	UBE2D2	Homo sapiens
10477	ubiquitin-conjugating enzyme E2E 3 (UBC4/5 homolog, yeast)	UBE2E3	Homo sapiens
7327	ubiquitin-conjugating enzyme E2G 2 (UBC7 homolog, yeast)	UBE2G2	Homo sapiens
7328	ubiquitin-conjugating enzyme E2H (UBC8 homolog, yeast)	UBE2H	Homo sapiens
51465	ubiquitin-conjugating enzyme E2, J1 (UBC6 homolog, yeast)	UBE2J1	Homo sapiens
7332	ubiquitin-conjugating enzyme E2L 3	UBE2L3	Homo sapiens
9246	ubiquitin-conjugating enzyme E2L 6	UBE2L6	Homo sapiens
606551	ubiquitin-conjugating enzyme E2M (UBC12 homolog, yeast); ubiquitin-conjugating enzyme E2M pseudogene 1	UBE2M	Homo sapiens
9040	ubiquitin-conjugating enzyme E2M (UBC12 homolog, yeast); ubiquitin-conjugating enzyme E2M pseudogene 1	UBE2M	Homo sapiens
27338	ubiquitin-conjugating enzyme E2S	UBE2S	Homo sapiens
7336	ubiquitin-conjugating enzyme E2 variant 2	UBE2V2	Homo sapiens
89910	ubiquitin protein ligase E3B	UBE3B	Homo sapiens
10277	ubiquitination factor E4B (UFD2 homolog, yeast)	UBE4B	Homo sapiens
5412	ubiquitin-like 3	UBL3	Homo sapiens
29855	ubinuclein 1	UBN1	Homo sapiens
29978	ubiquilin 2	UBQLN2	Homo sapiens

7345	ubiquitin carboxyl-terminal esterase L1 (ubiquitin thiolesterase)	UCHL1	Homo sapiens
7347	ubiquitin carboxyl-terminal esterase L3 (ubiquitin thiolesterase)	UCHL3	Homo sapiens
7349	urocortin	UCN	Homo sapiens
114131	urocortin 3 (stresscopin)	UCN3	Homo sapiens
7351	uncoupling protein 2 (mitochondrial, proton carrier)	UCP2	Homo sapiens
7353	ubiquitin fusion degradation 1 like (yeast)	UFD1L	Homo sapiens
7357	UDP-glucose ceramide glucosyltransferase	UGCG	Homo sapiens
7363	UDP glucuronosyltransferase 2 family, polypeptide B4	UGT2B4	Homo sapiens
7368	UDP glycosyltransferase 8	UGT8	Homo sapiens
8408	unc-51-like kinase 1 (C. elegans)	ULK1	Homo sapiens
9706	unc-51-like kinase 2 (C. elegans)	ULK2	Homo sapiens
7369	uromodulin	UMOD	Homo sapiens
7372	uridine monophosphate synthetase	UMPS	Homo sapiens
9094	unc-119 homolog (C. elegans)	UNC119	Homo sapiens
8633	unc-5 homolog C (C. elegans)	UNC5C	Homo sapiens
7374	uracil-DNA glycosylase	UNG	Homo sapiens
26019	UPF2 regulator of nonsense transcripts homolog (yeast)	UPF2	Homo sapiens
65109	UPF3 regulator of nonsense transcripts homolog B (yeast)	UPF3B	Homo sapiens
11045	uroplakin 1A	UPK1A	Homo sapiens
7378	uridine phosphorylase 1	UPP1	Homo sapiens
7381	similar to ubiquinol-cytochrome c reductase binding protein; ubiquinol-cytochrome c reductase binding protein pseudogene; ubiquinol-cytochrome c reductase binding protein	UQCRB	Homo sapiens
727947	similar to ubiquinol-cytochrome c reductase binding protein; ubiquinol-cytochrome c reductase binding protein pseudogene; ubiquinol-cytochrome c reductase binding protein	UQCRB	Homo sapiens
442454	similar to ubiquinol-cytochrome c reductase binding protein; ubiquinol-cytochrome c reductase binding protein pseudogene; ubiquinol-cytochrome c reductase binding protein	UQCRB	Homo sapiens
7384	ubiquinol-cytochrome c reductase core protein I	UQCRC1	Homo sapiens
7385	ubiquinol-cytochrome c reductase core protein II	UQCRC2	Homo sapiens
100128525	ubiquinol-cytochrome c reductase, Rieske iron-sulfur polypeptide-like 1; ubiquinol-cytochrome c reductase, Rieske iron-sulfur polypeptide 1	UQCRFS1	Homo sapiens
7386	ubiquinol-cytochrome c reductase, Rieske iron-sulfur polypeptide-like 1; ubiquinol-cytochrome c reductase, Rieske iron-sulfur polypeptide 1	UQCRFS1	Homo sapiens
7389	uroporphyrinogen decarboxylase	UROD	Homo sapiens
7390	uroporphyrinogen III synthase	UROS	Homo sapiens
7392	upstream transcription factor 2, c-fos interacting	USF2	Homo sapiens
7398	ubiquitin specific peptidase 1	USP1	Homo sapiens
9100	ubiquitin specific peptidase 10	USP10	Homo sapiens
8975	ubiquitin specific peptidase 13 (isopeptidase T-3)	USP13	Homo sapiens
9097	ubiquitin specific peptidase 14 (tRNA-guanine transglycosylase)	USP14	Homo sapiens
9958	ubiquitin specific peptidase 15	USP15	Homo sapiens
10869	ubiquitin specific peptidase 19	USP19	Homo sapiens
9099	ubiquitin specific peptidase 2	USP2	Homo sapiens
10868	ubiquitin specific peptidase 20	USP20	Homo sapiens

84669	similar to TBC1 domain family, member 3; ubiquitin specific peptidase 32	USP32	Homo sapiens
645822	similar to TBC1 domain family, member 3; ubiquitin specific peptidase 32	USP32	Homo sapiens
23032	ubiquitin specific peptidase 33	USP33	Homo sapiens
9736	ubiquitin specific peptidase 34	USP34	Homo sapiens
64854	ubiquitin specific peptidase 46	USP46	Homo sapiens
8078	ubiquitin specific peptidase 5 (isopeptidase T)	USP5	Homo sapiens
9098	ubiquitin specific peptidase 6 (Tre-2 oncogene)	USP6	Homo sapiens
7874	ubiquitin specific peptidase 7 (herpes virus-associated)	USP7	Homo sapiens
9101	ubiquitin specific peptidase 8	USP8	Homo sapiens
8239	ubiquitin specific peptidase 9, X-linked	USP9X	Homo sapiens
10090	uronyl-2-sulfotransferase	UST	Homo sapiens
7402	utrophin	UTRN	Homo sapiens
7404	ubiquitously transcribed tetratricopeptide repeat gene, Y-linked	UTY	Homo sapiens
7405	UV radiation resistance associated gene	UVRAG	Homo sapiens
6843	vesicle-associated membrane protein 1 (synaptobrevin 1)	VAMP1	Homo sapiens
9341	vesicle-associated membrane protein 3 (cellubrevin)	VAMP3	Homo sapiens
8674	vesicle-associated membrane protein 4	VAMP4	Homo sapiens
10791	vesicle-associated membrane protein 5 (myobrevin)	VAMP5	Homo sapiens
8673	vesicle-associated membrane protein 8 (endobrevin)	VAMP8	Homo sapiens
9218	VAMP (vesicle-associated membrane protein)-associated protein A, 33kDa	VAPA	Homo sapiens
9217	VAMP (vesicle-associated membrane protein)-associated protein B and C	VAPB	Homo sapiens
10493	vesicle amine transport protein 1 homolog (T. californica)	VAT1	Homo sapiens
7409	vav 1 guanine nucleotide exchange factor	VAV1	Homo sapiens
7410	vav 2 guanine nucleotide exchange factor	VAV2	Homo sapiens
10451	vav 3 guanine nucleotide exchange factor	VAV3	Homo sapiens
7412	vascular cell adhesion molecule 1	VCAM1	Homo sapiens
7414	vinculin	VCL	Homo sapiens
642585	voltage-dependent anion channel 1; similar to voltage-dependent anion channel 1	VDAC1	Homo sapiens
7416	voltage-dependent anion channel 1; similar to voltage-dependent anion channel 1	VDAC1	Homo sapiens
7423	vascular endothelial growth factor B	VEGFB	Homo sapiens
51442	vestigial like 1 (Drosophila)	VGLL1	Homo sapiens
9686	vestigial like 4 (Drosophila)	VGLL4	Homo sapiens
7429	villin 1	VIL1	Homo sapiens
7431	vimentin	VIM	Homo sapiens
7432	vasoactive intestinal peptide	VIP	Homo sapiens
7433	vasoactive intestinal peptide receptor 1	VIPR1	Homo sapiens
7434	vasoactive intestinal peptide receptor 2	VIPR2	Homo sapiens
8876	vanin 1	VNN1	Homo sapiens
55823	vacuolar protein sorting 11 homolog (S. cerevisiae)	VPS11	Homo sapiens
23230	vacuolar protein sorting 13 homolog A (S. cerevisiae)	VPS13A	Homo sapiens
27072	vacuolar protein sorting 41 homolog (S. cerevisiae)	VPS41	Homo sapiens

9525	vacuolar protein sorting 4 homolog B (S. cerevisiae)	VPS4B	Homo sapiens
6293	vacuolar protein sorting 52 homolog (S. cerevisiae)	VPS52	Homo sapiens
7443	vaccinia related kinase 1	VRK1	Homo sapiens
7444	vaccinia related kinase 2	VRK2	Homo sapiens
7447	visinin-like 1	VSNL1	Homo sapiens
7450	von Willebrand factor	VWF	Homo sapiens
7453	tryptophanyl-tRNA synthetase	WARS	Homo sapiens
7454	Wiskott-Aldrich syndrome (eczema-thrombocytopenia)	WAS	Homo sapiens
8936	WAS protein family, member 1	WASF1	Homo sapiens
10810	WAS protein family, member 3	WASF3	Homo sapiens
8976	Wiskott-Aldrich syndrome-like	WASL	Homo sapiens
23558	WW domain binding protein 2	WBP2	Homo sapiens
11193	WW domain binding protein 4 (formin binding protein 21)	WBP4	Homo sapiens
23001	WD repeat and FYVE domain containing 3	WDFY3	Homo sapiens
11169	WD repeat and HMG-box DNA binding protein 1	WDHD1	Homo sapiens
57418	WD repeat domain 18	WDR18	Homo sapiens
11152	WD repeat domain 45	WDR45	Homo sapiens
58189	WAP four-disulfide core domain 1	WFDC1	Homo sapiens
7466	Wolfram syndrome 1 (wolframin)	WFS1	Homo sapiens
11197	WNT inhibitory factor 1	WIF1	Homo sapiens
8840	WNT1 inducible signaling pathway protein 1	WISP1	Homo sapiens
8839	WNT1 inducible signaling pathway protein 2	WISP2	Homo sapiens
80326	wingless-type MMTV integration site family, member 10A	WNT10A	Homo sapiens
7480	wingless-type MMTV integration site family, member 10B	WNT10B	Homo sapiens
7481	wingless-type MMTV integration site family, member 11	WNT11	Homo sapiens
51384	wingless-type MMTV integration site family, member 16	WNT16	Homo sapiens
7472	wingless-type MMTV integration site family member 2	WNT2	Homo sapiens
7473	wingless-type MMTV integration site family, member 3	WNT3	Homo sapiens
54361	wingless-type MMTV integration site family, member 4	WNT4	Homo sapiens
7475	wingless-type MMTV integration site family, member 6	WNT6	Homo sapiens
7485	tryptophan rich basic protein	WRB	Homo sapiens
7486	similar to Werner syndrome protein; Werner syndrome, RecQ helicase-like	WRN	Homo sapiens
652522	similar to Werner syndrome protein; Werner syndrome, RecQ helicase-like	WRN	Homo sapiens
26118	WD repeat and SOCS box-containing 1	WSB1	Homo sapiens
7490	Wilms tumor 1	WT1	Homo sapiens
9589	Wilms tumor 1 associated protein	WTAP	Homo sapiens
51741	WW domain containing oxidoreductase	WWOX	Homo sapiens
11059	WW domain containing E3 ubiquitin protein ligase 1	WWP1	Homo sapiens
11060	WW domain containing E3 ubiquitin protein ligase 2	WWP2	Homo sapiens
7494	X-box binding protein 1	XBP1	Homo sapiens
6375	chemokine (C motif) ligand 1	XCL1	Homo sapiens
2829	chemokine (C motif) receptor 1	XCR1	Homo sapiens
7504	X-linked Kx blood group (McLeod syndrome)	XK	Homo sapiens
7507	xeroderma pigmentosum, complementation group A	XPA	Homo sapiens

7508	xeroderma pigmentosum, complementation group C	XPC	Homo sapiens
7511	X-prolyl aminopeptidase (aminopeptidase P) 1, soluble	XPNPEP1	Homo sapiens
7514	exportin 1 (CRM1 homolog, yeast)	XPO1	Homo sapiens
57510	exportin 5	XPO5	Homo sapiens
7515	X-ray repair complementing defective repair in Chinese hamster cells 1	XRCC1	Homo sapiens
7518	X-ray repair complementing defective repair in Chinese hamster cells 4	XRCC4	Homo sapiens
7520	X-ray repair complementing defective repair in Chinese hamster cells 5 (double-strand-break rejoining)	XRCC5	Homo sapiens
54464	5'-3' exoribonuclease 1	XRN1	Homo sapiens
22803	5'-3' exoribonuclease 2	XRN2	Homo sapiens
64131	xylosyltransferase I	XYLT1	Homo sapiens
10138	YY1 associated factor 2	YAF2	Homo sapiens
10413	Yes-associated protein 1, 65kDa	YAP1	Homo sapiens
8565	tyrosyl-tRNA synthetase	YARS	Homo sapiens
55689	YEATS domain containing 2	YEATS2	Homo sapiens
7525	v-yes-1 Yamaguchi sarcoma viral oncogene homolog 1	YES1	Homo sapiens
7529	tyrosine 3-monooxygenase/tryptophan 5-monooxygenase activation protein, beta polypeptide	YWHAB	Homo sapiens
440917	similar to 14-3-3 protein epsilon (14-3-3E) (Mitochondrial import stimulation factor L subunit) (MSF L); tyrosine 3-monooxygenase/tryptophan 5-monooxygenase activation protein, epsilon polypeptide	YWHAE	Homo sapiens
7531	similar to 14-3-3 protein epsilon (14-3-3E) (Mitochondrial import stimulation factor L subunit) (MSF L); tyrosine 3-monooxygenase/tryptophan 5-monooxygenase activation protein, epsilon polypeptide	YWHAE	Homo sapiens
7533	tyrosine 3-monooxygenase/tryptophan 5-monooxygenase activation protein, eta polypeptide	YWHAH	Homo sapiens
10971	tyrosine 3-monooxygenase/tryptophan 5-monooxygenase activation protein, theta polypeptide	YWHAQ	Homo sapiens
7534	tyrosine 3-monooxygenase/tryptophan 5-monooxygenase activation protein, zeta polypeptide	YWHAZ	Homo sapiens
7528	YY1 transcription factor	YY1	Homo sapiens
9889	zinc finger, BED-type containing 4	ZBED4	Homo sapiens
27107	zinc finger and BTB domain containing 11	ZBTB11	Homo sapiens
7704	zinc finger and BTB domain containing 16	ZBTB16	Homo sapiens
7709	zinc finger and BTB domain containing 17	ZBTB17	Homo sapiens
10009	zinc finger and BTB domain containing 33	ZBTB33	Homo sapiens
7541	zinc finger protein 161 homolog (mouse)	ZFP161	Homo sapiens
7538	zinc finger protein 36, C3H type, homolog (mouse)	ZFP36	Homo sapiens
7539	zinc finger protein 37 homolog (mouse)	ZFP37	Homo sapiens
7542	zinc finger protein-like 1	ZFPL1	Homo sapiens
9372	zinc finger, FYVE domain containing 9	ZFYVE9	Homo sapiens
7545	Zic family member 1 (odd-paired homolog, Drosophila)	ZIC1	Homo sapiens
51364	zinc finger, MYND-type containing 10	ZMYND10	Homo sapiens
10771	zinc finger, MYND domain containing 11	ZMYND11	Homo sapiens
7692	zinc finger protein 133	ZNF133	Homo sapiens

7694	zinc finger protein 135	ZNF135	Homo sapiens
7696	zinc finger protein 137	ZNF137	Homo sapiens
7701	zinc finger protein 142	ZNF142	Homo sapiens
7705	zinc finger protein 146	ZNF146	Homo sapiens
7707	zinc finger protein 148	ZNF148	Homo sapiens
7718	zinc finger protein 165	ZNF165	Homo sapiens
7727	zinc finger protein 174	ZNF174	Homo sapiens
7738	zinc finger protein 184	ZNF184	Homo sapiens
7741	zinc finger protein 187	ZNF187	Homo sapiens
7746	zinc finger protein 193	ZNF193	Homo sapiens
10168	zinc finger protein 197	ZNF197	Homo sapiens
7752	zinc finger protein 200	ZNF200	Homo sapiens
10520	zinc finger protein 211	ZNF211	Homo sapiens
7764	zinc finger protein 217	ZNF217	Homo sapiens
7570	zinc finger protein 22 (KOX 15)	ZNF22	Homo sapiens
7773	zinc finger protein 230	ZNF230	Homo sapiens
10472	zinc finger protein 238	ZNF238	Homo sapiens
7572	zinc finger protein 24	ZNF24	Homo sapiens
10127	zinc finger protein 263	ZNF263	Homo sapiens
9422	zinc finger protein 264	ZNF264	Homo sapiens
10781	zinc finger protein 266	ZNF266	Homo sapiens
10782	zinc finger protein 274	ZNF274	Homo sapiens
23528	zinc finger protein 281	ZNF281	Homo sapiens
7551	zinc finger protein 3	ZNF3	Homo sapiens
24149	zinc finger protein 318	ZNF318	Homo sapiens
7580	zinc finger protein 32	ZNF32	Homo sapiens
25799	zinc finger protein 324	ZNF324	Homo sapiens
27309	zinc finger protein 330	ZNF330	Homo sapiens
59348	zinc finger protein 350	ZNF350	Homo sapiens
57862	zinc finger protein 410	ZNF410	Homo sapiens
23090	zinc finger protein 423	ZNF423	Homo sapiens
51710	zinc finger protein 44	ZNF44	Homo sapiens
26036	zinc finger protein 451	ZNF451	Homo sapiens
58499	zinc finger protein 462	ZNF462	Homo sapiens
256051	zinc finger protein 549	ZNF549	Homo sapiens
27332	zinc finger protein 638	ZNF638	Homo sapiens
7553	zinc finger protein 7	ZNF7	Homo sapiens
7629	zinc finger protein 76 (expressed in testis)	ZNF76	Homo sapiens
7633	zinc finger protein 79	ZNF79	Homo sapiens
7554	zinc finger protein 8	ZNF8	Homo sapiens
11055	zona pellucida binding protein	ZPBP	Homo sapiens
9183	ZW10, kinetochore associated, homolog (Drosophila)	ZW10	Homo sapiens
7791	zyxin	ZYX	Homo sapiens
26009	zinc finger, ZZ-type containing 3	ZZZ3	Homo sapiens
